# Palladium-catalyzed benzocyclization reactions of quinoline-2-carboxamides via sequential C–H/N–H functionalization

**DOI:** 10.3762/bjoc.22.71

**Published:** 2026-06-09

**Authors:** Shoichi Sugita, Kentaro Okano, Atsunori Mori

**Affiliations:** 1 Research Center for Membrane and Film Technology, Kobe University, 1-1 Rokkodai, Nada, Kobe 657-8501, Japan; 2 Kobe Pharmaceutical University, 4-19-1 Motoyamakita-machi, Higashinada, Kobe 658-8558, Japan; 3 Department of Chemical Science and Engineering, Kobe University, 1-1 Rokkodai, Nada, Kobe 657-8501, Japan

**Keywords:** C–H activation, chemodivergent synthesis, C–N coupling, fused-ring system, sequential reaction

## Abstract

A novel benzocyclization protocol has been developed for the synthesis of quinoline-fused lactams by palladium-catalyzed sequential C–H/N–H functionalization of quinoline-2-carboxamides and 1,2-dihaloarenes. The reaction proceeds at the C–H bond on the quinoline adjacent to the amide group and at the amide N–H bond in the presence of 10 mol % Pd(OAc)_2_ in *o*-xylene as a solvent to afford the cyclized product in 34% yield. The yield increases to 81% when the reaction is carried out with 80 mol % P(4-MeOC_6_H_4_)_3_ as a ligand and with an increased catalyst loading of 20 mol %. The reaction affords lactams in up to 83% yield using amides containing various functional groups and substituted 1-bromo-2-iodobenzenes. Furthermore, 1,2-dibromo heteroarenes, such as benzothiophene and pyridine, undergo annulation to give the corresponding heterocycle-fused compounds. The high chemoselectivity of the 1,2-dihaloarene functional groups is confirmed in this reaction, thus enabling divergent synthesis of various multifused heterocyclic systems.

## Introduction

Cyclic structures comprising nitrogen-containing multifused rings are extremely important because such heterocycle-fused cyclic structures [1–2] are found in various advanced materials [3–4] and biologically essential molecules [5–7]. Quinoline is a particularly intriguing moiety in biologically active compounds (e.g., natural products used for medicines, quinine, and quinidine [5–7]) and synthesized pharmaceutical agents (e.g., quinolone antibiotics [8]). Moreover, quinoline-2-carboxamide derivatives are used as ligands in organic synthesis owing to their high metal affinity [9–10]. It is therefore expected that annulation of the amide moiety in quinoline-2-carboxamides would extend their functionality as biologically active structures, ligands, and extractants.

**Scheme 1:**
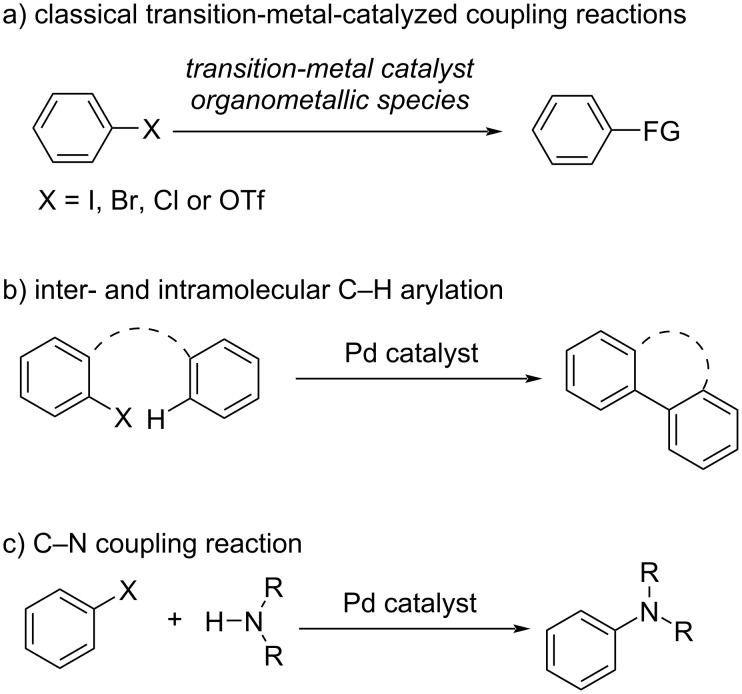
Various transition-metal-catalyzed coupling reactions involving aryl halides.

Transition-metal-catalyzed coupling reactions are crucial for constructing carbon–carbon and carbon–heteroatom bonds. The classical coupling reactions, such as Kumada–Tamao–Corriu coupling [11–13], Sonogashira coupling [14–15], Negishi coupling [16–17], Migita–Kosugi–Stille coupling [18–20], Suzuki–Miyaura coupling [21–22], and Hiyama coupling [23–24] involve carbon–halogen and carbon–metal species (Scheme 1a). Fagnou and co-workers reported direct arylation reactions with palladium(II) acetate to synthesize biaryl compounds via concerted metalation–deprotonation (CMD; Scheme 1b) [25–27]. Buchwald, Hartwig, and co-workers explored carbon–nitrogen coupling reactions that allow facile preparation of aromatic amines (Scheme 1c) [28–31]. In general, intramolecular C–H arylation reactions in the presence of a transition-metal catalyst have been reported extensively in recent years. These reactions enable an efficient formation of fused-ring systems [32–35]. Additionally, intramolecular C–H arylation reactions with *N*-heteroaromatics can be used to synthesize various functional molecules that serve as ligands for metal extraction [36–41].

**Scheme 2:**
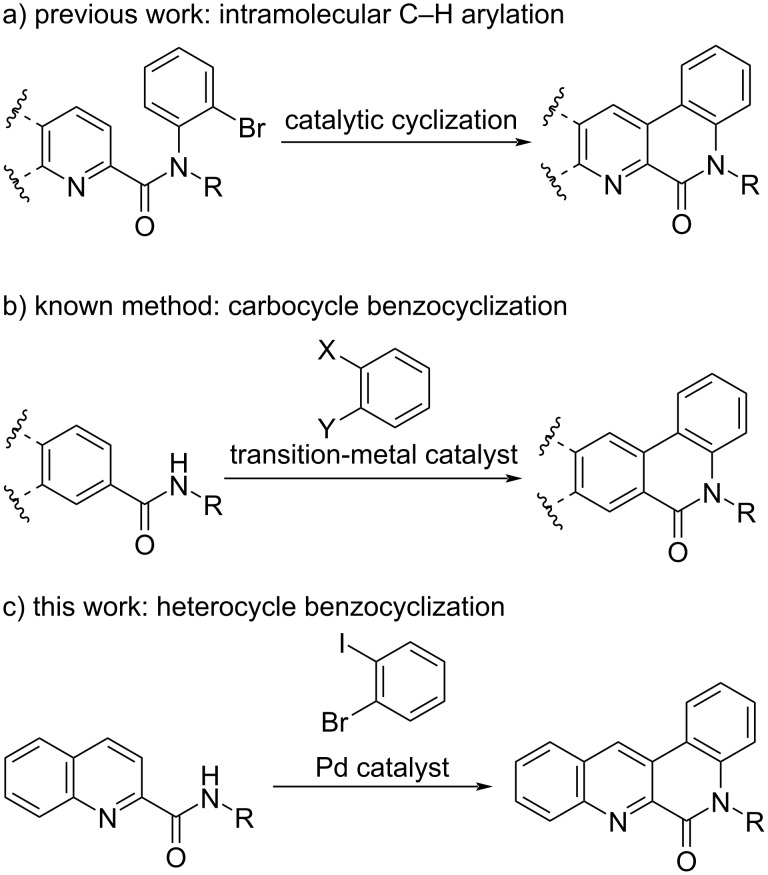
Synthetic strategy for preparing fused lactams.

Our group developed a cyclization reaction for the intramolecular C–H arylation with *N*-heterocycles, such as phenanthroline and quinoline, containing amide groups (Scheme 2a) [38,40]. These reactions efficiently provide the corresponding annulation products; however, it remains difficult to selectively obtain a variety of substituent positions following cyclization. Moreover, these methodologies necessitate the preparation of annulation precursors through *o*-brominated aniline derivatives [36–41]. Furthermore, the reported methodologies for synthesizing *N*-heterocycle-fused lactams are characterized by either low efficiency or protracted processes [42–43]. On the other hand, benzocyclization reactions of aryl carboxamides offer a method for synthesizing chemodivergent products from a single substrate. Importantly, these reactions are controlled by the different reactivities of the halogen atoms in the reagent structures. Several carbocycle C–H/N–H activated benzocyclizations have already been reported [44–54], although the reaction mechanism with π-deficient *N*-heteroaromatics has not been elucidated (Scheme 2b). It is therefore valuable to investigate the differences in reactivity between C–H and N–H for intermolecular arylation in the presence of transition-metal catalysts. This can reveal their selectivity in terms of reaction position(s) in chemodivergent synthesis (Scheme 2c). The present report explores benzocyclization reactions involving sequential C–H/N–H functionalization by a palladium catalyst.

## Results and Discussion

First, the C–H/N–H annulation reaction between quinoline-2-carboxamide **1a** and 1-bromo-2-iodobenzene (**2a**) was tested. When **1a** was treated with 1.0 equiv of **2a**, 10 mol % Pd(OAc)_2,_ 40 mol % PPh_3_, and 3.0 equiv of Cs_2_CO_3_ in dimethylformamide (DMF) at 140 °C, the desired lactam **3aa** was obtained in 19% yield (Table 1, entry 1). This result indicated that the anticipated C–H and N–H intermolecular–intramolecular coupling reactions occurred. When PdCl_2_(PPh_3_)_2_ was used instead of Pd(OAc)_2_ as a catalyst in the absence of PPh_3_, **3aa** was afforded in 10% yield (Table 1, entry 2). When Pd(OCOCF_3_)_2_ was used as the catalyst, the desired product was obtained in 39% yield (Table 1, entry 3). Using any base other than Cs_2_CO_3_ resulted in lower yields, thus confirming that Cs_2_CO_3_ was the optimal base for this reaction (Table 1, entries 4–6). Increasing the concentration of **1a** from 0.1 to 1.0 M led to an increased yield, even if Pd(OAc)_2_ was used as the catalyst (Table 1, entry 7). When the reaction temperature was reduced to 110 °C, 120 °C, or 130 °C, product **3aa** was obtained in lower yields in all cases compared with entry 7 (Table 1, entries 8–10). Using *o*-xylene as the solvent gave results similar to those obtained with DMF (Table 1, entry 11). Notably, the reaction using *o*-xylene as the solvent supported a good mass balance of **1a** and **3aa**. Therefore, the coupling reaction conditions were further optimized using *o*-xylene as the solvent. The ligand effect was examined, and the results indicated that electron-donating ligands were more efficient than electron-withdrawing ligands, particularly in the context of tris(4-methoxyphenyl)phosphine (P(4-MeOC_6_H_4_)_3_) (Table 1, entries 12–15). Additionally, the annulation product was detected in less than 1% yield in the presence of bulky ligands, such as tri(*o*-tolyl)phosphine (Table 1, entry 16). Next, the relative amounts of reagents were optimized for this annulation reaction (Table 1, entries 17–19). Ultimately, it was determined that the optimal reagent quantities were 20 mol % Pd(OAc)_2_, 80 mol % P(4-MeOC_6_H_4_)_3_, and 2.0 equiv of **2a**, which afforded the product **3aa** in 81% yield (Table 1, entry 19).

**Table 1:** Investigation of this C–H/N–H functionalization reaction conditions.

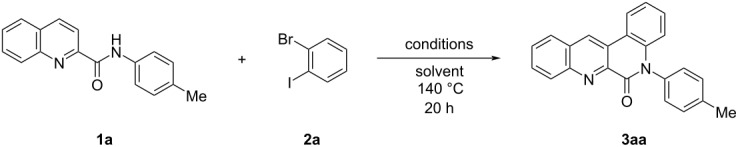							

Entry	Catalyst (mol %)	Ligand (mol %)	Base (equiv)	**2a** (equiv)	Solvent (M)	Yield (%)^a^	

						**3aa**	**1a**

1	Pd(OAc)_2_ (10)	PPh_3_ (40)	Cs_2_CO_3_ (3.0)	1.0	DMF (0.1)	19	52
2	PdCl_2_(PPh_3_)_2_ (10)	none	Cs_2_CO_3_ (3.0)	1.0	DMF (0.1)	10	85
3	Pd(OCOCF_3_)_2_ (10)	PPh_3_ (40)	Cs_2_CO_3_ (3.0)	1.0	DMF (0.1)	39	32
4	Pd(OAc)_2_ (10)	PPh_3_ (40)	K_2_CO_3_ (3.0)	1.0	DMF (0.1)	6	72
5	Pd(OAc)_2_ (10)	PPh_3_ (40)	*t-*BuOK (3.0)	1.0	DMF (0.1)	0	quant.
6	Pd(OAc)_2_ (10)	PPh_3_ (40)	*t-*BuONa (3.0)	1.0	DMF (0.1)	9	63
7	Pd(OAc)_2_ (10)	PPh_3_ (40)	Cs_2_CO_3_ (3.0)	1.0	DMF (1.0)	46	28
8^b^	Pd(OAc)_2_ (10)	PPh_3_ (40)	Cs_2_CO_3_ (3.0)	1.0	DMF (1.0)	4	64
9^c^	Pd(OAc)_2_ (10)	PPh_3_ (40)	Cs_2_CO_3_ (3.0)	1.0	DMF (1.0)	19	61
10^d^	Pd(OAc)_2_ (10)	PPh_3_ (40)	Cs_2_CO_3_ (3.0)	1.0	DMF (1.0)	40	32
11	Pd(OAc)_2_ (10)	PPh_3_ (40)	Cs_2_CO_3_ (3.0)	1.0	*o*-xylene (1.0)	34	58
12	Pd(OAc)_2_ (10)	PPh_3_ (100)	Cs_2_CO_3_ (3.0)	1.0	*o*-xylene (1.0)	39	30
13	Pd(OAc)_2_ (10)	P(*p*-tolyl)_3_ (40)	Cs_2_CO_3_ (3.0)	1.0	*o*-xylene (1.0)	35	30
14	Pd(OAc)_2_ (10)	P(4-MeOC_6_H_4_)_3_ (40)	Cs_2_CO_3_ (3.0)	1.0	*o*-xylene (1.0)	44	33
15	Pd(OAc)_2_ (10)	P(4-FC_6_H_4_)_3_ (40)	Cs_2_CO_3_ (3.0)	1.0	*o*-xylene (1.0)	21	55
16	Pd(OAc)_2_ (10)	P(*o*-tolyl)_3_ (40)	Cs_2_CO_3_ (3.0)	1.0	*o*-xylene (1.0)	<1	quant.
17^e^	Pd(OAc)_2_ (10)	P(4-MeOC_6_H_4_)_3_ (40)	Cs_2_CO_3_ (3.0)	1.0 + 1.0	*o*-xylene (1.0)	53	15
18	Pd(OAc)_2_ (10)	P(4-MeOC_6_H_4_)_3_ (80)	Cs_2_CO_3_ (3.0)	2.0	*o*-xylene (1.0)	45	34
19	Pd(OAc)_2_ (20)	P(4-MeOC_6_H_4_)_3_ (80)	Cs_2_CO_3_ (3.0)	2.0	*o*-xylene (1.0)	81^f^	N.D.^g^

^a^The yields were determined by ^1^H NMR spectroscopy using 1,1,2,2-tetrachloroethane as an internal standard; ^b^the reaction was performed at 110 °C; ^c^the reaction was performed at 120 °C; ^d^the reaction was performed at 130 °C; ^e^after stirring for 20 hours at 140 °C, an additional equivalent of **2a** was added to the reaction system, and the reaction was allowed to proceed for another 20 hours at 140 °C; ^f^isolated yield; ^g^not detected.

The optimized conditions were used to investigate the substrate scope of this C–H/N–H functionalized reaction. First, the substituents at the amido group of quinoline-2-carboxamides **1** were examined (Table 2). The C–H/N–H functionalization reactions afforded good to excellent yields, regardless of the presence of a non-substituted phenyl group or various substituents at the 4-position of the phenyl moiety (Table 2, entries 1–5). In contrast, the quinoline-2-carboxamide containing a 4-nitrophenyl group gave the corresponding product in a low yield (Table 2, entry 6). This result indicated that an increased acidity of the N–H proton of the amides, caused by substituent groups, could potentially inhibit the annulation reaction. Similarly, amides bearing mesityl, 2-nitrophenyl, or 2-methoxyphenyl groups afforded the corresponding products in low yields due to steric hindrance (Table 2, entries 7–9). The reaction with benzyl-substituted carboxamide also resulted in a low yield owing to the lower acidity of the amide proton compared with aromatic amides (Table 2, entry 10). Based on these results quinoline carboxamides **1** bearing aromatic substituents, which avoid steric effects and induce moderate acidity of the amide N–H proton, exhibit higher propensity for reaction in comparison to aliphatic amides.

**Table 2:** Investigation of the substrate scope and substituent limitations of amide groups for the C–H/N–H functionalization reaction.

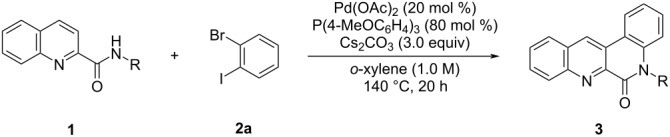		

Entry	R	Yield (%)

1	Ph	81 (**3ba**)
2	4-MeOC_6_H_4_	68 (**3ca**)
3	4-*t*-BuC_6_H_4_	72 (**3da**)
4	4-CNC_6_H_4_	67 (**3ea**)
5	4-ClC_6_H_4_	75 (**3fa**)
6	4-NO_2_C_6_H_4_	18 (**3ga**)
7	mesityl	21 (**3ha**)
8	2-NO_2_C_6_H_4_	14 (**3ia**)
9^a^	2-MeOC_6_H_4_	17 (**3ja**)
10^a^	Bn	21 (**3ka**)

^a^The reaction time was prolonged to 96 h.

Next, the C–H/N–H functionalization reaction was evaluated using various 1,2-dihaloarenes **2** (Table 3). When 1-bromo-2-iodo-5-methylbenzene (**2b**) was reacted with **1a**, the corresponding cycloadduct **3ab** was obtained in good yield (Table 3, entry 1). In contrast, 1-bromo-2-iodo-4-methylbenzene (**2c**) afforded the annulation product **3ac** in low yield (Table 3, entry 2). However, the desired products **3ab** and **3ac** were obtained in excellent yields when the reaction period was prolonged from 20 to 96 h. Although relatively low product yields were obtained when using dihaloarenes **2d** and **2e**, both containing a *tert*-butyl group, after 20 h, the corresponding products **3ad** and **3ae** were obtained in good to excellent yields when the reaction time was prolonged to 96 h (Table 3, entries 3 and 4). Similar trends were observed in the reactions involving 2-bromo-1-iodo-4-methoxybenzene (**2f**) and 1-bromo-2-iodo-4-methoxybenzene (**1g**) (Table 3, entries 5 and 6). The oxidative addition of the palladium catalyst to the C–I bond in these electron-donating group-containing bromo(iodo)benzenes occurred slowly, requiring an extended reaction time to reach completion. Particularly, when 2-bromo-1-iodobenzenes containing electron-donating groups at the C4 position were utilized in this reaction, the yields of the products **3** would be decreased due to the increased electron density of bromo(iodo)benzenes which results in an inhibition of the catalyst’s oxidative addition to the C–I bond. Notably, the substrates containing electron-withdrawing groups, such as cyano or nitro groups, resulted in low yields, even after longer reaction times (Table 3, entries 7 and 8). These results were attributed to homo-coupling of 1-bromo-2-iodobenzenes or protonation of activated haloarenes and deactivation of the palladium catalyst. It is therefore indicated that bromo(iodo)benzenes with high electron density utilized in this reaction require significantly longer reaction times to achieve good to excellent yields of products **3**, whereas the use of bromo(iodo)benzenes with low electron density leads to their decomposition, which results in decreased yields of product **3**. Finally, the coupling reactions with heteroarenes, such as 2,3-dibromobenzothiophene (**2j**) and 2,3-dibromopyridine (**2k**), afforded the corresponding products in moderate yields with high regioselectivity (Table 3, entries 9 and 10).

**Table 3:** Investigation of the dihaloarene substrate scope for the C–H/N–H functionalization reaction.

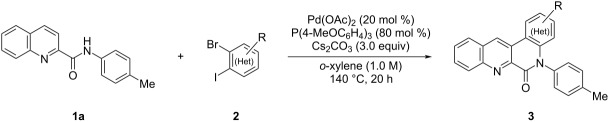		

Entry	**2**	Product (yield, %)

1	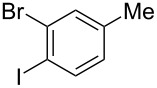 **2b**	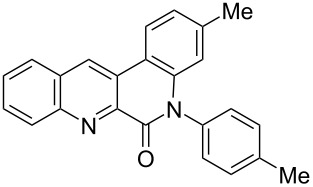 **3ab** (63, 79^a^)
2	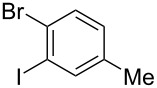 **2c**	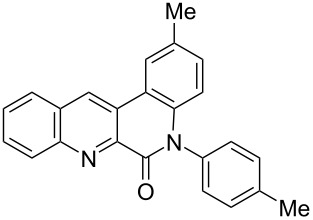 **3ac** (17, 83^a^)
3	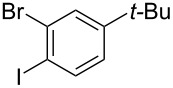 **2d**	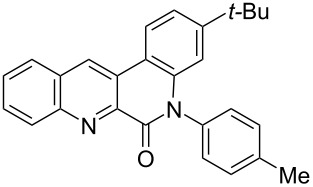 **3ad** (28, 55^a^)
4	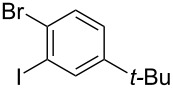 **2e**	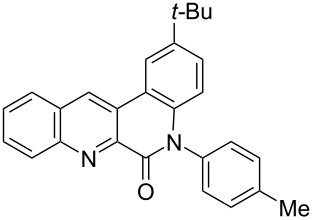 **3ae** (18, 81^a^)
5	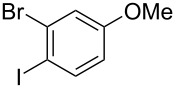 **2f**	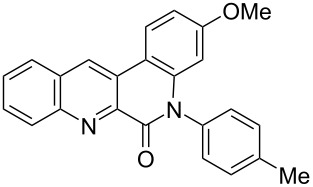 **3af** (32, 51^a^)
6	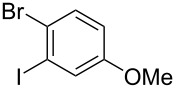 **2g**	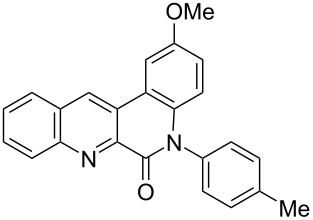 **3ag** (24, 82^a^)
7	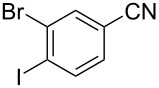 **2h**	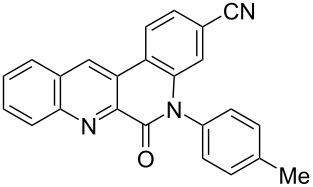 **3ah** (26, 21^a^)
8	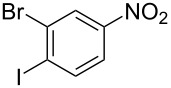 **2i**	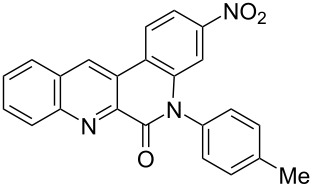 **3ai** (7, 7^a^)
9	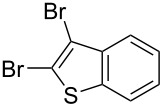 **2j**	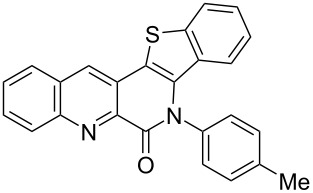 **3aj** (21)
10	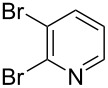 **2k**	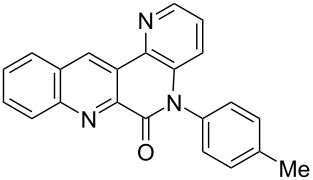 **3ak** (31)

^a^These yields were obtained when the reaction time was prolonged to 96 h.

**Figure 1:**
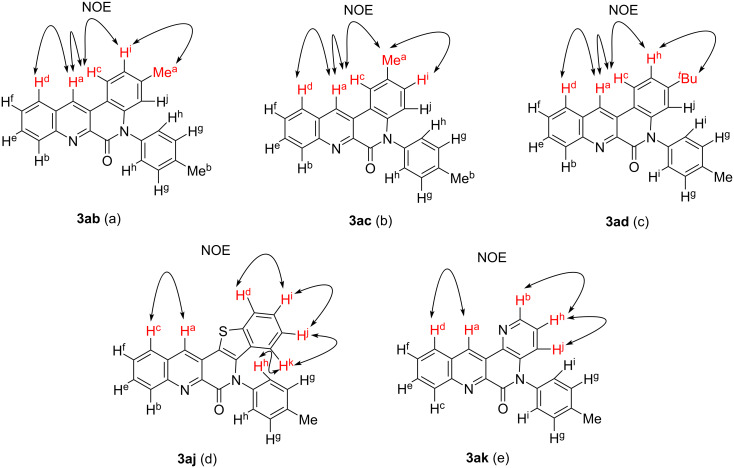
Nuclear Overhauser effect (NOE) correlations in products (a) **3ab**, (b) **3ac**, (c) **3ad**, (d) **3aj**, and (e) **3ak**.

**Scheme 3:**
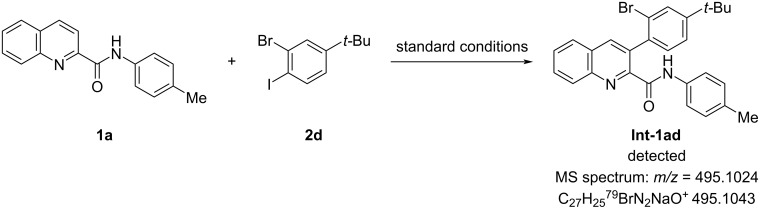
Detection of the intermediate **Int-1ad** in the annulation reaction of **1a** with 1-bromo-5-*tert*-butyl-2-iodobenzene (**2d**).

The structures of the isomeric products were confirmed by nuclear Overhauser effect spectroscopy (NOESY, see Supporting Information File 1). First, the structures of lactams **3ab**, **3ac**, and **3ad** were determined (Figure 1a–c, respectively). The products **3ab** and **3ac**, which were synthesized from **2b** and **2c**, were detected as the corresponding isomers, with the methyl group attached to the C3 or C2 position of products **3ab** and **3ac**, respectively. In the case of product **3ad**, derived from 1-bromo-2-iodobenzene **2d**, which contains a *tert*-butyl group in the C5 position, a similar substitution pattern was detected as for compound **3ad** derived from 1-bromo-2-iodo-5-methylbenzene (**2b**). Additionally, during the coupling reaction carried out with 5-*tert*-butyl-1-bromo-2-iodobenzene, **Int-1ad** species was detected as a reaction intermediate, as confirmed by ^1^H NMR and mass spectra (Scheme 3; see also Supporting Information File 1). This result indicated that the C–C bond was formed first in the reaction. The structures of other products were inferred from their ^1^H NMR spectra, while the structures of products **3aj** and **3ak** were also determined by NOESY (Figure 1d and e).

**Scheme 4:**
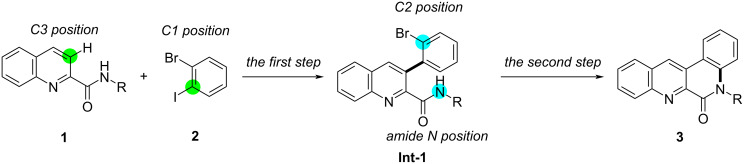
Stepwise formation of C–C and C–N bonds during the annulation reaction.

**Scheme 5:**
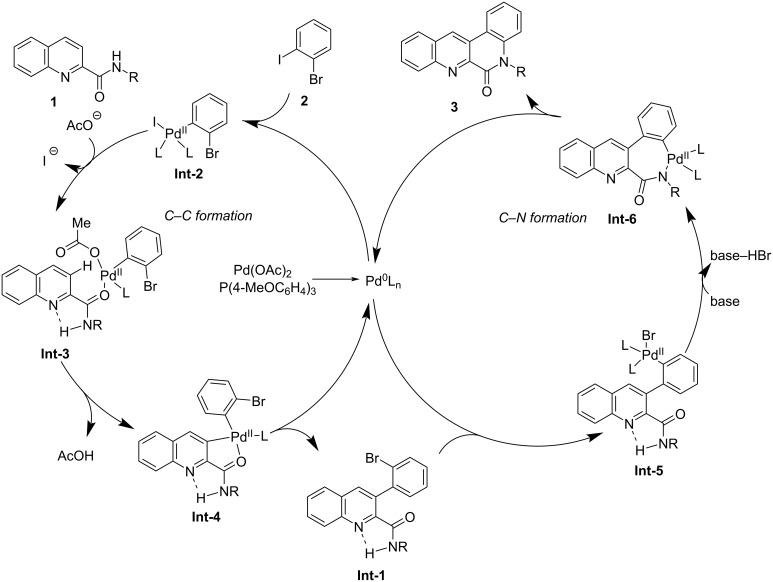
Plausible reaction mechanism for the sequential C–H/N–H functionalization reaction.

These results suggested that the carbon–iodine bond in 1-bromo-2-iodobenzene was involved in the formation of the C–C bond, while the carbon–bromine bond was involved in the formation of the C–N bond (Scheme 4). Accordingly, a plausible reaction mechanism is proposed in Scheme 5. First, the activated palladium(0) catalyst is inserted into the carbon–iodine bond of the 1-bromo-2-iodoarene **2** via oxidative addition. The intermediate **Int-2** then undergoes ligand exchange from iodine and phosphine to acetate and quinoline-2-carboxamide to generate intermediate **Int-3**. Then, through a concerted metalation-deprotonation (CMD) process, a carbon–palladium bond is formed to give the palladacycle intermediate **Int-4**. Next, reductive elimination between the quinoline and arene moieties forms the C–C bond to give intermediate **Int-1** and regenerates the palladium(0) catalyst. Another oxidative addition to the carbon–bromine bond of **Int-1** generates **Int-5**, and a nitrogen–palladium bond is formed to afford the seven membered palladacycle intermediate **Int-6**. Finally, this intermediate undergoes reductive elimination between the nitrogen and carbon atoms to provide the lactam product **3**.

## Conclusion

This study explored a novel palladium-catalyzed C–H/N–H activated annulation reaction for the synthesis of quinoline-fused lactams. The annulation reaction afforded the desired products in up to 83% yield. The reaction displayed broad tolerance for various substituents. Moreover, the reaction demonstrated high chemoselectivity because it proceeds via initial C–C bond formation at C–I, followed by C–N bond formation at C–Br. Thus, the positions of substituents on the products are controlled based on the position of the substituents in the 1-bromo-2-iodobenzene substrates, thus providing facile and efficient access to chemodivergent products. The developed reaction protocol is expected to be applicable to the synthesis of functional materials and bioactive molecules.

## Supporting Information

File 1 Experimental details and copies of ^1^H and ^13^C{^1^H} NMR spectra.

## Data Availability

Additional research data generated and analyzed during this study is not shared.

## References

[1] Ganguly A K, Wang C H, David M, Bartner P, Chan T M (2002). Tetrahedron Lett.

[2] Das S, Kundu S, Metya A, Maji M S (2024). Chem Sci.

[3] Guo Z, Yan C, Zhu W-H (2020). Angew Chem, Int Ed.

[4] Yu C P, Yamamoto A, Kumagai S, Takeya J, Okamoto T (2023). Angew Chem, Int Ed.

[5] Michael J P (2002). Nat Prod Rep.

[6] Shang X-F, Morris‐Natschke S L, Liu Y-Q, Guo X, Xu X-S, Goto M, Li J-C, Yang G-Z, Lee K-H (2018). Med Res Rev.

[7] Yadav T T, Murahari M, Peters G J, YC M (2022). Eur J Med Chem.

[8] Pham T D M, Ziora Z M, Blaskovich M A T (2019). Med Chem Commun.

[9] Patra S, Hirose T, Himeda Y (2025). Inorg Chim Acta.

[10] Kumar R, Majumder S, Singh C P, Samanta C, Newalkar B L, Krishnamurty S, Das R K (2025). ChemSusChem.

[11] Tamao K, Sumitani K, Kumada M (1972). J Am Chem Soc.

[12] Corriu R J P, Masse J P (1972). J Chem Soc, Chem Commun.

[13] Heravi M M, Zadsirjan V, Hajiabbasi P, Hamidi H (2019). Monatsh Chem.

[14] Sonogashira K, Tohda Y, Hagihara N (1975). Tetrahedron Lett.

[15] Chinchilla R, Nájera C (2011). Chem Soc Rev.

[16] King A O, Okukado N, Negishi E-i (1977). J Chem Soc, Chem Commun.

[17] Muzammil, Zahoor A F, Parveen B, Javed S, Akhtar R, Tabassum S (2024). Chem Pap.

[18] Kosugi M, Sasazawa K, Shimizu Y, Migita T (1977). Chem Lett.

[19] Milstein D, Stille J K (1978). J Am Chem Soc.

[20] Srivastav N, Singh R, Kaur V (2015). RSC Adv.

[21] Miyaura N, Suzuki A (1979). J Chem Soc, Chem Commun.

[22] Lennox A J J, Lloyd-Jones G C (2014). Chem Soc Rev.

[23] Hatanaka Y, Hiyama T (1988). J Org Chem.

[24] Hiyama T, Minami Y, Mori A, Hiyama T, Oestreich M (2019). Transition-Metal-Catalyzed Cross-coupling of Organosilicon Compounds. Organosilicon Chemistry.

[25] Campeau L-C, Parisien M, Leblanc M, Fagnou K (2004). J Am Chem Soc.

[26] Campeau L-C, Parisien M, Jean A, Fagnou K (2006). J Am Chem Soc.

[27] Liégault B, Petrov I, Gorelsky S I, Fagnou K (2010). J Org Chem.

[28] Paul F, Patt J, Hartwig J F (1994). J Am Chem Soc.

[29] Guram A S, Buchwald S L (1994). J Am Chem Soc.

[30] Yamamoto T, Nishiyama M, Koie Y (1998). Tetrahedron Lett.

[31] Dorel R, Grugel C P, Haydl A M (2019). Angew Chem, Int Ed.

[32] de Groot L H M, García‐Mateos C, Johnson C E, Freyr Hlynsson V, Sharma A K, Lomoth R, Wärnmark K (2025). Chem – Eur J.

[33] Brufani G, Bazzica E, Gu Y, Mauriello F, Vaccaro L (2026). Chin Chem Lett.

[34] Langer P (2024). Synlett.

[35] Langer P (2022). Synlett.

[36] Ustynyuk N A, Lavrov H V, Zarubin D N, Dolgushin F M, Ezernitskaya M G, Gloriozov I P, Zhokhov S S, Zhokhova N I, Ustynyuk Y A (2018). Russ Chem Bull.

[R37] 37Asano, T.; Kobayashi, T.; Shimojo, K.; Yamaoka, S.; Torii, R.; Okano, K.; Yaita, T.; Mori, A. Preparation of multiply fused phenanthroline derivatives employing catalytic C-H arylation and the highly selective extraction of lanthanides. Presented at the 41st Symposium on Solvent Extraction, Tokyo, November 25, 2022.

[38] Asano T, Nakanishi Y, Sugita S, Okano K, Narita H, Kobayashi T, Yaita T, Mori A (2024). ChemRxiv.

[39] Pramanik S, Li B, Driscoll D M, Johnson K R, Evans B R, Damron J T, Ivanov A S, Jiang D-e, Einkauf J, Popovs I (2024). J Am Chem Soc.

[40] Nakanishi Y, Sugita S, Okano K, Mori A (2024). Beilstein J Org Chem.

[41] Wang S, Lou J, Yang X, Xu X, Xu L, Fang D, Xu C, Xiao C (2025). Ind Eng Chem Res.

[42] Hamada Y, Takeuchi I, Hirota M (1974). Chem Pharm Bull.

[43] Chen S, Priebbenow D L, Somkhit J, Scullino C V, Agama K, Pommier Y, Flynn B L (2022). Chem – Eur J.

[44] Yedage S L, Bhanage B M (2016). J Org Chem.

[45] Yu Y, Yue Y, Wang D, Li X, Chen C, Peng J (2016). Synthesis.

[46] Saha R, Sekar G (2018). J Catal.

[47] Manikandan T S, Ramesh R, Semeril D (2019). Organometallics.

[48] Aleti R R, Festa A A, Voskressensky L G, Van der Eycken E V (2021). Molecules.

[49] Liu Z-S, Xie P-P, Hua Y, Wu C, Ma Y, Chen J, Cheng H-G, Hong X, Zhou Q (2021). Chem.

[50] Pan C, Wang L, Han J (2022). Adv Synth Catal.

[51] Babu Pathi V, Manna A, Srinath R, Adhikary S, Banerji B (2023). Eur J Org Chem.

[52] Li L-J, Zhou Z-Q, Liu Z-K, He Y-Y, Jia F-C, Hu X-Q (2023). Chem Commun.

[53] Jin L, Li Y, Mao Y, He X-B, Lu Z, Zhang Q, Shi B-F (2024). Nat Commun.

[54] Talukdar V, Paul S, Mondal K, Das P (2026). Adv Synth Catal.

