# Supplementary material for: Palladium-catalyzed benzocyclization reactions of quinoline-2-carboxamides via sequential C–H/N–H functionalization
Source: Beilstein J Org Chem. 2026 Jun 9;22:905–14. doi: 10.3762/bjoc.22.71 (PMC13267496; doi:10.3762/bjoc.22.71)
Supplement: File 1 — Experimental details and copies of 1H and 13C{1H} NMR spectra. [file Beilstein_J_Org_Chem-22-905-s001.pdf]

BEILSTEIN JOURNAL OF ORGANIC CHEMISTRY

Supporting Information

for

Palladium-catalyzed benzocyclization reactions of quinoline-2-carboxamides via sequential C–H/N–H functionalization

Shoichi Sugita, Kentaro Okano and Atsunori Mori

Beilstein J. Org. Chem. **2026,** *22,* 905–914. doi:10.3762/bjoc.22.71

Experimental details and copies of ^{1}H and $^{13}C\{^{1}H\}$ NMR spectra

License and Terms: This is a supporting information file under the terms of the Creative Commons Attribution License (https://creativecommons.org/licenses/by/4.0). Please note that the reuse, redistribution and reproduction in particular requires that the author(s) and source are credited and that individual graphics may be subject to special legal provisions.
The license is subject to the *Beilstein Journal of Organic Chemistry* terms and conditions: (https://www.beilstein-journals.org/bjoc/terms)

Table of contents

S1: General experimental details S2

S2: Preparation of substrates S3

S3: Method of C–H/N–H annulation reaction S15

S4: Additional data for the optimization of the C–H/N–H coupling reaction S24

S5: 2D NOESY spectra S26

S6: Confirmation of the structure of Int-1ad S31

S7: NMR spectra S32

References S112

S1: General experimental details

General

Analytical thin-layer chromatography (TLC) was performed on Merck 60 F254 aluminum sheets precoated with a 0.25 mm thickness of silica gel. Flash column chromatography was performed on Wakogel® C-300 (45–75 µm, Fujifilm Wako Pure Chemical Co.). Melting points (mp) were measured on a Yanaco MP-J3. ^{1}H (400 MHz) and ^{13}C{^{1}H} (100 MHz) NMR spectra were measured on a JEOL ECZ 400 NMR spectrometer at Research Center for Membrane and Film Technology, Kobe University. The chemical shifts were expressed in ppm with tetramethylsilane (0 ppm for ^{1}H NMR as an internal standard in $CDCl_3$) and $CDCl_3$ (77.16 ppm for ^{13}C NMR). IR spectra were recorded on Bruker Alpha with an ATR attachment (Ge) by single bounce ATR. HRMS spectra were measured with a JEOL JMS-T100L AccuTOF LC-Plus (ESI) with a JEOL MS-5414DART attachment.

Materials

Quinoline-2-carboxylic acid, 2-bromo-4-nitroaniline, 2-iodo-4-methylaniline, and *N*-iodosuccinimide (NIS) were purchased from BLD Pharmatech Ltd. *p*-Toluidine, tris(4-methoxyphenyl)phosphine ($P(4\text{-}MeOC_6H_4)_3$), 1,8-diazabicyclo[5,4,0]undec-2-ene (DBU), 2-bromo-4-*tert*-butylaniline, hydrobromic acid (HBr aq., 47%), *N*-bromosuccinimide (NBS), tri(*p*-tolyl)phosphine, and tri(*o*-tolyl)phosphine were purchased from Tokyo Chemical Industry Co., Ltd. Aniline, *o*-nitroaniline, acetic anhydride (Ac_2O), nitric acid (HNO_3 aq.), hydrochloric acid (HCl aq.), *o*-xylene, and tris(4-fluorophenly)phosphine were purchased from Fujifilm Wako Pure Chemical Co. *p*-Anisidine, *p*-chloroaniline, *o*-anisidine, acetic acid (AcOH), sulfuric acid (H_2SO_4), triethylamine (Et_3N), *N,N*-dimethylformamide (DMF), cesium carbonate (Cs_2CO_3), sodium nitrile ($NaNO_2$), potassium iodide (KI), acetonitrile (MeCN), *p*-toluenesulfonic acid monohydrate ($p\text{-}TsOH{\cdot}H_2O$), copper(I) bromide (CuBr), zinc powder, and sodium thiosulfate ($Na_2S_2O_3$) were purchased from NAKALAI TESQUE Inc. 4-*tert*-Butylaniline, 4-aminobenzonitrile, and 1-iodo-2-bromobenzene were purchased from Angene International Ltd. 2,4,6-Trimethylaniline, benzylamine, and palladium diacetate ($Pd(OAc)_2$) were purchased from Sigma-Aldrich Co. Oxalyl chloride was purchased from Tokyo Chemical industry Co., Ltd. and NAKALAI TESQUE Inc. Dichloromethane was purchased from Kanto Chemical Co., Inc.

S2: Preparation of substrates

S2-1: Synthesis of 2-quinolinecarboxamides

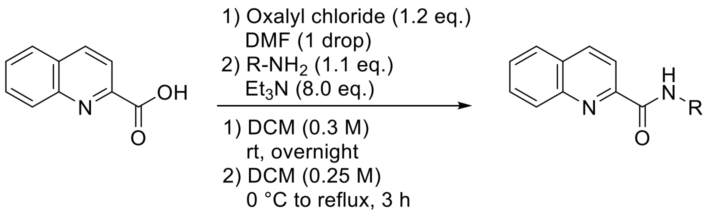

N-(4-Methylphenyl)-2-quinolinecarboxamide (1a)[1)]

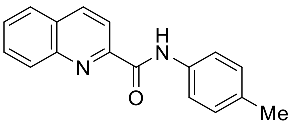

To a Schlenk tube equipped with a magnetic stirring bar was added quinoline-2-carboxylic acid (5 mmol, 1.0 equiv) and dissolved in dichloromethane (0.3 M). The mixture was cooled to 0 °C and stirred. To the mixture were added dropwise oxalyl chloride (6.0 mmol, 1.2 equiv) and DMF (1 drop, cat.) via syringe at 0 °C. The resulting mixture was warmed to room temperature and stirred for overnight. The solvent was removed under vacuum and the residue was suspended in dichloromethane (0.25 M). To the mixture was added Et_3N (40 mmol, 8.0 equiv) and the amine (5.5 mmol, 1.1 equiv) at 0 °C, then the mixture was stirred at the same temperature for 1 hour, followed by heating to reflux temperature and further stirring for 2 hours. The reaction was quenched with 1 M HCl aq. and extracted thrice with dichloromethane. The organic phases were washed with sat. $NaHCO_3$ aq. and brine, then dried over Na_2SO_4, filtered, and evaporated under reduced pressure to give a crude product. The product was separated with silica gel column chromatography (hexane/AcOEt 2:1) to give the corresponding amide in 89% yield as a light brown solid. ^{1}H NMR ($CDCl_3$): 10.18 (s, 1H), 8.40 (dd, J = 8.8, 8.0 Hz, 1H), 8.38 (dd, J = 8.8, 8.8 Hz, 1H), 8.19 (d, J = 8.8 Hz, 1H), 7.92 (d, J = 7.6 Hz, 1H), 7.81 (dd, J = 8.0, 7.2 Hz, 1H), 7.70-7.79 (m, 2H), 7.66 (ddd, J = 7.6, 7.2, 0.8 Hz, 1H), 7.17-7.25 (m, 2H), 2.37 (s, 3H); ^{13}C{^{1}H} NMR ($CDCl_3$):162.1, 149.9, 146.4, 137.9, 135.4, 134.1, 130.4, 129.8, 129.7, 129.5, 128.2, 127.9, 119.9, 118.9, 21.1.

The syntheses of **1b** to **1f** and **1h–k** was carried out in a similar manner, spectroscopic characteristics and analytical properties are shown below.

N-Phenyl-2-quinolinecarboxamide (1b)[1)]

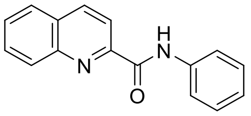

Yield: 76% as a brown solid; ^{1}H NMR ($CDCl_3$): 10.25 (s, 1H), 8.40 (dd, *J* = 8.8, 8.4 Hz, 1H), 8.38 (dd, *J* = 8.8, 8.4 Hz, 1H), 8.20 (d, *J* = 8.8 Hz, 1H), 7.92 (d, *J* = 8.0 Hz, 1H), 7.84-7.89 (m, 2H), 7.82 (ddd, *J* = 8.0, 7.2, 1.6 Hz, 1H), 7.66 (dd, *J* = 8.0, 7.2 Hz, 1H), 7.38-7.47 (m, 2H), 7.15-7.21 (m, 1H); $^{13}C\{^1H\}$ NMR ($CDCl_3$): 162.3, 149.8, 146.4, 138.0, 137.9, 130.5, 129.8, 129.5, 129.2, 128.3, 127.9, 124.5, 119.9, 118.9.

N-(4-Methoxyphenyl)-2-quinolinecarboxamide (1c)[2)]

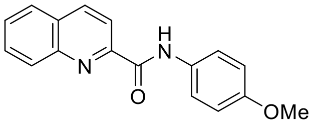

Yield: 82% as a white solid; ^{1}H NMR ($CDCl_3$): 10.13 (s, 1H), 8.32-8.42 (m 2H), 8.17 (d, *J* = 8.4 Hz, 1H), 7.90 (d, *J* = 8.4 Hz, 1H), 7.80 (ddd, *J* = 8.4, 6.8, 1.2 Hz, 1H), 7.74-7.79 (m, 2H), 7.64 (ddd, *J* = 8.4, 6.8, 0.8 Hz, 1H), 6.92-6.99 (m, 2H), 3.83 (s, 3H); $^{13}C\{^1H\}$ NMR ($CDCl_3$): 162.0, 156.5, 149.9, 146.4, 137.9, 131.2, 130.4, 129.8, 129.5, 128.2, 127.9, 121.4, 118.8, 114.4, 55.6.

N-[4-(*tert*-Butyl)phenyl]-2-quinolinecarboxamide (1d)

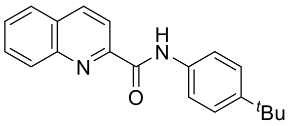

Yield: 45% as a brown solid; Mp: 99.4–101.4 ºC; ^{1}H NMR ($CDCl_3$): 10.19 (s, 1H), 8.35-8.44 (m, 2H), 8.20 (d, *J* = 8.4 Hz, 1H), 7.92 (d, *J* = 8.0 Hz, 1H), 7.81 (ddd, *J* = 8.4, 7.6, 0.8 Hz, 1H), 7.74-7.80 (m, 2H), 7.66 (dd, *J* = 8.0, 6.8 Hz, 1H), 7.40-7.48 (m, 2H), 1.35 (s, 9H); $^{13}C\{^1H\}$ NMR ($CDCl_3$): 162.2, 149.9, 147.4, 146.4, 137.9, 135.3, 130.4, 129.8, 127.9, 126.0, 119.7, 118.9, 34.6, 31.5; IR (ATR): 3353, 3343, 2961, 2901, 2870, 1684, 1589, 1529, 1502, 1408, 1320, 1269, 1133, 1114, 1097; HRMS (DART$^+$) *m/z* $[M+H]^+$ calcd. for $C_{20}H_{21}N_2O$ 305.1649; found, 305.1662.

N-(4-Cyanophenyl)-2-quinolinecarboxamide (1e)[2)]

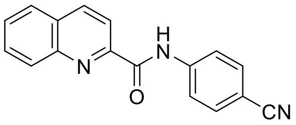

Yield: 63% as a pale yellow solid; ^{1}H NMR ($CDCl_3$): 10.47 (s, 1H), 8.37-8.44 (m, 2H), 8.20 (d, *J* = 8.8 Hz, 1H), 7.97-8.03 (m, 2H), 7.94 (d, *J* = 8.0 Hz, 1H), 7.84 (dd, *J* = 8.4, 7.2 Hz, 1H), 7.70-7.74 (m,

2H), 7.69 (ddd, J = 8.4, 7.2, 1.2 Hz, 1H); $^{13}C\{^{1}H\}$ NMR ($CDCl_3$): 162.6, 148.8, 146.3, 141.8, 138.3, 133.5, 130.8, 129.7, 128.7, 128.0, 119.8, 119.1, 118.7, 114.5, 107.3.

***N*-(4-Chlorophenyl)-2-quinolinecarboxamide (1f)**[1)]

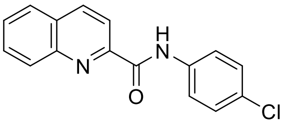

Yield: 81% as a white solid; ^{1}H NMR ($CDCl_3$): 10.25 (s, 1H), 8.35-8.41 (m, 2H), 8.18 (d, J = 8.8 Hz, 1H), 7.92 (dd, J = 8.4, 1.2 Hz, 1H), 7.78-7.85 (m, 3H), 7.66 (ddd, J = 8.4, 7.2, 1.2 Hz, 1H), 7.34-7.71 (m, 2H); $^{13}C\{^{1}H\}$ NMR ($CDCl_3$): 162.3, 149.4, 146.4, 138.1, 136.5, 130.6, 129.7, 129.6, 129.4, 129.2, 128.4, 128.0, 121.1, 118.8.

***N*-Mesityl-2-quinolinecarboxamide (1h)**

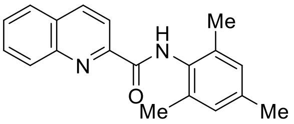

Yield: 65% as a brown solid; Mp: 142.8–144.5 ºC; ^{1}H NMR ($CDCl_3$): 9.64 (s, 1H), 8.34-8.44 (m, 2H), 8.17 (d, J = 8.8 Hz, 1H), 7.92 (d, J = 8.4 Hz, 1H), 7.80 (ddd, J = 8.4, 7.2, 0.8 Hz, 1H), 7.66 (dd, J = 8.0, 7.2 Hz, 1H), 6.97 (s, 2H), 2.32 (s, 3H), 2.29 (s, 6H); $^{13}C\{^{1}H\}$ NMR ($CDCl_3$): 162.9, 149.9, 146.6, 137.7, 136.9, 135.3, 131.3, 130.3, 129.9, 129.5, 129.1, 128.1, 127.9, 119.2, 21.1, 18.7; IR (ATR): 3358, 2920, 1681, 1516, 1496, 1426, 1161; HRMS (DART^{+}) *m/z* $[M+H]^{+}$ calcd. for $C_{19}H_{19}N_2O$ 291.1492; found, 291.1503.

***N*-(2-Nitrophenyl)-2-quinolinecarboxamide (1i)**

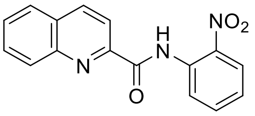

Yield: 71% as a yellow solid; Mp: 175.1–175.6 ºC; ^{1}H NMR ($CDCl_3$): 13.09 (s, 1H), 9.09 (dd, J = 8.4, 1.2 Hz, 1H), 8.32-8.39 (m, 2H), 8.30 (dd, J = 8.0, 1.2 Hz, 1H), 8.29 (d, J = 8.0 Hz, 1H), 7.90 (d, J = 8.0 Hz, 1H), 7.82 (ddd, J = 8.4, 7.2, 1.2 Hz, 1H), 7.72 (ddd, J = 8.4, 7.2, 1.2 Hz, 1H), 7.66 (ddd, J = 8.4, 7.2, 1.2 Hz, 1H), 7.23 (ddd, J = 8.4, 7.2, 1.2 Hz); $^{13}C\{^{1}H\}$ NMR ($CDCl_3$): 163.7, 149.0, 146.5, 138.0, 137.0, 135.9, 134.8, 130.6, 130.4, 129.7, 128.7, 127.7, 126.1, 123.4, 121.9, 118.7; IR (ATR): 3261, 1698, 1606, 1581, 1503, 1449, 1439, 1424, 1344, 1314, 1273, 1147, 1124; HRMS (DART^{+}) *m/z* $[M+H]^{+}$ calcd. for $C_{16}H_{12}N_3O_3$ 294.0874; found, 294.0882.

***N*-(2-Methoxyphenyl)-2-quinolinecarboxamide (1j)**[1)]

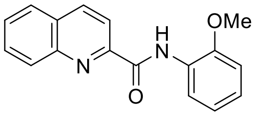

Yield: 86% as a white solid; ^{1}H NMR ($CDCl_3$): 10.82 (s, 1H), 8.67 (dd, *J* = 8.0, 2.0 Hz, 1H), 8.32-8.43 (m, 2H), 8.21 (d, *J* = 8.0 Hz, 1H), 7.89 (d, *J* = 8.0 Hz, 1H), 7.79 (ddd, *J* = 8.0, 6.8, 1.6 Hz, 1H), 7.63 (ddd, *J* = 8.0, 6.8, 1.2 Hz, 1H), 7.12 (ddd, *J* = 8.0, 7.8, 2.0 Hz, 1H), 7.06 (ddd, *J* = 8.0, 7.8, 1.6 Hz), 4.02 (s, 3H); ^{13}C{^{1}H} NMR ($CDCl_3$): 162.3, 150.2, 149.0, 146.5, 137.8, 130.2, 130.1, 129.4, 128.1, 127.9, 127.7, 124.1, 121.2, 119.9, 118.9, 110.2, 56.0.

***N*-Benzyl-2-quinolinecarboxamide (1k)**[3)]

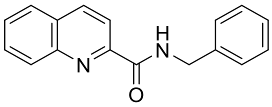

Yield: 82% as a white solid; ^{1}H NMR ($CDCl_3$): 8.61 (br s, 1H), 8.30-8.38 (m, 2H), 8.07 (d, *J* = 8.4 Hz, 1H), 7.89 (d, *J* = 8.0 Hz, 1H), 7.75 (ddd, *J* = 8.0, 6.8, 1.2 Hz, 1H), 7.62 (ddd, *J* = 8.0, 7.2, 1.2 Hz, 1H), 7.40-7.47 (m, 2H), 7.34-7.41 (m, 2H), 7.27-7.34 (m, 1H), 4.75 (d, *J* = 6.0 Hz, 2H); ^{13}C{^{1}H} NMR ($CDCl_3$): 164.5, 149.7, 146.6, 138.4, 137.6, 130.2, 129.8, 129.4, 128.8, 128.0, 127.8, 127.6, 119.0, 43.7.

S2-2: Synthesis of *N*-(4-nitrophenyl)-2-quinolinecarboxamide (1g)

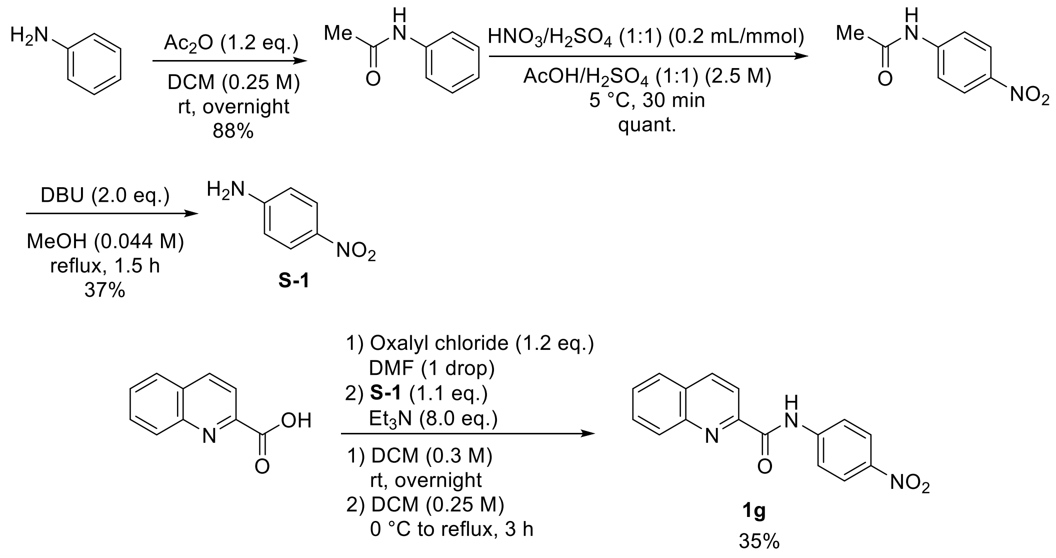

Acetanilide was synthesized according to the literature procedure[4)]. To a round-bottomed flask equipped with a magnetic stirring bar were added aniline (10 mmol) and dichloromethane (0.25 M). Ac_2O (12 mmol, 1.2 equiv) was then added dropwise to the stirring mixture. The resulting mixture was stirred overnight at room temperature and the reaction was monitored by TLC. The solution was replaced to separation funnel and washed with 1 M HCl aq. and sat. $NaHCO_3$ aq. The organic phase was dried over Na_2SO_4, filtered, and the solvent was removed under reduced pressure to give 88% of the corresponding acetanilide which was used in the next reaction step without further purification.

The nitration product was synthesized according to the literature procedure[5)]. To a round-bottomed flask equipped with a magnetic stirring bar were added acetanilide (8.8 mmol) and a 1:1 mixture of AcOH and H_2SO_4 solution (2.5 M). The mixture was heated until all solids completely dissolved. The solution was cooled to 5 °C, then to the mixture was added a 1:1 mixture of HNO_3 and H_2SO_4 (1.76 mL). The resulting mixture was stirred for 30 min at 5 °C, then poured into ice–water. The precipitate was filtered and dried under vacuum to give the corresponding 4-nitroacetanilide in quantitative yield which was used in the next reaction step without further purification.

The deprotected product was synthesized according to the literature procedure[6)]. To a round-bottomed flask equipped with a magnetic stirring bar were added 4-nitroacetanilide (8.8 mmol), MeOH (200 mL), and DBU (17.6 mmol, 2.0 equiv). The mixture was stirred for 1.5 h at reflux, then poured into H_2O to induce precipitation. The precipitate was filtered and dried to give the corresponding 4-nitroaniline in 37% yield, which was used in the amidation reaction without further purification.

The amide was synthesized according to the general procedure. To a Schlenk tube equipped with a magnetic stirring bar was added quinoline-2-carboxylic acid (2.9 mmol, 1.0 equiv), dissolved in dichloromethane (0.3 M) and cooled to 0 °C. To the stirred solution were added dropwise oxalyl

chloride (3.5 mmol, 1.2 equiv) and DMF (1 drop, cat.) via syringe at 0 °C. The resulting mixture was warmed to room temperature and stirred for overnight. The solvent was removed under vacuum and the residue was suspended in dichloromethane (0.25 M). To the mixture were added Et_3N (23.2 mmol, 8.0 equiv) and 4-nitroaniline (3.2 mmol, 1.1 equiv) at 0 °C, then the mixture was stirred at the same temperature for 1 hour, followed by heating to refluxing temperature and further stirring for 2 hours. The reaction was quenched with 1 M HCl aq. and extracted thrice with dichloromethane. The organic phases were washed with sat. $NaHCO_3$ aq. and brine, then dried over Na_2SO_4, filtered, and evaporated under reduced pressure to give a crude product. The product was separated with silica gel column chromatography (hexane/AcOEt 2:1) to give the corresponding amide in 35% yield as a yellow solid.

_N_-(4-Nitrophenyl)-2-quinolinecarboxamide (1g)[1)]

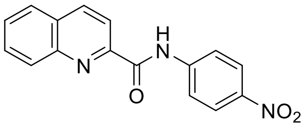

^{1}H NMR ($CDCl_3$): 10.58 (s, 1H), 8.36-8.43 (m, 2H), 8.27-8.34 (m, 2H), 8.20 (d, *J* = 8.8 Hz, 1H), 8.00-8.07 (m, 2H), 7.94 (d, *J* = 8.0 Hz, 1H), 7.84 (ddd, *J* = 8.0, 6.8, 1.2 Hz, 1H), 7.69 (ddd, *J* = 8.0, 6.8, 0.8 Hz, 1H); ^{13}C{^{1}H} NMR ($CDCl_3$): 162.7, 148.7, 146.4, 143.73, 143.66, 138.4, 130.9, 129.82, 129.77, 128.8, 128.1, 125.4, 119.4, 118.8.

S2-3: Synthesis of 1-bromo-2-iodobenzene derivatives

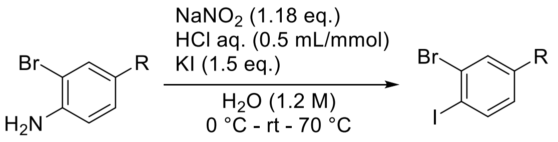

The iodination was carried out according to the literature procedure. [7] To a stirring solution of 2-bromoaniline derivatives (10 mmol, 1.0 equiv) in H_2O (17 mL) and HCl aq. (5 mL) at 0 °C was added $NaNO_2$ (1.18 equiv, 11.8 mmol) in H_2O (17 mL) and the resulting mixture was stirred for 10 minutes at the same temperature. To the diazonium solution was then added dropwise KI (1.50 equiv, 15 mmol) in H_2O (3.8 mL) at 0 °C. The mixture was warmed to room temperature and stirred for 30 minutes, followed by heating at 70 °C for 1 h. The resulting mixture was quenched with sat. $Na_2S_2O_3$ aq. and extracted thrice with dichloromethane. The combined organic layers were dried over Na_2SO_4, filtered, and evaporated under reduced pressure. The crude product was purified with silica gel column chromatography (hexane) to give the corresponding 1-bromo-2-iodobenzene.

1-Bromo-2-iodo-5-*tert*-butylbenzene (2d)[8]

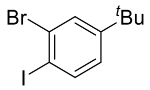

Yield: 81% as a colorless oil; ^{1}H NMR ($CDCl_3$): 7.75 (d, J = 8.4 Hz, 1H), 7.63 (d, J = 2.0 Hz, 1H), 7.02 (dd, J = 8.4, 2.0 Hz, 1H), 1.29 (s, 9H); $^{13}C\{^1H\}$ NMR ($CDCl_3$): 153.5, 139.9, 130.2, 129.6, 126.1, 97.2, 34.8, 31.1.

1-Bromo-2-iodo-5-nitrobenzene (2i)[9]

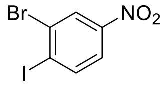

Yield: 62% as a yellow solid; ^{1}H NMR ($CDCl_3$): 8.44 (d, J = 3.0 Hz, 1H), 8.07 (d, J = 8.8 Hz, 1H), 7.84 (dd, J = 8.8, 3.0 Hz, 1H); $^{13}C\{^1H\}$ NMR ($CDCl_3$); 148.3, 141.1, 131.0, 127.4, 122.8, 110.4.

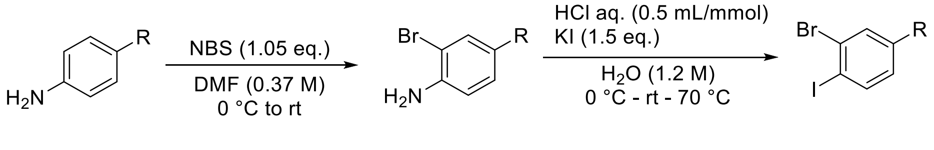

The bromination was carried out according to the literature procedure.[10] To a solution of 4-substituted

aniline (10 mmol, 1.0 equiv) in DMF (0.37 M) was added portionwise NBS (10.5 mmol, 1.05 equiv) at 0 °C. The reaction mixture was stirred for 30 min at 0 °C, then warmed to room temperature and stirred for 18 hours at the same temperature. The reaction mixture was diluted with CH_2Cl_2 and washed with brine. The organic phase was dried over Na_2SO_4, filtered, and evaporated under reduced pressure. The crude product was purified with silica gel column chromatography (hexane/AcOEt 20:1) to give the corresponding 2-bromo-4-substituted aniline.[11,12)]

The iodination was carried out according to the literature procedure. [7)] To a stirring solution of 2-bromoaniline derivatives (10 mmol, 1 equiv) in H_2O (17 mL) and HCl aq. (5 mL) at 0 °C was added $NaNO_2$ (1.18 equiv, 11.8 mmol) in H_2O (17 mL) and the resulting mixture was stirred for 10 minutes at the same temperature. To the diazonium solution was then added dropwise KI (1.50 equiv, 15 mmol) in H_2O (3.8 mL) at 0 °C. The mixture was warmed to room temperature and stirred for 30 minutes, followed by heating at 70 °C for 1 h. The resulting mixture was quenched with sat. $Na_2S_2O_3$ aq. and extracted thrice with dichloromethane. The combined organic layers were dried over Na_2SO_4, filtered, and evaporated under reduced pressure. The crude product was purified with silica gel column chromatography (hexane) to give the corresponding 1-bromo-2-iodobenzene.

3-Bromo-4-iodotoluene (2b)[13)]

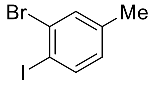

Yield: 65% as a colorless oil; ^{1}H NMR ($CDCl_3$): 7.70 (d, J = 8.0 Hz, 1H), 7.46 (d, J = 2.0 Hz, 1H), 7.00 (dd, J = 8.0, 2.0 Hz, 1H), 2.28 (s, 3H); $^{13}C\{^1H\}$ NMR ($CDCl_3$): 140.02, 139.98, 133.5, 129.7, 129.5, 97.0, 20.8; IR (ATR): 2921, 1458, 1370, 1259, 1208, 1097, 1006; HRMS (DART$^+$) *m/z* $[M+H]^+$ calcd. for $C_7H_6{}^{79}BrI$ 295.8698; found, 295.8689.

3-Bromo-4-iodoanisole (2f)[14)]

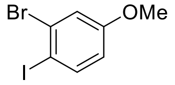

Yield: 54% as a colorless oil; ^{1}H NMR ($CDCl_3$): 7.67 (d, J = 8.8 Hz, 1H), 7.18 (d, J = 3.2 Hz, 1H), 6.59 (dd, J = 8.8, 3.2 Hz, 1H), 3.77 (s, 3H); $^{13}C\{^1H\}$ NMR ($CDCl_3$): 160.3, 140.3, 130.0, 118.5, 115.4, 89.6, 55.7.

Synthesis of 3-bromo-4-iodobenzonitrile (2h)

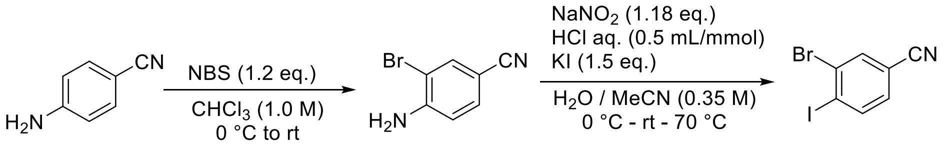

To a stirred solution of 4-aminobenzonitrile (7.5 mmol, 1.0 equiv) in chloroform (7.5 mL, 1.0 M) was added portionwise NBS (9.0 mmol, 1.2 equiv) during 1 hour at 0 °C. The reaction mixture was warmed to room temperature and stirred for 4 h, then the resulting precipitate was filtered off. The filtrate was washed with H_2O, dried over Na_2SO_4, filtered, and evaporated under reduced pressure. The crude product was purified with silica gel column chromatography (hexane/AcOEt 2:1) to give 3-bromo-4-aminobenzonitrile in 58% yield.

The iodination was carried out according to the modified literature procedure. [7)] To a stirring solution of 3-bromo-4-aminobenzonitrile (4.3 mmol, 1.0 equiv) in H_2O/acetonitrile 3:2 (0.35 M) and HCl aq. (2.25 mL) at 0 °C was added $NaNO_2$ (5.1 mmol, 1.18 equiv) in H_2O (1.9 mL) and the resulting mixture was stirred for 10 minutes at the same temperature. To the diazonium solution was then added dropwise KI (6.5 mmol, 1.50 equiv) in H_2O (1.9 mL) at 0 °C. The mixture was warmed to room temperature and stirred for 30 minutes, followed by heating at 70 °C for 1 h. The resulting mixture was quenched with sat. $Na_2S_2O_3$ aq. and extracted thrice with dichloromethane. The combined organic layers were dried over Na_2SO_4, filtered, and evaporated under reduced pressure. The crude product was purified with silica gel column chromatography (hexane) to give 3-bromo-4-iodobenzonitrile.

3-Bromo-4-iodobenzonitrile (2h)[15)]

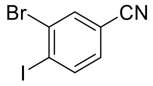

Yield: 59% as a colorless solid; ^{1}H NMR ($CDCl_3$): 7.99 (dd, J = 8.4, 0.8 Hz, 1H), 7.87 (m, 1H), 7.26 (dd, J = 8.4, 1.6 Hz, 1H); ^{13}C{^{1}H} NMR ($CDCl_3$): 141.2, 135.3, 131.1, 131.0, 117.0, 113.6, 108.3.

Synthesis of 4-substituted 1-bromo-2-iodobenzene derivatives

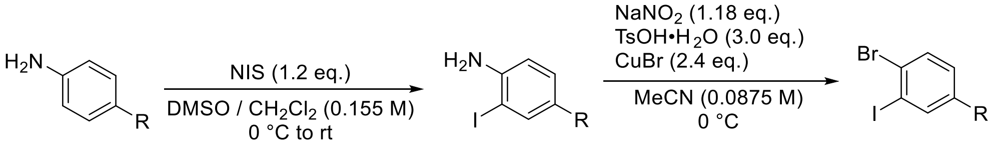

The iodination was carried out according to the literature procedure.[16)] To a stirring solution of 4-substituted aniline (10 mmol, 1.0 equiv) in DMSO/dichloromethane 2:1 (0.155 M) was added *N*-iodosuccinimide (12 mmol, 1.2 equiv) at 0 °C. The mixture was stirred at the same temperature for 10 minutes. The resulting mixture was quenched with sat. $Na_2S_2O_3$ aq. The biphasic layer was extracted thrice with dichloromethane and the combined organic layers were washed with brine. The organic phase was dried over Na_2SO_4, filtered, and evaporated. The crude product was purified with silica gel column chromatography (hexane/AcOEt 10:1) to give the corresponding 2-iodo-4-substituted aniline.

The bromination was carried out according to the literature procedure.[17)] To a stirring solution of 2-

iodo-4-substituted aniline (10.0 mmol, 1.0 equiv) in MeCN (0.0875 M) was added *p*-TsOH·H_2O (30.0 mmol, 3.0 equiv) at 0 °C. To the mixture was added dropwise a solution of $NaNO_2$ (19.4 mmol, 1.94 equiv) in H_2O (7.8 mL) at the same temperature. The mixture was stirred for 2 hours at 0 °C. To the resulting diazonium solution was added CuBr (24.0 mmol, 2.4 equiv) at 0 °C. The reaction mixture was stirred for additional 1 hour at the same temperature. The reaction mixture was diluted with H_2O and the biphasic layer extracted thrice with AcOEt. The combined organic layers were washed with brine, dried over Na_2SO_4, filtered, and evaporated. The crude product was purified with silica gel column chromatography (hexane) to give the corresponding 1-bromo-2-iodo-4-substituted benzene.

4-Bromo-3-iodotoluene (2c)

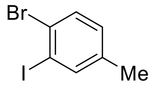

Yield: 65% as a colorless oil; ^1H NMR ($CDCl_3$): 7.69 (d, *J* = 1.6 Hz, 1H), 7.47 (d, *J* = 8.4 Hz, 1H), 7.00 (dd, *J* = 8.4, 1.6 Hz, 1H), 2.26 (s, 3H); $^{13}C\{^1H\}$ NMR ($CDCl_3$): 140.9, 138.8, 132.3, 130.5, 126.3, 101.1, 20.5; IR (ATR): 2920, 1456, 1369, 1257, 1100, 1010; HRMS (DART$^+$) *m/z* $[M+H]^+$ calcd. for $C_7H_6{}^{79}BrI$ 295.8698; found, 295.8707.

1-Bromo-2-iodo-4-*tert*-butylbenzene (2e)[18)]

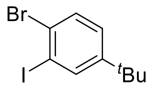

Yield: 79% as a colorless oil; ^1H NMR ($CDCl_3$): 7.84 (d, *J* = 2.0 Hz, 1H), 7.52 (d, *J* = 8.0 Hz, 1H), 7.00 (dd, *J* = 8.0, 2.0 Hz, 1H), 1.28 (s, 9H); $^{13}C\{^1H\}$ NMR ($CDCl_3$): 152.2, 137.6, 132.3, 127.1, 126.5, 101.3, 34.6, 31.2.

Synthesis of 4-bromo-3-iodoanisole

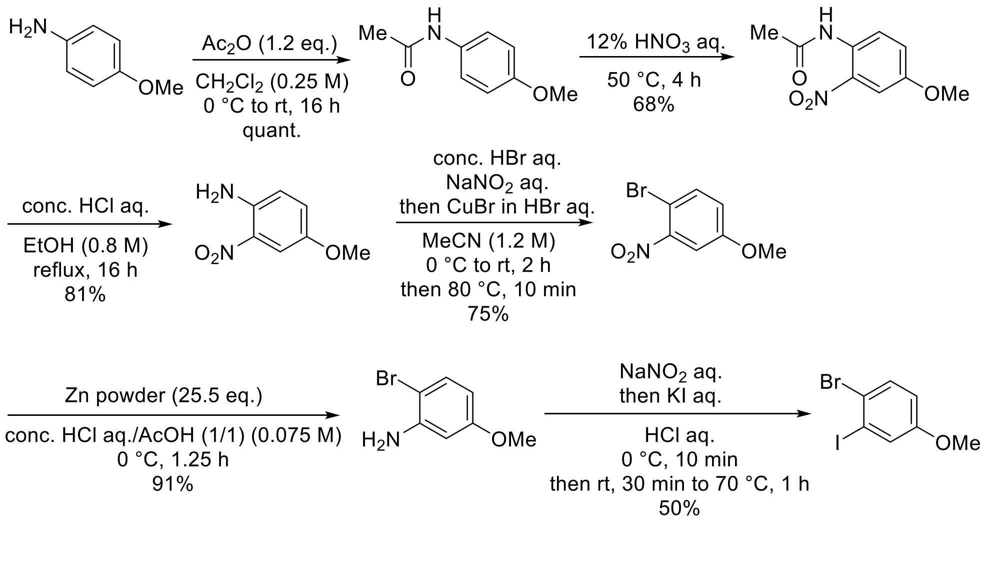

To a solution of *p*-anisidine (10 mmol, 1.0 equiv) in CH_2Cl_2 (0.25 M) at 0 °C was added acetic anhydride (12 mmol, 1.2 equiv), then the reaction mixture was warmed to room temperature and stirred for 16 h. The mixture was washed with sat. $NaHCO_3$ aq., 1.0 M HCl aq., H_2O, and brine. The organic phase was dried over Na_2SO_4, filtered, and evaporated under reduced pressure to give *p*-acetanisidide as a colorless solid.[19] The product was used in the next reaction without further purification.

To a solution of *p*-acetanisidide (10 mmol, 1.0 equiv) in 12% of HNO_3 aq. was stirred at 50 °C for 4 h, then the reaction mixture was cooled to room temperature. After the reaction, cold H_2O was added to the reaction mixture. The resulting precipitate was filtered, washed with H_2O, then dried under vacuum to give *N*-(4-methoxy-2-nitrophenyl)acetamide in 68% yield (over 2 steps) as a yellow solid.[20] The product was used in the next reaction without further purification.

To a solution of *N*-(4-methoxy-2-nitrophenyl)acetamide (6.8 mmol, 1 equiv) in EtOH (0.8 M) was added dropwise conc. HCl aq. (4.2 mL), and the reaction mixture was stirred for 16 h at reflux. The reaction mixture was cooled to room temperature and quenched with 28% NH_3 aq. (3.4 mL) under ice bath cooling. The resulting precipitate was filtered, washed with H_2O, and dried under vacuum to give 4-methoxy-2-nitroaniline in 81% yield as a red solid.[21] The product was used in the next reaction without further purification.

The bromination was carried out according to the literature procedure.[22] To a suspension of 4-methoxy-2-nitroaniline (5.5 mmol, 1 equiv) in MeCN (1.2 M) was added HBr (47%, 5.5 mL) and the mixture was cooled to 0 °C. A solution of $NaNO_2$ (1.0 equiv) in H_2O (2.3 mL) was added dropwise to the reaction mixture at the same temperature, then the mixture was warmed to room temperature and stirred for 2 hours. To the mixture was added a solution of CuBr (0.9 equiv) in HBr (47%, 2.3 mL) and the reaction mixture was heated to 80 °C for 10 minutes. The mixture was cooled to room temperature and solid NaOH was added until the red color changed to blue-green. The mixture was extracted thrice with AcOEt and the organic layers were washed with brine, dried over Na_2SO_4, and concentrated at reduced pressure. The residue was purified with silica gel column chromatography (hexane/AcOEt 5:1) to give 1-bromo-4-methoxy-2-nitrobenzene in 75% yield as a yellow solid.[22]

The reduction was carried out according to the literature procedure.[23] To a mixture of concentrated HCl aq./AcOH 1:1 (0.075 M) at 0 °C was added 1-bromo-4-methoxy-2-nitrobenzene (4.1 mmol 1.0 equiv). Zinc powder (6.9 g, 25.5 equiv) was then added portionwise to the mixture over 1 hour after which point the reaction mixture was stirred for an additional 15 minutes at 0 °C. Then the reaction was quenched by the addition of ammonium hydroxide until slightly basic. The crude product was extracted thrice with DCM and washed with brine. The organic layers were dried over Na_2SO_4 and concentrated under reduced pressure. The residue was purified with silica gel column chromatography (hexane/AcOEt 5:1) to give 2-bromo-5-methoxyaniline in 91% yield as a brown oil.

The iodination was carried out according to the literature procedure.[7] To a stirring solution of 2-bromo-5-methoxyaniline (3.8 mmol, 1.0 equiv) in H_2O and HCl aq. at 0 °C was added $NaNO_2$ in H_2O

and the resulting mixture was stirred for 10 minutes at the same temperature. To the diazonium solution was then added dropwise KI in H_2O at 0 °C. The mixture was warmed to room temperature and stirred for 30 minutes, followed by heating at 70 °C for 1 h. The resulting mixture was quenched with sat. $Na_2S_2O_3$ aq. and extracted thrice with dichloromethane. The combined organic layers were dried over Na_2SO_4, filtered, and evaporated under reduced pressure. The crude product was purified with silica gel column chromatography (hexane) to give 4-bromo-3-iodoanisole in 50% yield as a colorless oil.

4-Bromo-3-iodoanisole (2g)[24)]

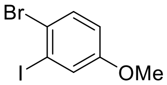

^{1}H NMR ($CDCl_3$): 7.46 (d, J = 9.2 Hz, 1H), 7.38 (d, J = 2.8 Hz, 1H), 6.76 (dd, J = 8.0, 2.8 Hz, 1H), 3.76 (s, 3H); ^{13}C{^{1}H} NMR ($CDCl_3$): 158.7, 132.7, 125.4, 120.3, 116.1, 101.2, 55.8.

S3: Method of C–H/N–H annulation reaction

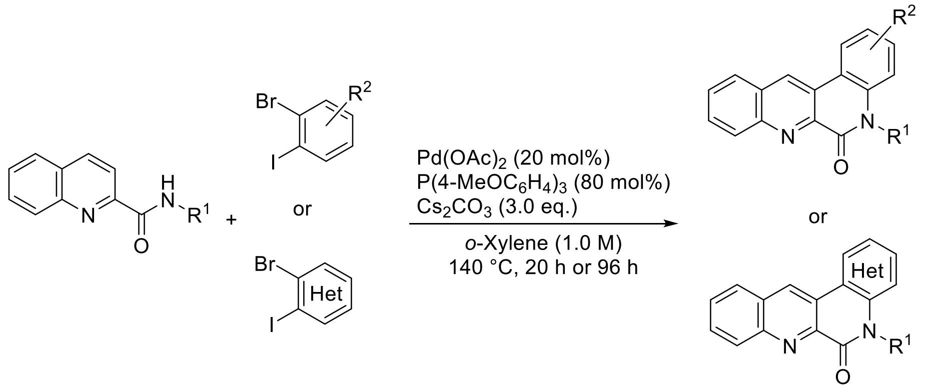

To a screw-capped test tube equipped with a magnetic stirring bar were added quinoline-2-carboxamide (0.05 mmol, 1.0 equiv), $Pd(OAc)_2$ (0.01 mmol, 20 mol %), $P(4\text{-}MeOC_6H_4)_3$ (0.04 mmol, 80 mol %), Cs_2CO_3 (0.15 mmol, 3.0 equiv), and 1-iodo-2-bromobenzene derivatives (0.1 mmol, 2.0 equiv), followed by dissolution in *o*-xylene (1.0 M). The test tube was backfilled with N_2 atmosphere and sealed with a screw cap. The mixture was stirred at 140 °C for 20 hours (Method A) or 96 hours (Method B). The reaction mixture was quenched with H_2O and extracted thrice with dichloromethane. The combined organic layers were dried over Na_2SO_4, filtered, and evaporated under reduced pressure. The crude product was purified with silica gel column chromatography (hexane to hexane/AcOEt 1:9 gradient) to give the corresponding lactam.

5-(4-Methylphenyl)dibenzo[*b*,*f*][1,7]naphthyridin-6(5*H*)-one (3aa)

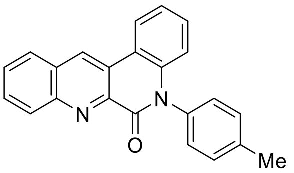

Method A; Yield: 81% as a brown solid; Mp: over 250 ºC (decomp.); ^{1}H NMR ($CDCl_3$): 9.15 (s, 1H), 8.47 (d, *J* = 8.8 Hz, 1H), 8.36-8.44 (m, 1H), 8.06 (d, *J* = 8.4 Hz, 1H), 7.83 (ddd, *J* = 8.8, 6.8, 1.2 Hz, 1H), 7.71 (ddd, *J* = 8.4, 6.8, 1.2 Hz, 1H), 7.43 (d, *J* = 8.4 Hz, 2H), 7.30-7.38 (m, 2H), 7.25 (d, *J* = 8.4 Hz, 2H), 6.71-6.79 (m, 1H), 3.15 (s, 3H); ^{13}C{^{1}H} NMR ($CDCl_3$): 160.3, 148.4, 142.4, 139.0, 138.8, 135.3, 131.2, 131.0, 130.5, 130.2, 129.6, 129.3, 128.8, 128.6, 127.7, 126.7, 123.2, 123.0, 117.9, 117.4, 21.3; IR (ATR): 2980, 2964, 2936, 2925, 1673, 1605, 1511, 1493, 1351, 1331, 1315; HRMS (DART$^+$) *m/z* $[M+H]^+$ calcd. for $C_{23}H_{17}N_2O$ 337.1341; found, 337.1349.

5-Phenyldibenzo[*b*,*f*][1,7]naphthyridin-6(5*H*)-one (3ba)

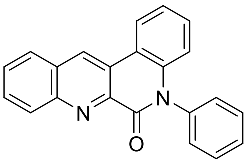

Method A; Yield: 81% as a brown solid; Mp: over 250 ºC (decomp.); ^{1}H NMR ($CDCl_3$): 9.16 (s, 1H), 8.48 (d, J = 8.8 Hz, 1H), 8.38-8.45 (m, 1H), 8.08 (dd, J = 8.0, 0.8 Hz, 1H), 7.85 (ddd, J = 8.8, 6.8, 1.6 Hz, 1H), 7.72 (ddd, J = 8.0, 6.8, 1.2 Hz, 1H), 7.61-7.69 (m, 2H). 7.56) 7.53-7.60 (m, 1H), 7.36-7.41 (m. 2H), 7.32-7.37 (m, 2H), 6.68-6.24 (m, 1H); ^{13}C{^{1}H} NMR ($CDCl_3$): 160.3, 148.6, 142.4, 138.9, 138.1, 131.2, 130.7, 130.4, 129.8, 129.4, 129.1, 129.04, 128.99, 127.8, 126.9, 123.4, 123.2, 118.0, 117.4; IR (ATR): 3059, 2980, 2961, 2921, 2849, 1672, 1604, 1493, 1465, 1412, 1384, 1352, 1331, 1315, 1283, 1225, 1149, 1125; HRMS (DART$^+$) *m/z* [M+H]$^+$ calcd. for $C_{22}H_{15}N_2O$ 323.1184; found, 323.1186.

5-(4-Methoxyphenyl)dibenzo[*b*,*f*][1,7]naphthyridin-6(5*H*)-one (3ca)

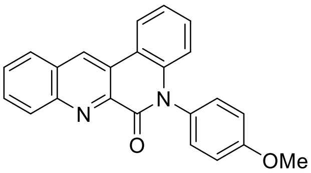

Method A; Yield: 68% as a brown solid; Mp: over 250 ºC (decomp.); ^{1}H NMR ($CDCl_3$): 9.16 (s, 1H), 8.48 (d, J = 8.8 Hz, 1H), 8.38-8.44 (m, 1H), 8.07 (d, J = 7.6 Hz, 1H), 7.84 (ddd, J = 8.4, 6.8, 1.2 Hz, 1H), 7.72 (ddd, J = 8.4, 6.8, 1.2 Hz, 1H), 7.31-7.39 (m, 1H), 7.26-7.31 (m, 2H), 7.11-7.18 (m, 2H), 6.74-6.81 (m, 1H), 3.92 (s, 3H); ^{13}C{^{1}H} NMR ($CDCl_3$): 160.6, 159.8, 148.5, 142.5, 139.3, 131.2, 130.6, 130.3, 130.0, 129.8, 129.4, 128.9, 127.8, 126.9, 123.4, 123.1, 118.0, 117.5, 115.6, 55.6; IR (ATR): 3071, 2975, 2961, 2934, 2912, 1671, 1605, 1510, 1464, 1352, 1329, 1315, 1299, 1249, 1225, 1169, 1149, 1125, 1105, 1030; HRMS (DART$^+$) *m/z* [M+H]$^+$ calcd. for $C_{23}H_{17}N_2O_2$ 353.1290; found, 353.1280.

5-(4-*tert*-Butylphenyl)dibenzo[*b*,*f*][1,7]naphthyridin-6(5*H*)-one (3da)

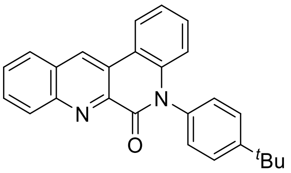

Method A; Yield: 72% as a brown solid; Mp: over 250 ºC (decomp.); ^{1}H NMR ($CDCl_3$): 9.16 (s, 1H), 8.48 (d, J = 8.8 Hz, 1H), 8.38-8.44 (m, 1H), 8.07 (d, J = 8.4 Hz, 1H), 7.84 (ddd, J = 8.4, 6.8, 0.8 Hz, 1H), 7.72 (ddd, J = 8.4, 6.8, 0.8 Hz, 1H), 7.60-7.68 (m, 2H), 7.33-7.37 (m, 1H), 6.70-6.71 (m, 1H),

6.70-6.75 (m, 1H), 1.43 (s, 9H); $^{13}C\{^1H\}$ NMR ($CDCl_3$): 160.3, 151.8, 148.6, 142.5, 139.2, 135.3, 131.3, 130.6, 130.3, 129.8, 129.4, 128.9, 128.4, 127.8, 127.3, 126.9, 123.3, 123.1, 118.0, 117.6, 34.9, 31.5; IR (ATR): 2964, 2913, 2866, 1674, 1605, 1502, 1464, 1412, 1384, 1352, 1330, 1314, 1283, 1227, 1210, 1153, 1112, 1102; HRMS (DART$^+$) *m/z* $[M+H]^+$ calcd. for $C_{26}H_{23}N_2O$ 379.1810; found, 379.1800.

5-(4-Cyanophenyl)dibenzo[*b*,*f*][1,7]naphthyridin-6(5*H*)-one (3ea)

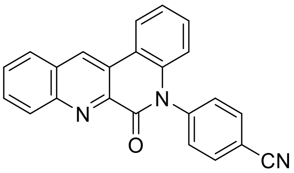

Method A; Yield: 67% as a brown solid; Mp: over 250 ºC (decomp.); ^1H NMR ($CDCl_3$): 9.17 (s, 1H), 8.47 (d, *J* = 8.4 Hz, 1H), 8.39-8.46 (m, 1H), 8.09 (d, *J* = 8.0 Hz, 1H), 7.92-8.00 (m, 2H), 7.87 (d, *J* = 7.6 Hz, 1H), 7.75 (d, *J* = 7.6 Hz, 1H), 7.51-7.61 (m, 2H), 7.35-7.43 (m, 2H), 6.59-6.67 (m, 1H); $^{13}C\{^1H\}$ NMR ($CDCl_3$): 160.0, 148.7, 148.6, 142.3, 141.9. 138.0, 134.3, 131.2, 131.0, 130.60, 130.56, 130.0, 129.5, 129.3, 127.9, 126.7, 123.7, 118.2, 118.2, 116.8, 113.3; IR (ATR): 3040, 2935, 2230, 1683, 1605, 1558, 1465, 1411, 1384, 1351, 1339, 1330, 1316, 1285, 1226, 1149, 1125; HRMS (DART$^+$) *m/z* $[M+H]^+$ calcd. for $C_{23}H_{14}N_3O$ 348.1137; found, 348.1127.

5-(4-Chlorophenyl)dibenzo[*b*,*f*][1,7]naphthyridin-6(5*H*)-one (3fa)

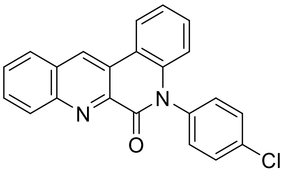

Method A; Yield: 75% as a brown solid; Mp: over 250 ºC (decomp.); ^1H NMR ($CDCl_3$): 9.16 (s, 1H), 8.48 (d, *J* = 8.4 Hz, 1H), 8.38-8.45 (m, 1H), 8.07 (d, *J* = 8.4 Hz, 1H), 7.85 (dd, *J* = 8.0, 7.6 Hz, 1H), 7.73 (dd, *J* = 7.6, 7.2 Hz, 1H), 7.58-7.66 (m, 2H), 7.34-7.42 (m, 2H), 7.31-7.36 (m, 2H), 6.68-6.75 (m, 1H); $^{13}C\{^1H\}$ NMR ($CDCl_3$): 160.3, 148.6, 142.2, 138.6, 136.6, 135.0, 131.2, 130.1, 130.7, 130.6, 130.5, 129.9, 129.4, 129.1, 127.8, 126.8, 123.5, 123.4, 118.1, 117.2; IR (ATR): 3073, 3059, 2988, 2977, 2967, 1674, 1604, 1492, 1465, 1412, 1383, 1352, 1330, 1316, 1284, 1261, 1226, 1149, 1126, 1090, 1017; HRMS (DART$^+$) *m/z* $[M+H]^+$ calcd. for $C_{22}H_{14}{}^{35}ClN_2O$ 357.0795; found, 357.0790.

5-(4-Nitrophenyl)dibenzo[*b*,*f*][1,7]naphthyridin-6(5*H*)-one (3ga)

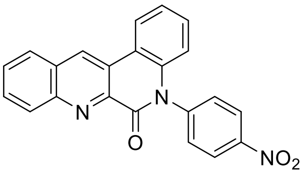

Method A; Yield: 18% as a yellow solid; Mp: over 250 ºC (decomp.); ^{1}H NMR ($CDCl_3$): 9.18 (s, 1H), 8.50-8.56 (m, 2H), 8.48 (d, *J* = 9.2 Hz, 1H), 8.43-8.47 (m, 1H), 8.10 (d, *J* = 8.0 Hz), 7.88 (ddd, *J* = 8.0, 6.8, 1.2 Hz, 1H), 7.76 (ddd, *J* = 8.0, 6.8, 0.8 Hz, 1H), 7.60-7.65 (m, 2H), 7.36-7.44 (m, 1H), 6.62-6.68 (m, 1H); ^{13}C{^{1}H} NMR ($CDCl_3$): 160.1, 148.7, 148.1, 144.0, 141.9, 138.0, 131.3, 131.1, 130.8, 130.7, 130.1, 129.6, 129.4, 127.9, 126.8, 125.9, 123.9, 123.8, 118.3, 116.8; IR (ATR): 3087, 1678, 1606, 1593, 1523, 1494, 1466, 1348, 1312, 1225, 1149, 1127, 1103, 1089; HRMS (DART^{+}) *m/z* $[M+H]^{+}$ calcd. for $C_{22}H_{14}N_3O_3$ 368.1035; found, 368.1028.

5-Mesityldibenzo[*b*,*f*][1,7]naphthyridin-6(5*H*)-one (3ha)

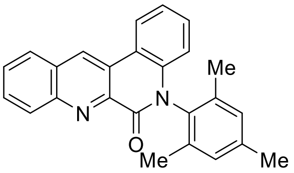

Method A; Yield: 21% as a brown solid; Mp: over 89.4–94.1 ºC; ^{1}H NMR ($CDCl_3$): 9.18 (s, 1H), 8.49 (d, *J* = 8.8 Hz, 1H), 8.41-8.46 (m, 1H), 8.08 (d, *J* = 8.4 Hz, 1H), 7.85 (dd, *J* = 7.6, 7.2 Hz, 1H), 7.72 (dd, *J* = 8.0, 7.2 Hz, 1H), 7.32-7.42 (m, 2H), 7.10 (s, 2H), 6.58-6.68 (m, 1H), 2.40 (s, 3H), 2.01 (s, 6H); ^{13}C{^{1}H} NMR ($CDCl_3$): 159.4, 148.5, 142.5, 138.9, 137.5, 135.8, 133.3, 131.4, 130.7, 130.48, 130.45, 130.1, 129.5, 129.1, 127.8, 127.1, 123.6, 123.4, 118.3, 116.2, 21.4, 17.7; IR (ATR): 2954, 2924, 2848, 1671, 1605, 1493, 1464, 1410, 1383, 1352, 1329, 1315, 1284, 1260, 1224, 1100, 1030; HRMS (DART^{+}) *m/z* $[M+H]^{+}$ calcd. for $C_{25}H_{21}N_2O$ 365.1654; found, 365.1650.

5-(2-Nitrophenyl)dibenzo[*b*,*f*][1,7]naphthyridin-6(5*H*)-one (3ia)

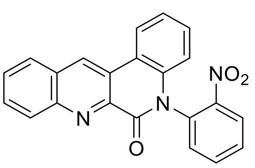

Method A; Yield: 14% as a yellow solid; Mp: over 250 ºC (decomp.); ^{1}H NMR ($CDCl_3$): 9.17 (s, 1H), 8.41-8.50 (m, 2H), 8.38 (dd, *J* = 8.0, 1.2 Hz, 1H), 8.09 (d, *J* = 8.0 Hz, 1H), 7.91 (ddd, *J* = 8.0, 7.6, 1.2 Hz, 1H), 7.86 (ddd, *J* = 8.4, 7.2, 1.2 Hz, 1H), 7.78 (ddd, *J* = 8.4, 7.2, 1.2 Hz, 1H), 7.74 (ddd, *J* = 8.4, 7.2, 1.2 Hz, 1H), 7.54 (dd, *J* = 8.0, 1.2 Hz, 1H), 7.34-7.42 (m, 2H), 6.59-6.65 (m, 1H); ^{13}C{^{1}H} NMR ($CDCl_3$): 148.6, 146.9, 138.0, 135.3, 132.21, 132.16, 131.4, 130.9, 130.7, 130.5, 130.1, 129.9, 129.6,

129.3, 127.9, 127.0, 126.7, 123.85, 123.78, 123.3, 118.5, 116.4; IR (ATR): 2926, 1679, 1616, 1529, 1465, 1353, 1339, 1315, 1290, 1262; HRMS (DART$^+$) *m/z* [M+H]$^+$ calcd. for $C_{22}H_{14}N_3O_3$ 368.1035; found, 368.1047.

5-(2-Methoxyphenyl)dibenzo[*b*,*f*][1,7]naphthyridin-6(5*H*)-one (3ja)

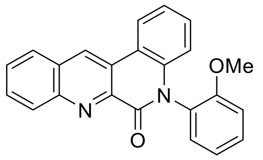

Method B; Yield: 17% as a brown solid; Mp: 207.0–211.2 ºC (decomp.); ^{1}H NMR (CDCl$_3$): 9.15 (s, 1H), 8.47 (d, *J* = 8.4 Hz, 1H), 8.37-8.44 (m, 1H), 8.07 (d, *J* = 8.8 Hz, 1H), 7.83 (ddd, *J* = 8.4, 7.2, 1.6 Hz, 1H), 7.71 (ddd, *J* = 8.0, 7.2, 0.8 Hz, 1H), 7.54 (ddd, *J* = 9.2, 8.4, 1.6 Hz, 1H), 7.30-7.40 (m, 1H), 7.31 (dd, *J* = 8.8, 1.2 Hz, 1H), 7.14-7.22 (m, 1H), 6.69-6.77 (m, 1H), 3.14 (s, 3H); ^{13}C{^{1}H} NMR (CDCl$_3$): 160.0, 155.7, 148.5, 142.7, 138.7, 131.4, 130.7, 130.6, 130.34, 130.33, 130.0, 129.4, 128.9, 127.8, 127.1, 126.5, 123.4, 123.1, 121.8, 118.1, 116.9, 112.9, 56.0; IR (ATR): 2939, 2851, 1674, 1603, 1500, 1465, 1412, 1384, 1352, 1340, 1330, 1316, 1281, 1255, 1222, 1180, 1162, 1148, 1119, 1101, 1045, 1025; HRMS (DART$^+$) *m/z* [M+H]$^+$ calcd. for $C_{23}H_{17}N_2O_2$ 353.1290; found, 353.1295.

5-Benzyldibenzo[*b*,*f*][1,7]naphthyridin-6(5*H*)-one (3ka)

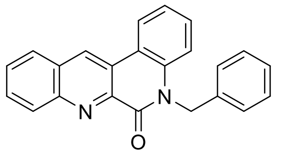

Method B; Yield: 21% as a brown solid; Mp: 232.0–235.5 ºC (decomp.); ^{1}H NMR (CDCl$_3$): 9.13 (s, 1H), 8.51 (dd, *J* = 8.4, 0.8 Hz, 1H), 8.40 (dd, *J* = 8.0, 1.2 Hz, 1H), 8.06 (d, *J* = 8.4 Hz, 1H), 7.86 (ddd, *J* = 8.8, 6.8, 1.6 Hz, 1H), 7.72 (ddd, *J* = 8.4, 6.8, 1.2 Hz, 1H), 7.45 (ddd, *J* = 8.4, 7.2, 1.6 Hz, 1H), 7.34-7.40 (m, 3H), 7.28-7.34 (m, 3H), 7.21-7.26 (m, 1H), 5.75 (br s, 2H); ^{13}C{^{1}H} NMR (CDCl$_3$): 160.9, 148.6, 142.1, 137.0, 136.4, 131.3, 130.8, 130.34, 130.32, 129.4, 129.03, 128.96, 127.9, 127.5, 127.0, 126.7, 123.7, 123.2, 118.5, 116.5, 47.3; IR (ATR): 2963, 2926, 2859, 1664, 1606, 1496, 1467, 1456, 1412, 1386, 1371, 1332, 1306, 1243, 1210, 1162, 1152, 1124, 1081; HRMS (DART$^+$) *m/z* [M+H]$^+$ calcd. for $C_{23}H_{17}N_2O$ 337.1349; found, 337.1339.

3-Methyl-5-(4-methylphenyl)dibenzo[*b*,*f*][1,7]naphthyridin-6(5*H*)-one (3ab)

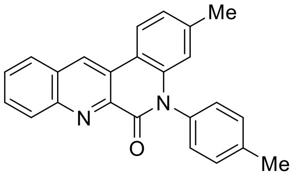

Method B; Yield: 79% as a brown solid; Mp: over 250 ºC (decomp.); ^{1}H NMR ($CDCl_3$): 9.10 (s, 1H), 8.46 (d, *J* = 8.4 Hz, 1H), 8.28 (d, *J* = 8.0 Hz, 1H), 8.05 (d, *J* = 8.0 Hz, 1H), 7.82 (ddd, *J* = 8.4, 6.8, 1.2 Hz, 1H), 7.70 (ddd, *J* = 8.4, 6.8, 0.8 Hz, 1H), 7.40-7.47 (m, 2H), 7.22-7.30 (m, 2H), 7.16 (dd, *J* = 8.0, 0.8 Hz, 1H), 6.53 (s, 1H), 2.51 (s, 3H), 2.32 (s, 3H); ^{13}C{^{1}H} NMR ($CDCl_3$): 160.5, 148.3, 142.3, 140.4, 139.1, 138.8, 135.5, 131.3, 131.0, 130.4, 129.9, 129.4, 128.8, 128.7, 127.7, 127.0, 124.3, 123.3, 117.7, 115.5, 21.9, 21.5; IR (ATR): 2972, 2961, 2930, 2921, 2873, 1722, 1673, 1616, 1592, 1541, 1513, 1490, 1456, 1400, 1381, 1352, 1340, 1326, 1286, 1227, 1211, 1192, 1159, 1147, 1106, 1066, 1047; HRMS (DART^{+}) *m/z* $[M+H]^{+}$ calcd. for $C_{24}H_{19}N_2O$ 351.1497; found, 351.1486.

2-Methyl-5-(4-methylphenyl)dibenzo[*b*,*f*][1,7]naphthyridin-6(5*H*)-one (3ac)

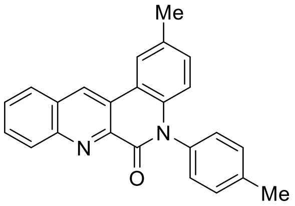

Method B; Yield: 83% as a brown solid; Mp: over 250 ºC (decomp.); ^{1}H NMR ($CDCl_3$): 9.14 (s, 1H), 8.47 (d, *J* = 8.8 Hz, 1H), 8.20 (s, 1H), 8.06 (d, *J* = 7.6 Hz, 1H), 7.83 (ddd, *J* = 8.8, 6.8, 1.2 Hz, 1H), 7.71 (ddd, *J* = 8.0, 6.8, 1.2 Hz, 1H), 7.39-7.46 (m, 2H), 7.21-7.27 (m, 2H), 7.15 (dd, *J* = 8.8, 1.6 Hz, 1H), 6.64 (d, *J* = 8.8 Hz, 1H), 2.50 (s, 3H), 2.49 (s, 3H); ^{13}C{^{1}H} NMR ($CDCl_3$): 160.2, 148.5, 142.6, 138.8, 137.0, 135.6, 132.6, 131.2, 131.0, 130.8, 130.5, 130.3, 129.3, 128.8, 128.7, 127.8, 126.8, 123.5, 117.8, 117.4, 21.5, 21.0; IR (ATR): 2970, 2922, 1673, 1620, 1510, 1486, 1462, 1381, 1339, 1315, 1280, 1226; HRMS (DART^{+}) *m/z* $[M+H]^{+}$ calcd. for $C_{24}H_{19}N_2O$ 351.1497; found, 351.1500.

3-*tert*-Butyl-5-(4-methylphenyl)dibenzo[*b*,*f*][1,7]naphthyridin-6(5*H*)-one (3ad)

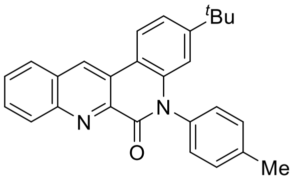

Method B; Yield: 55% as a brown solid; Mp: over 250 ºC (decomp.); ^{1}H NMR ($CDCl_3$): 9.09 (s, 1H), 8.44 (d, *J* = 8.4 Hz, 1H), 8.30 (d, *J* = 8.8 Hz, 1H), 8.04 (d, *J* = 8.4 Hz), 7.80 (dd, *J* = 8.0, 7.6 Hz, 1H), 7.68 (dd, *J* = 7.6, 7.6 Hz, 1H), 7.38-7.47 (m, 2H), 7.36 (dd, *J* = 8.0, 2.0 Hz, 1H), 7.21-7.29 (m, 2H),

6.74 (d, J = 2.0 Hz, 1H), 2.49 (s, 3H), 1.20 (s, 9H); $^{13}C\{^1H\}$ NMR ($CDCl_3$): 160.6, 153.5, 148.4, 142.5, 138.9, 138.8, 135.5, 131.3, 131.0, 130.4, 129.9, 129.4, 128.8, 128.7, 127.7, 126.9, 123.1, 120.6, 115.5, 114.4, 35.1, 31.1, 21.5; IR (ATR): 2963, 2929, 2907, 1673, 1613, 1511, 1488, 1400, 1383, 1340, 1326, 1284, 1256, 1233, 1211, 1166, 1147, 1124; HRMS (DART$^+$) m/z [M+H]$^+$ calcd. for $C_{27}H_{25}N_2O$ 393.1967; found, 393.1966.

2-*tert*-Butyl-5-(4-methylphenyl)dibenzo[*b*,*f*][1,7]naphthyridin-6(5*H*)-one (3ae)

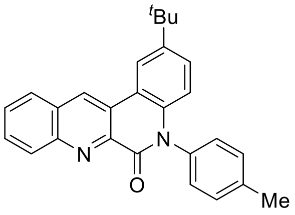

Method B; Yield: 81% as a brown solid; Mp: over 250 ºC (decomp.); ^1H NMR ($CDCl_3$): 9.16 (s, 1H), 8.47 (d, J = 8.4 Hz, 1H), 8.38 (d, J = 2.0 Hz, 1H), 8.10 (d, J = 8.0 Hz, 1H), 7.83 (ddd, J = 8.8, 6.8, 1.2 Hz, 1H), 7.71 (ddd, J = 8.4, 6.8, 0.8 Hz, 1H), 7.37-7.46 (m, 3H), 7.22-7.29 (m, 2H), 6.69, (d, J = 8.4 Hz, 1H), 2.49 (s, 3H), 1.44 (s, 9H); $^{13}C\{^1H\}$ NMR ($CDCl_3$): 160.3, 148.5, 146.0, 142.7, 138.9, 137.0, 135.5, 131.4, 131.1, 130.6, 130.1, 129.4, 128.9, 128.8, 127.8, 127.5, 127.2, 119.5, 117.4, 34.8, 31.6, 21.5; IR (ATR): 2963, 2954, 2930, 2918, 2904, 2871, 1674, 1613, 1597, 1511, 1485, 1462, 1380, 1366, 1340, 1315, 1282, 1262, 1231, 1209; HRMS (DART$^+$) m/z [M+H]$^+$ calcd. for $C_{27}H_{25}N_2O$ 393.1967; found, 393.1967.

3-Methoxy-5-(4-methylphenyl)dibenzo[*b*,*f*][1,7]naphthyridin-6(5*H*)-one (3af)

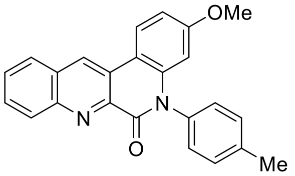

Method B; Yield: 51% as a brown solid; Mp: over 250 ºC (decomp.); ^1H NMR ($CDCl_3$): 8.98 (s, 1H), 8.43 (d, J = 8.4 Hz, 1H), 8.28 (d, J = 8.8 Hz, 1H), 8.01 (d, J = 8.0 Hz, 1H), 7.78 (ddd, J = 8.4, 7.2, 1.6 Hz, 1H), 7.67 (dd, J = 8.0, 7.2 Hz, 1H), 7.36-7.44 (m, 2H), 7.20-7.27 (m, 2H), 6.89 (dd, J = 8.8, 2.4 Hz, 1H), 6.21 (d, J = 2.4 Hz, 1H), 3.71 (s, 3H), 2.47 (s, 3H); $^{13}C\{^1H\}$ NMR ($CDCl_3$): 160.8, 160.7, 148.0, 141.8, 140.6, 138.9, 135.4, 131.3, 131.1, 130.1, 129.5, 129.2, 128.9, 128.6, 127.6, 127.1, 124.8, 111.4, 109.5, 102.7, 55.5, 21.5; IR (ATR): 2963, 2923, 2852, 1673, 1612, 1512, 1491, 1452, 1406, 1383, 1352, 1341, 1327, 1304, 1283, 1229, 1179, 1150, 1130, 1105, 1048; HRMS (DART$^+$) m/z [M+H]$^+$ calcd. for $C_{24}H_{19}N_2O_2$ 367.1447; found, 367.1431.

2-Methoxy-5-(4-methylphenyl)dibenzo[*b*,*f*][1,7]naphthyridin-6(5*H*)-one (3ag)

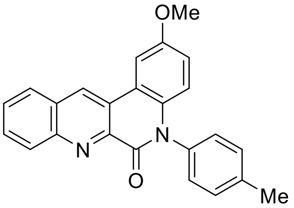

Method B; Yield: 82% as a brown solid; Mp: over 250 ºC (decomp.); ^{1}H NMR ($CDCl_3$): 9.08 (s, 1H), 8.47 (d, *J* = 8.4 Hz, 1H), 8.06 (d, *J* = 8.4 Hz, 1H), 7.79-7.91 (m, 2H), 7.70 (dd, *J* = 7.6, 6.8 Hz, 1H), 7.38-7.46 (m, 2H), 7.21-7.29 (m, 2H), 6.93 (dd, *J* = 9.2, 2.8 Hz, 1H), 6.67 (d, *J* = 9.2 Hz, 1H), 3.94 (s, 3H), 2.48 (s, 3H); $^{13}C\{^1H\}$ NMR ($CDCl_3$): 159.9, 155.6, 148.6, 142.7, 138.9, 135.7, 133.4, 131.3, 131.1, 130.7, 130.5, 129.3, 129.0, 128.8, 127.8, 126.6, 118.9, 118.8, 116.6, 107.3, 56.0, 21.5; IR (ATR): 2921, 1669, 1618, 1598, 1581, 1507, 1488, 1469, 1440, 1403, 1383, 1340, 1326, 1299, 1239, 1223, 1184, 1146, 1105, 1043; HRMS ($DART^+$) *m/z* $[M+H]^+$ calcd. for $C_{24}H_{19}N_2O_2$ 367.1447; found, 367.1434.

3-Cyano-5-(4-methylphenyl)dibenzo[*b*,*f*][1,7]naphthyridin-6(5*H*)-one (3ah)

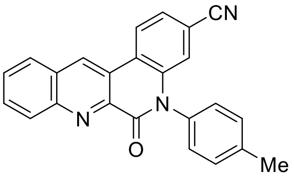

Method A; Yield: 26% as a brown solid; Mp: over 250 ºC (decomp.); ^{1}H NMR ($CDCl_3$): 9.18 (s, 1H), 8.50 (d, *J* = 8.8 Hz, 1H), 8.49 (d, *J* = 8.4 Hz, 1H), 8.11 (d, *J* = 8.4 Hz, 1H), 7.91 (ddd, *J* = 8.4, 6.8, 1.6 Hz, 1H), 7.78 (ddd, *J* = 8.4, 6.8, 1.6 Hz, 1H), 7.59 (dd, *J* = 8.4, 2.0 Hz, 1H), 7.44-7.50 (m, 2H), 7.20-7.25 (m, 2H), 7.03 (d, *J* = 2.0 Hz, 1H), 2.53 (s, 3H); $^{13}C\{^1H\}$ NMR ($CDCl_3$): 160.0, 149.3, 142.5, 139.9, 139.4, 134.4, 131.8, 131.6, 131.4, 129.6, 129.3, 128.5, 128.1, 125.9, 125.4, 124.3, 121.9, 121.2, 118.4, 112.9, 21.5; IR (ATR): 2928, 2918, 2228, 1678, 1592, 1521, 1503, 1481, 1445, 1408, 1377, 1335, 1317, 1298, 1258, 1226, 1192; HRMS ($DART^+$) *m/z* $[M+H]^+$ calcd. for $C_{24}H_{16}N_3O$ 362.1293; found, 362.1305.

3-Nitro-5-(4-methylphenyl)dibenzo[*b*,*f*][1,7]naphthyridin-6(5*H*)-one (3ai)

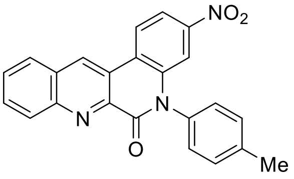

Method A; Yield: 7% as a yellow solid; Mp: over 250 ºC (decomp.); ^{1}H NMR ($CDCl_3$): 9.22 (s, 1H), 8.56 (d, *J* = 9.2 Hz, 1H), 8.51 (d, *J* = 8.4 Hz, 1H), 8.17 (dd, *J* = 8.8, 2.0 Hz, 1H), 8.13 (d, *J* = 8.4 Hz,

1H), 7.93 (ddd, J = 8.8, 6.8, 1.2 Hz, 1H), 7.79 (ddd, J = 8.4, 6.8, 1.2 Hz, 1H), 7.64 (d, J = 2.0 Hz, 1H), 7.45-7.51 (m, 2H), 7.23-7.29 (m, 2H), 2.53 (s, 3H); $^{13}C\{^1H\}$ NMR ($CDCl_3$): 160.1, 149.5, 148.2, 139.9, 139.7, 134.4, 132.1, 132.0, 131.6, 131.4, 129.7, 129.3, 128.4, 128.2, 125.1, 124.5, 123.4, 117.5, 112.8, 21.6; IR (ATR): 2926, 2854, 1718, 1678, 1618, 1588, 1526, 1491, 1448, 1382, 1342, 1317, 1287, 1263, 1221, 1149, 1131, 1118, 1093; HRMS (DART$^+$) *m/z* $[M+H]^+$ calcd. for $C_{23}H_{16}N_3O_3$ 382.1192; found, 382.1193.

5-(4-Methylpehnyl)benzothieno[2,3-*f*]benzo[*b*][1,7]naphthyridin-6(5*H*)-one (3aj)

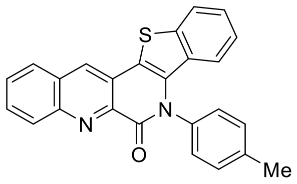

Method A; Yield: 21% as a yellow solid; Mp: over 250 ºC (decomp.); ^1H NMR ($CDCl_3$): 8.61 (s, 1H), 8.46 (d, J = 8.4 Hz, 1H), 8.03 (d, J = 8.4 Hz, 1H), 7.84 (d, J = 8.0 Hz, 1H), 7.81 (ddd, J = 8.8, 7.2, 1.2 Hz, 1H), 7.71 (ddd, J = 7.6, 6.8, 0.8 Hz, 1H), 7.41-7.47 (m, 2H), 7.34-7.40 (m, 2H), 7.31 (dd, J = 8.8, 7.6 Hz, 1H), 7.03 (ddd, J = 8.0, 7.2, 0.8 Hz, 1H), 6.13 (d, J = 8.4 Hz, 1H), 2.55 (s, 3H); $^{13}C\{^1H\}$ NMR ($CDCl_3$): 161.5, 148.4, 141.4, 139.7, 138.6, 136.4, 133.1, 131.6, 131.2, 130.9, 130.7, 130.5, 129.6, 129.3, 128.9, 127.5, 126.1, 124.7, 124.0, 123.4, 115.5, 21.7; IR (ATR): 2969, 2958, 2925, 2908, 1661, 1622, 1540, 1511, 1395, 1376, 1263; HRMS (DART$^+$) *m/z* $[M+H]^+$ calcd. for $C_{25}H_{17}N_2OS$ 393.1062; found, 393.1070.

5-(4-Methylphenyl)quinolino[2,3-*c*][1,5]naphthyridin-6(5*H*)-one (3ak)

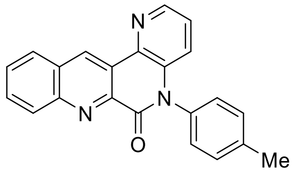

Method A; Yield: 31% as a brown solid; Mp: over 250 ºC (decomp.); ^1H NMR ($CDCl_3$): 9.78 (s, 1H), 8.60 (dd, J = 4.0, 1.2 Hz, 1H), 8.51 (d, J = 8.4 Hz, 1H), 8.16 (d, J = 8.0 Hz, 1H), 7.89 (ddd, J = 8.4, 6.8, 1.6 Hz, 1H), 7.74 (ddd, J = 8.4, 6.8, 1.2 Hz, 1H), 7.41-7.48 (m, 2H), 7.29 (dd, J = 8.0, 4.0 Hz, 1H), 7.23-7.28 (m, 2H), 2.50 (s, 3H); $^{13}C\{^1H\}$ NMR ($CDCl_3$): 160.1, 149.6, 144.4, 143.1, 139.4, 136.3, 135.5, 134.5, 133.2, 131.34, 131.29, 129.7, 129.1, 128.7, 128.6, 128.0, 124.2, 124.0, 106.3, 21.5; IR (ATR): 2922, 2854, 1680, 1606, 1511, 1464, 1438, 1417, 1382, 1355, 1318, 1262, 1213, 1197, 1145, 1128, 1109; HRMS (DART$^+$) *m/z* $[M+H]^+$ calcd. for $C_{22}H_{16}N_3O$ 338.1293; found, 338.1303.

S4: Additional data for the optimization of the C–H/N–H coupling reaction

Table S1.

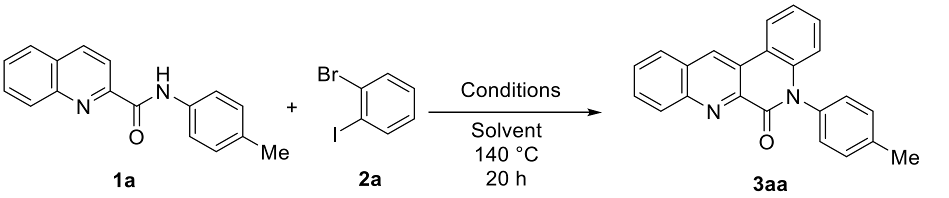

Entry	Catalyst (mol %)	Ligand (mol %)	Base (equiv)	**2a** (equiv)	Solvent (M)	Yield (%)[a]
S1	$Pd(OAc)_2$ (10)	PPh_3 (40)	Cs_2CO_3 (3.0)	1.0	DMF (0.1)	19 (52)
S2	$PdCl_2(PPh_3)_2$ (10)	none	Cs_2CO_3 (3.0)	1.0	DMF (0.1)	10 (85)
S3	$Pd(TFA)_2$ (10)	PPh_3 (40)	Cs_2CO_3 (3.0)	1.0	DMF (0.1)	39 (32)
S4	$Pd(OAc)_2$ (10)	PPh_3 (40)	K_2CO_3 (3.0)	1.0	DMF (0.1)	6 (72)
S5	$Pd(OAc)_2$ (10)	PPh_3 (40)	*t*-BuOK (3.0)	1.0	DMF (0.1)	N. R.
S6	$Pd(OAc)_2$ (10)	PPh_3 (40)	*t*-BuONa (3.0)	1.0	DMF (0.1)	9 (63)
S7	$Pd(OAc)_2$ (10)	PPh_3 (40)	Cs_2CO_3 (3.0)	1.0	DMF (1.0)	46 (28)
S8[b)]	$Pd(OAc)_2$ (10)	PPh_3 (40)	Cs_2CO_3 (3.0)	1.0	DMF (1.0)	4 (64)
S9[c)]	$Pd(OAc)_2$ (10)	PPh_3 (40)	Cs_2CO_3 (3.0)	1.0	DMF (1.0)	19 (61)
S10[d)]	$Pd(OAc)_2$ (10)	PPh_3 (40)	Cs_2CO_3 (3.0)	1.0	DMF (1.0)	40 (32)
S11	$Pd(OAc)_2$ (10)	PhDave-Phos (40)	Cs_2CO_3 (3.0)	1.0	DMF (1.0)	9 (55)
S12	$Pd(OAc)_2$ (10)	P(*p*-tolyl)$_3$ (40)	Cs_2CO_3 (3.0)	1.0	DMF (1.0)	15 (18)
S13	$Pd(OAc)_2$ (10)	PPh_3 (20)	Cs_2CO_3 (3.0)	1.0	DMF (1.0)	7 (57)
S14	$Pd(OAc)_2$ (10)	none	Cs_2CO_3	1.0	DMF (1.0)	N. R.

Entry	Catalyst (mol %)	Ligand (mol %)	Base (equiv)	**2a** (equiv)	Solvent (M)	Yield (%)[a]
			(3.0)			
S15	$Pd(TFA)_2$ (10)	PPh_3 (40)	Cs_2CO_3 (3.0)	1.0	DMF (1.0)	39 (32)
S16	$Pd(OAc)_2$ (10)	PPh_3 (40)	Cs_2CO_3 (3.0)	1.0	*o*-xylene (1.0)	34 (58)
S17	$Pd(OAc)_2$ (10)	PPh_3 (100)	Cs_2CO_3 (3.0)	1.0	*o*-xylene (1.0)	39 (30)
S18	$Pd(OAc)_2$ (10)	P(*p*-tolyl)$_3$ (40)	Cs_2CO_3 (3.0)	1.0	*o*-xylene (1.0)	35 (30)
S19	$Pd(OAc)_2$ (10)	P(*p*-MeOC$_6$H$_4$)$_3$ (40)	Cs_2CO_3 (3.0)	1.0	*o*-xylene (1.0)	44 (33)
S20	$Pd(OAc)_2$ (10)	P(4-FC$_6$H$_4$)$_3$ (40)	Cs_2CO_3 (3.0)	1.0	*o*-xylene (1.0)	21 (55)
S21	$Pd(OAc)_2$ (10)	P(*o*-tolyl)$_3$ (40)	Cs_2CO_3 (3.0)	1.0	*o*-xylene (1.0)	N. R.
S22	$Pd(OAc)_2$ (10)	PhDave-Phos (40)	Cs_2CO_3 (3.0)	1.0	*o*-xylene (1.0)	15 (58)
S23	$Pd(OAc)_2$ (10)	Xantphos (40)	Cs_2CO_3 (3.0)	1.0	*o*-xylene (1.0)	16 (59)
S24	$Pd(OAc)_2$ (10)	P(2-furyl)$_3$ (40)	Cs_2CO_3 (3.0)	1.0	*o*-xylene (1.0)	9 (62)
S25	Herrmann's catalyst (10)	none	Cs_2CO_3 (3.0)	1.0	*o*-xylene (1.0)	N. R.
S26[e)]	$Pd(OAc)_2$ (10)	P(*p*-MeOC$_6$H$_4$)$_3$ (40)	Cs_2CO_3 (3.0)	1.0 + 1.0	*o*-xylene (1.0)	53 (15)
S27	$Pd(OAc)_2$ (10)	P(*p*-MeOC$_6$H$_4$)$_3$ (80)	Cs_2CO_3 (3.0)	2.0	*o*-xylene (1.0)	45 (34)
S28	$Pd(OAc)_2$ (20)	P(*p*-MeOC$_6$H$_4$)$_3$ (80)	Cs_2CO_3 (3.0)	2.0	*o*-xylene (1.0)	81[f)]

a) Yields in parentheses correspond to recovered **1a**. The yields were determined by ^{1}H NMR spectroscopy using 1,1,2,2-tetrachloroethane as an internal standard. b) The reaction was performed at 110 °C. c) The reaction was performed at 120 °C. d) The reaction was performed at 130 °C. e) After stirring for 20 hours at 140 °C, an additional one equivalent of **2a** was added to the reaction system and the reaction was carried out for further 20 hours at 140 °C. f) Isolated yield.

S5: 2D NOESY spectra

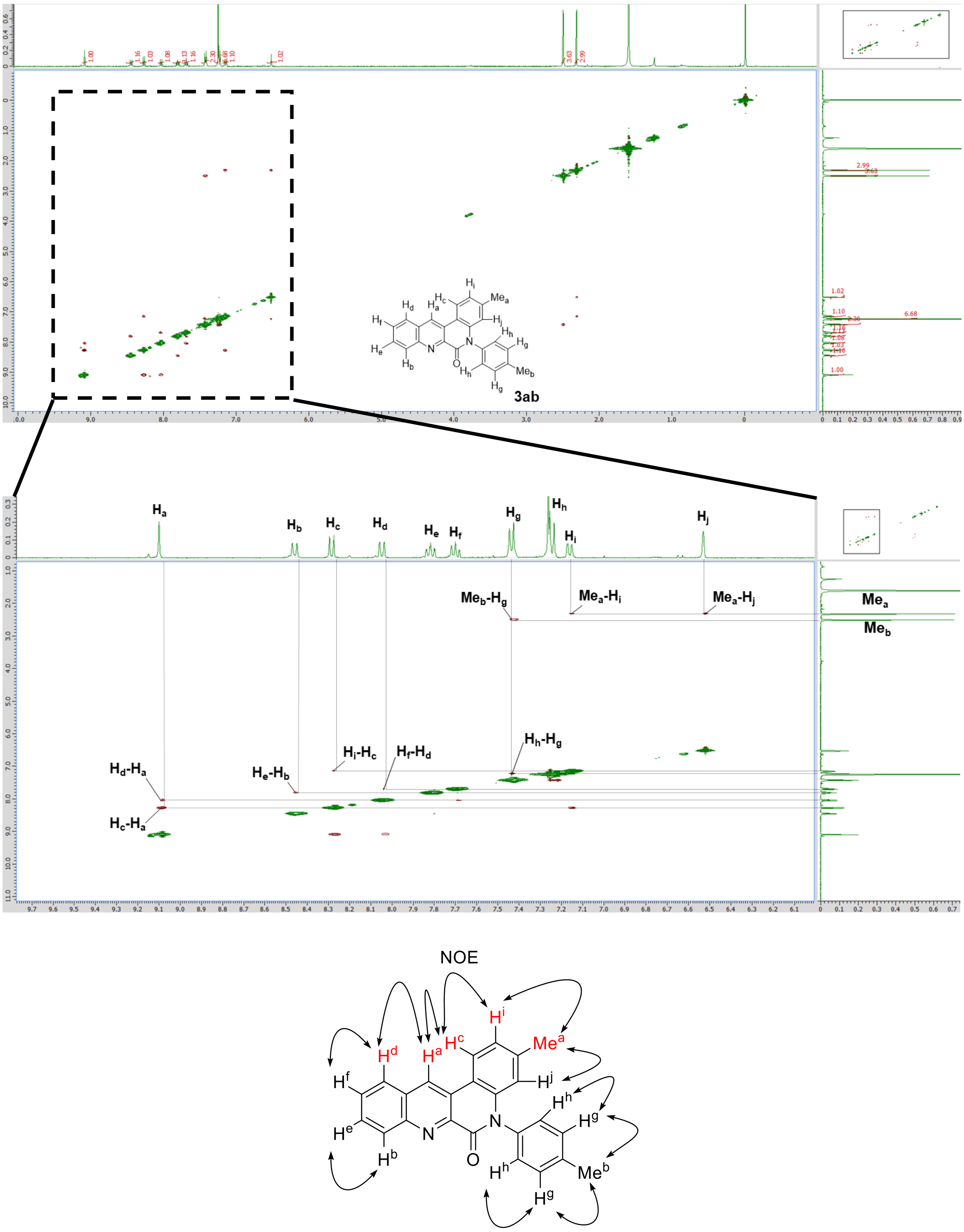

Figure S1. NOESY spectrum of **3ab**.

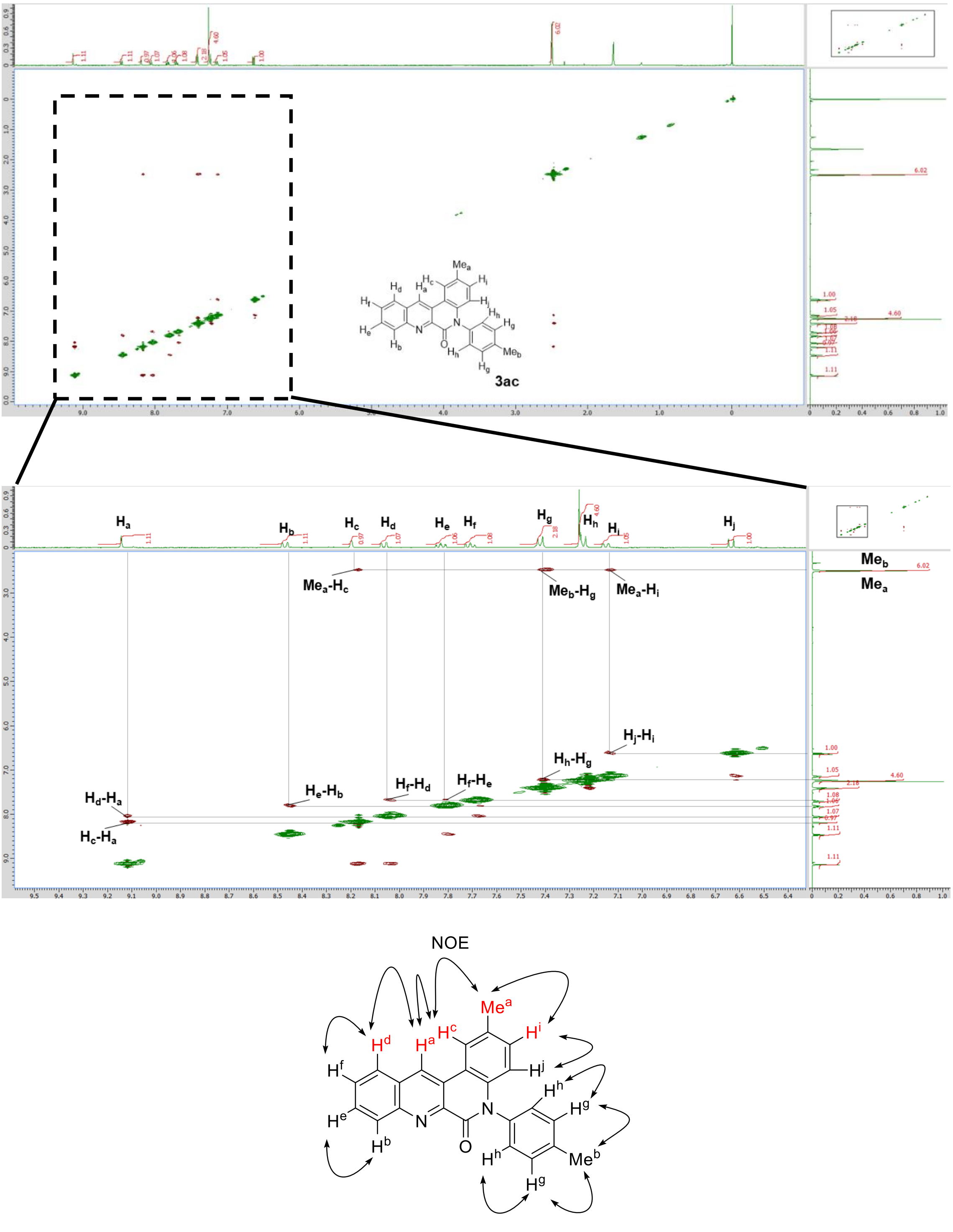

Figure S2. NOESY spectrum of **3ac**.

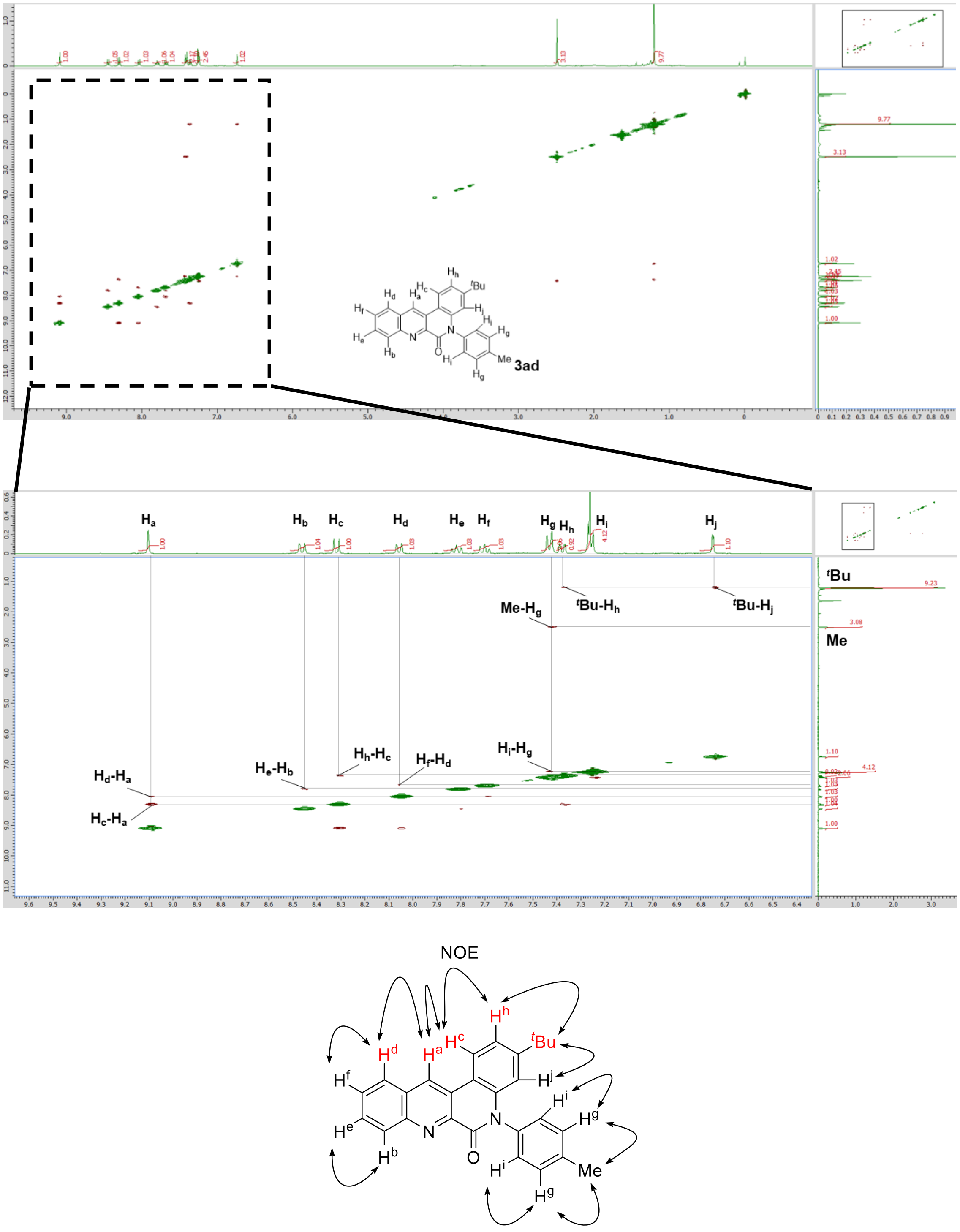

Figure S3. NOESY spectrum of **3ad**.

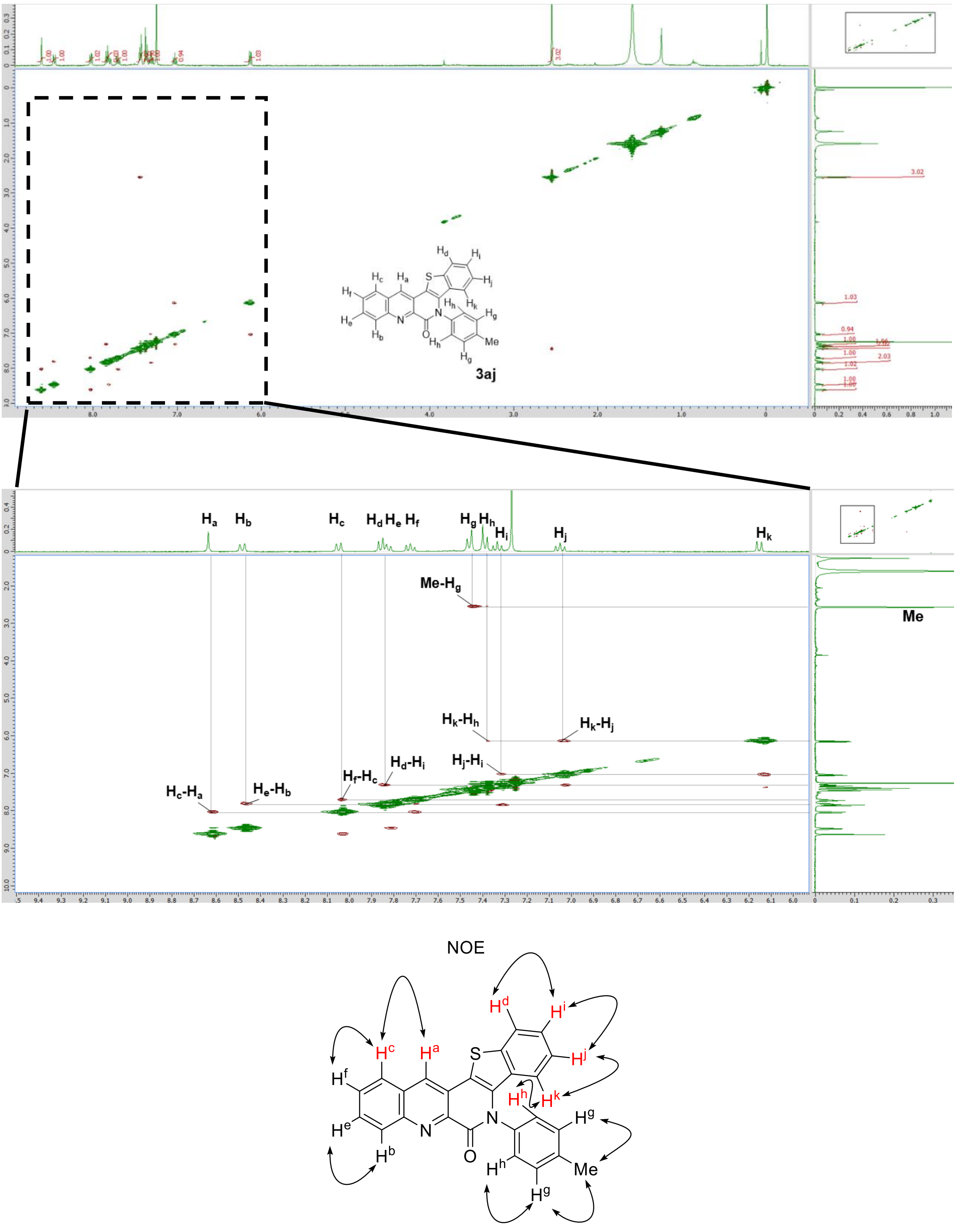

Figure S4. NOESY spectrum of **3aj**.

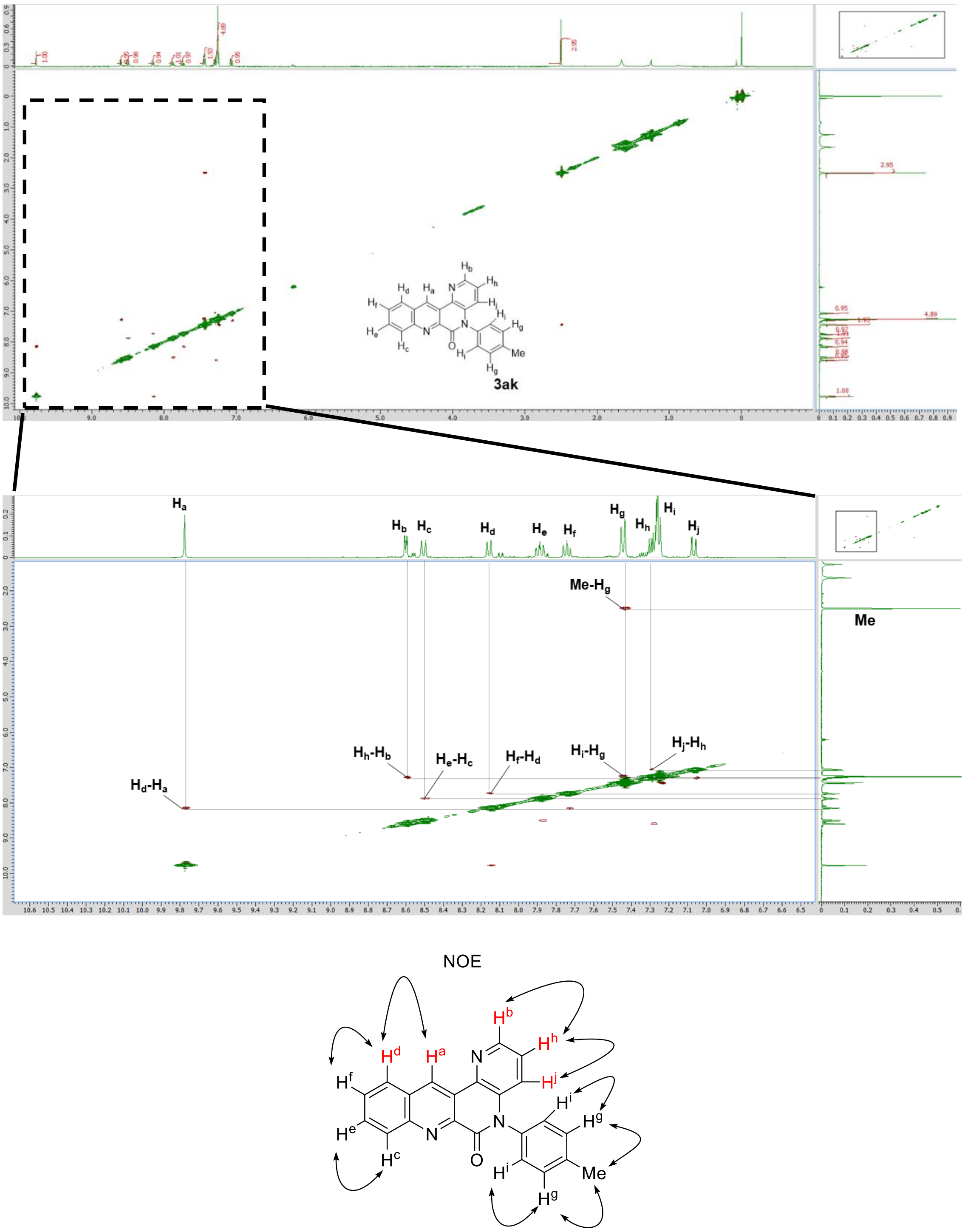

Figure S5. NOESY spectrum of **3ak**.

S6: Confirmation of the structure of Int-1ad

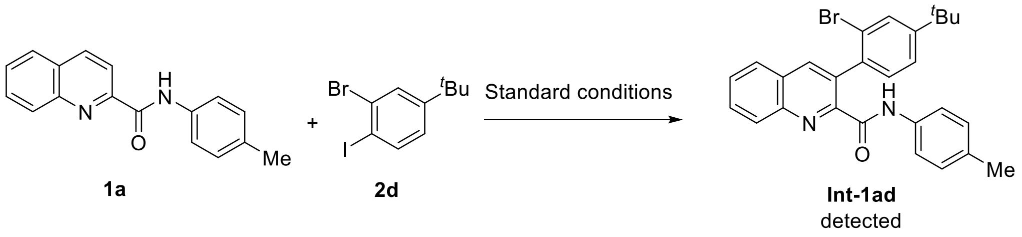

Int-1ad

HRMS (DART$^+$) *m/z* [M+Na]$^+$ calcd. for $C_{27}H_{25}{}^{79}BrN_2ONa$ calcd. for 495.1024; found, 495.1043.

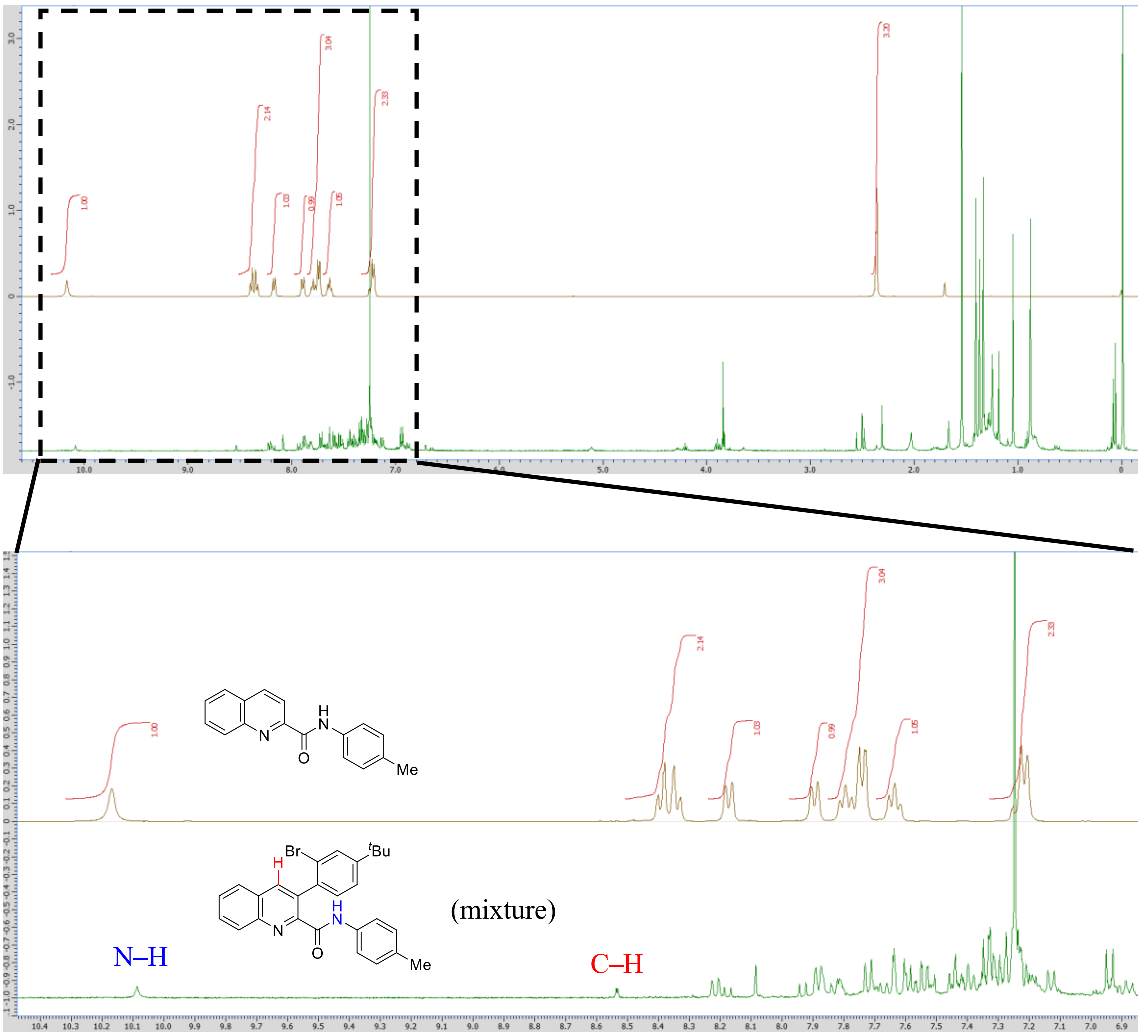

S7: NMR spectra

^{1}H NMR (400 MHz, $CDCl_3$)

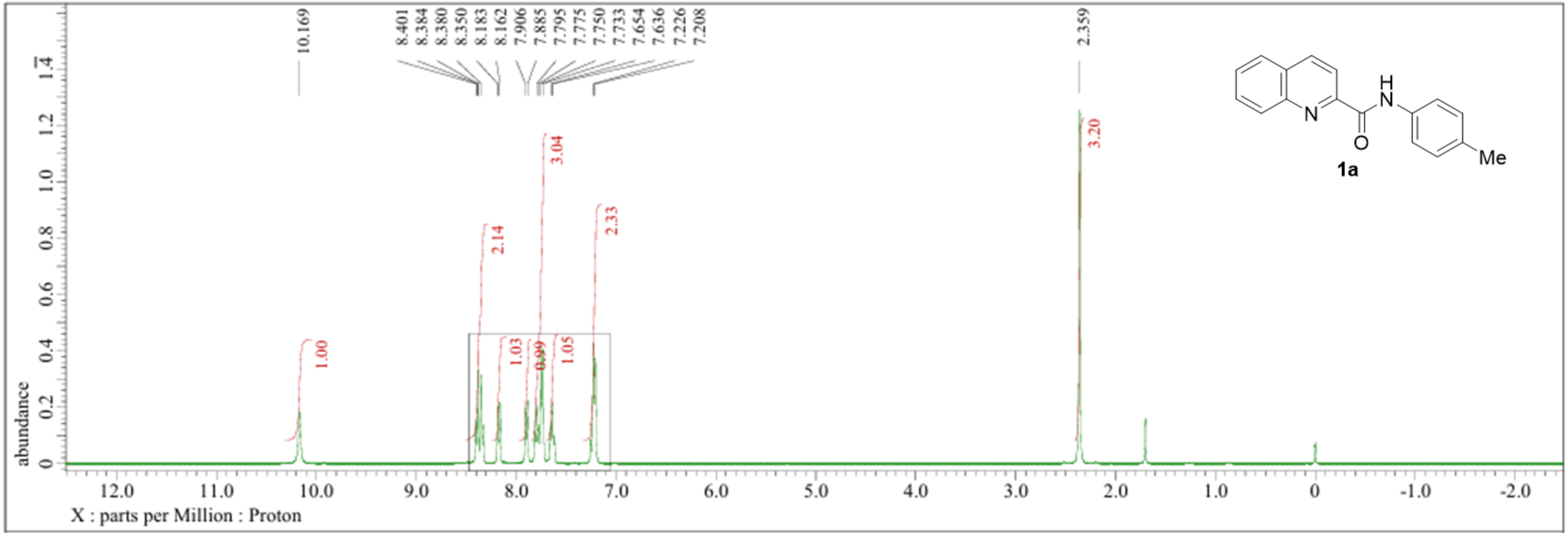

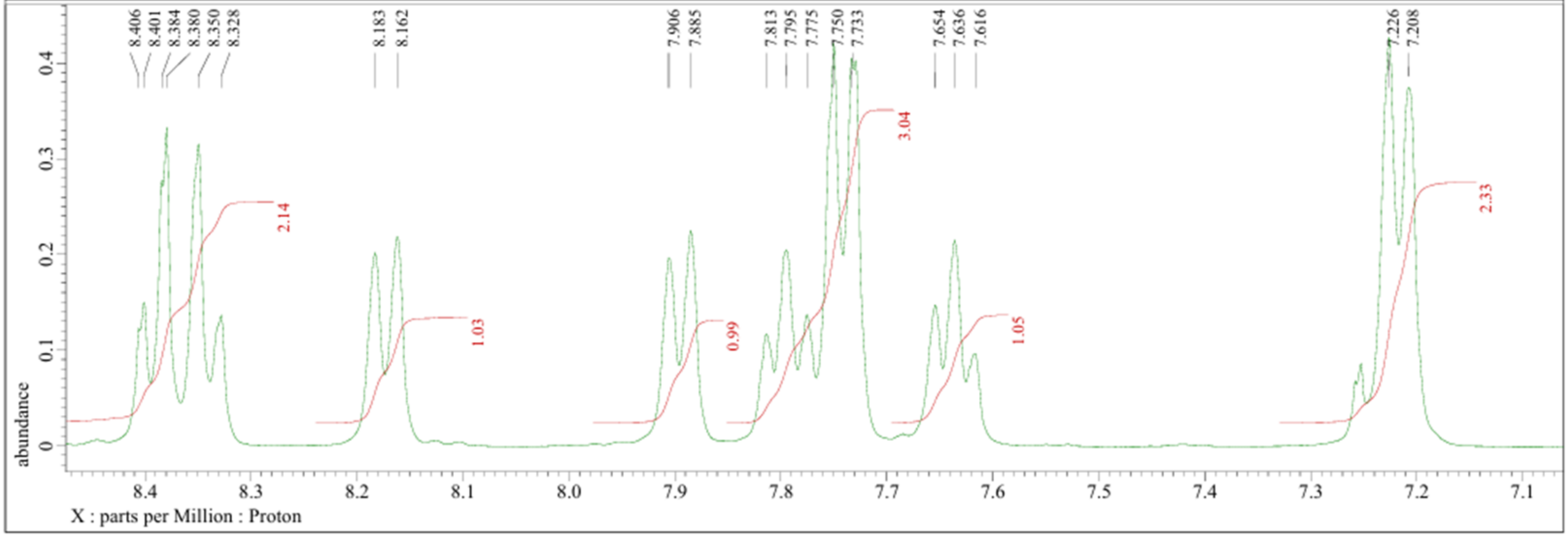

$^{13}C\{^1H\}$ NMR (100 MHz, $CDCl_3$)

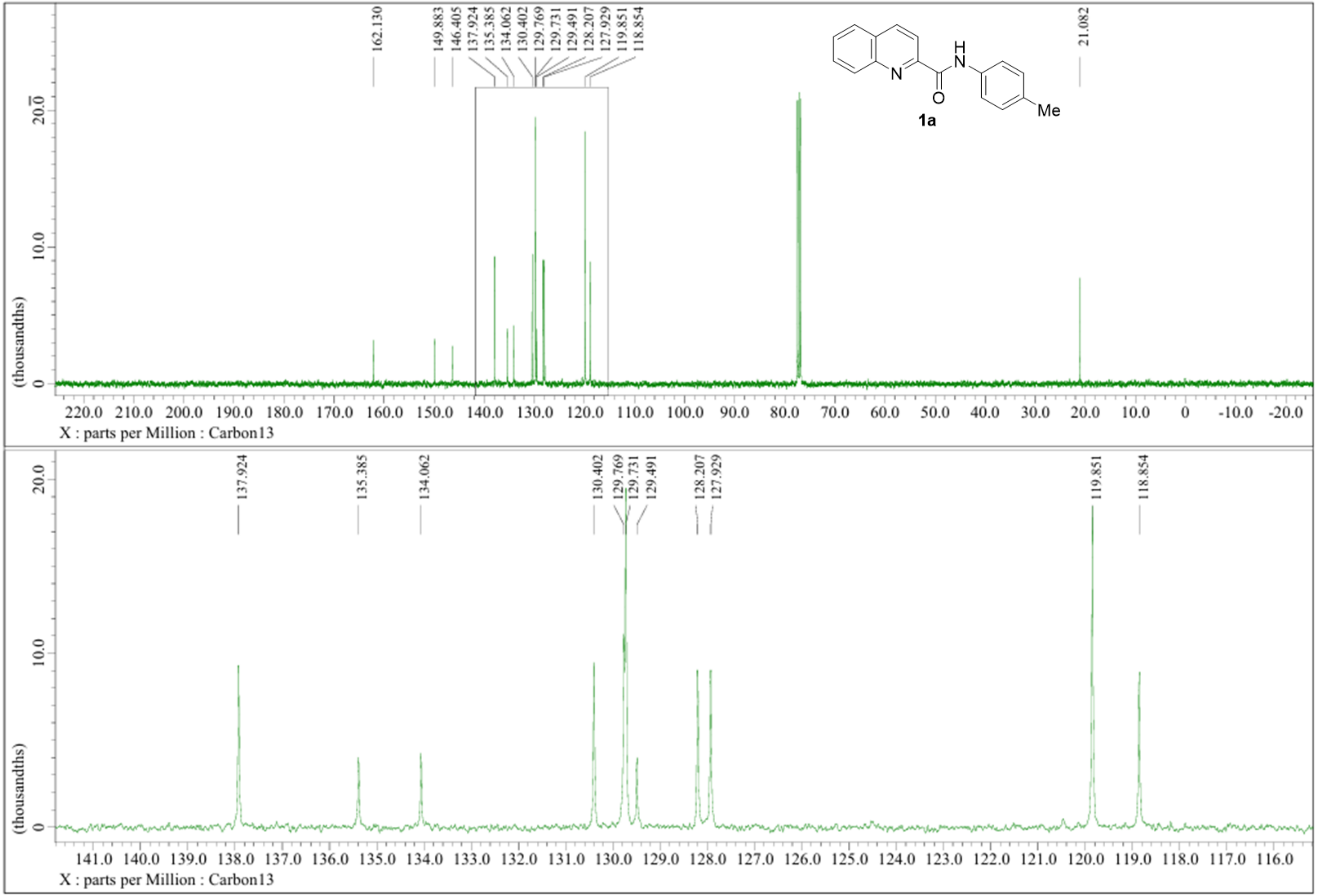

^{1}H NMR (400 MHz, $CDCl_3$)

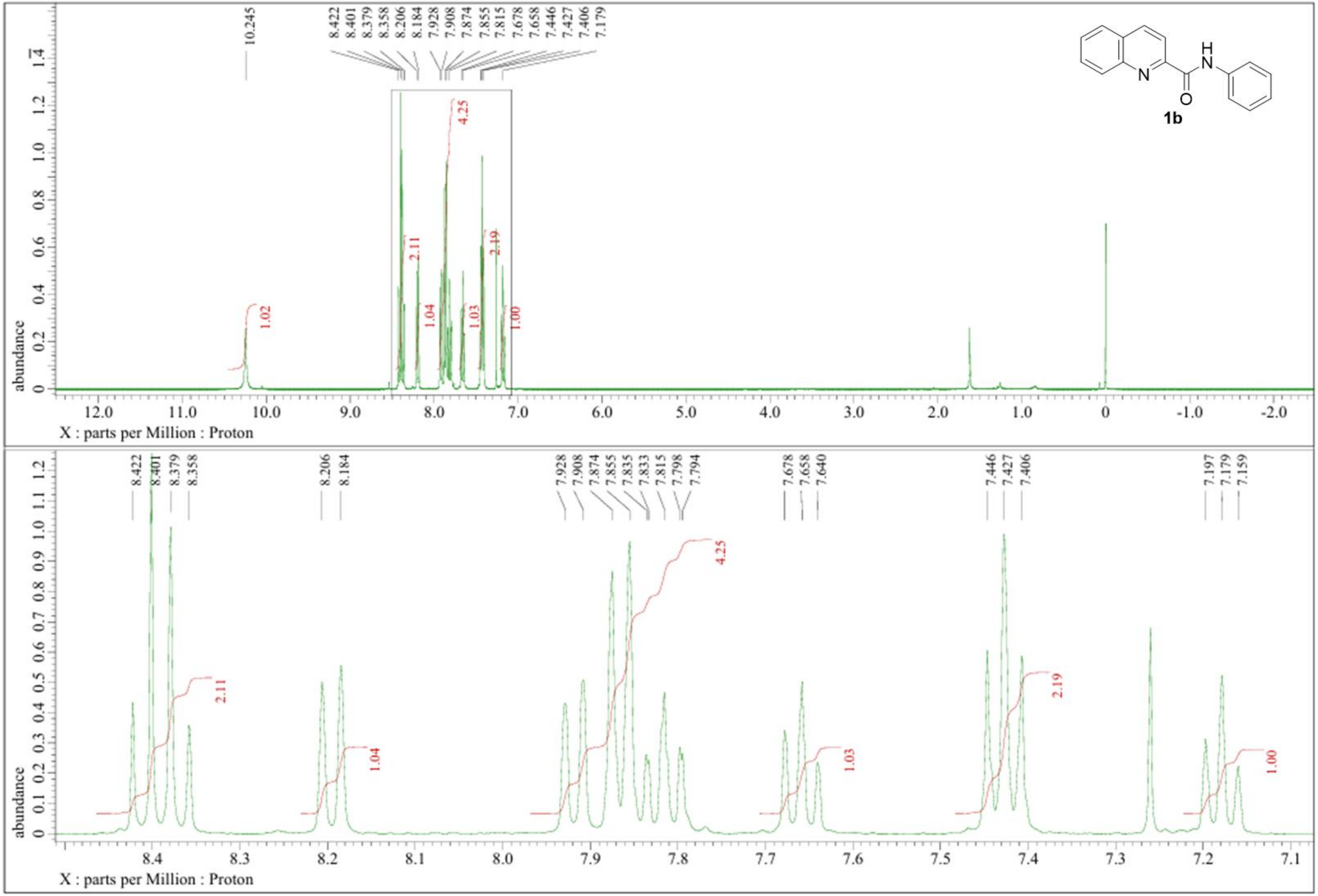

$^{13}C\{^1H\}$ NMR (100 MHz, $CDCl_3$)

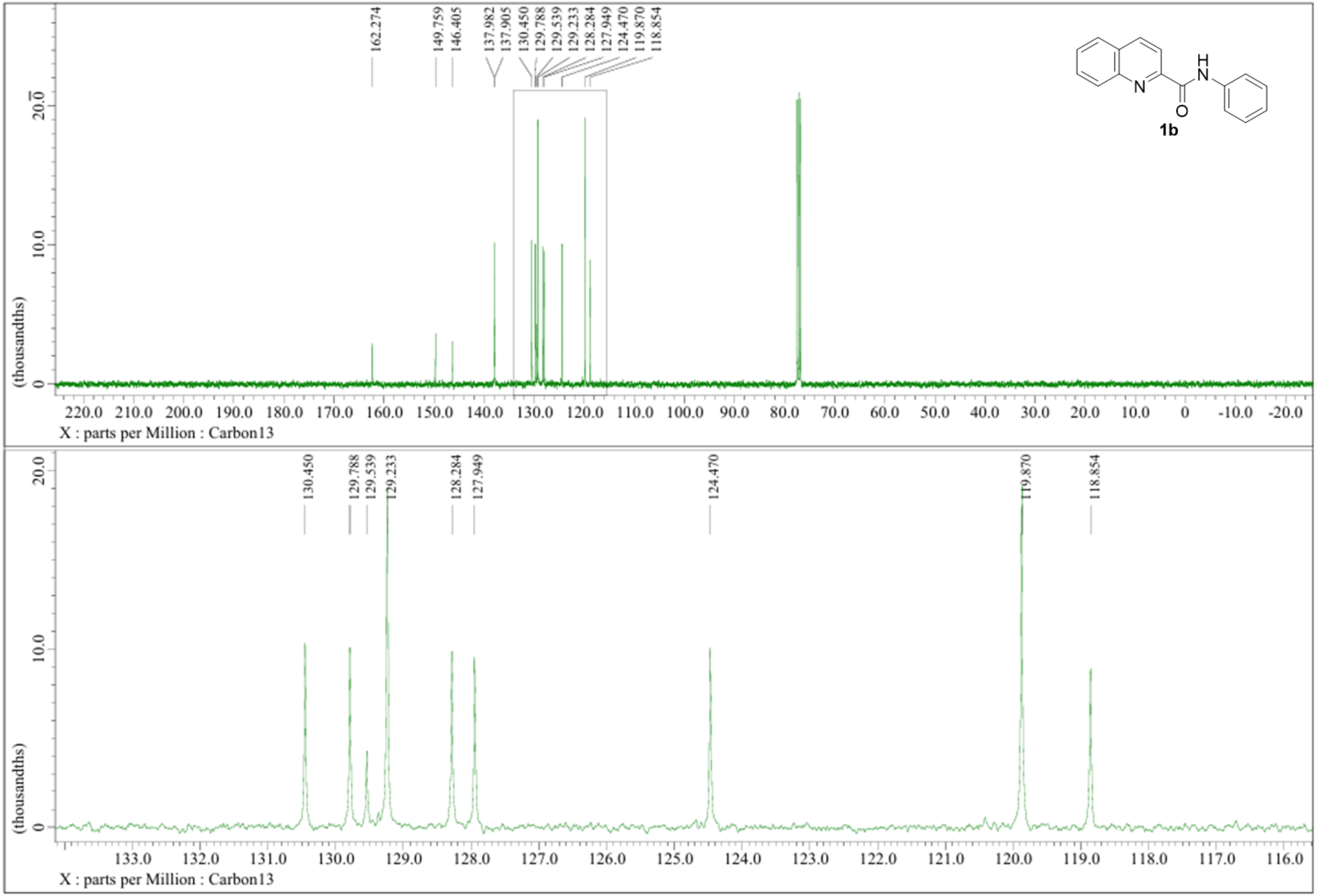

^1H NMR (400 MHz, $CDCl_3$)

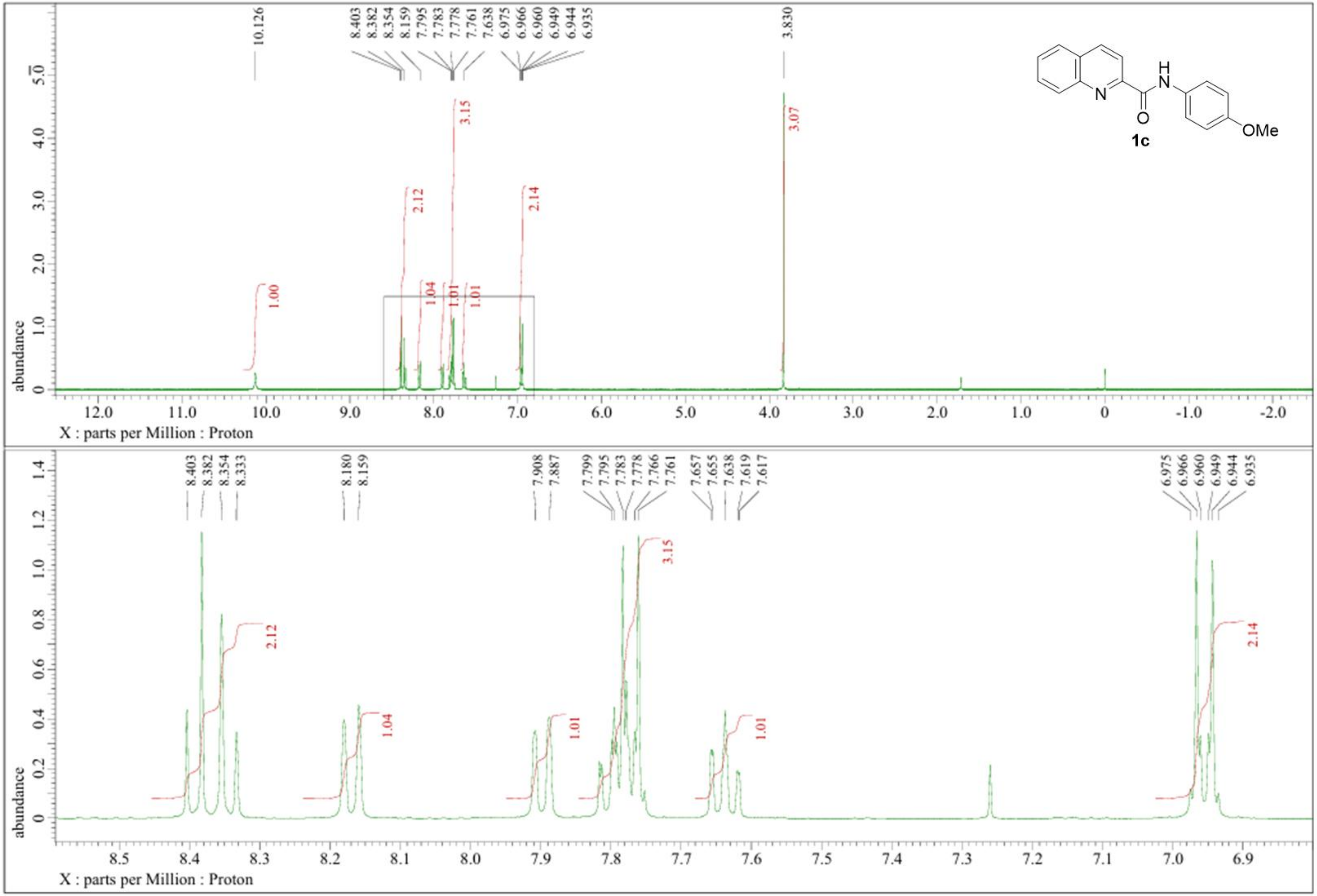

$^{13}C\{^1H\}$ NMR (100 MHz, $CDCl_3$)

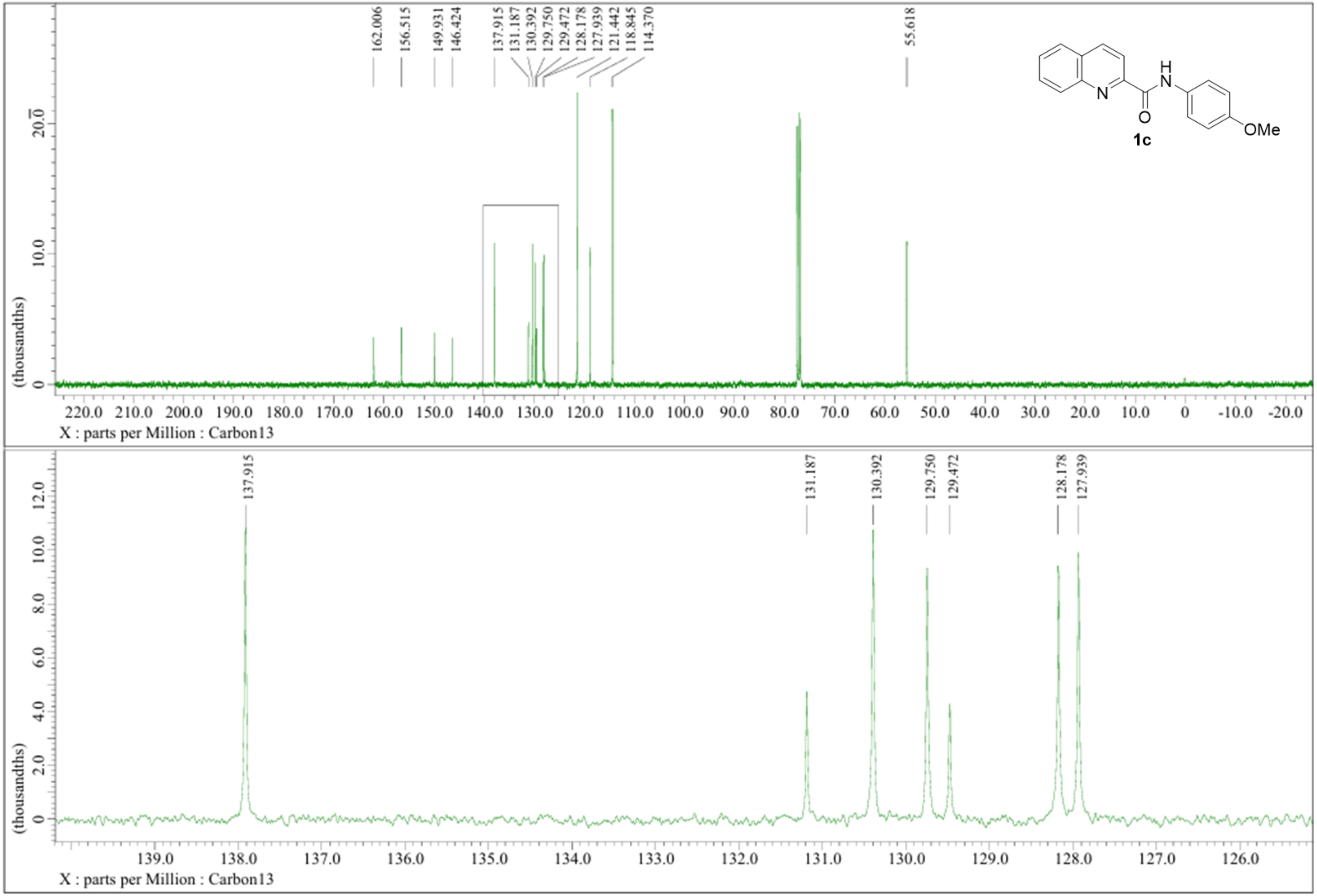

^1H NMR (400 MHz, $CDCl_3$)

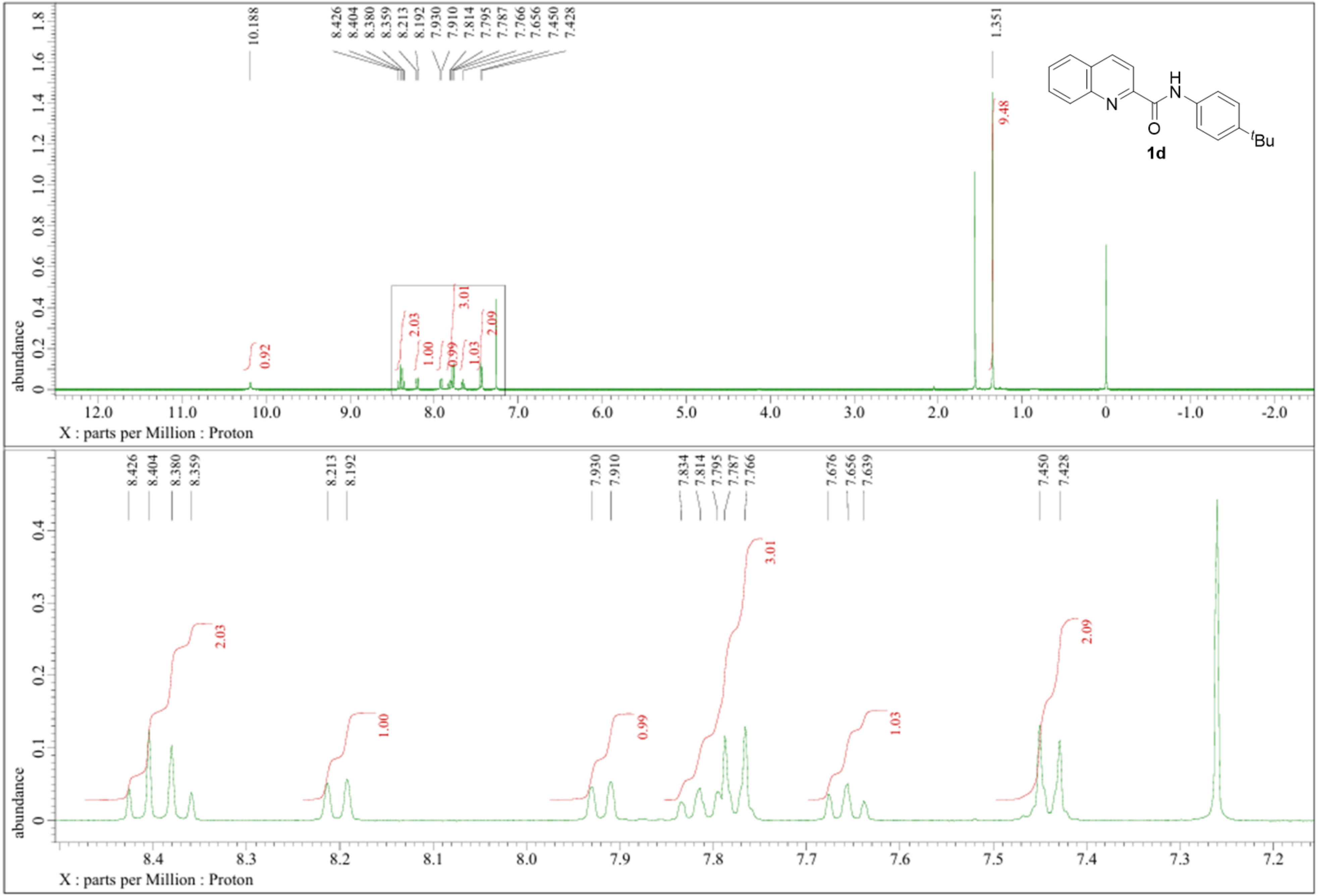

$^{13}C\{^{1}H\}$ NMR (100 MHz, $CDCl_3$)

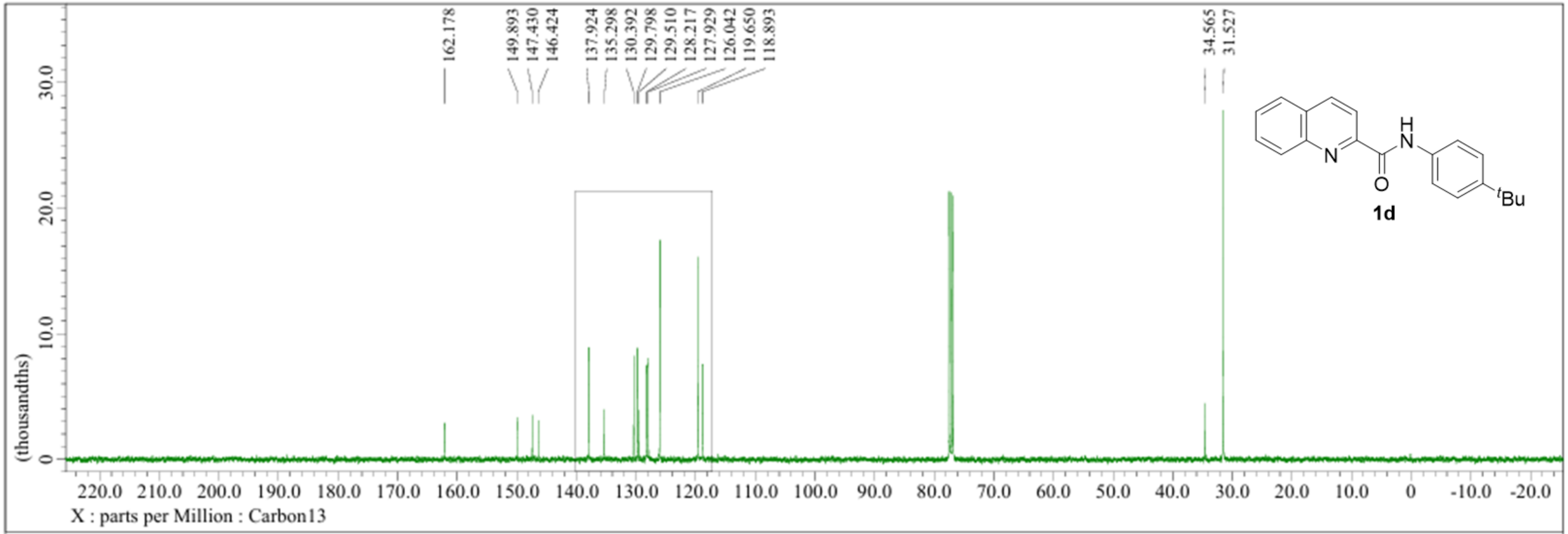

1d

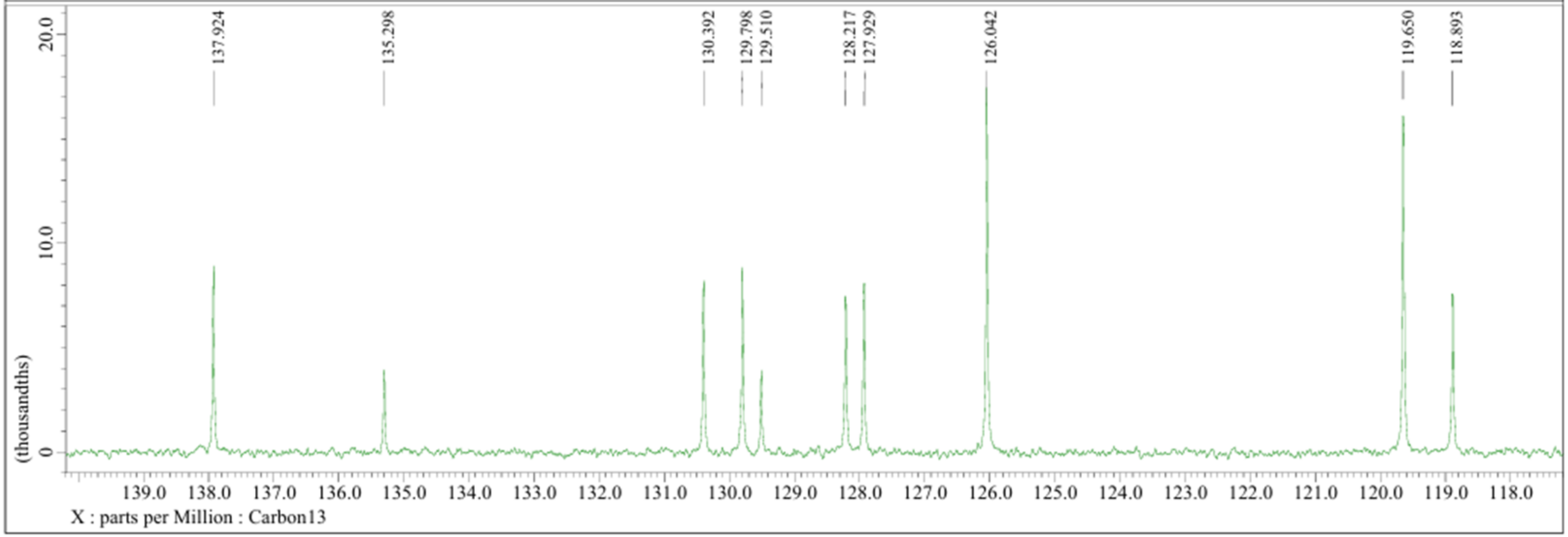

^{1}H NMR (400 MHz, $CDCl_3$)

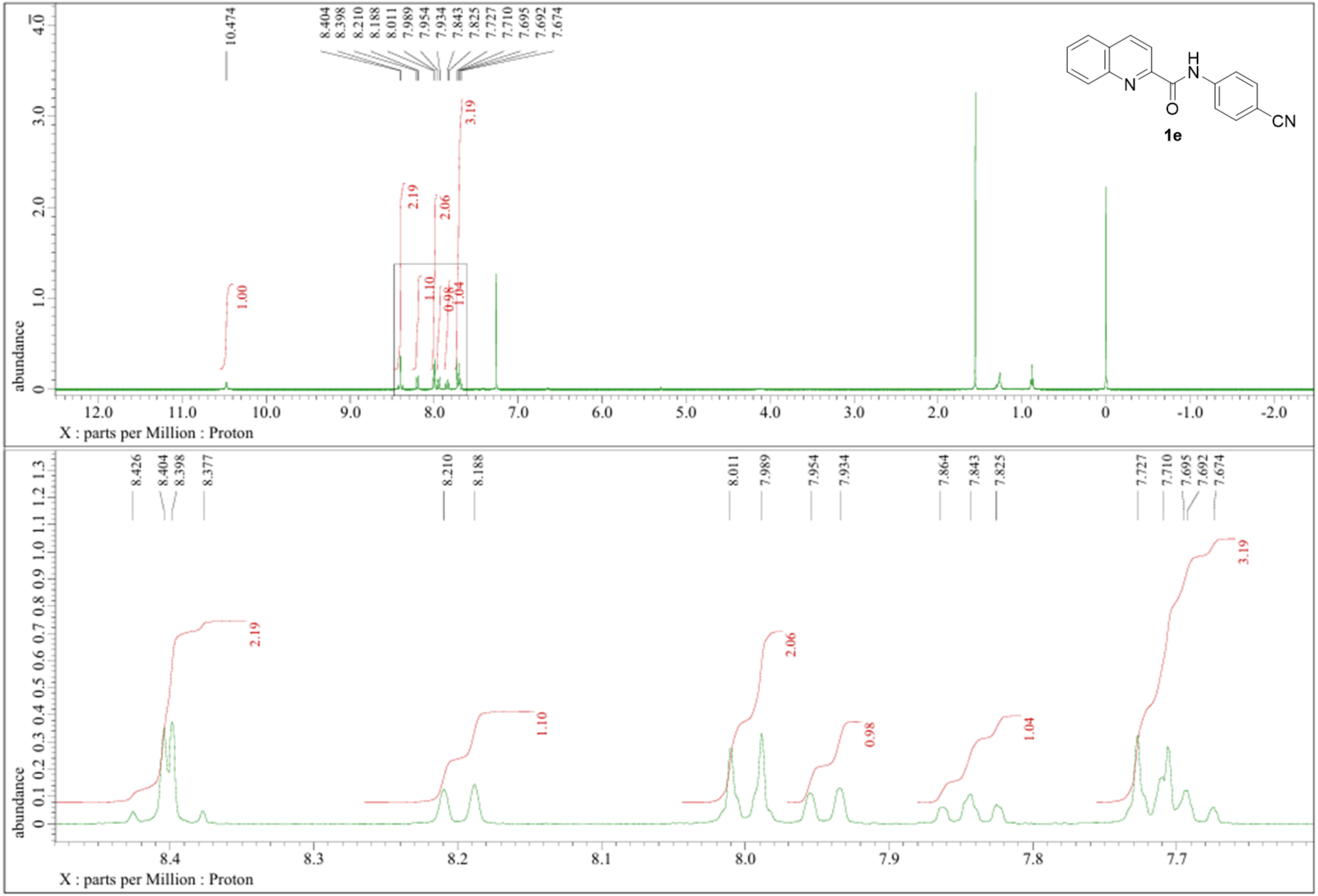

$^{13}C\{^1H\}$ NMR (100 MHz, $CDCl_3$)

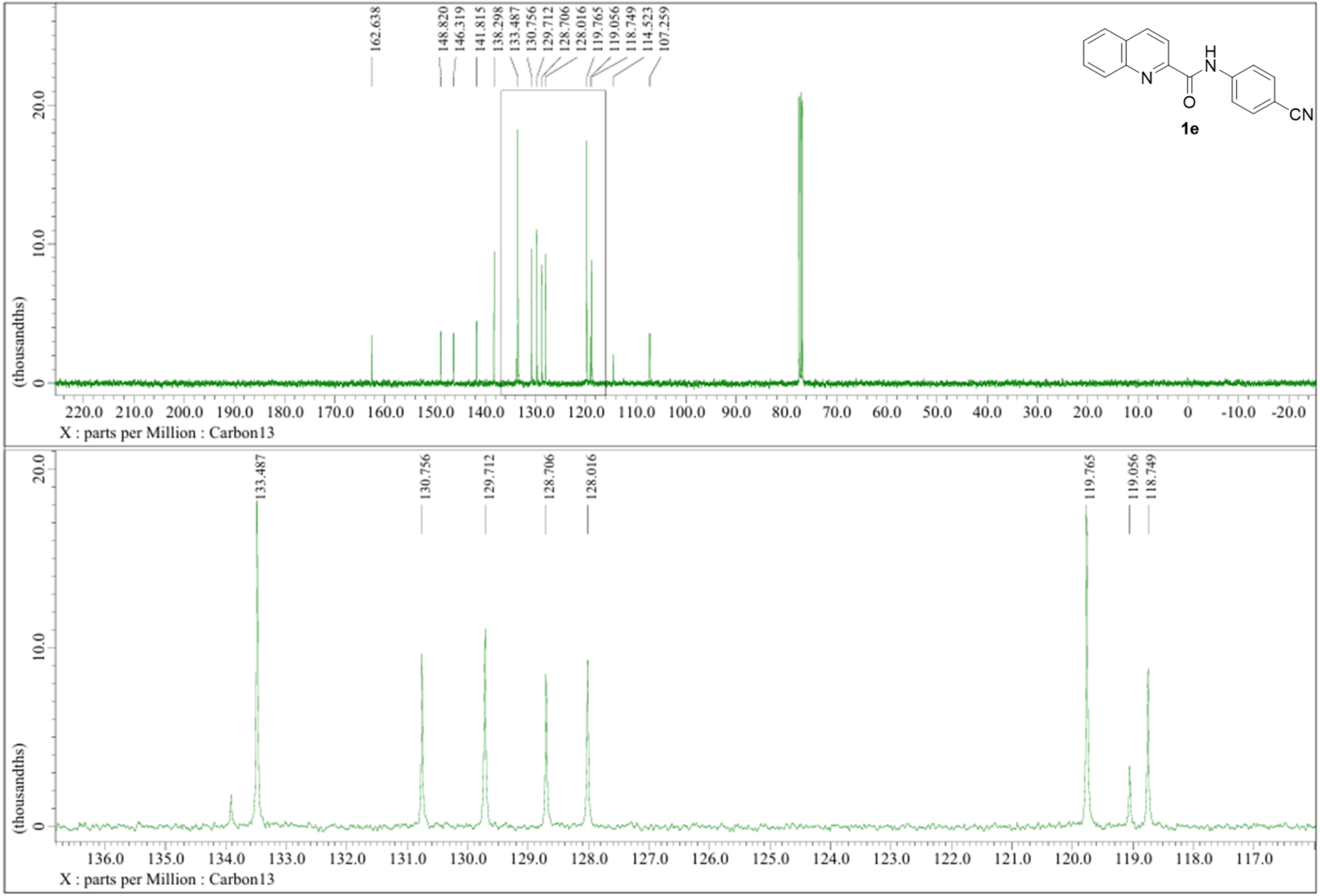

^{1}H NMR (400 MHz, $CDCl_3$)

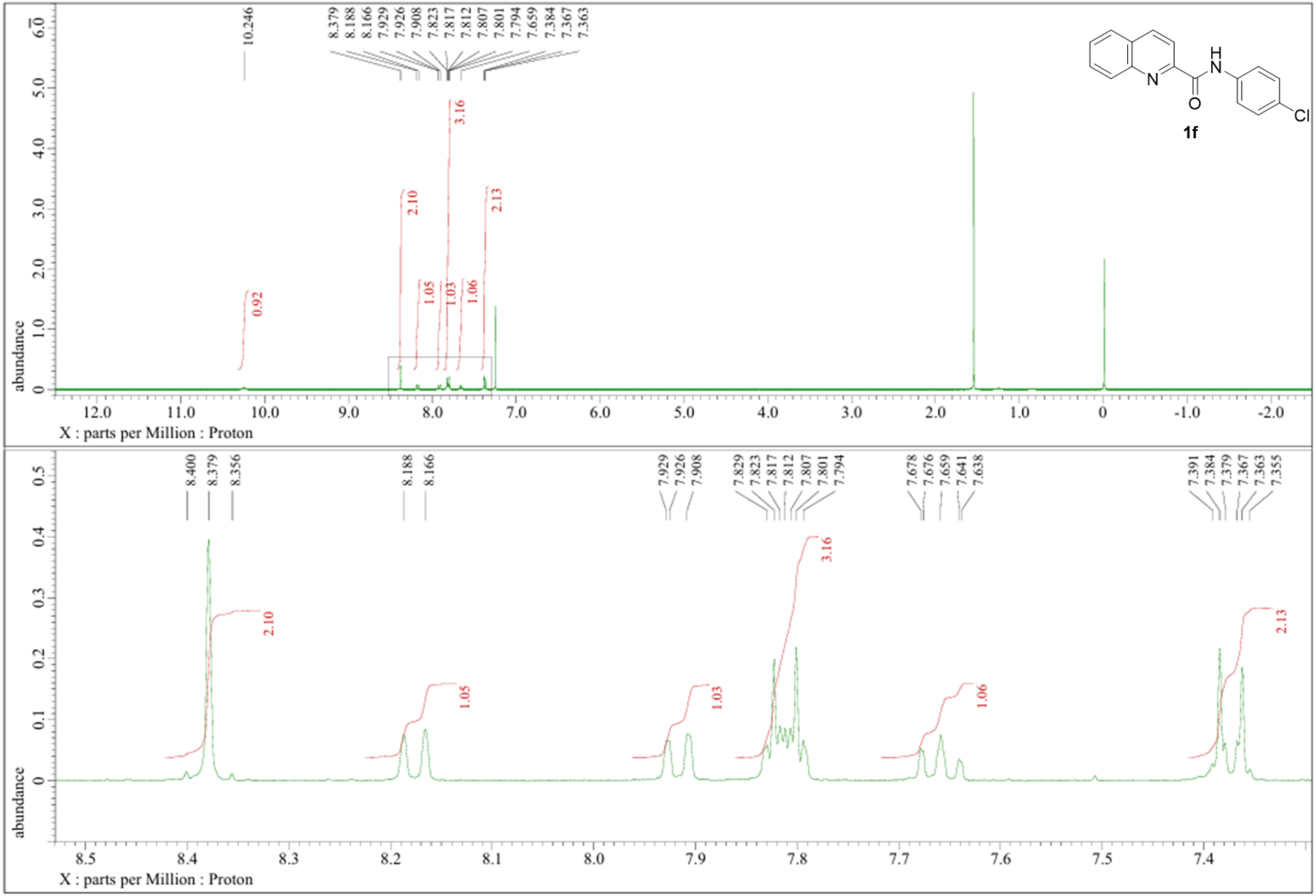

$^{13}C\{^1H\}$ NMR (100 MHz, $CDCl_3$)

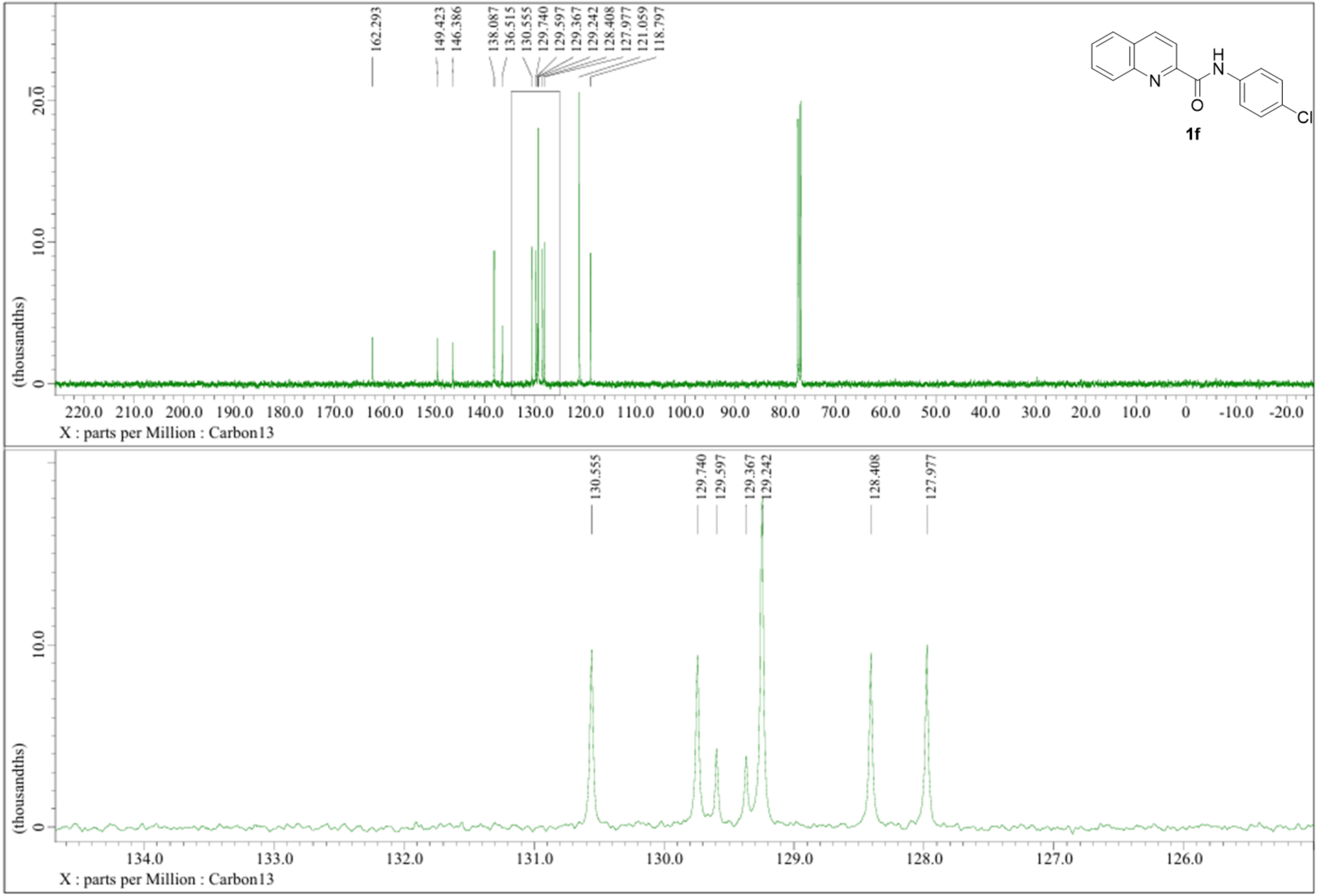

^{1}H NMR (400 MHz, $CDCl_3$)

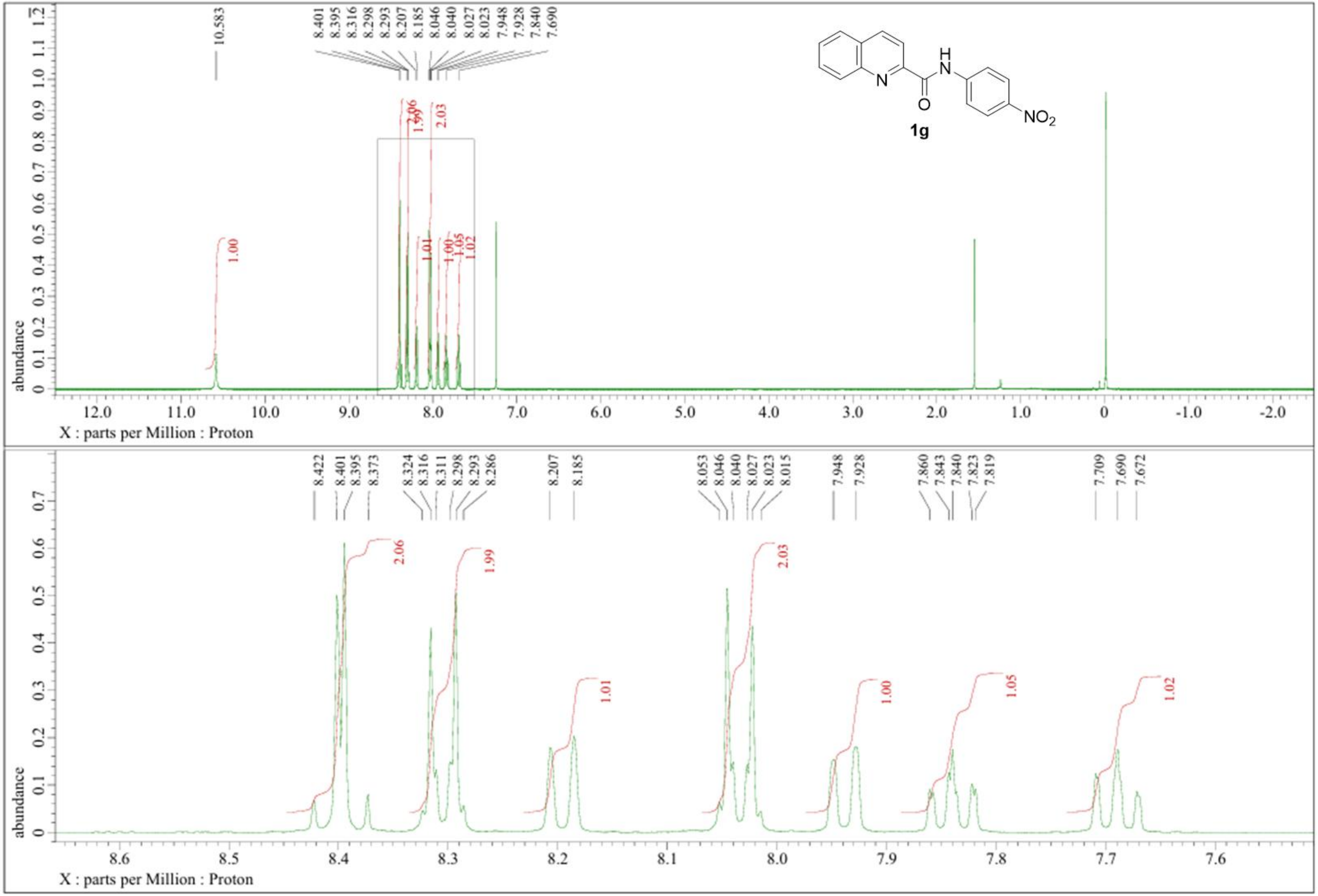

$^{13}C\{^1H\}$ NMR (100 MHz, $CDCl_3$)

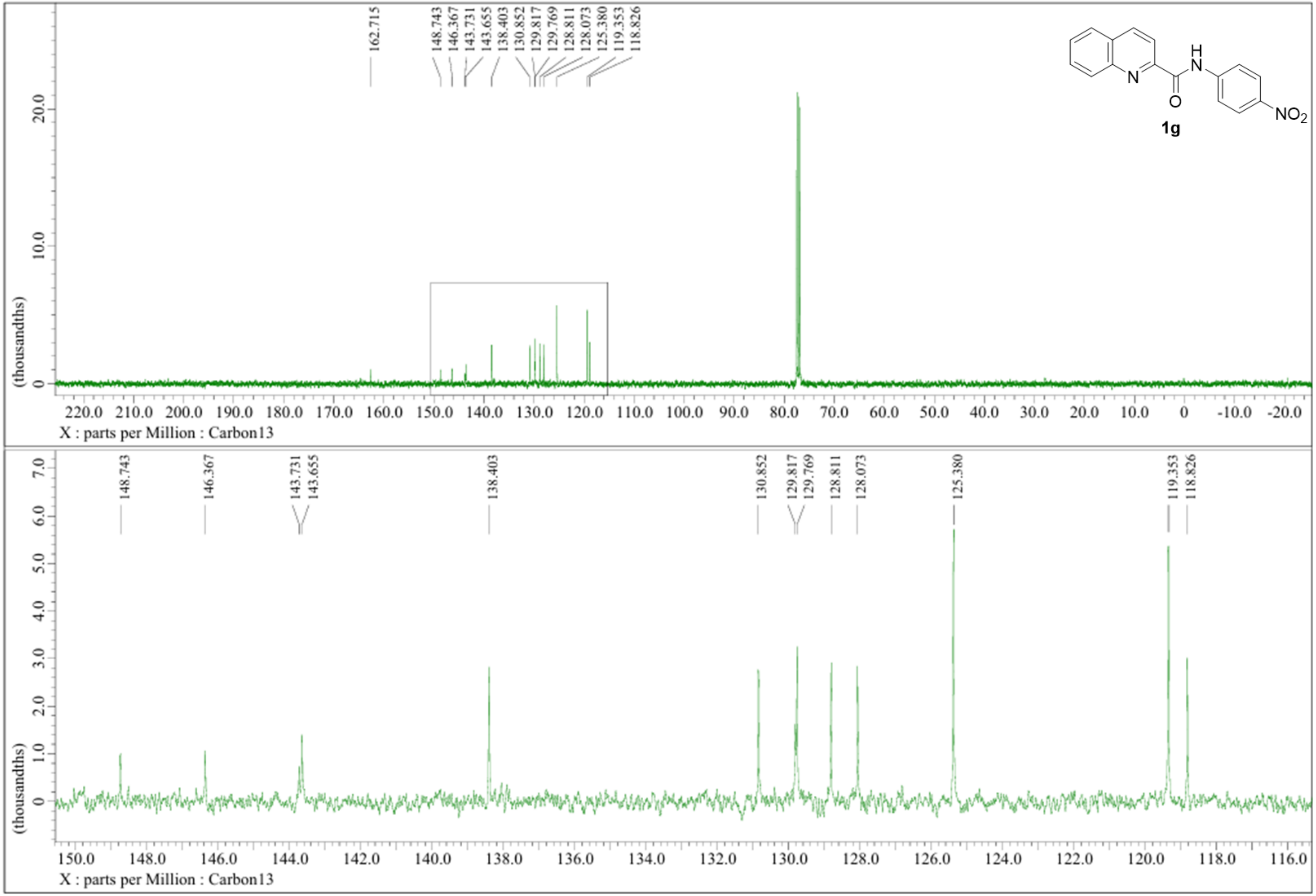

^{1}H NMR (400 MHz, $CDCl_3$)

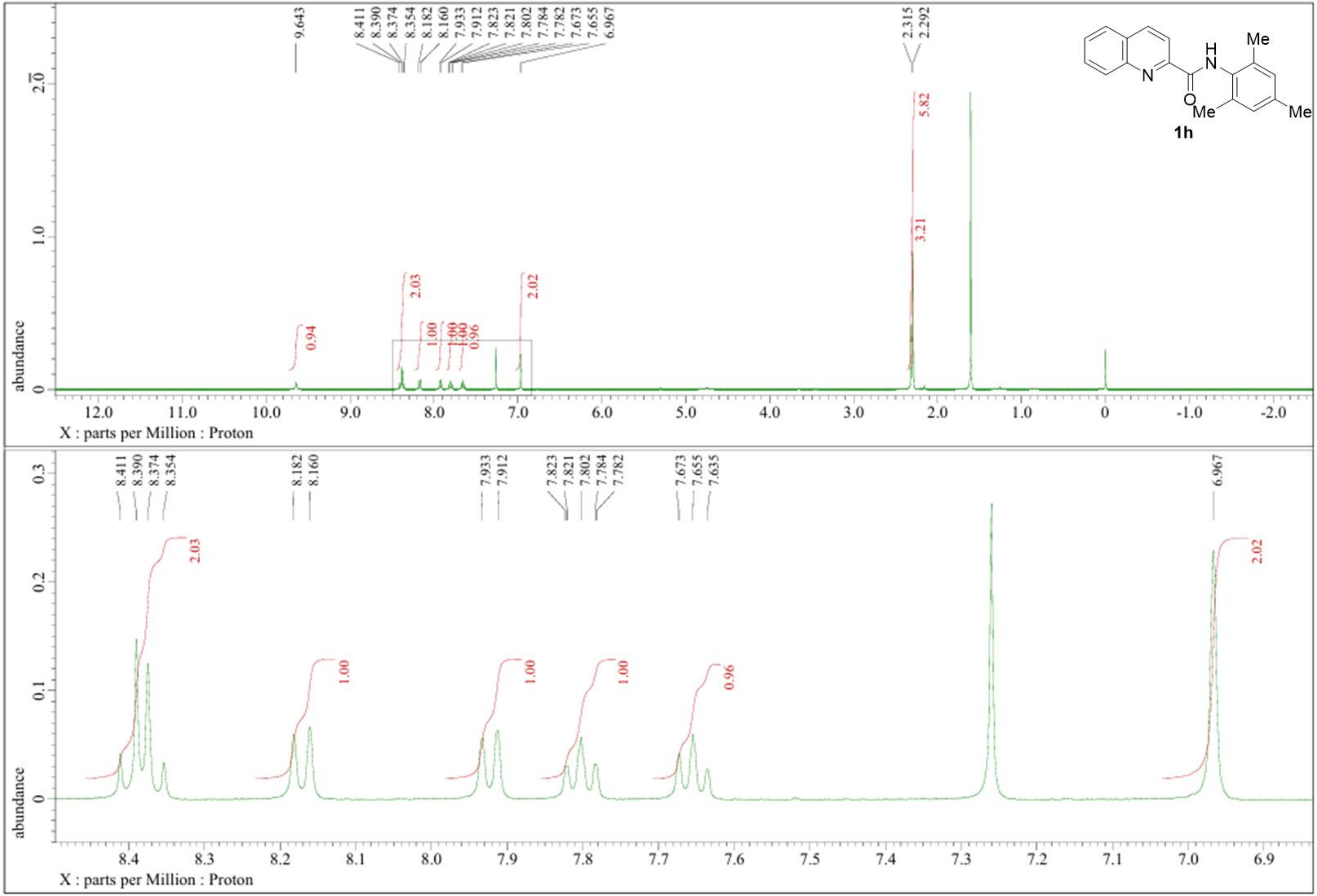

$^{13}C\{^1H\}$ NMR (100 MHz, $CDCl_3$)

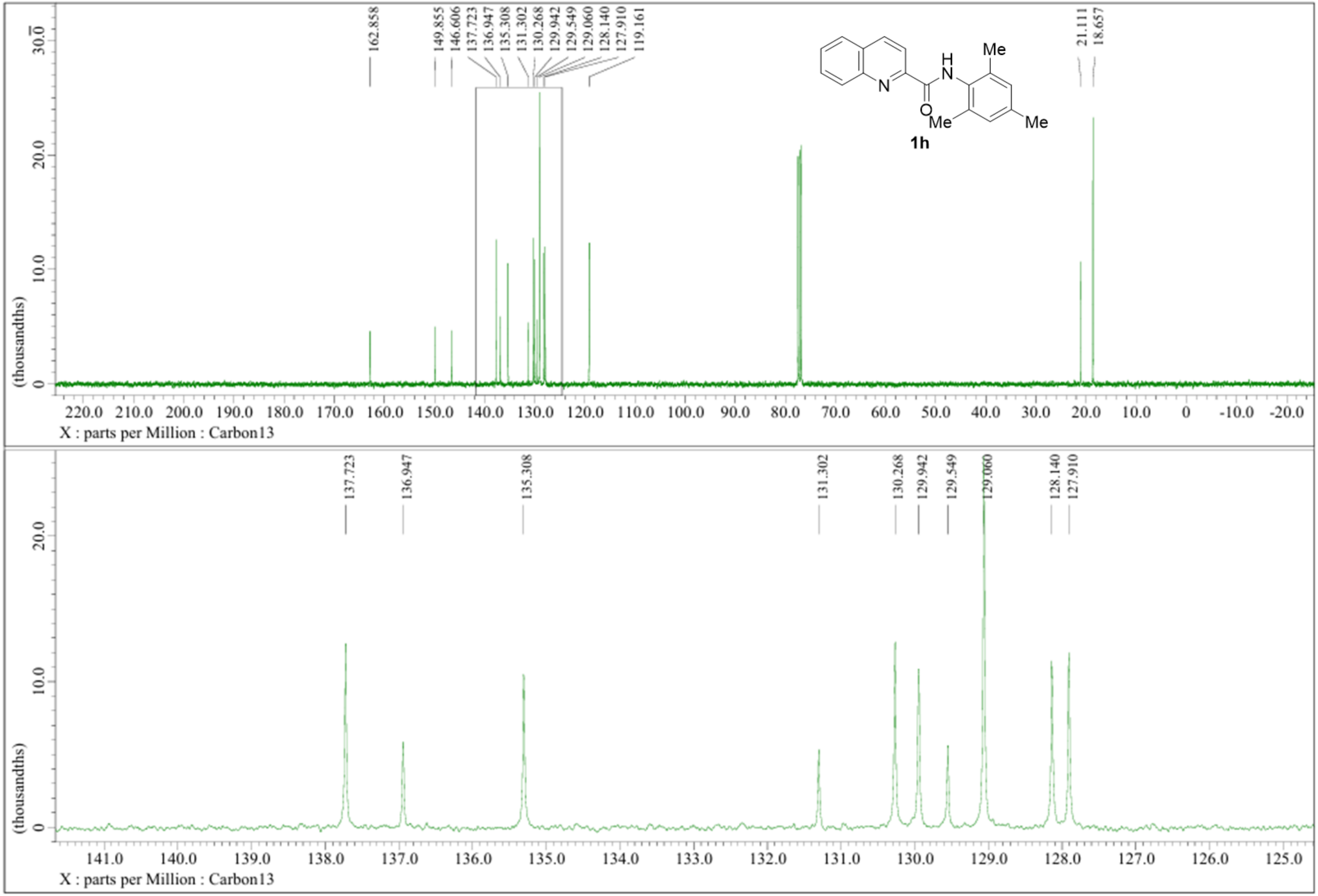

^{1}H NMR (400 MHz, $CDCl_3$)

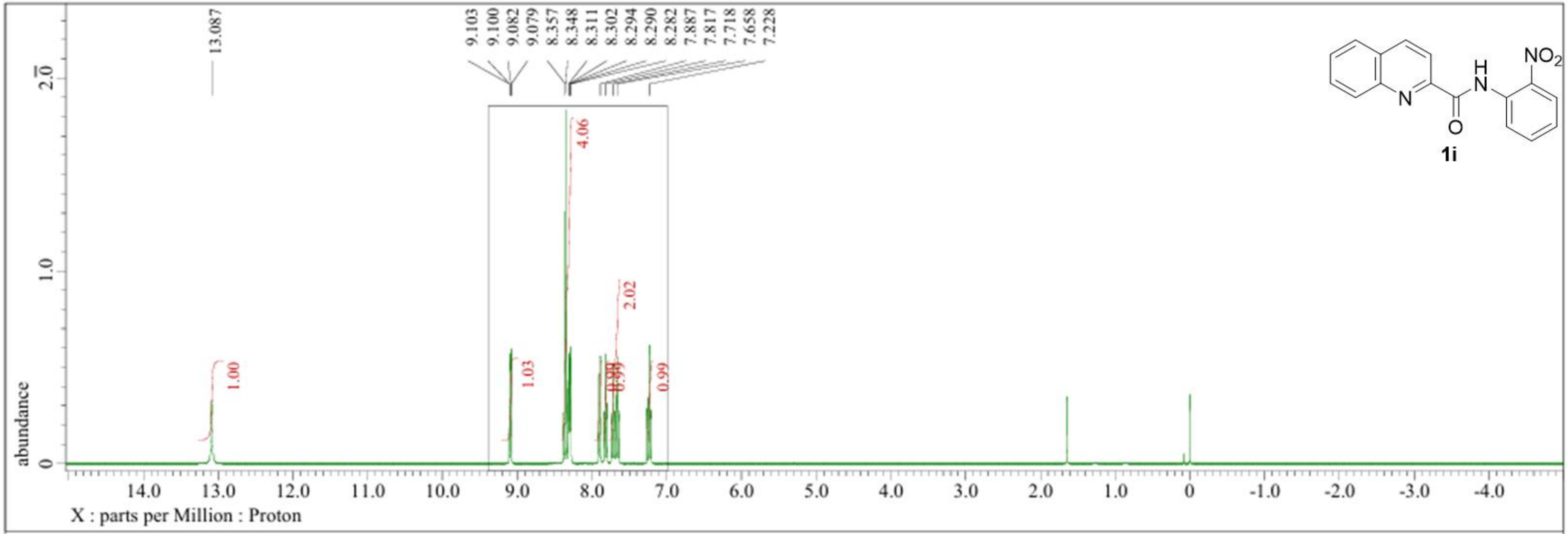
1i

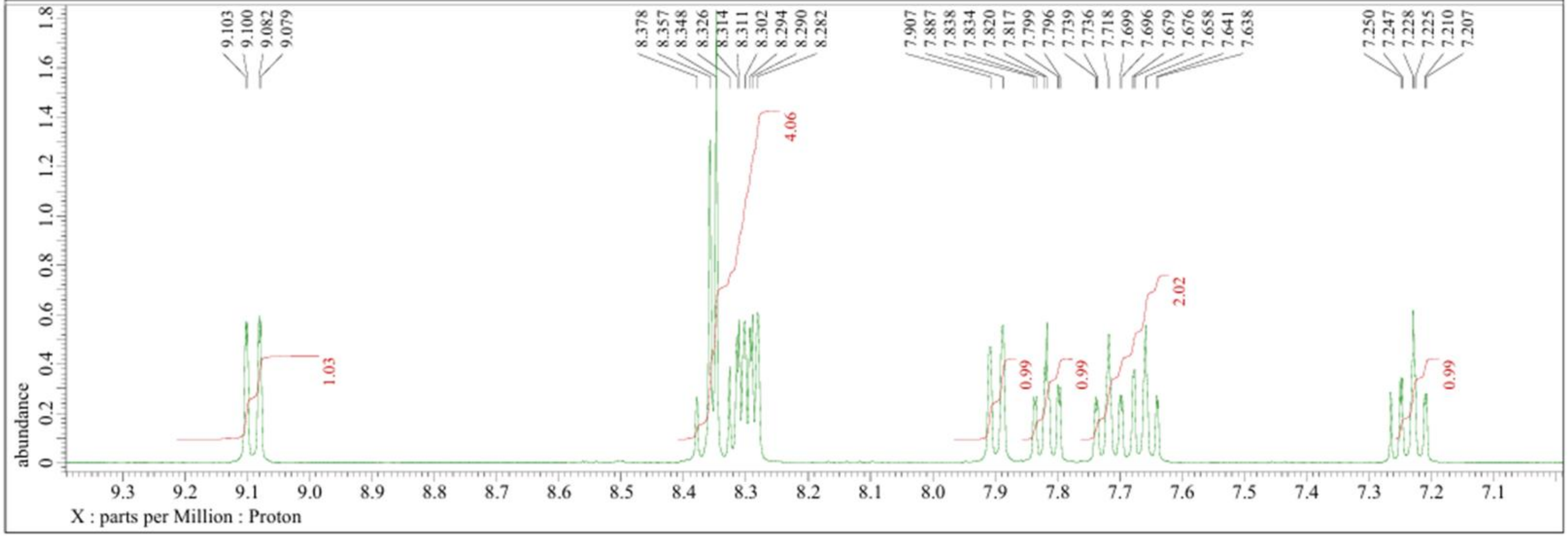

$^{13}C\{^1H\}$ NMR (100 MHz, $CDCl_3$)

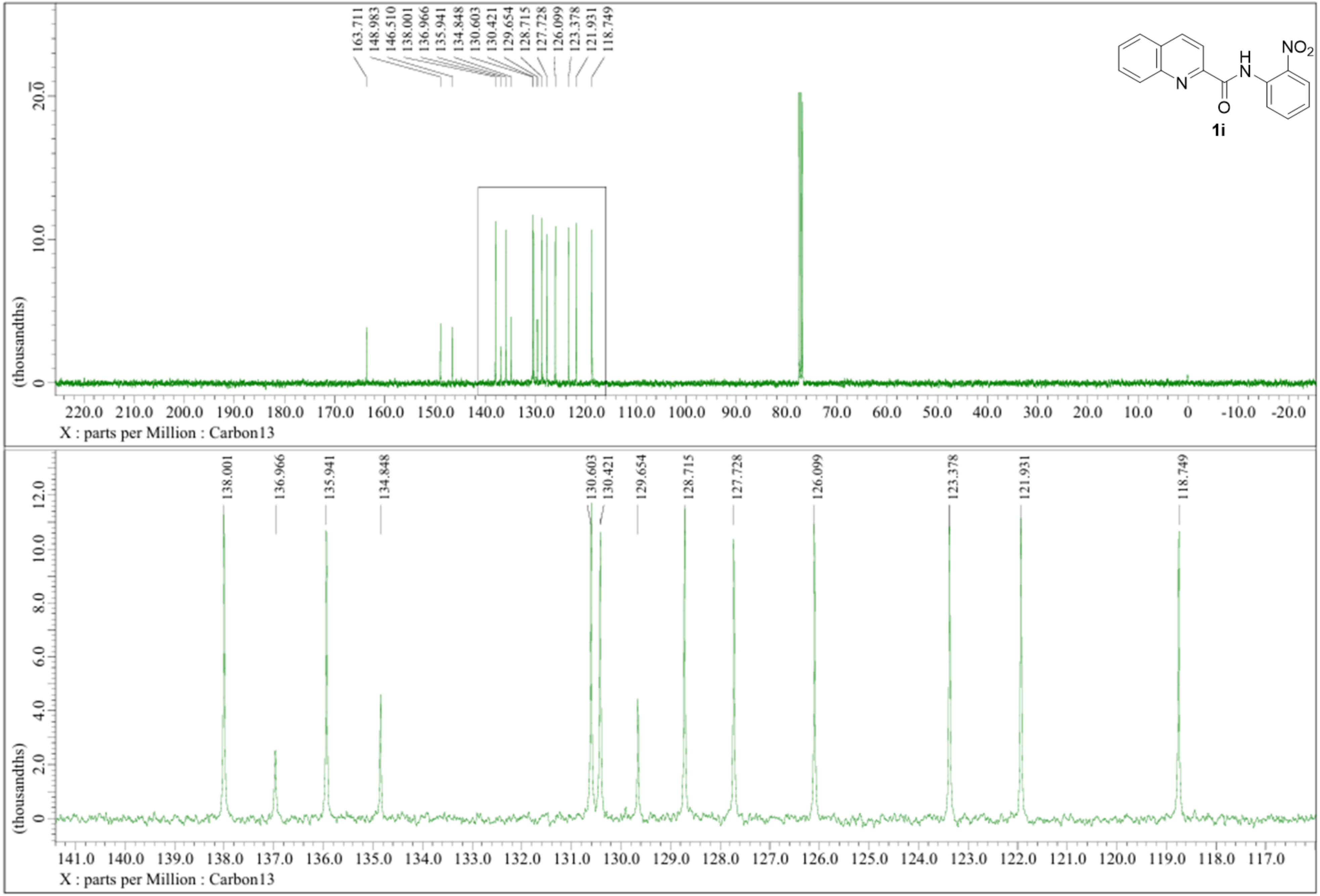

^{1}H NMR (400 MHz, $CDCl_3$)

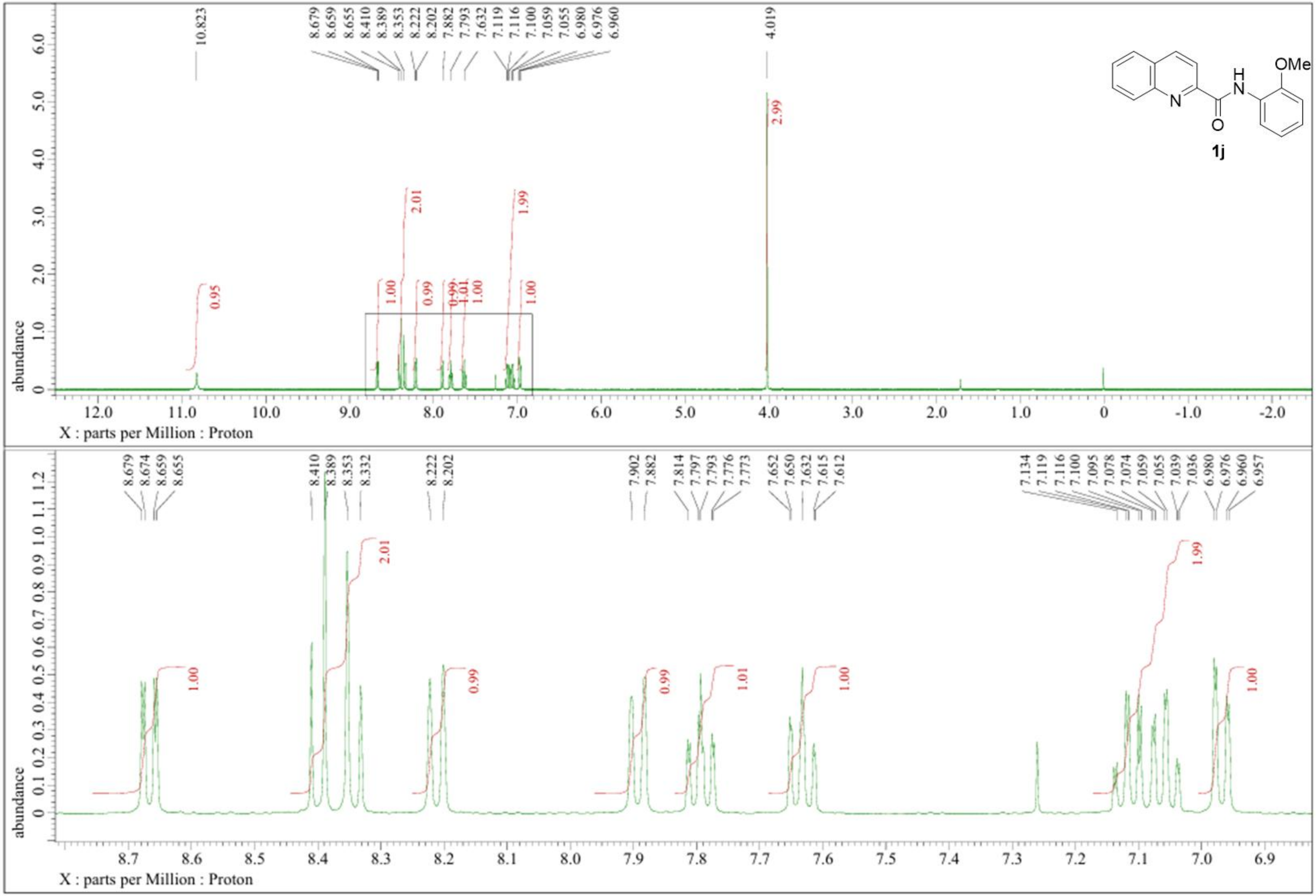

$^{13}C\{^{1}H\}$ NMR (100 MHz, $CDCl_3$)

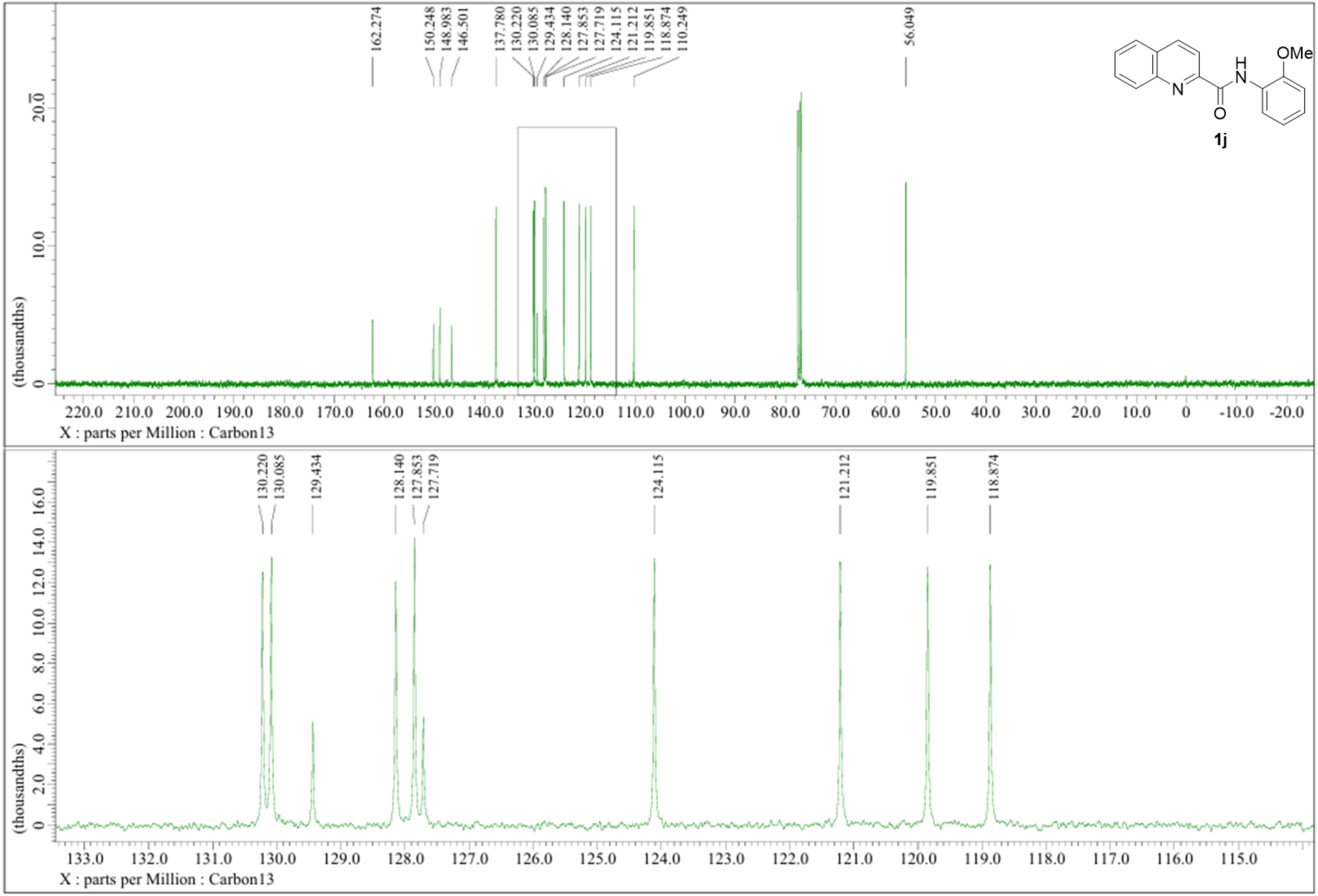

^{1}H NMR (400 MHz, $CDCl_3$)

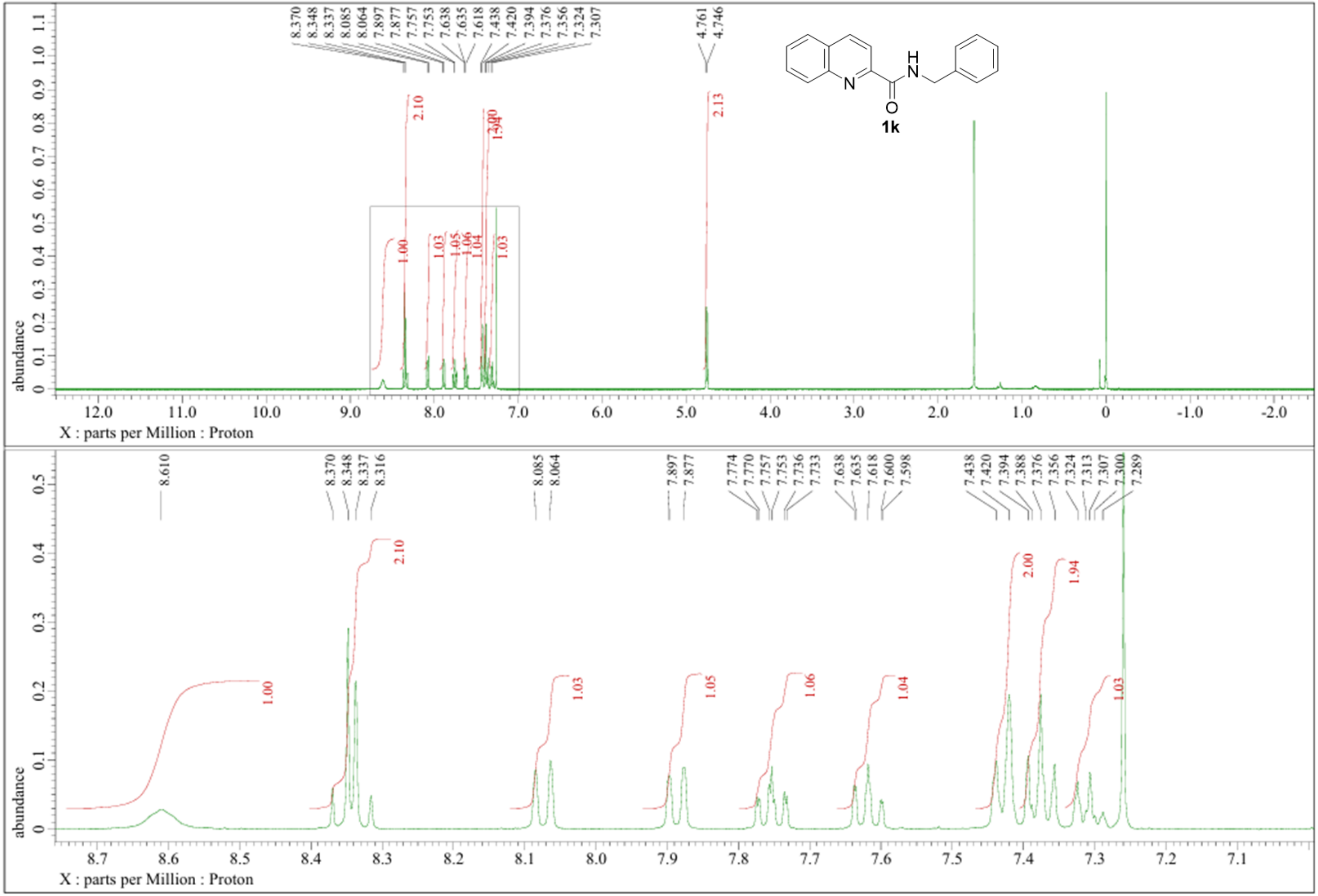

1k

$^{13}C\{^1H\}$ NMR (100 MHz, $CDCl_3$)

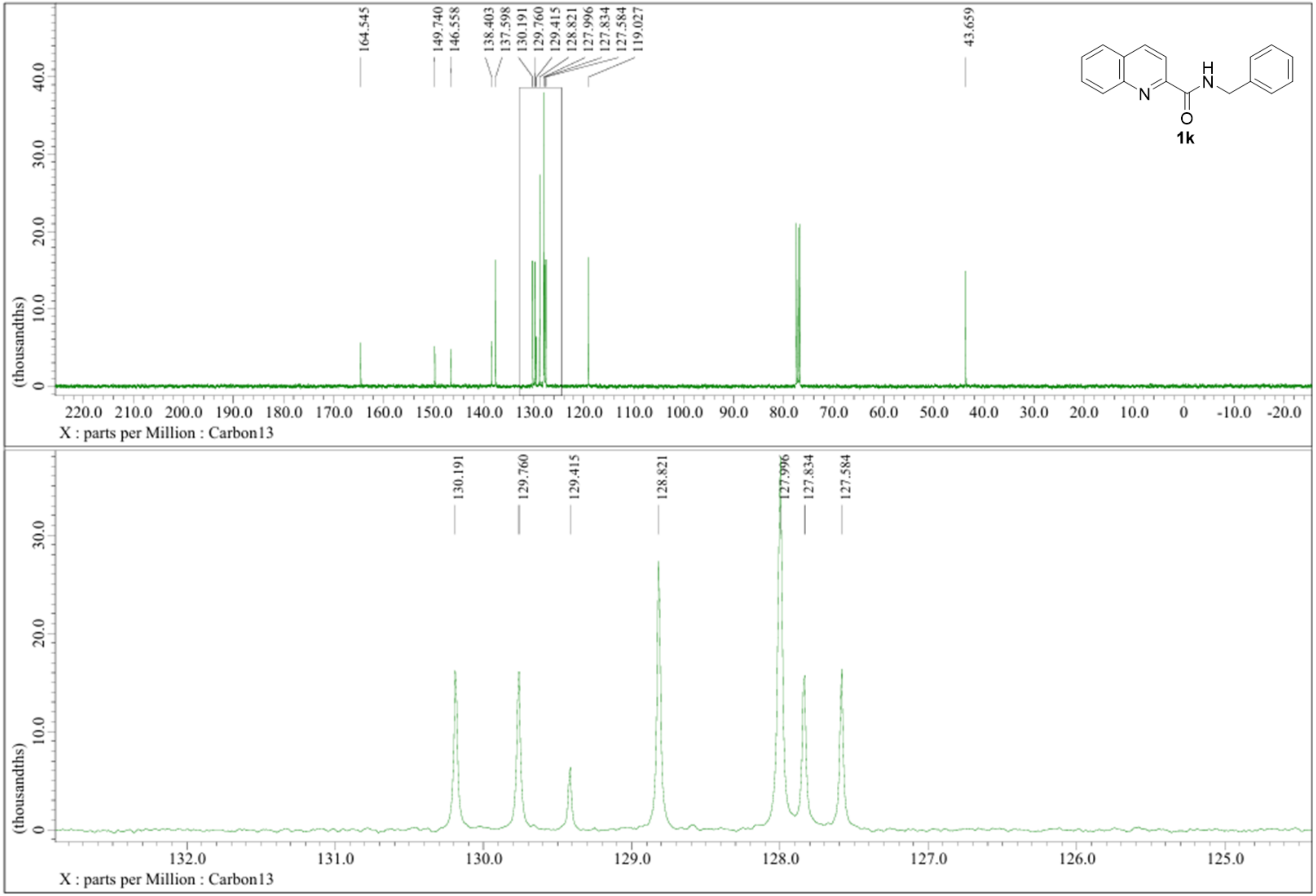

^{1}H NMR (400 MHz, $CDCl_3$)

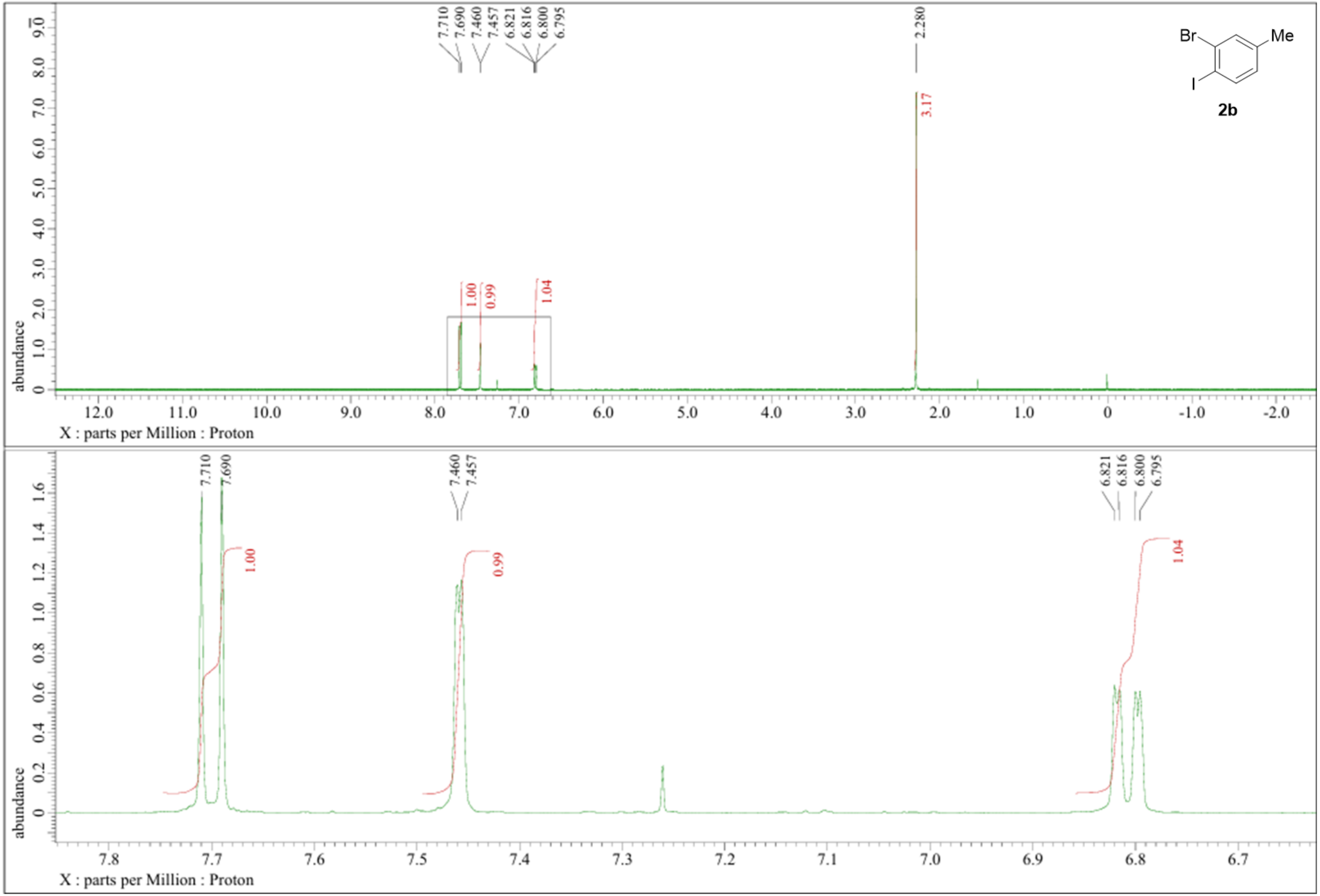

$^{13}C\{^1H\}$ NMR (100 MHz, $CDCl_3$)

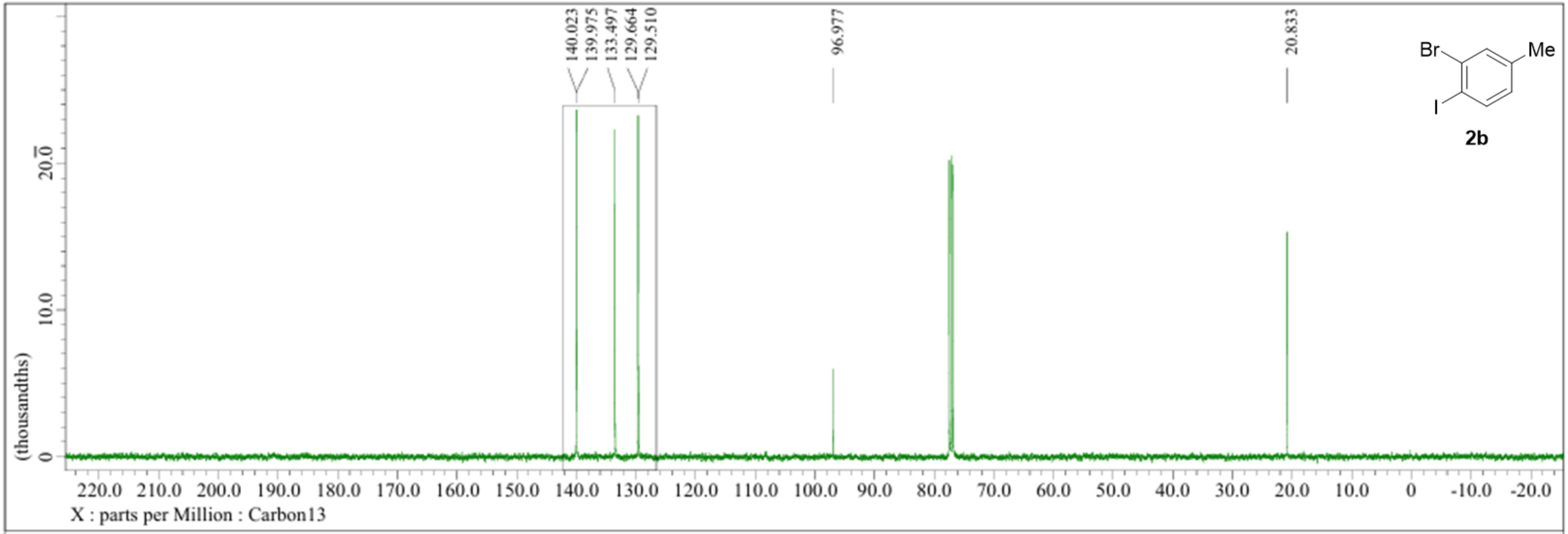

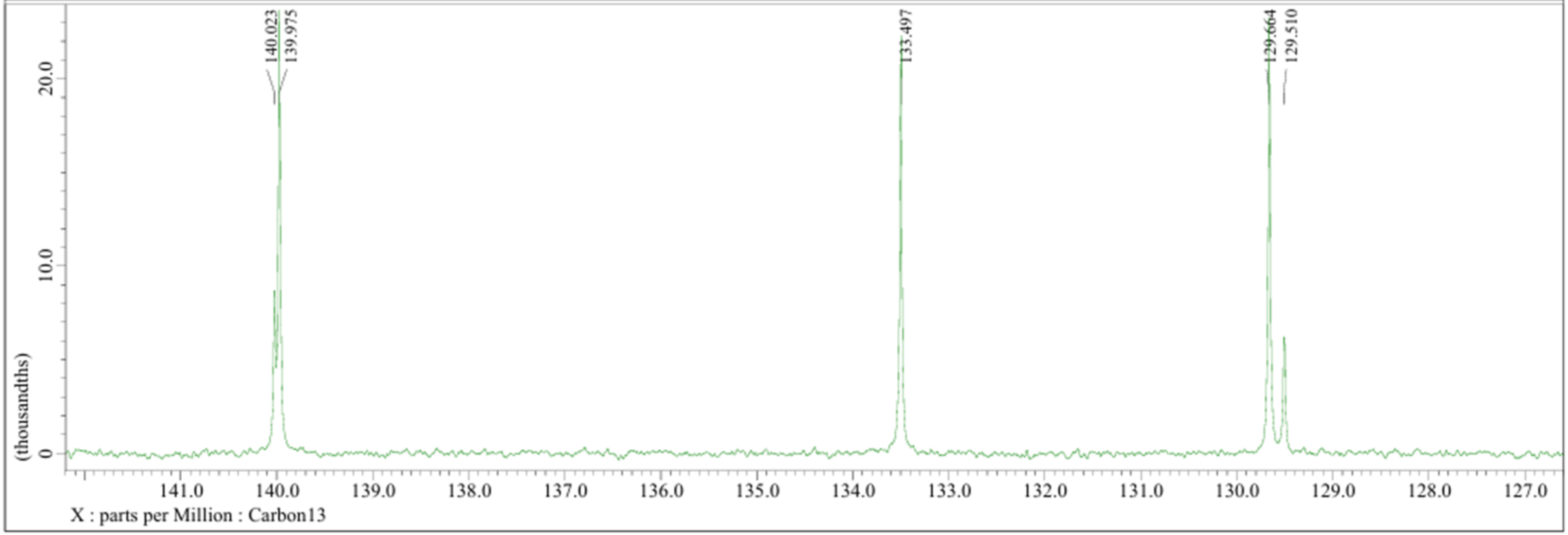

^{1}H NMR (400 MHz, CDCl$_3$)

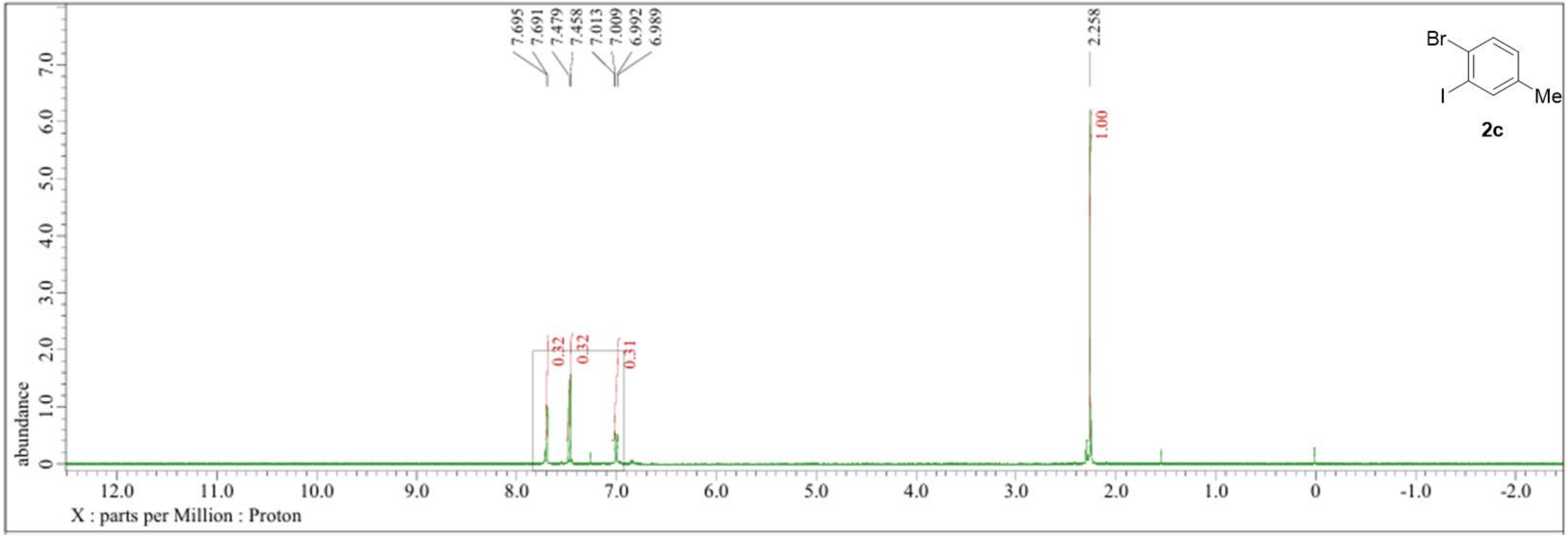

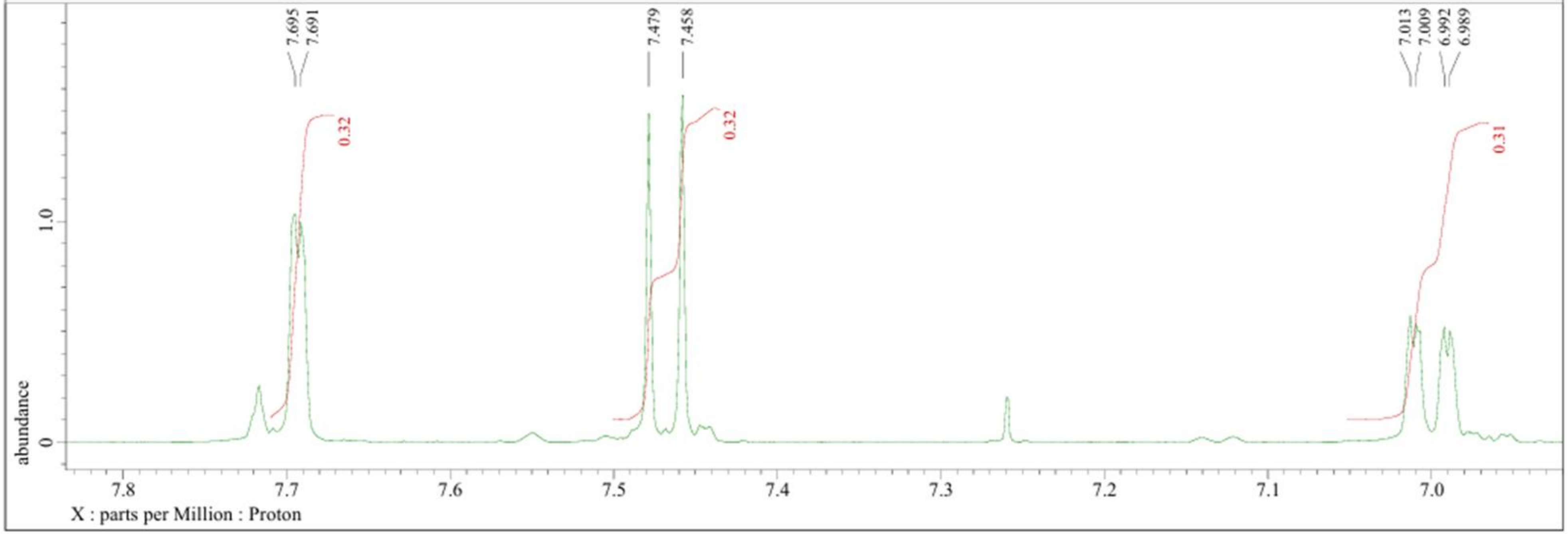

$^{13}C\{^{1}H\}$ NMR (100 MHz, $CDCl_3$)

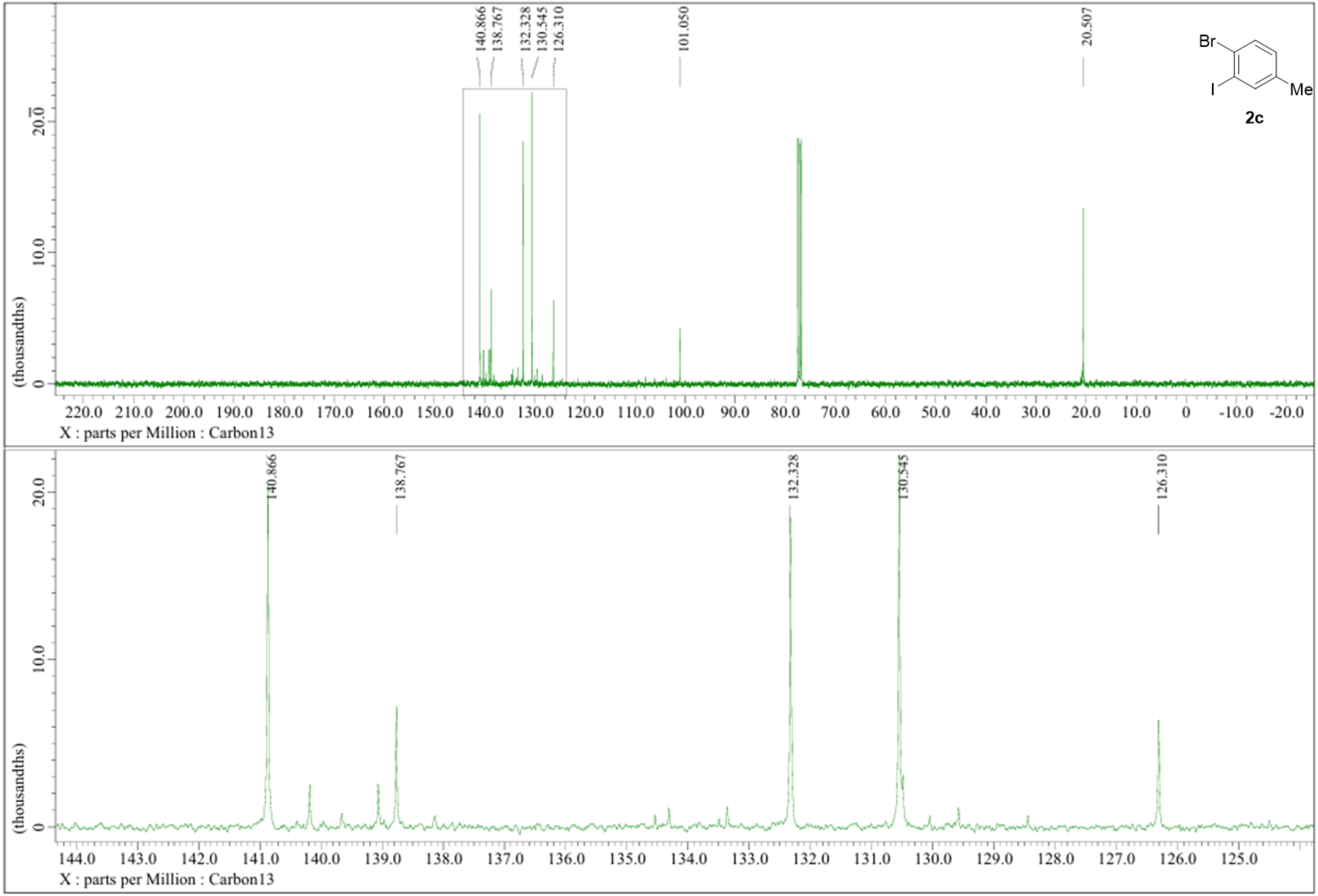

$^{13}C\{^{1}H\}$ NMR (100 MHz, $CDCl_3$)

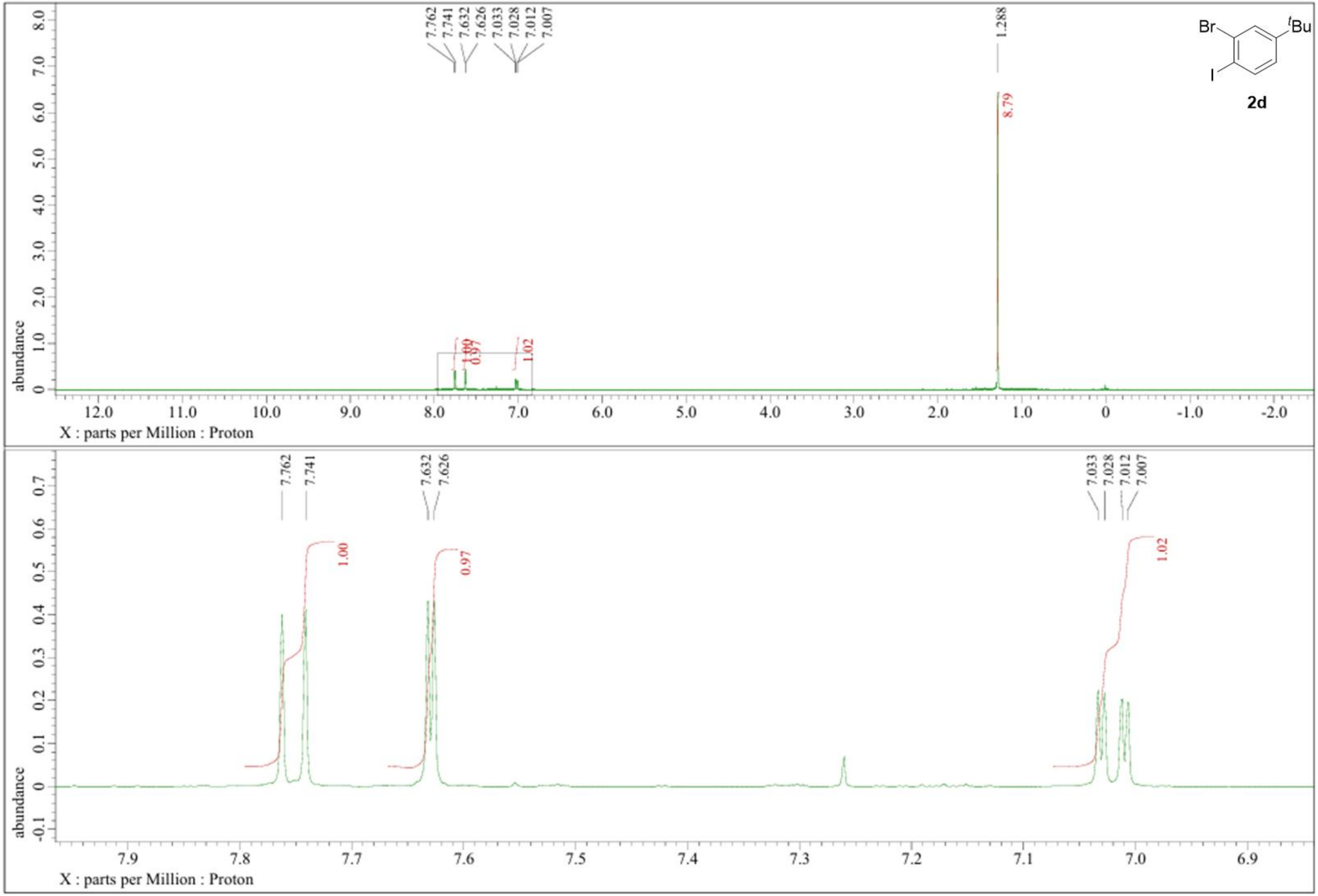

$^{13}C\{^1H\}$ NMR (100 MHz, $CDCl_3$)

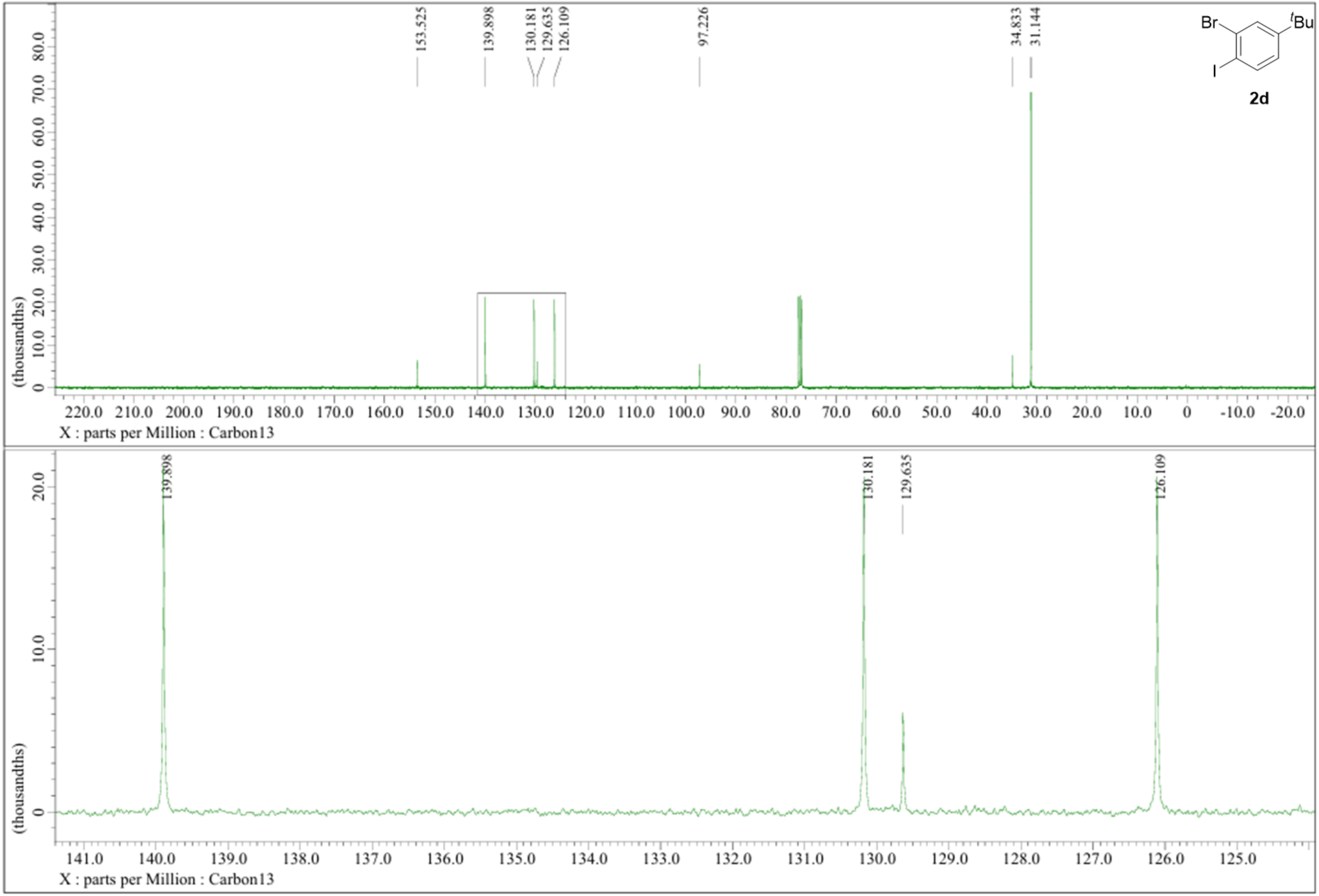

^{1}H NMR (400 MHz, $CDCl_3$)

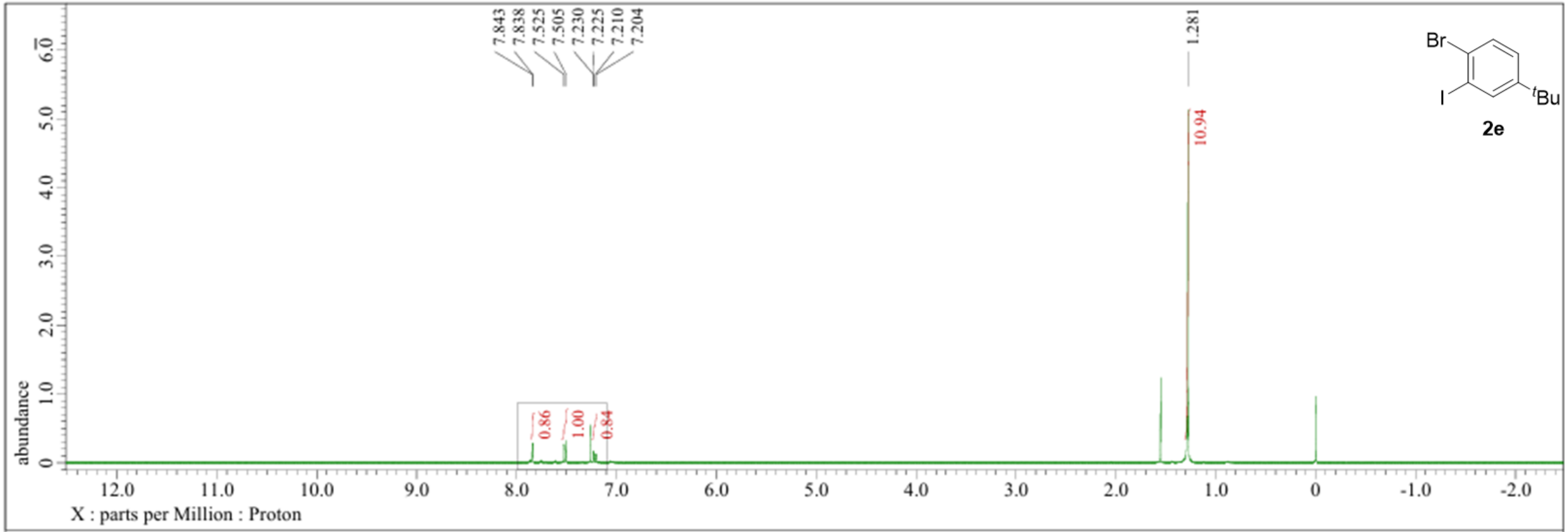

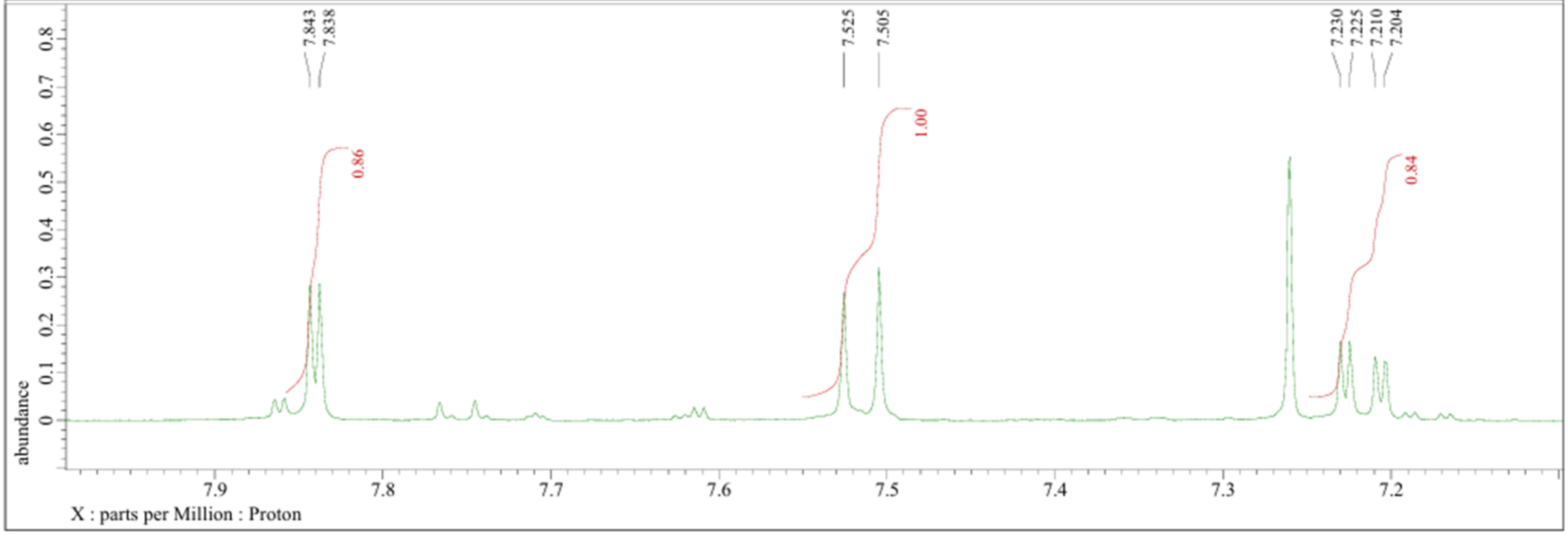

$^{13}C\{^1H\}$ NMR (100 MHz, $CDCl_3$)

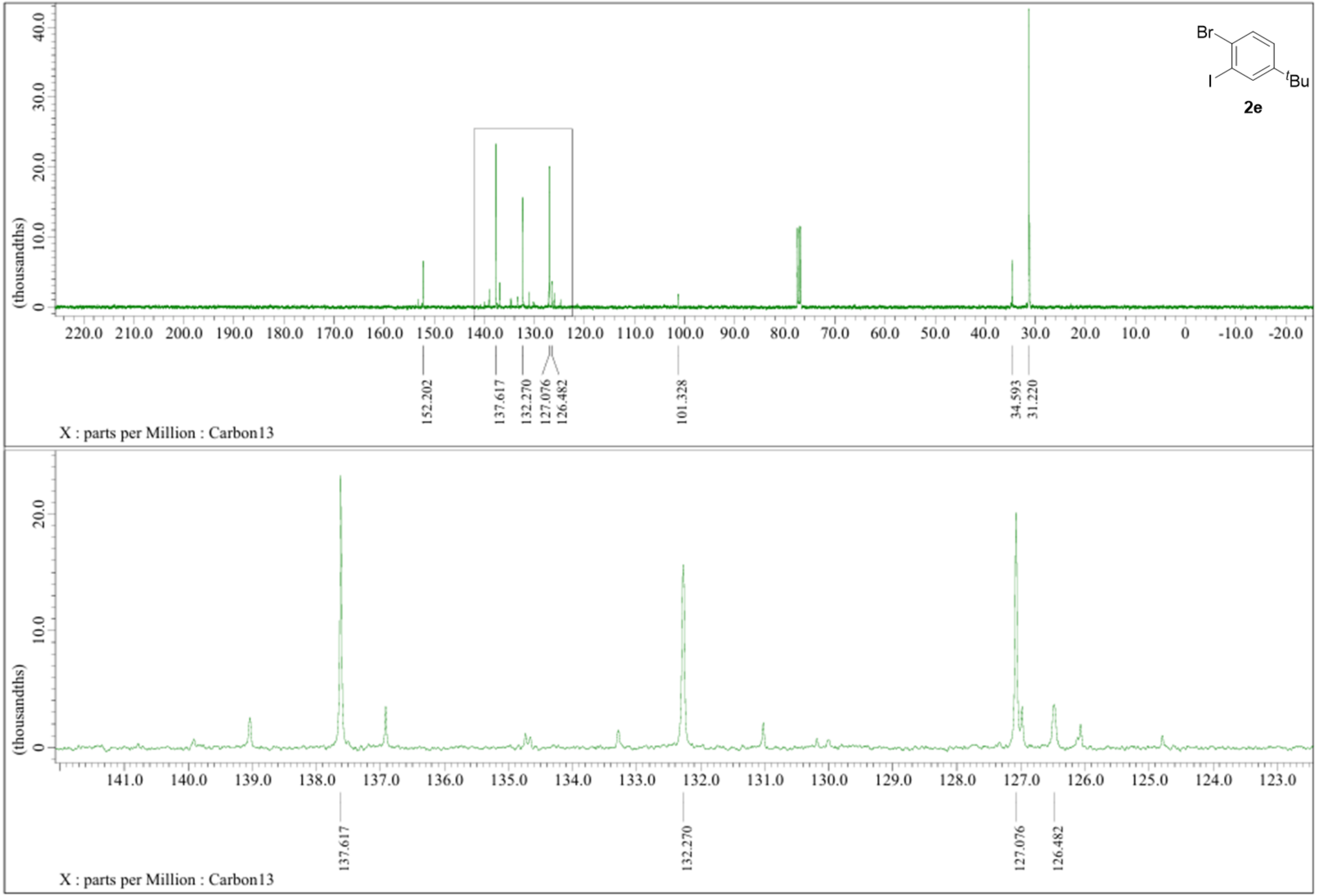

^{1}H NMR (400 MHz, $CDCl_3$)

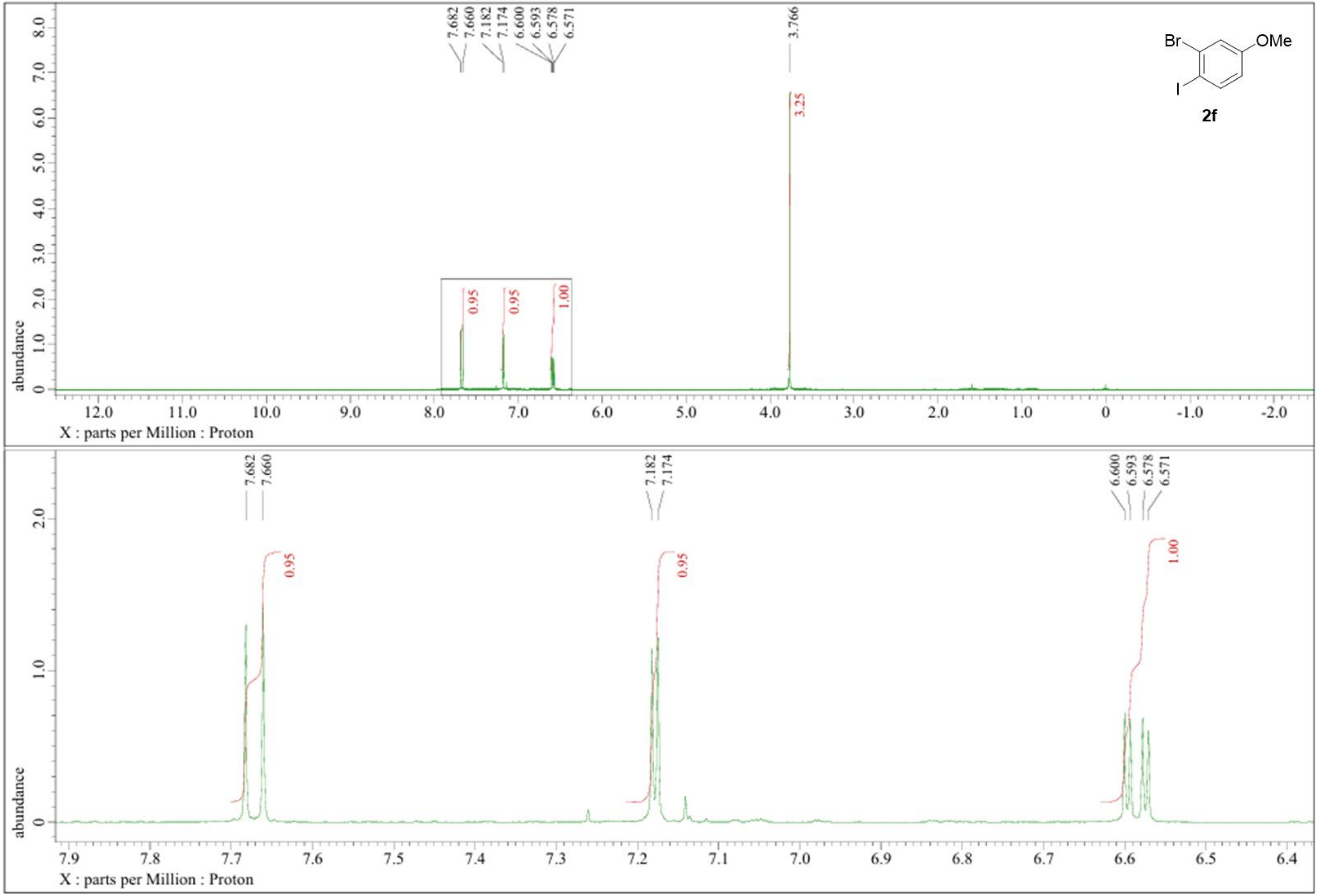

$^{13}C\{^1H\}$ NMR (100 MHz, $CDCl_3$)

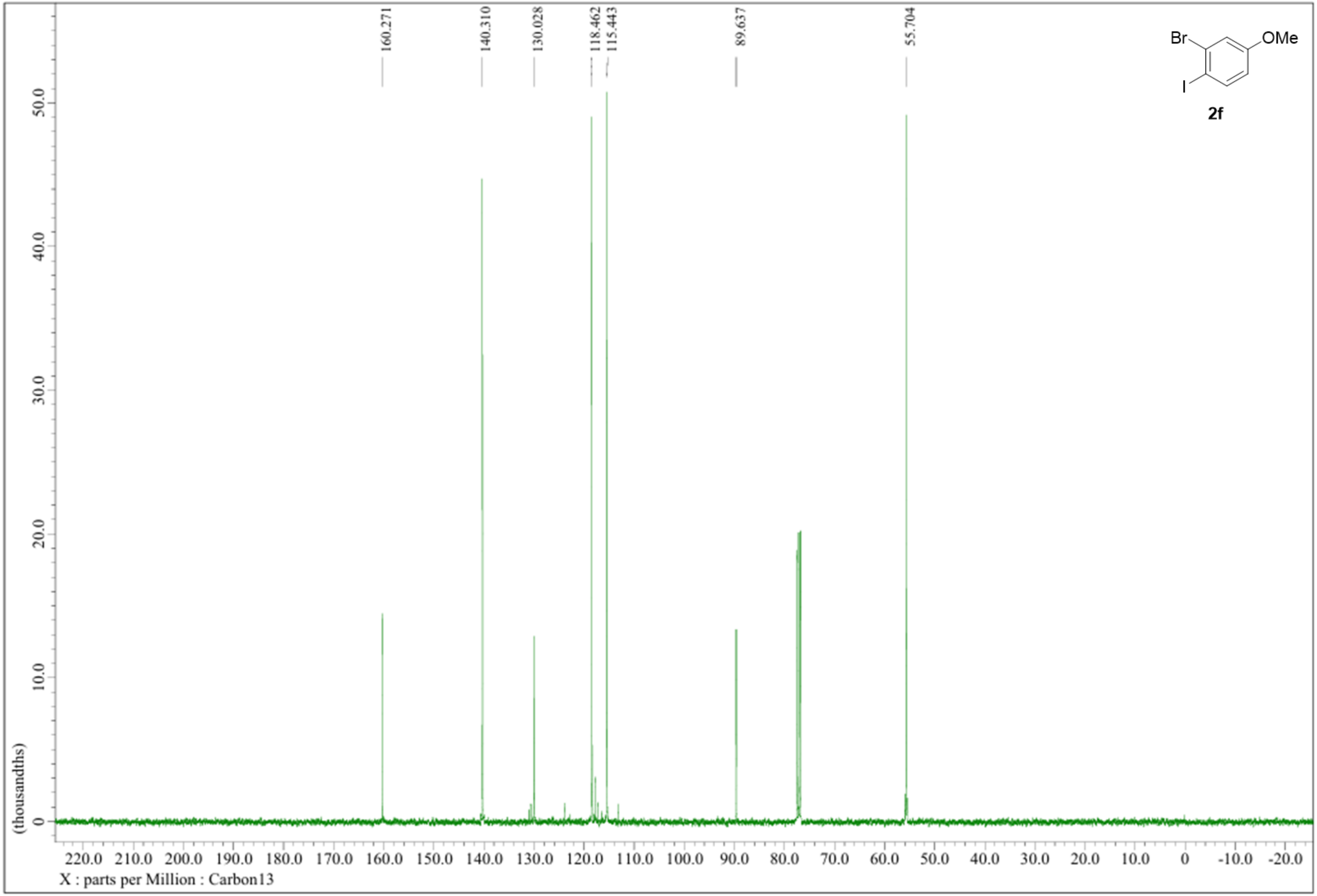

^{1}H NMR (400 MHz, $CDCl_3$)

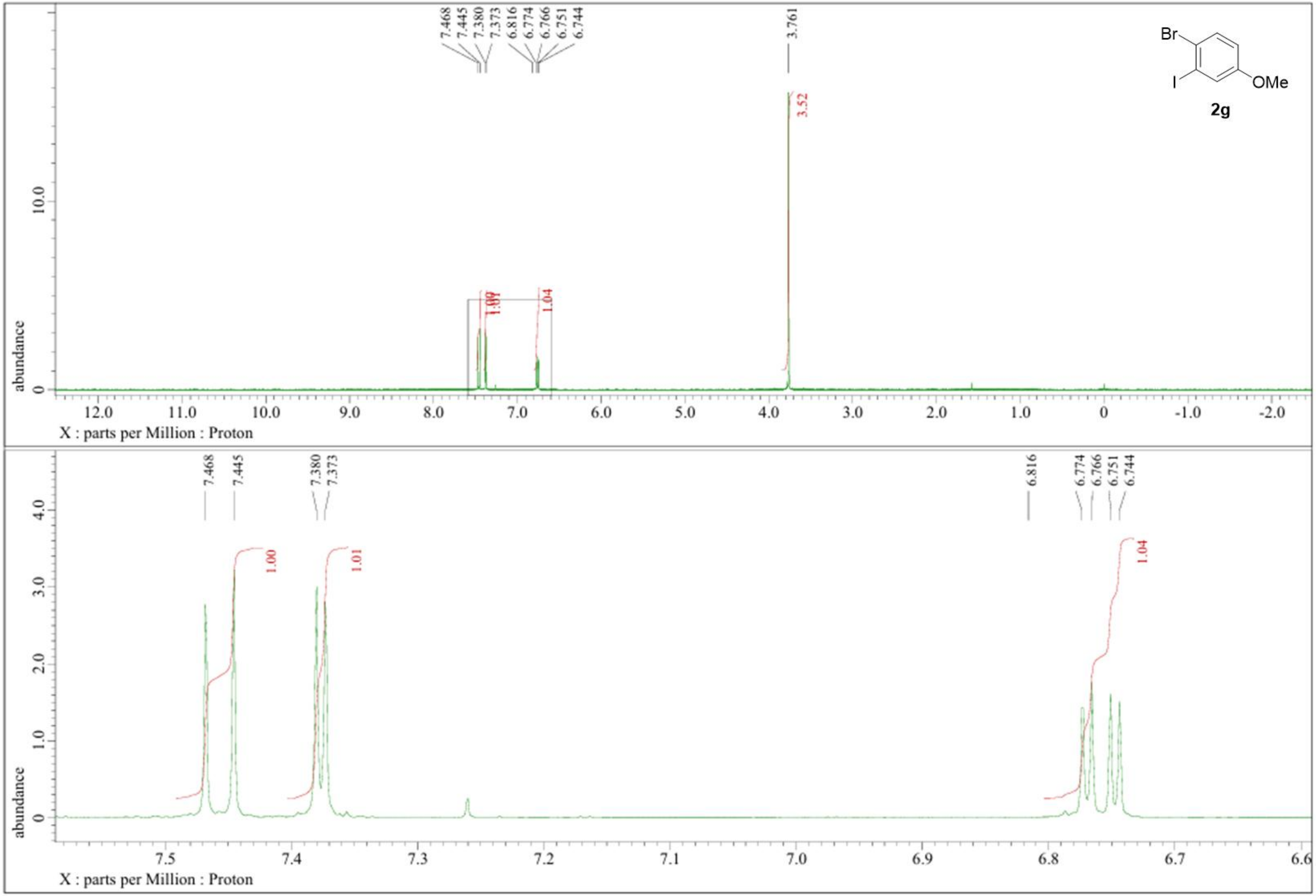

$^{13}C\{^1H\}$ NMR (100 MHz, $CDCl_3$)

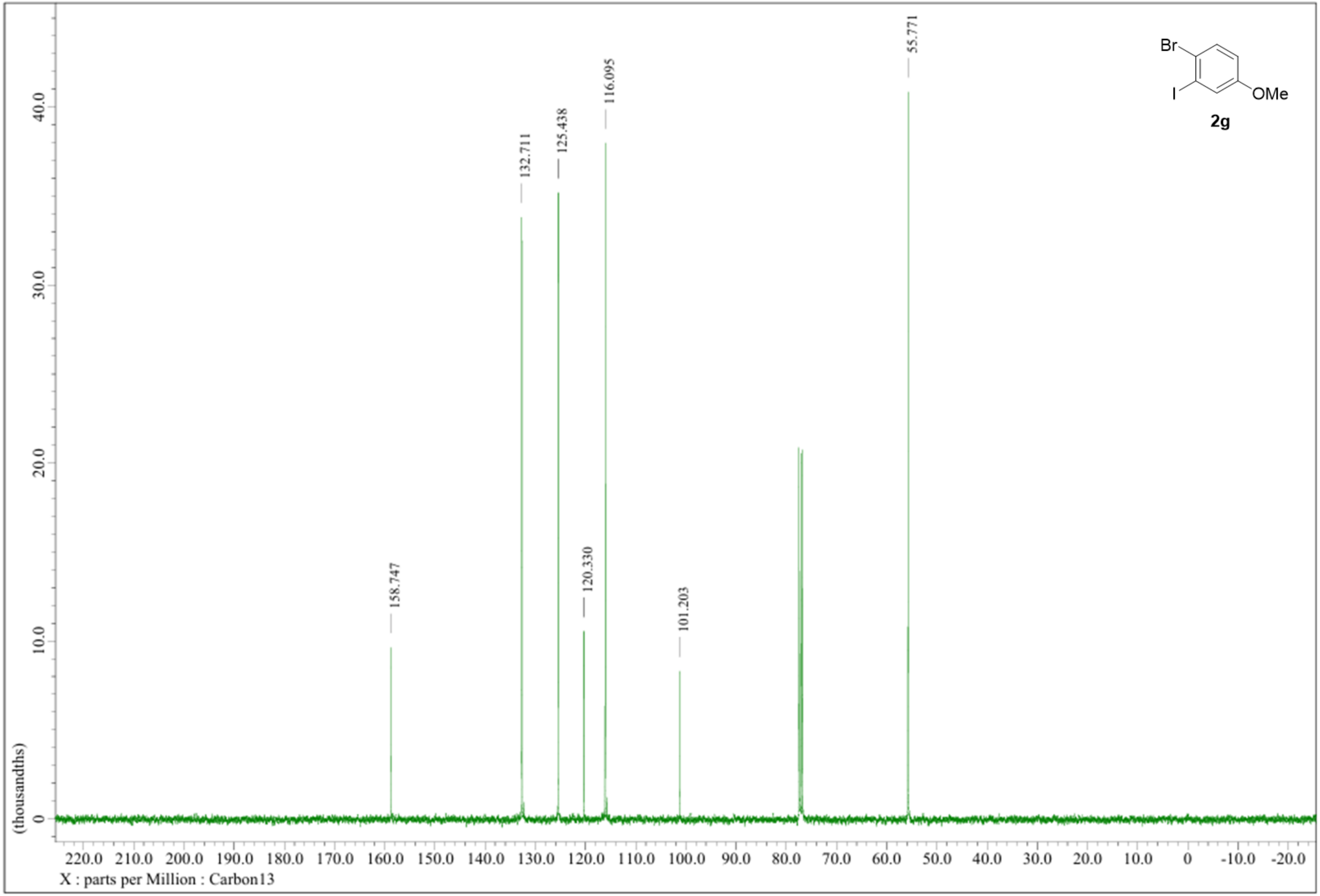

^{1}H NMR (400 MHz, $CDCl_3$)

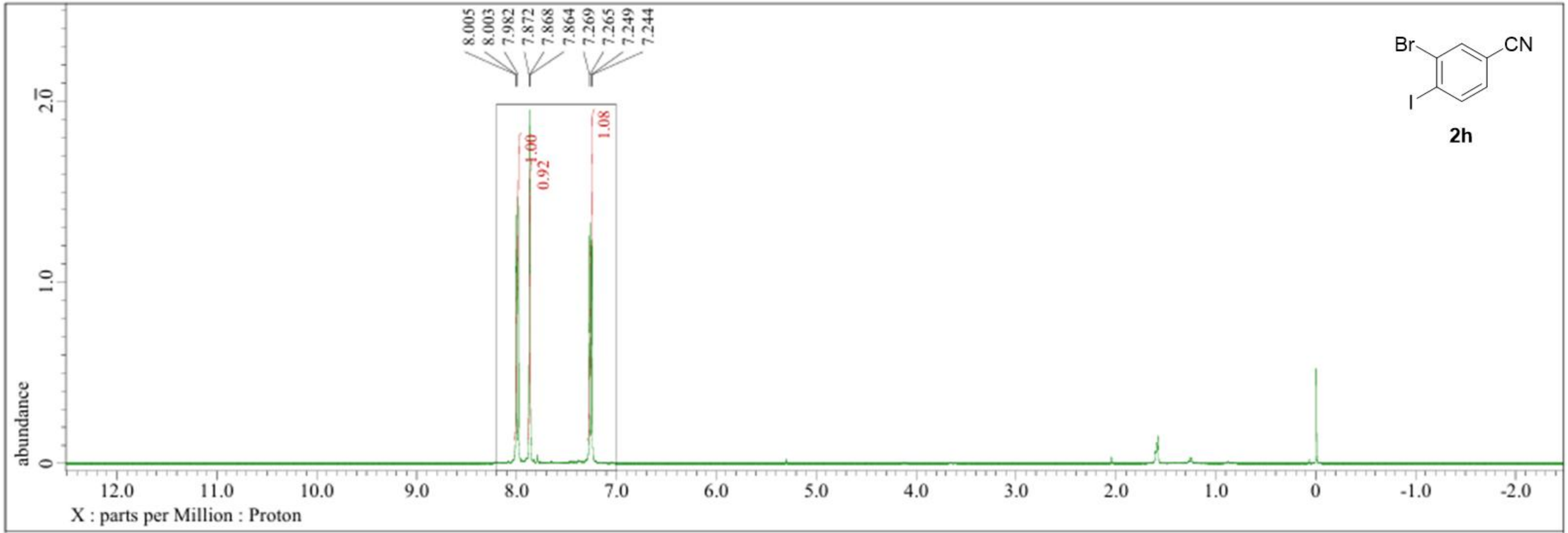

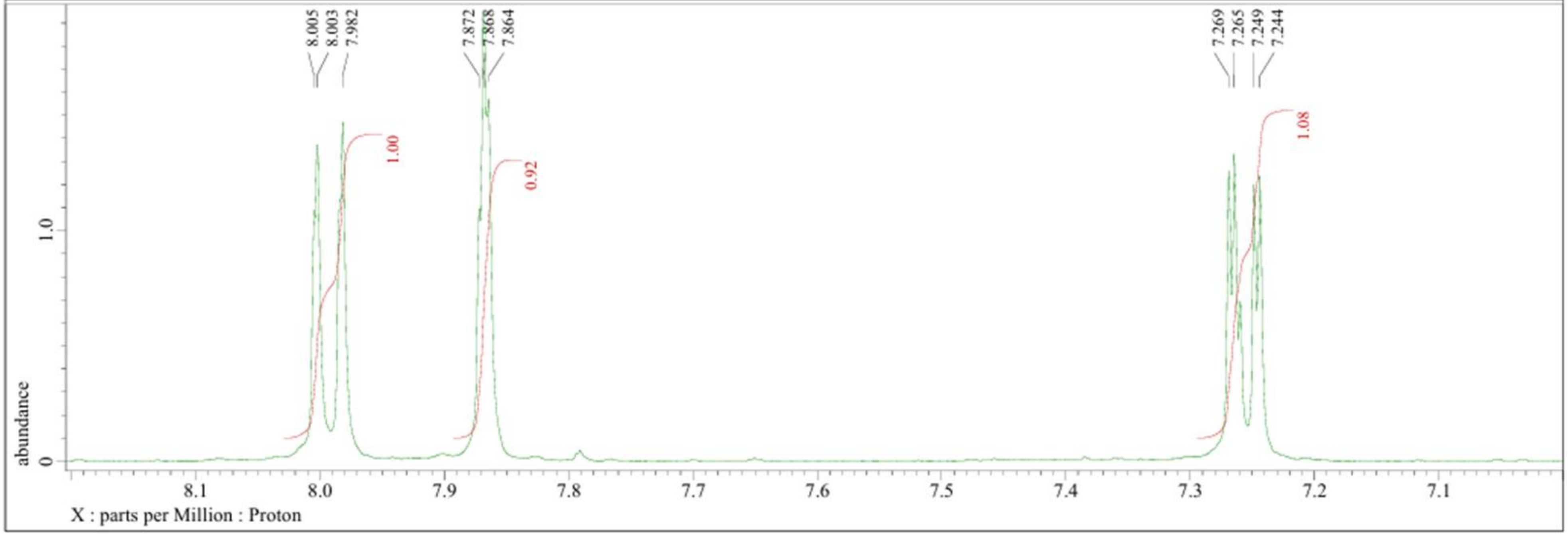

$^{13}C\{^1H\}$ NMR (100 MHz, $CDCl_3$)

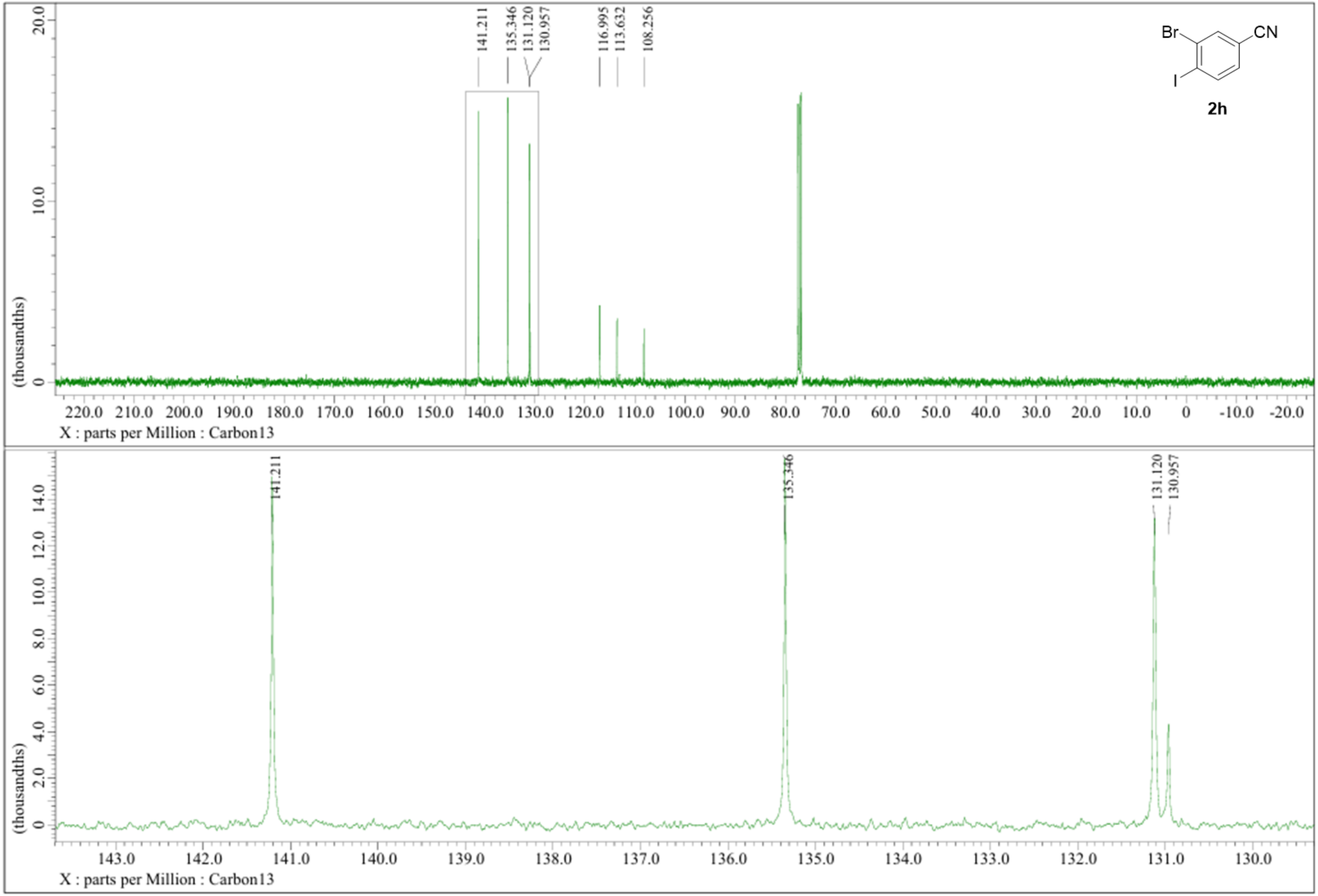

^{1}H NMR (400 MHz, $CDCl_3$)

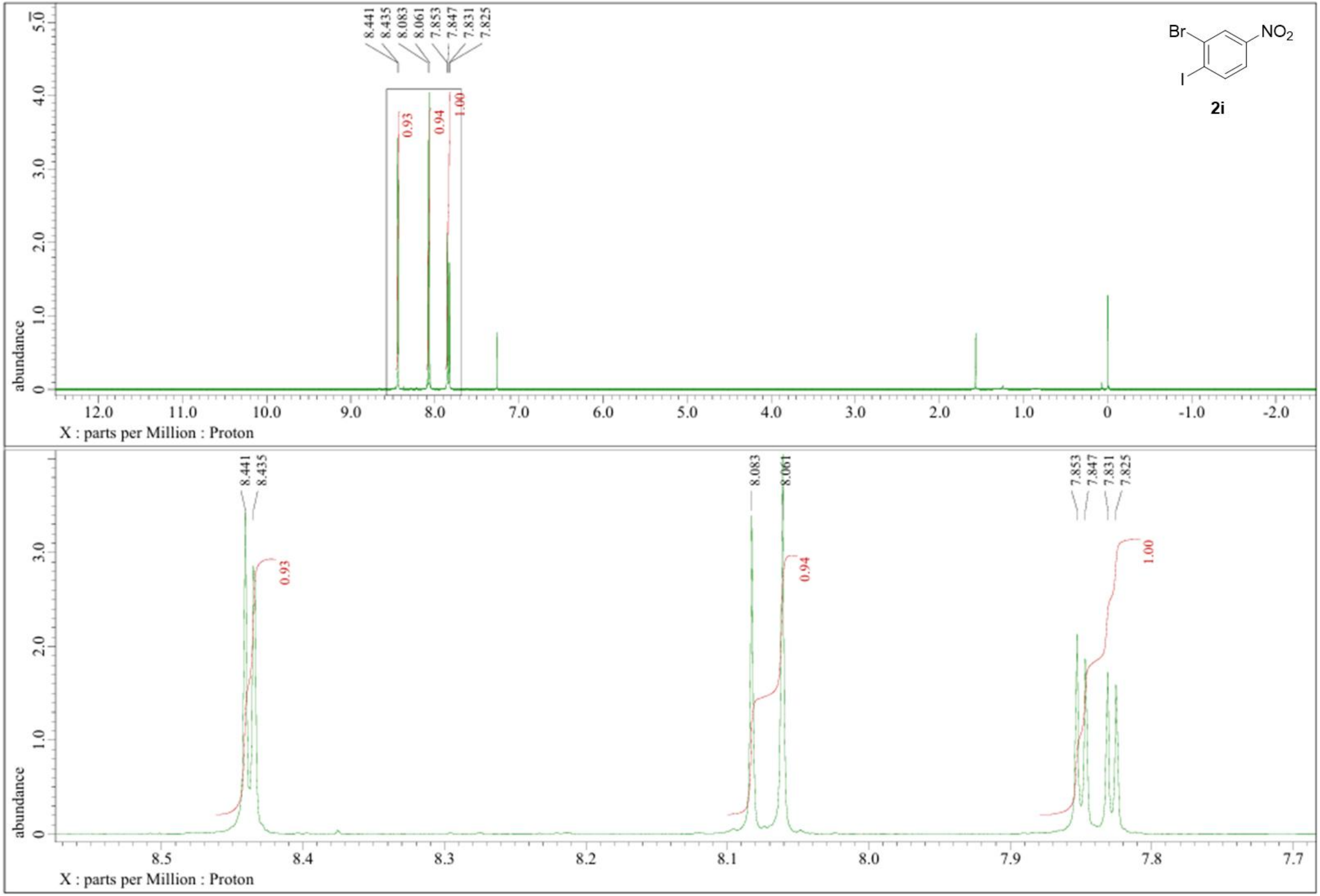

$^{13}C\{^{1}H\}$ NMR (100 MHz, $CDCl_3$)

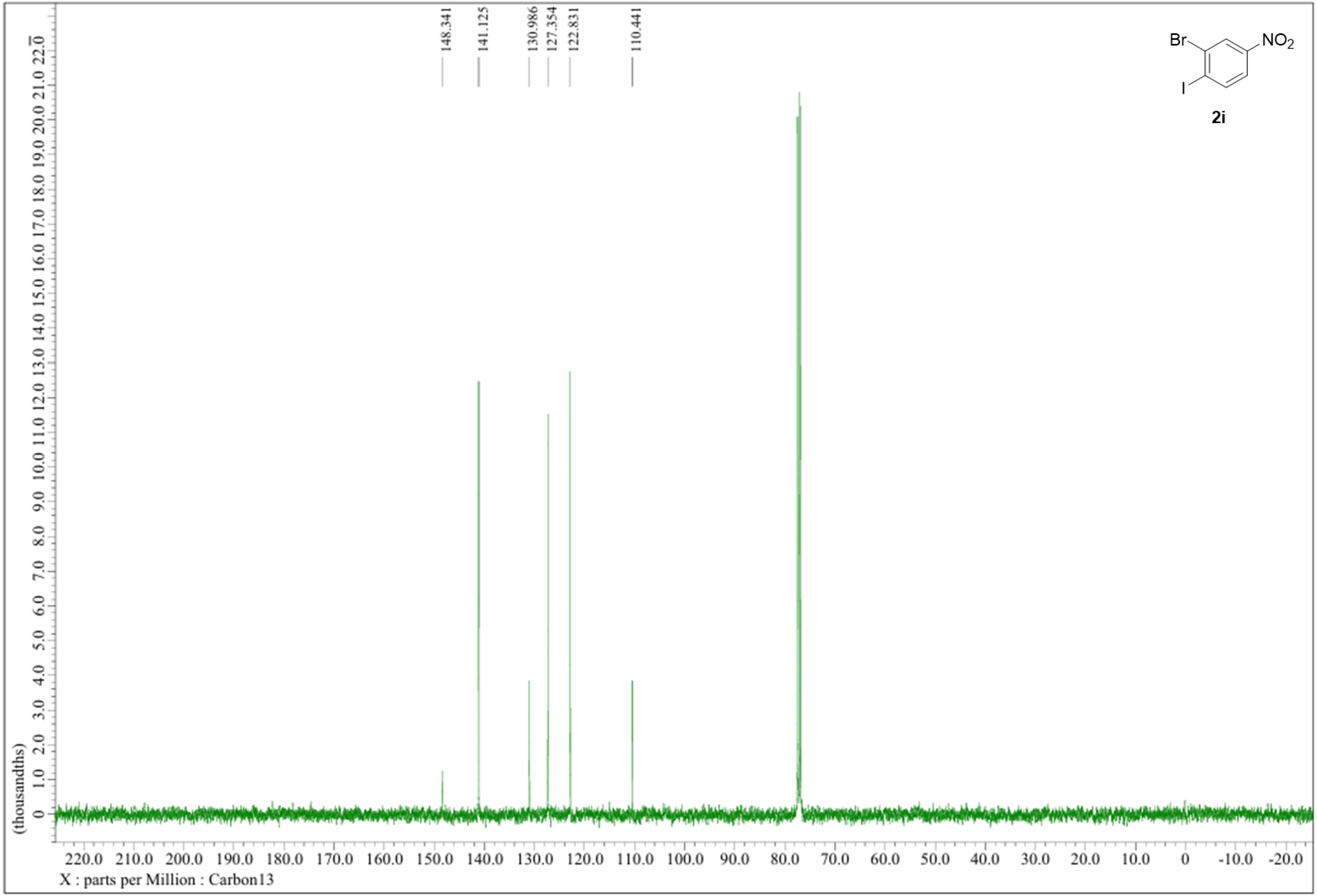

^{1}H NMR (400 MHz, $CDCl_3$)

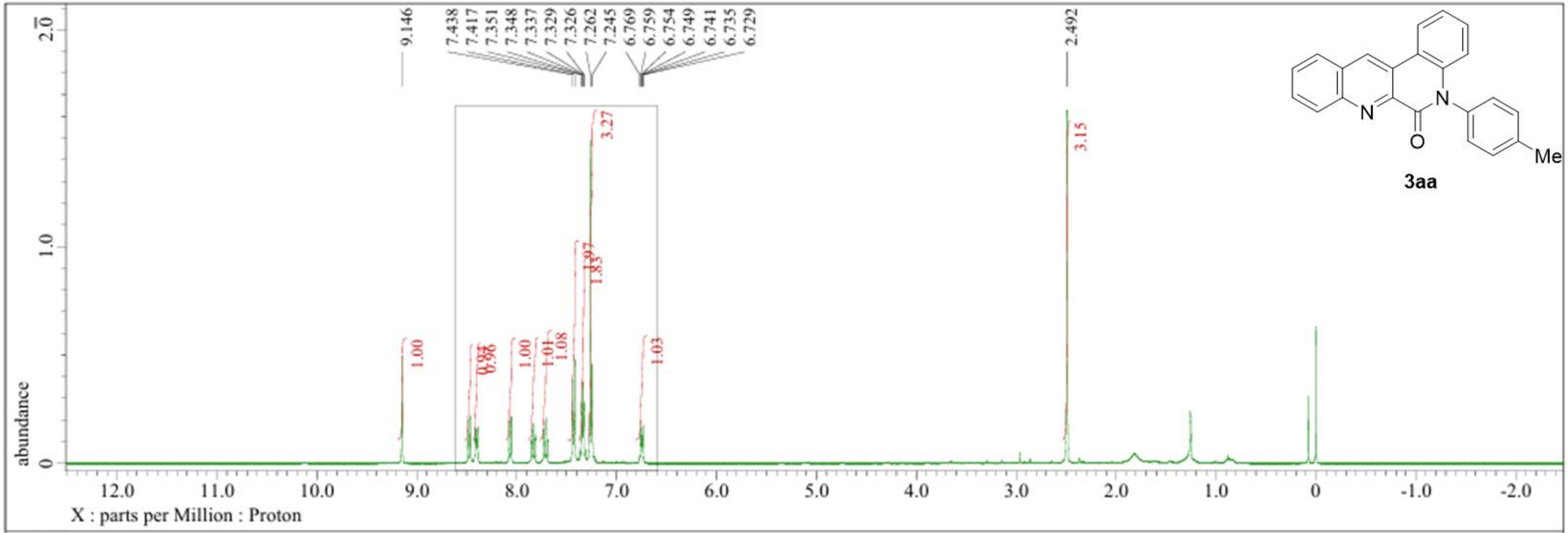

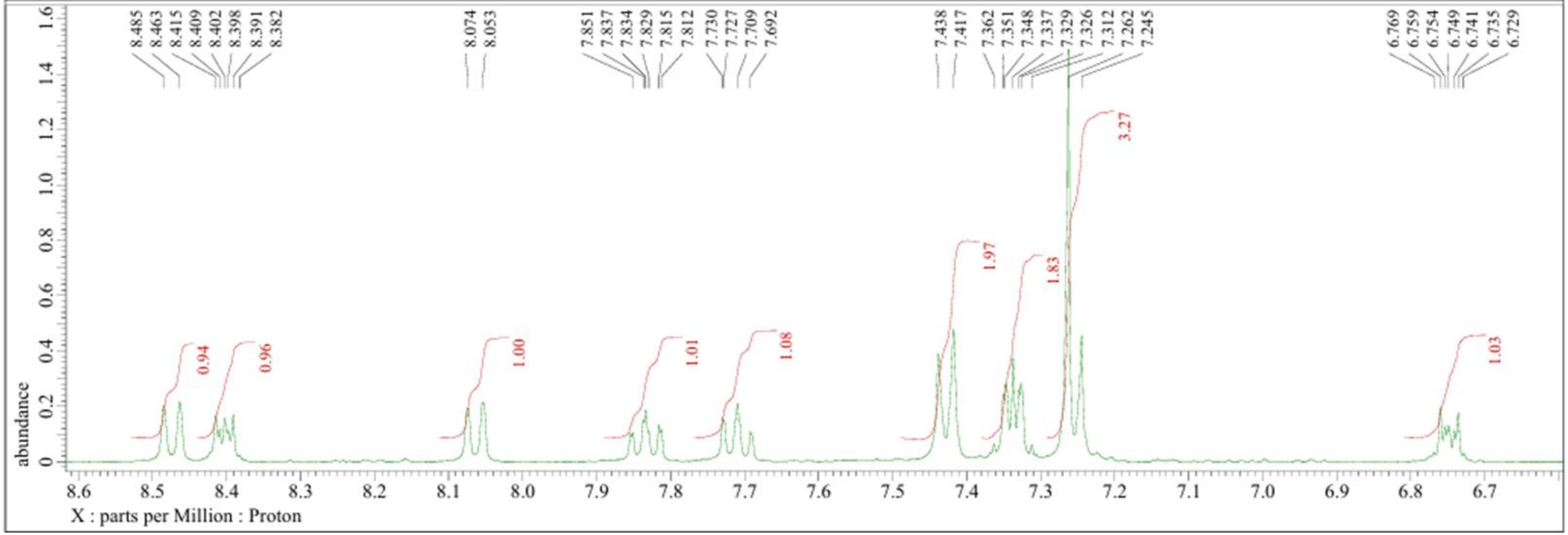

$^{13}C\{^1H\}$ NMR (100 MHz, $CDCl_3$)

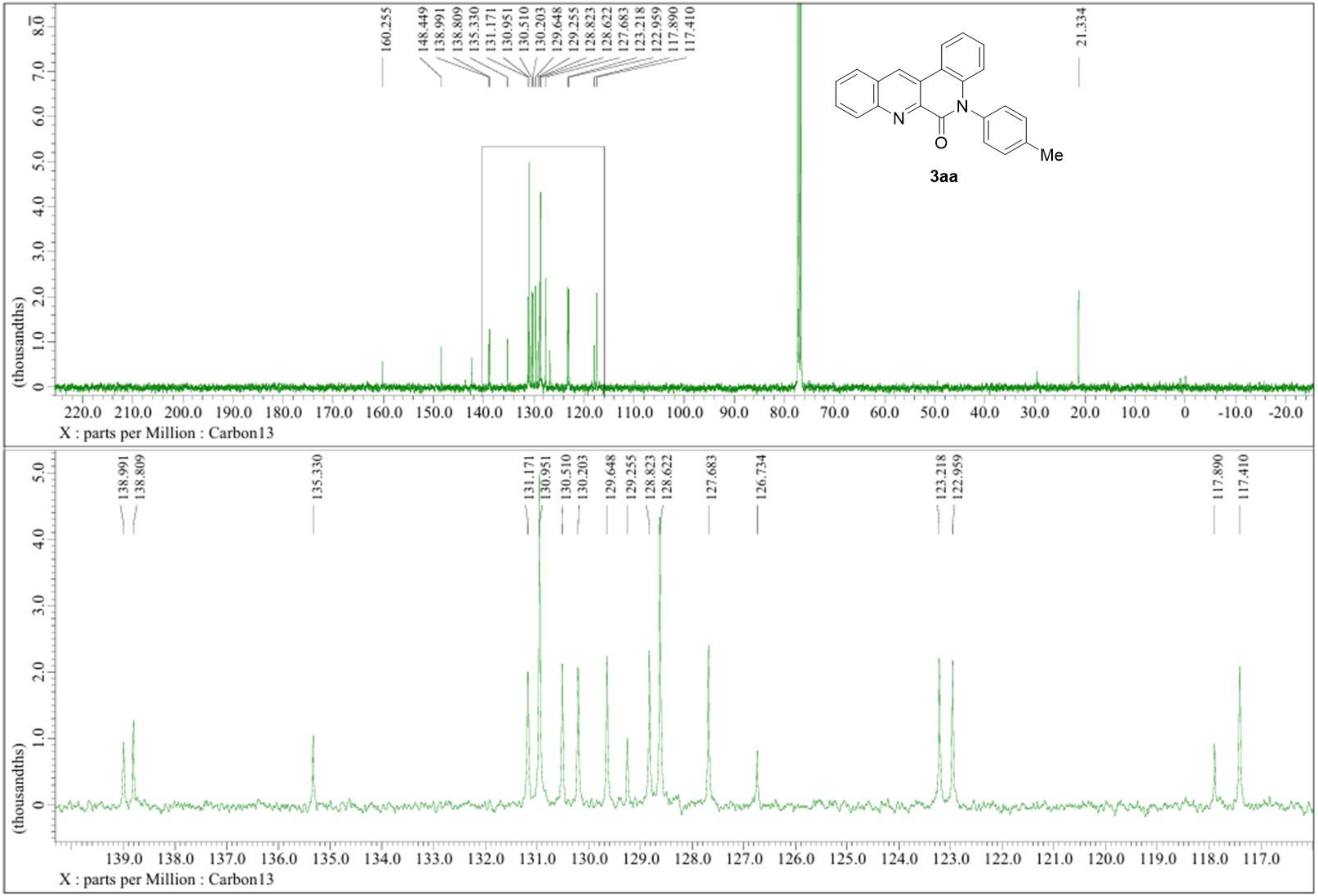

^{1}H NMR (400 MHz, $CDCl_3$)

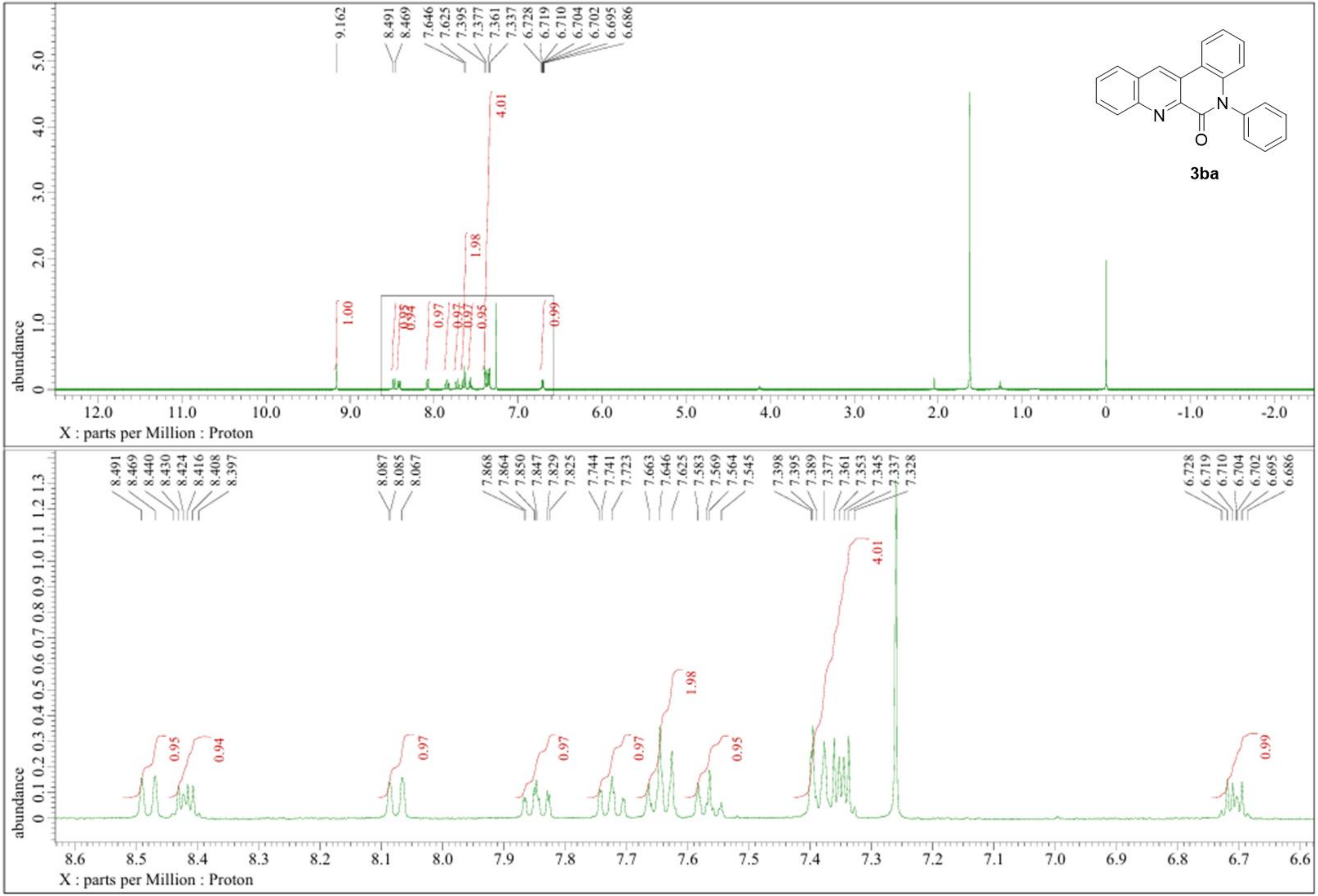

$^{13}C\{^{1}H\}$ NMR (100 MHz, $CDCl_3$)

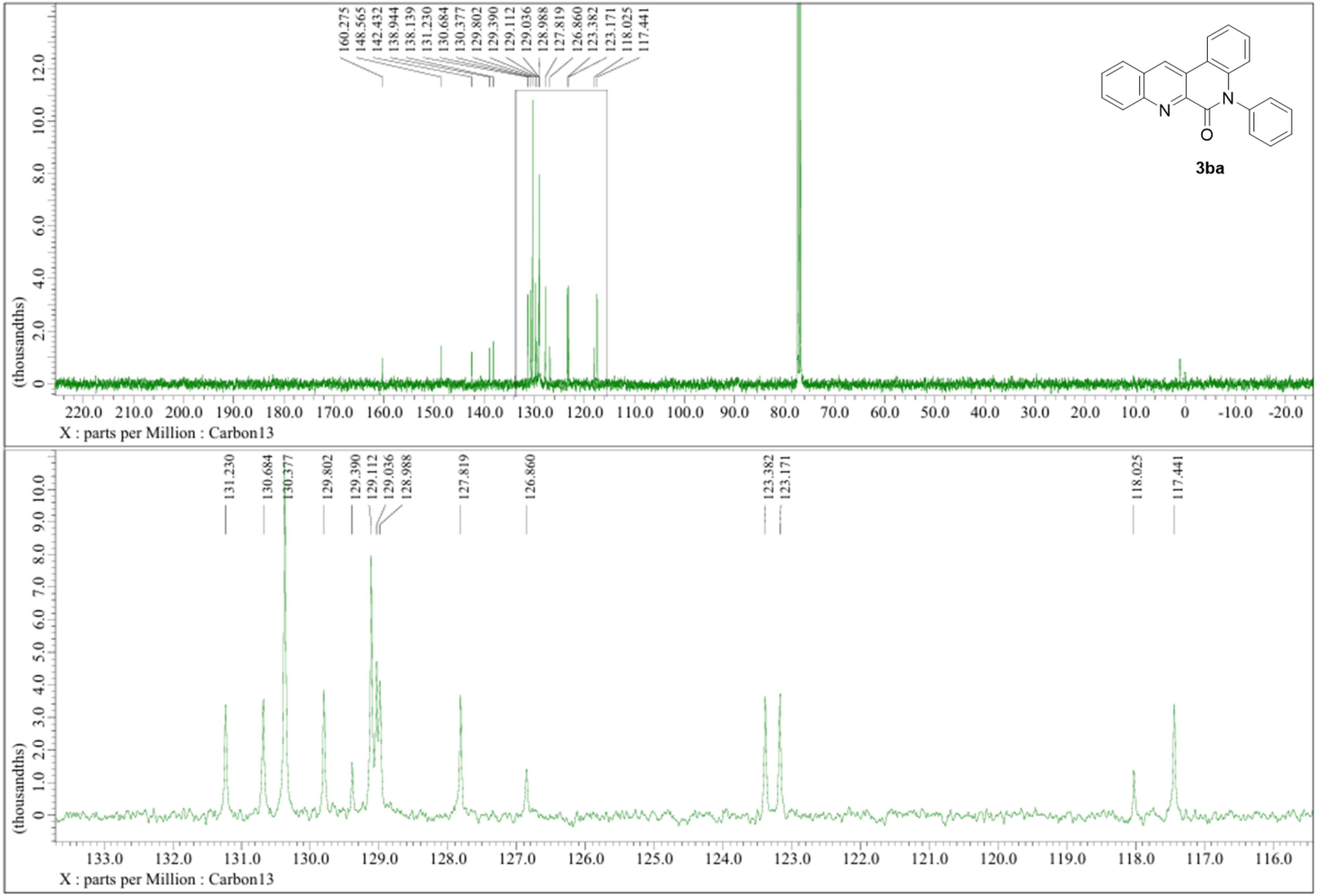

^{1}H NMR (400 MHz, $CDCl_3$)

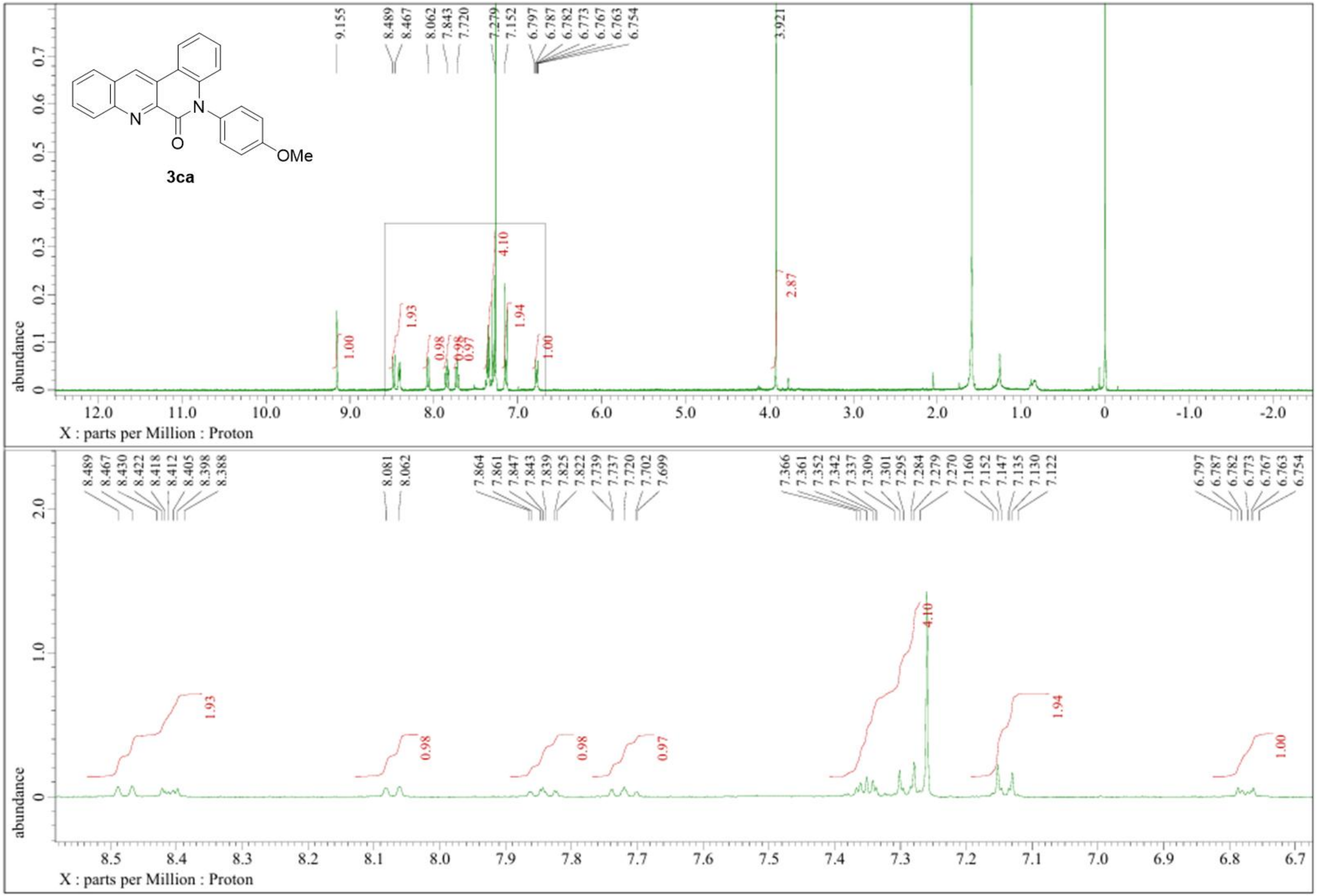

$^{13}C\{^1H\}$ NMR (100 MHz, $CDCl_3$)

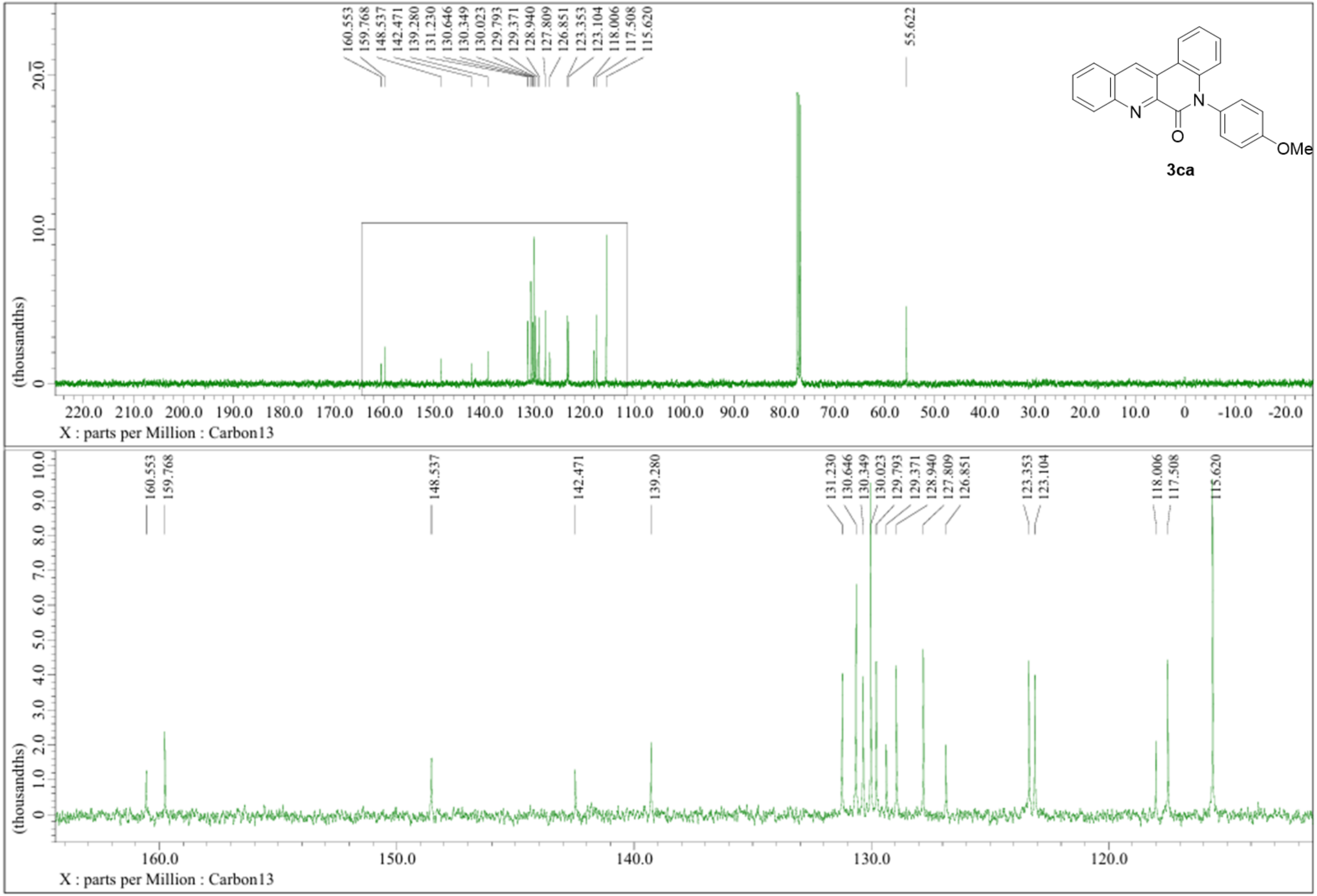

^{1}H NMR (400 MHz, $CDCl_3$)

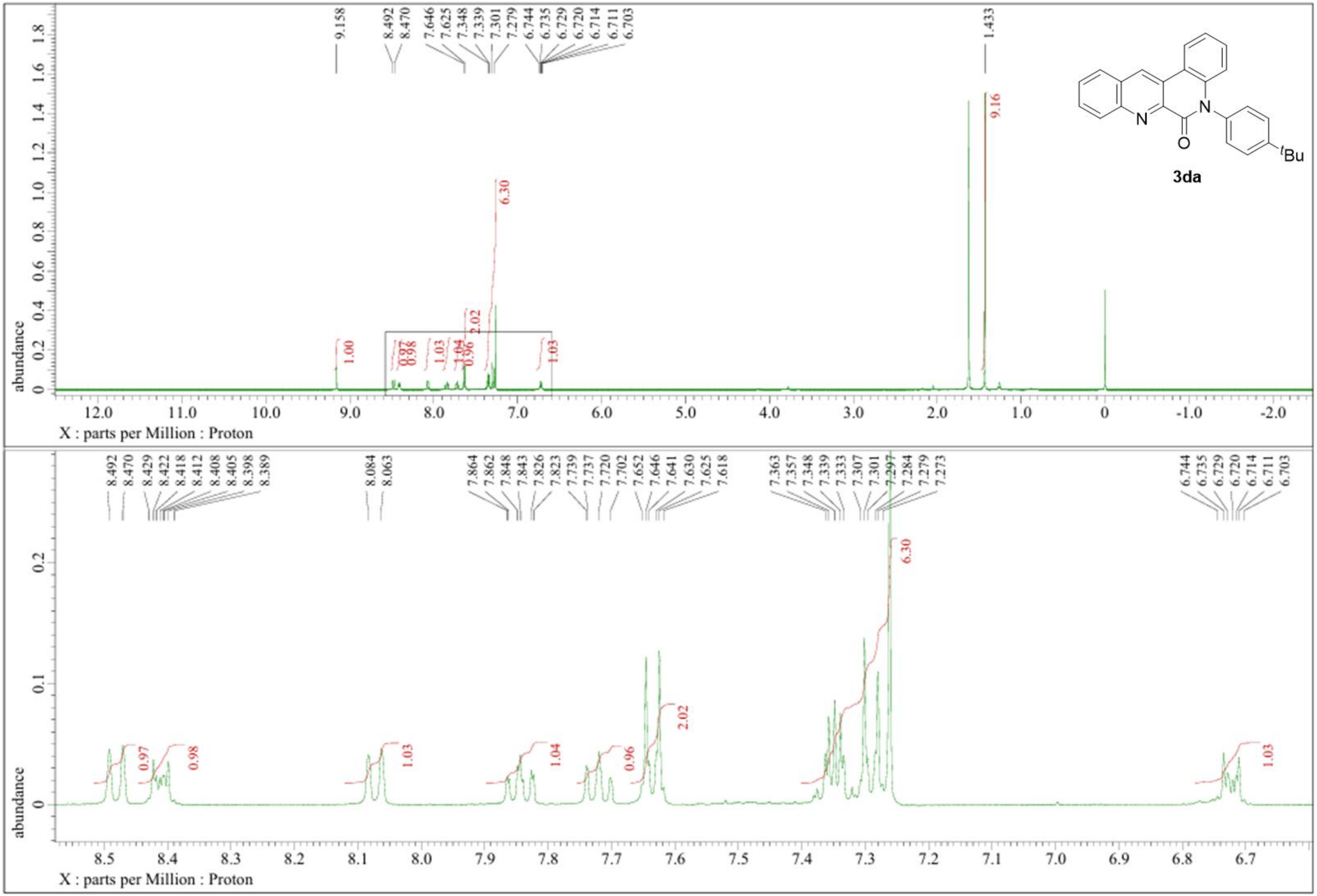

$^{13}C\{^{1}H\}$ NMR (100 MHz, $CDCl_3$)

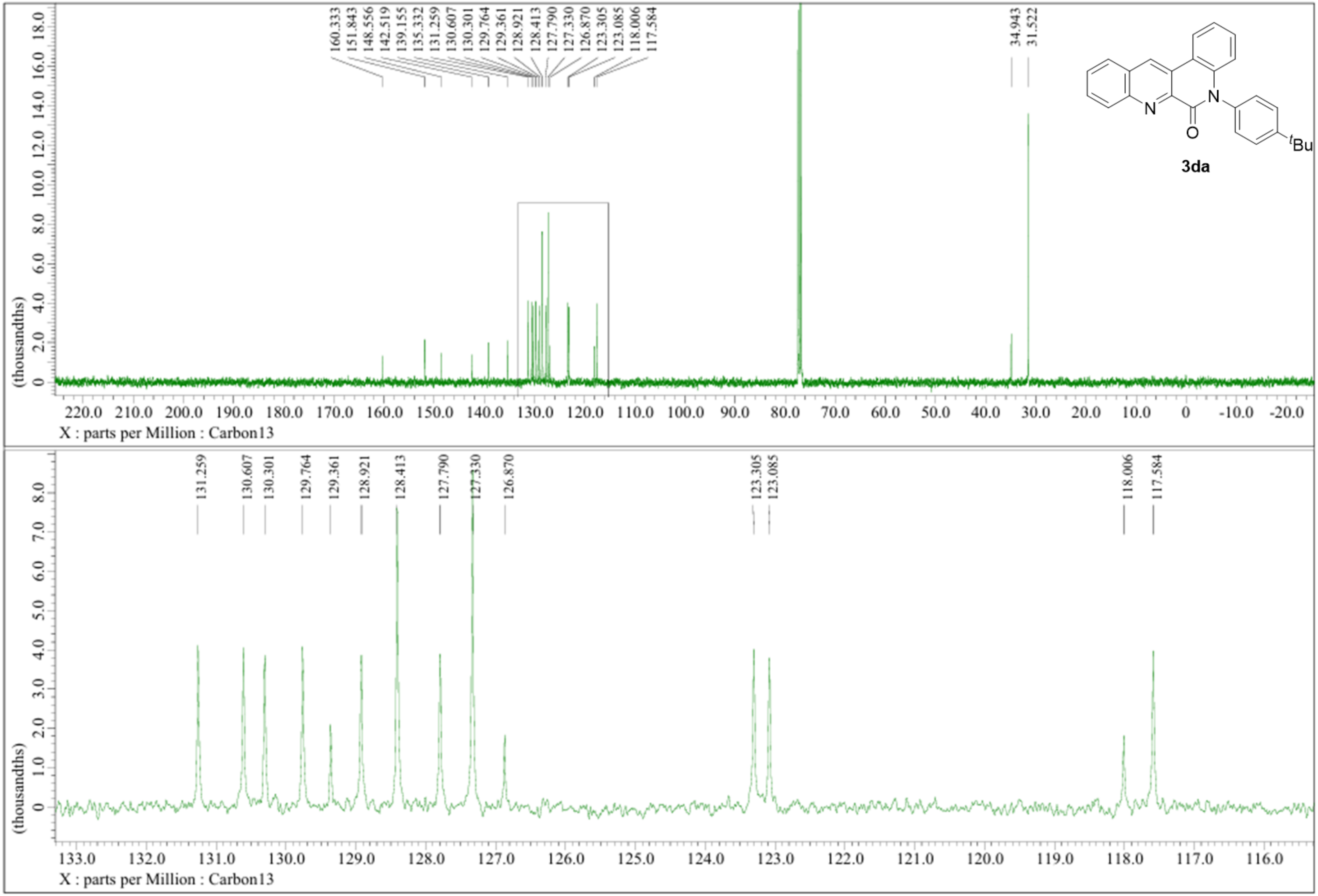

^{1}H NMR (400 MHz, $CDCl_3$)

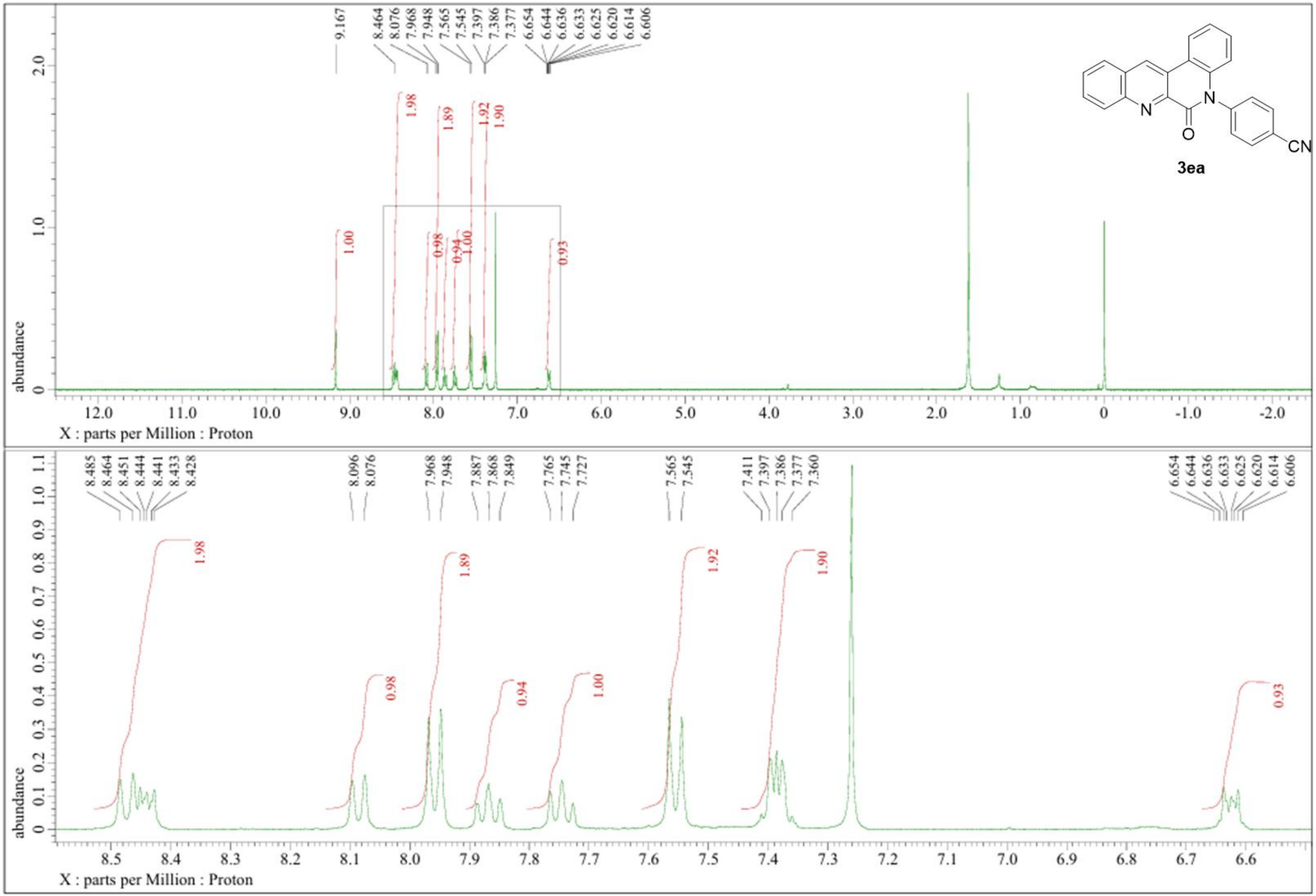

$^{13}C\{^{1}H\}$ NMR (100 MHz, $CDCl_3$)

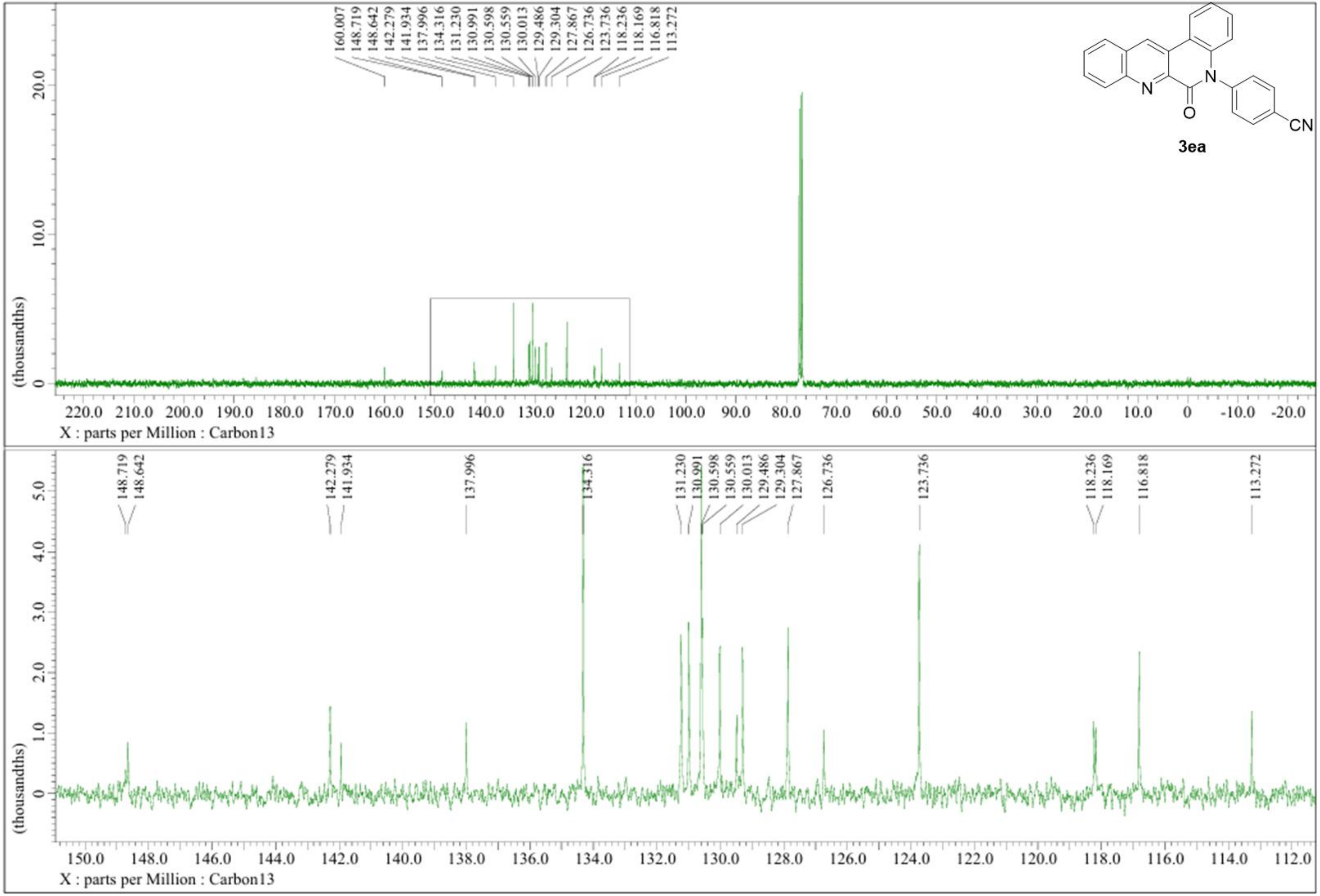

^{1}H NMR (400 MHz, $CDCl_3$)

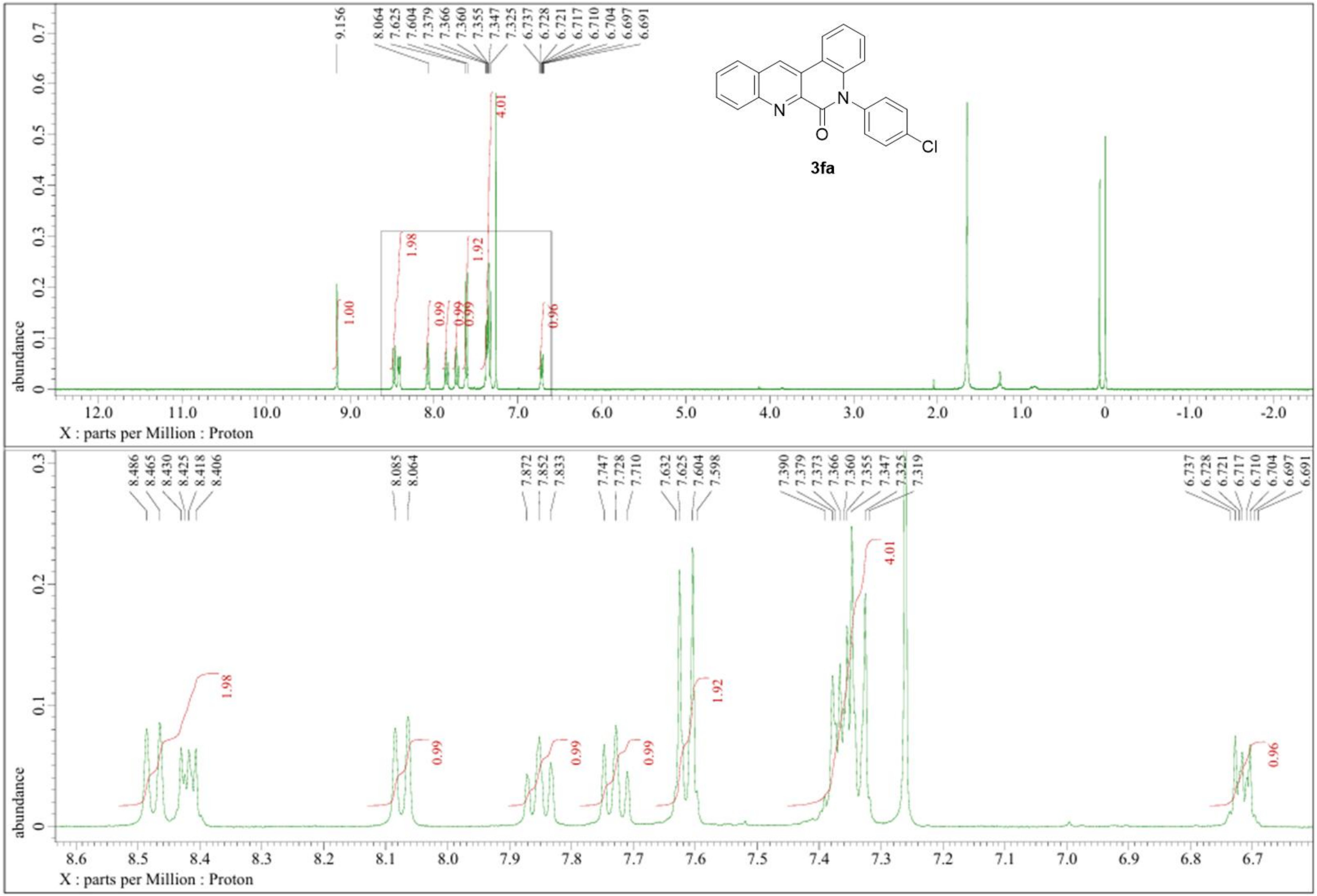

$^{13}C\{^{1}H\}$ NMR (100 MHz, $CDCl_3$)

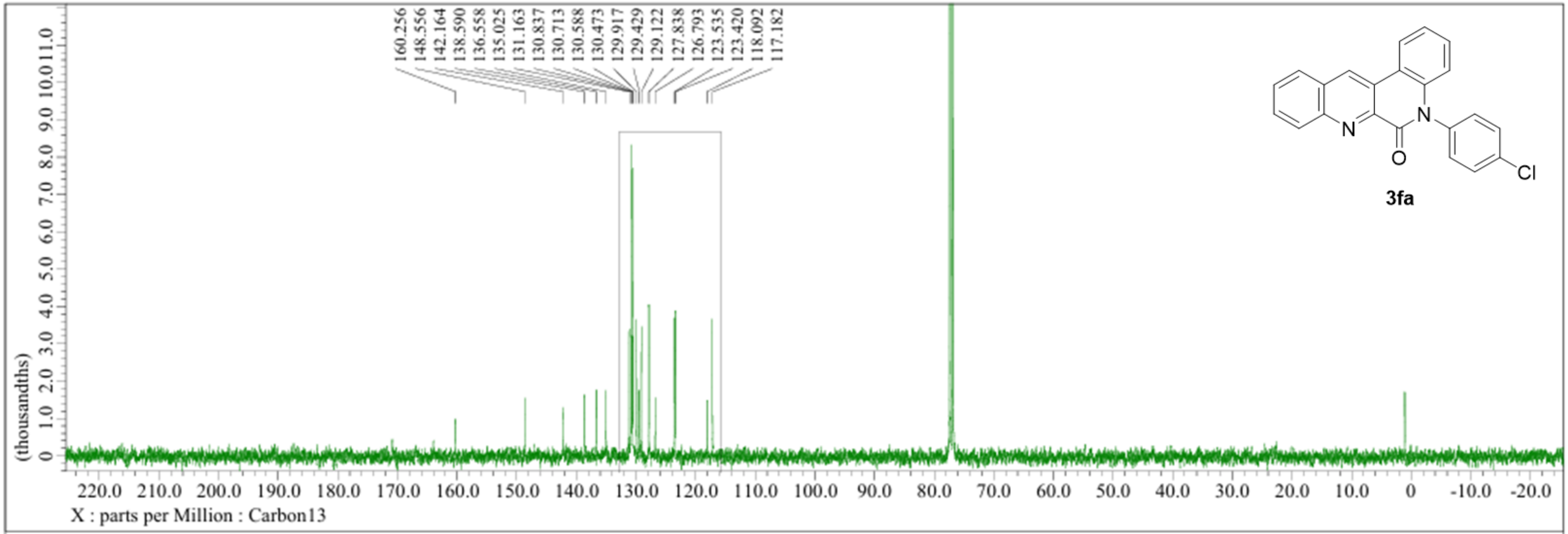

3fa

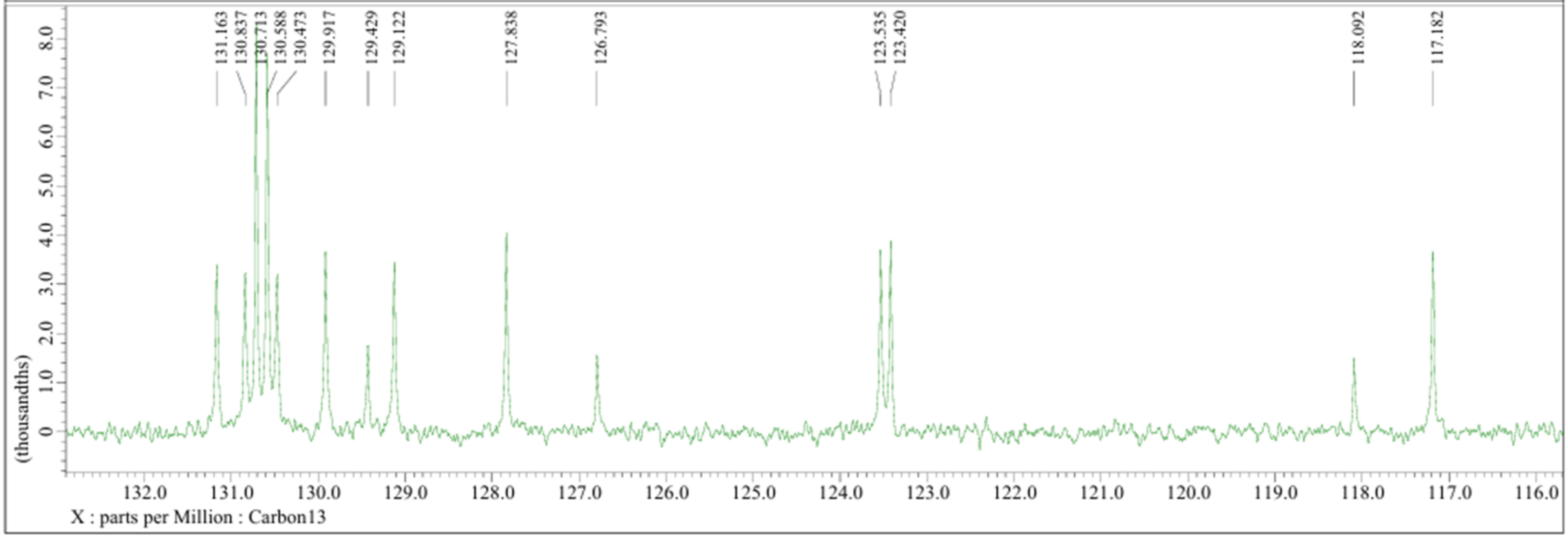

^{1}H NMR (400 MHz, $CDCl_3$)

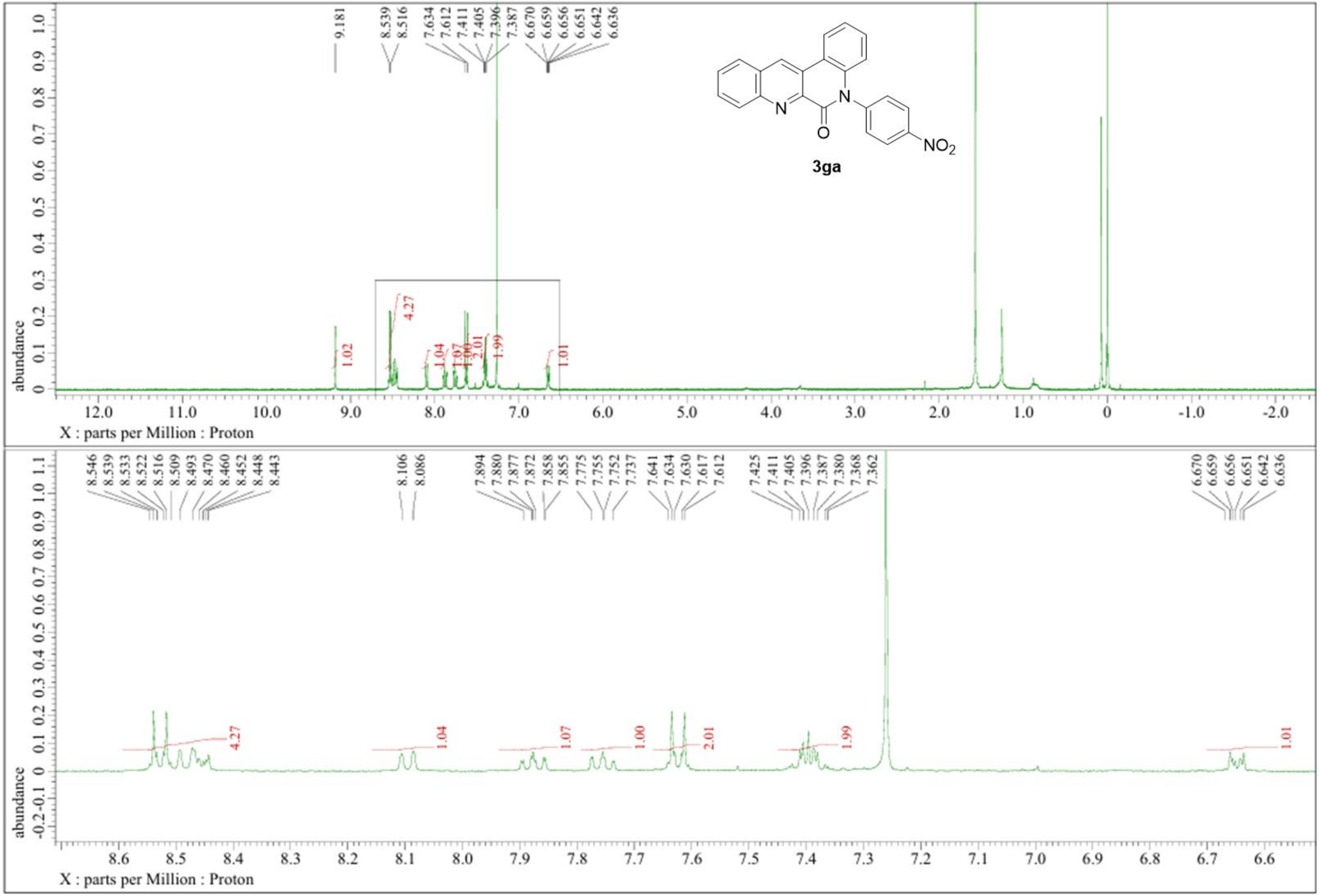

$^{13}C\{^{1}H\}$ NMR (100 MHz, CDCl$_3$)

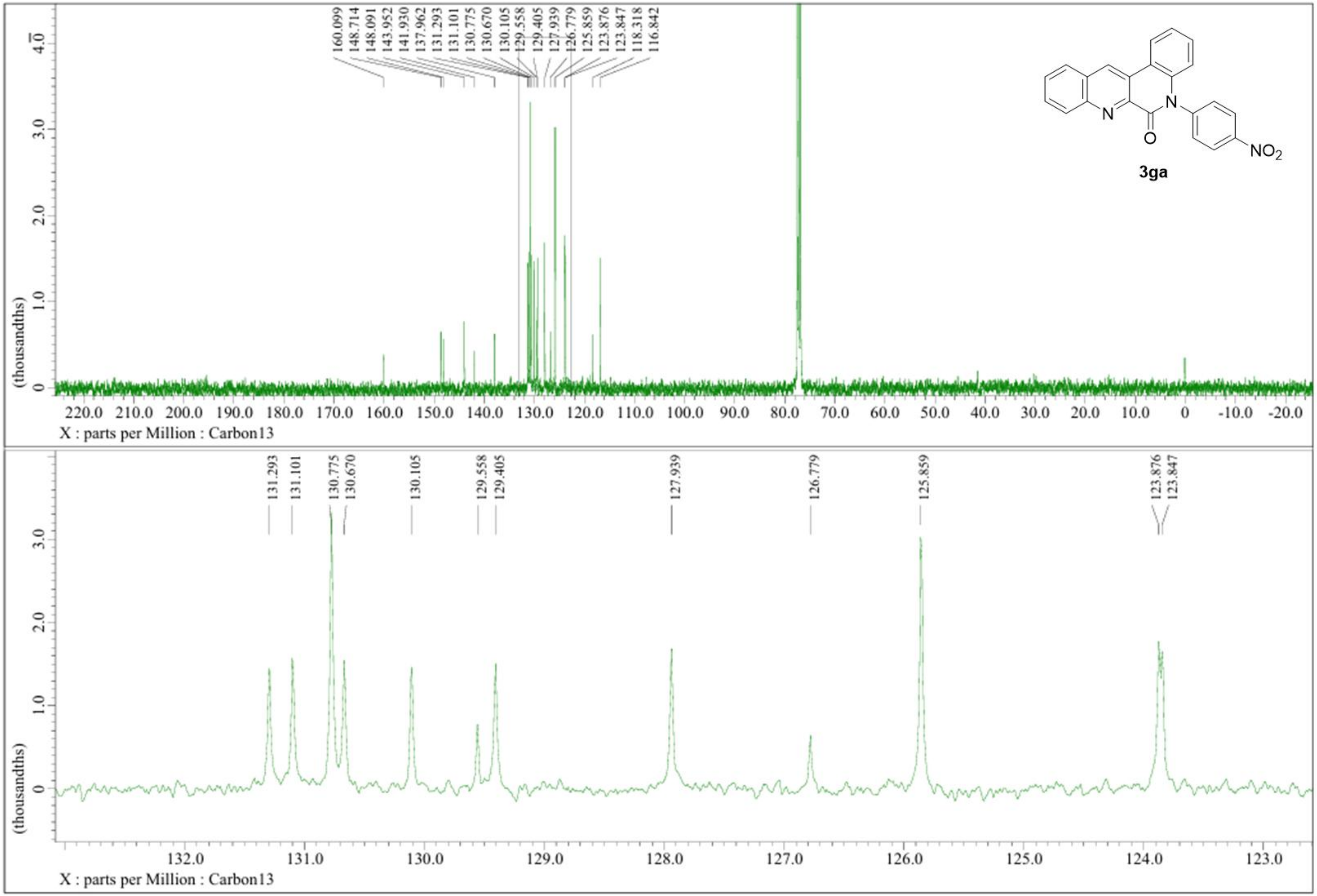

^{1}H NMR (400 MHz, $CDCl_3$)

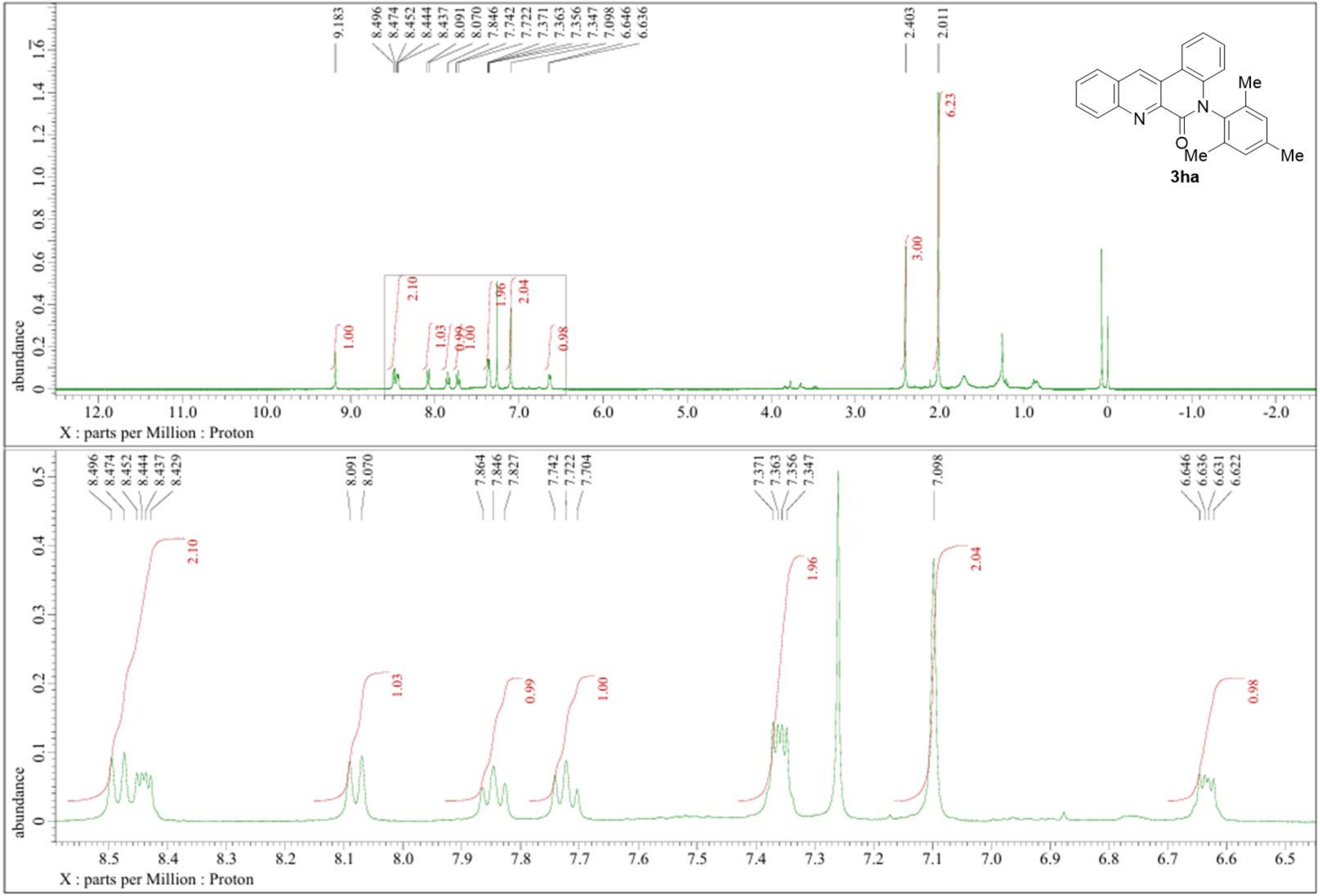

$^{13}C\{^1H\}$ NMR (100 MHz, $CDCl_3$)

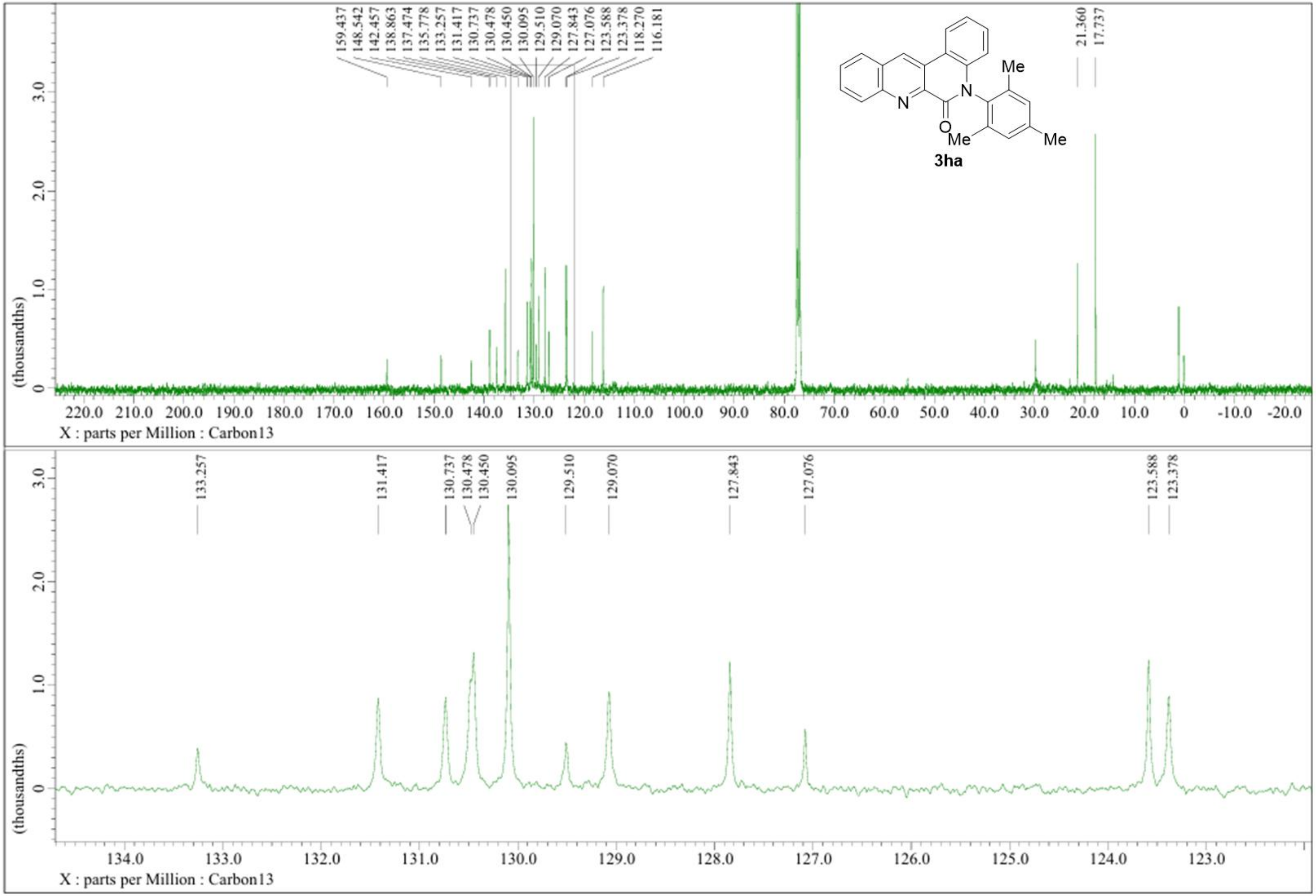

^{1}H NMR (400 MHz, $CDCl_3$)

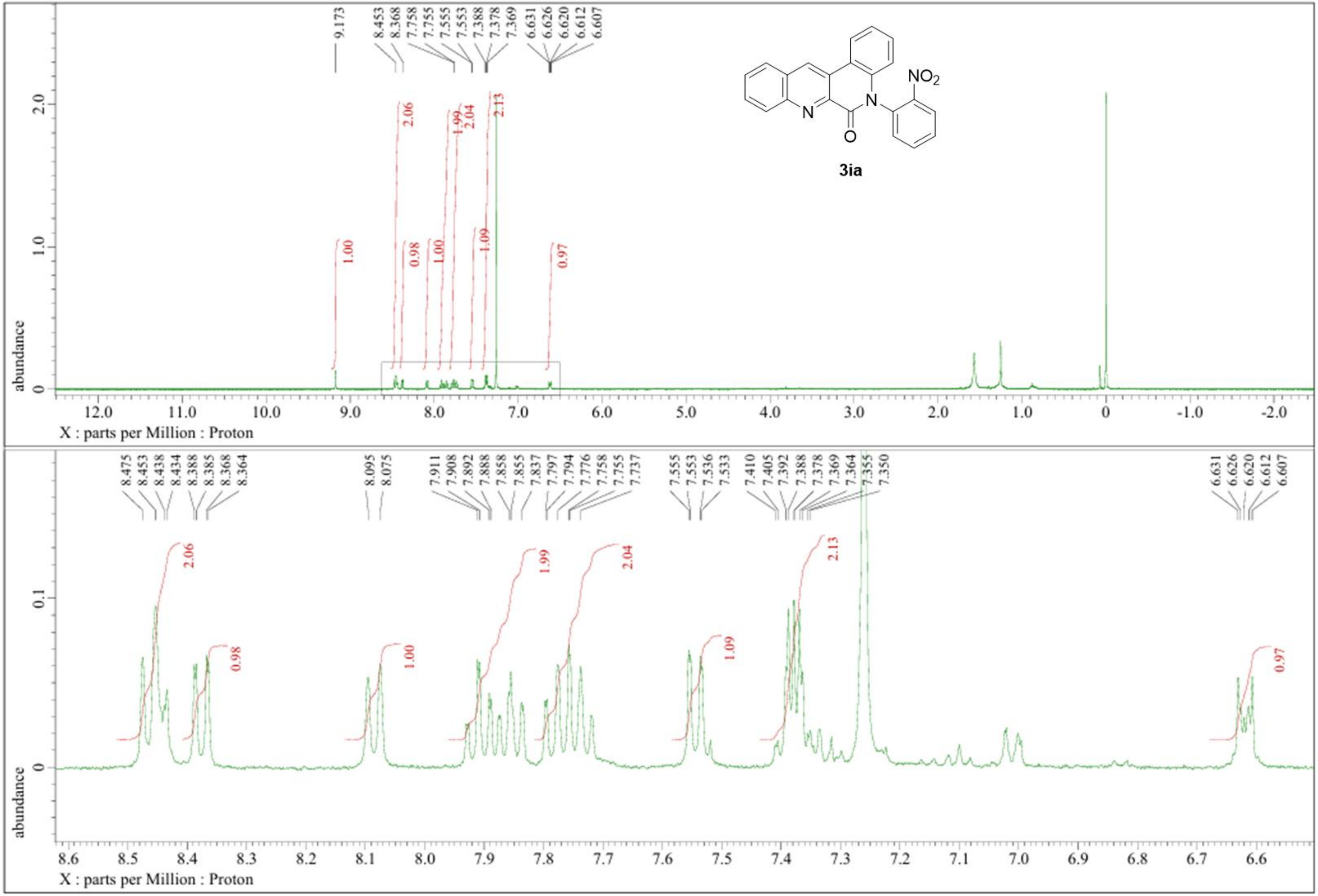

$^{13}C\{^1H\}$ NMR (100 MHz, $CDCl_3$)

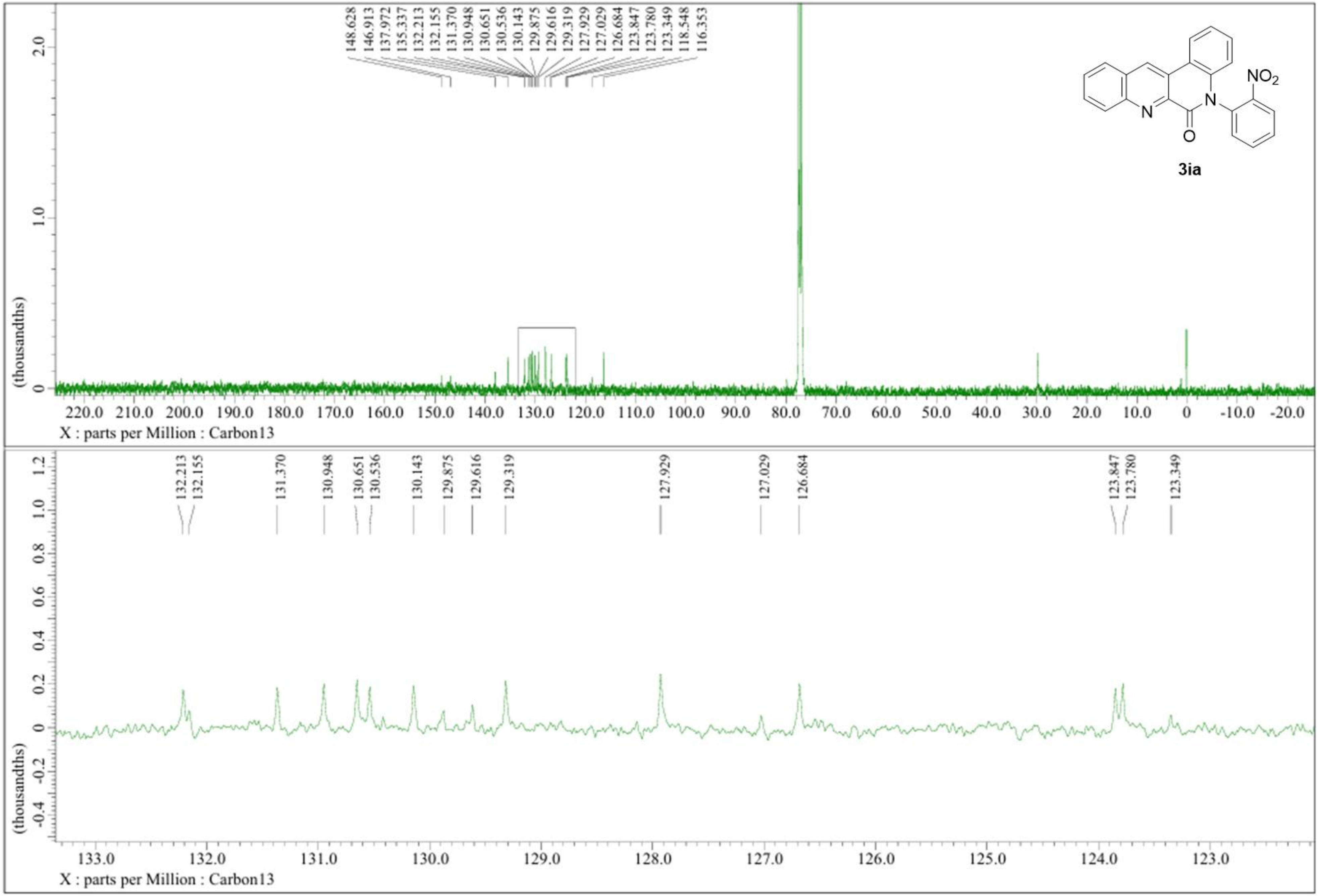

^{1}H NMR (400 MHz, $CDCl_3$)

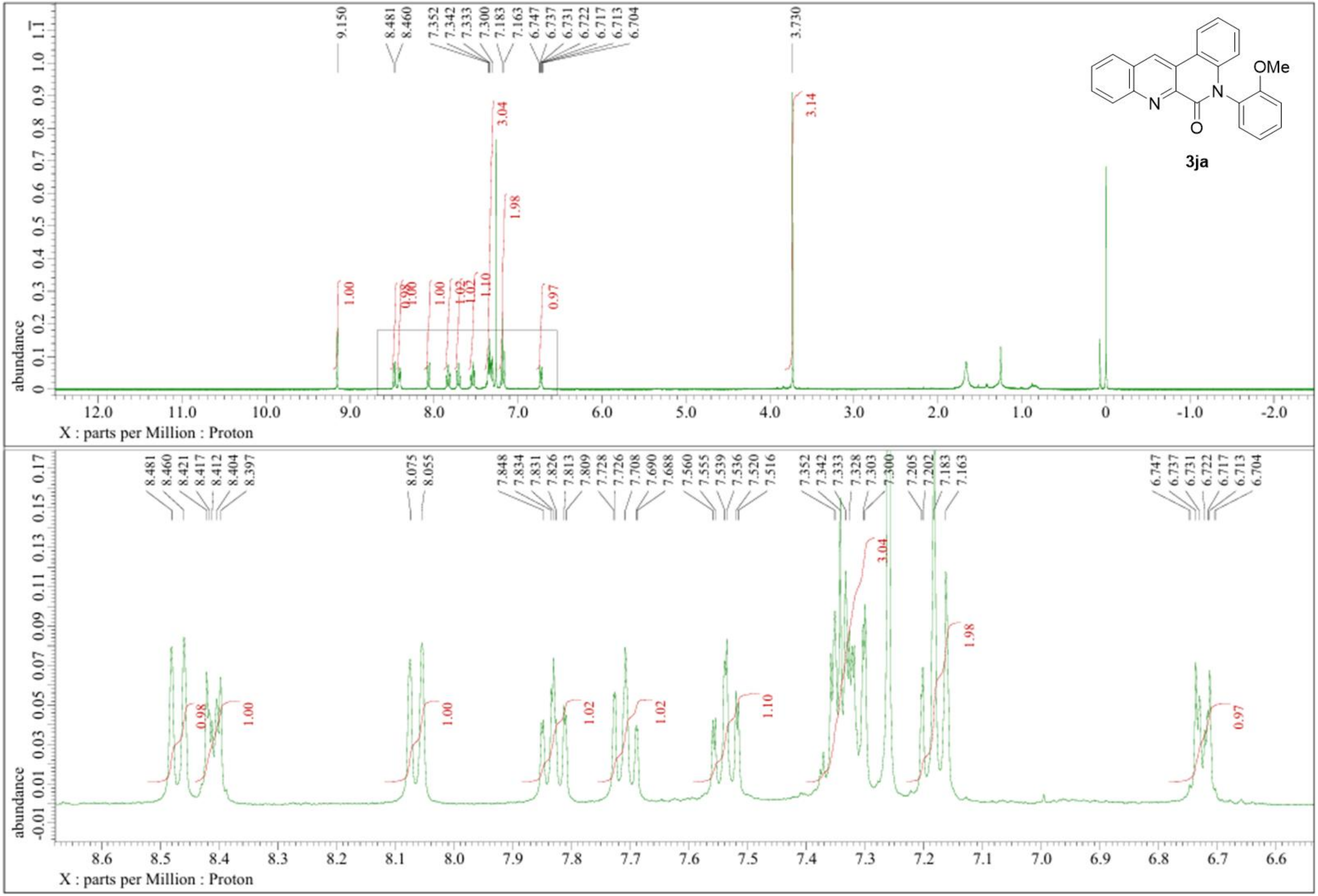

$^{13}C\{^{1}H\}$ NMR (100 MHz, $CDCl_3$)

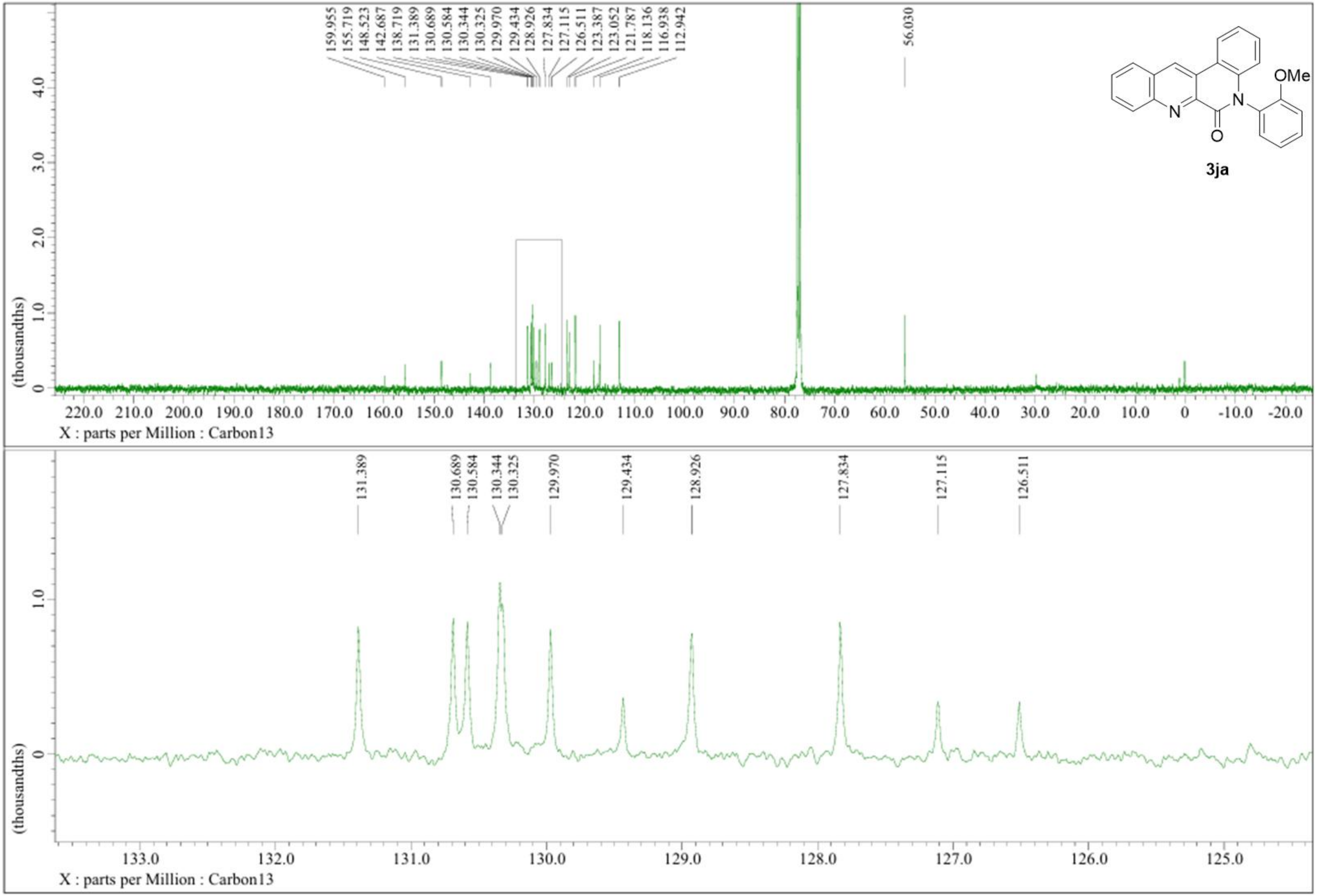

^{1}H NMR (400 MHz, $CDCl_3$)

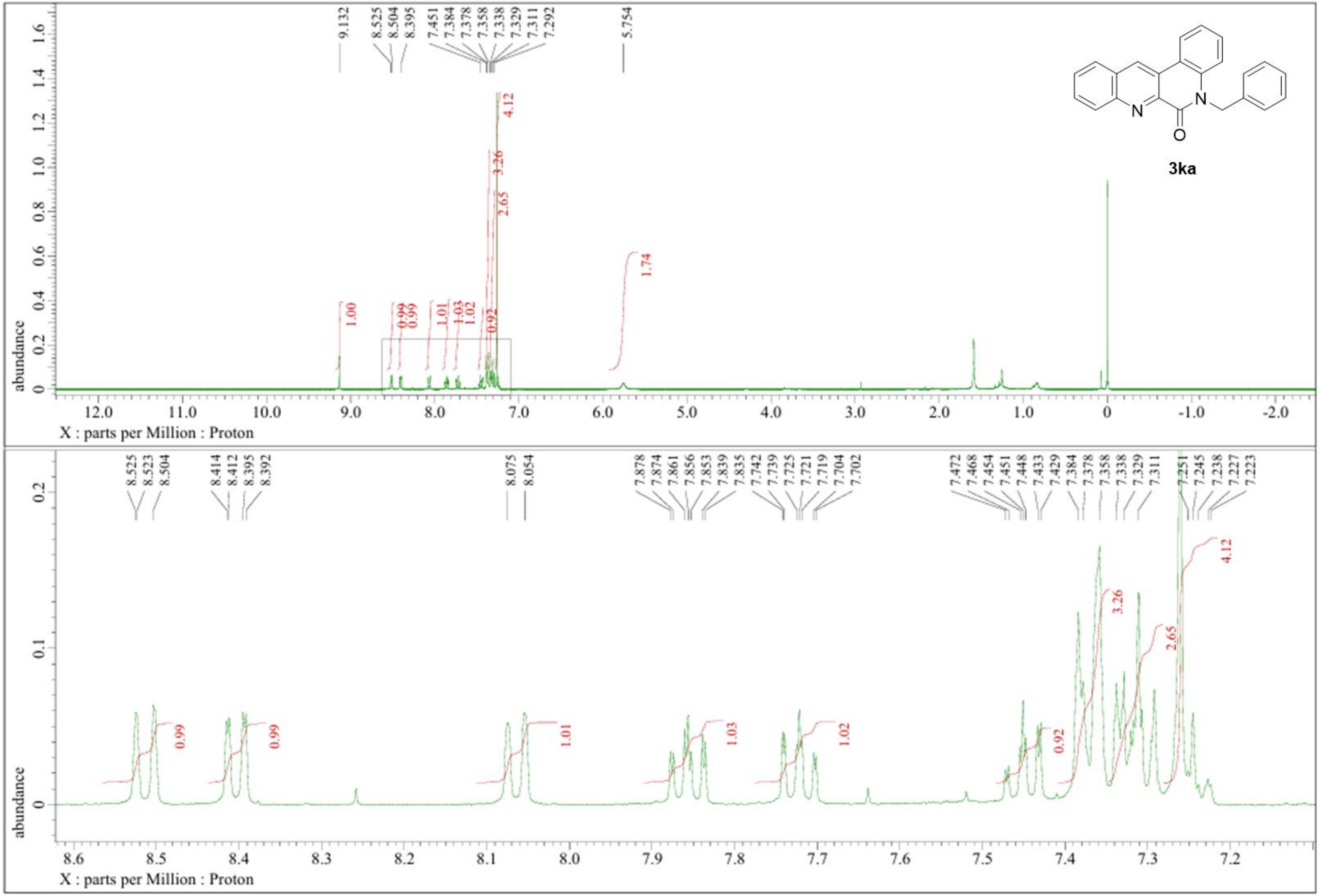

$^{13}C\{^1H\}$ NMR (100 MHz, $CDCl_3$)

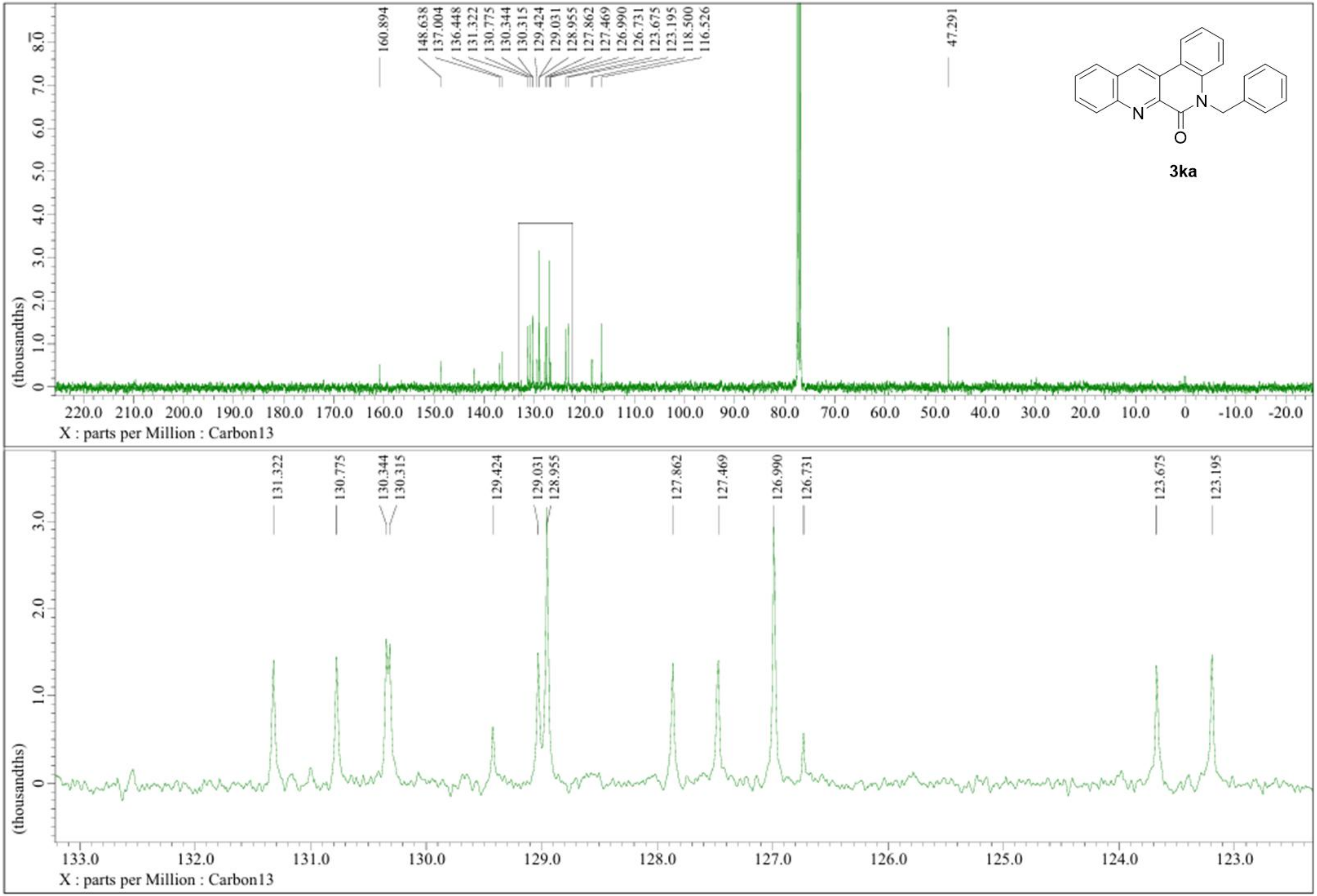

^{1}H NMR (400 MHz, $CDCl_3$)

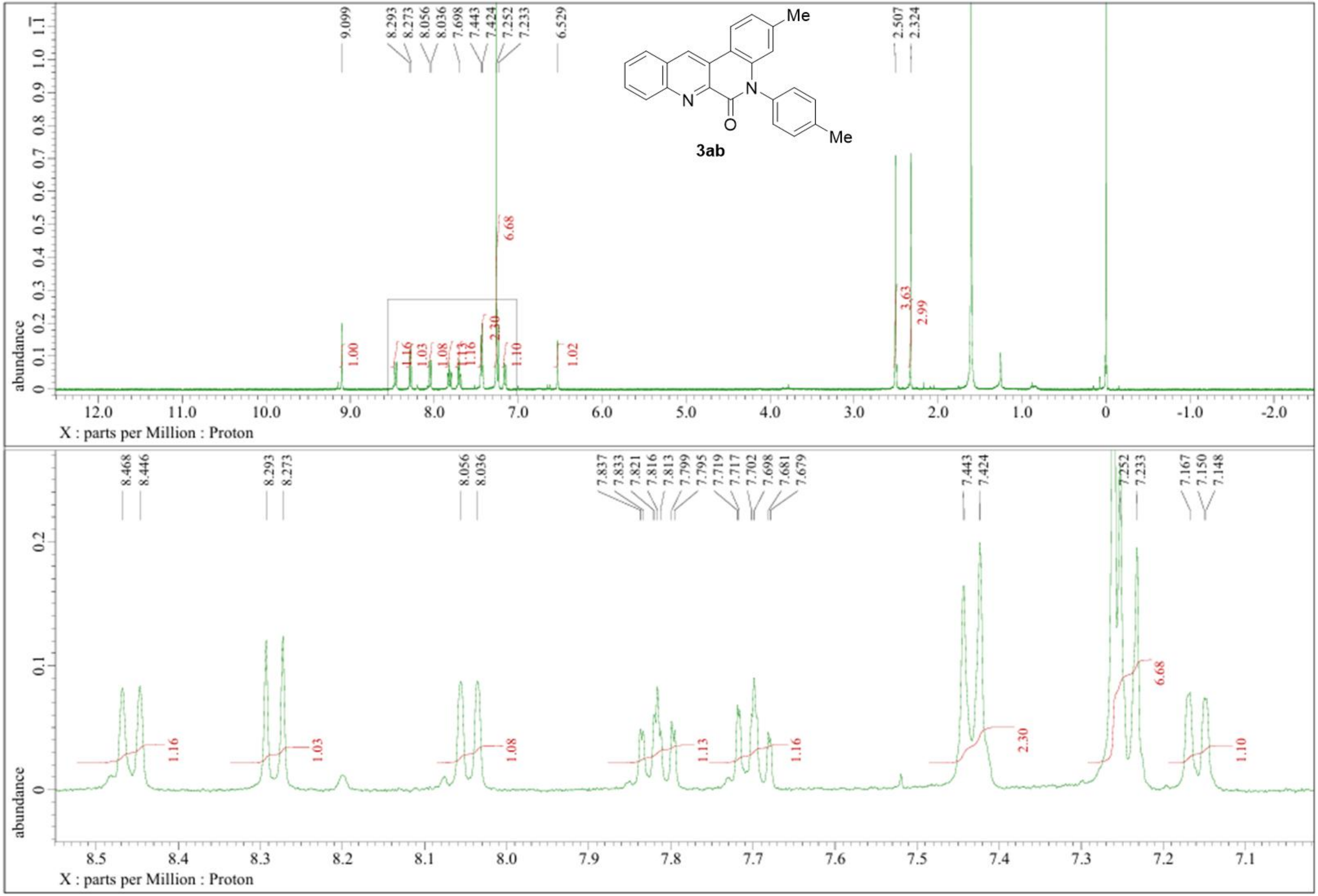

$^{13}C\{^1H\}$ NMR (100 MHz, $CDCl_3$)

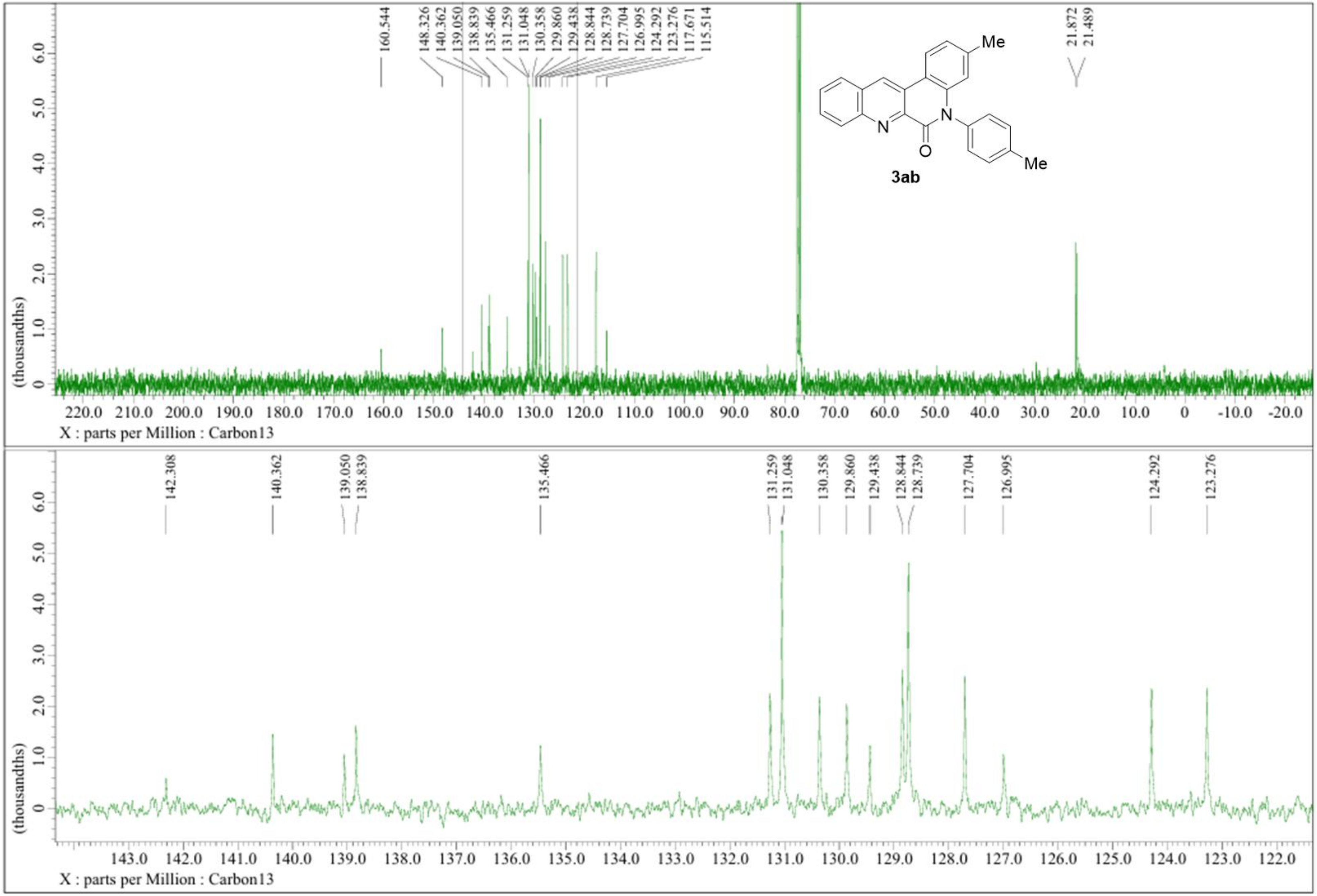

^{1}H NMR (400 MHz, $CDCl_3$)

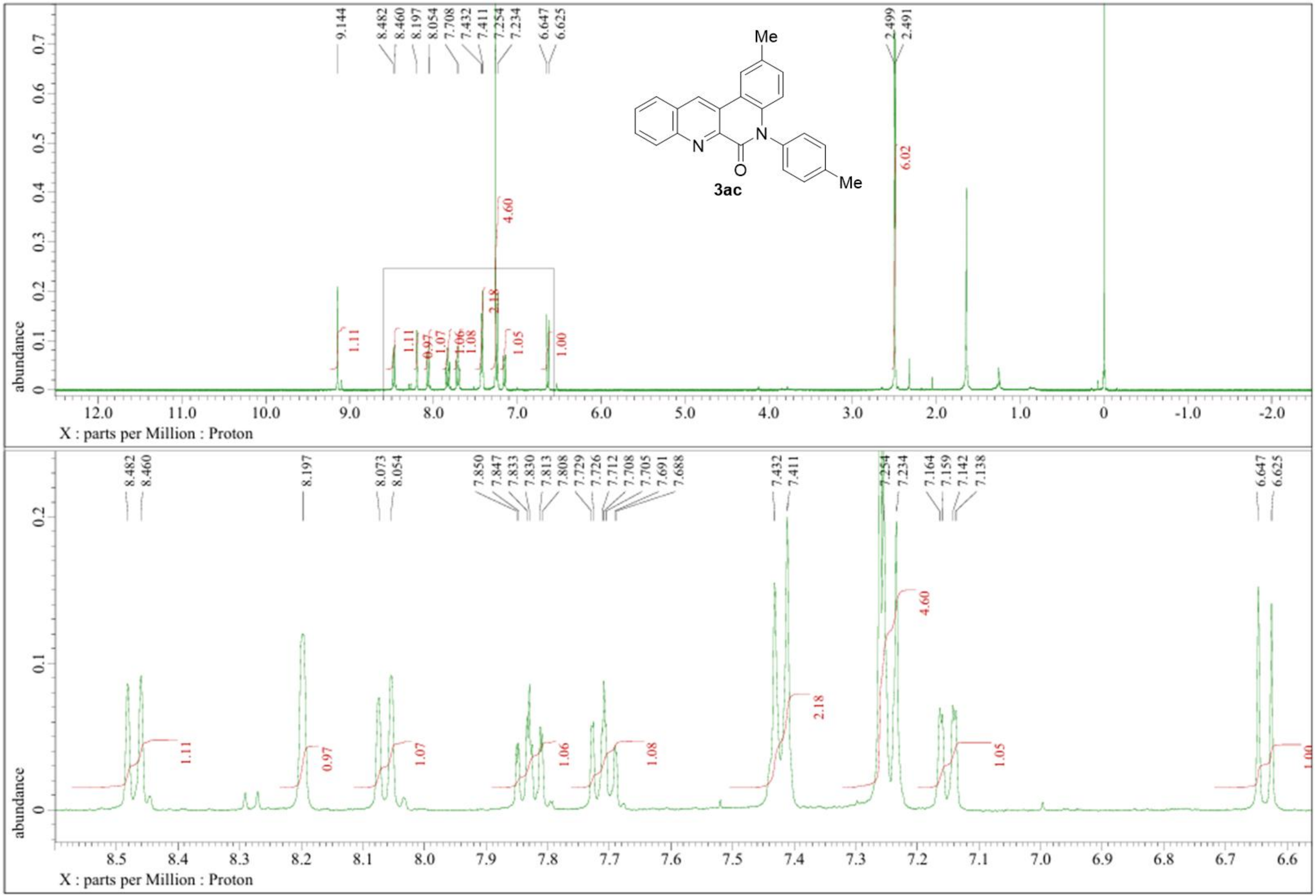

$^{13}C\{^1H\}$ NMR (100 MHz, $CDCl_3$)

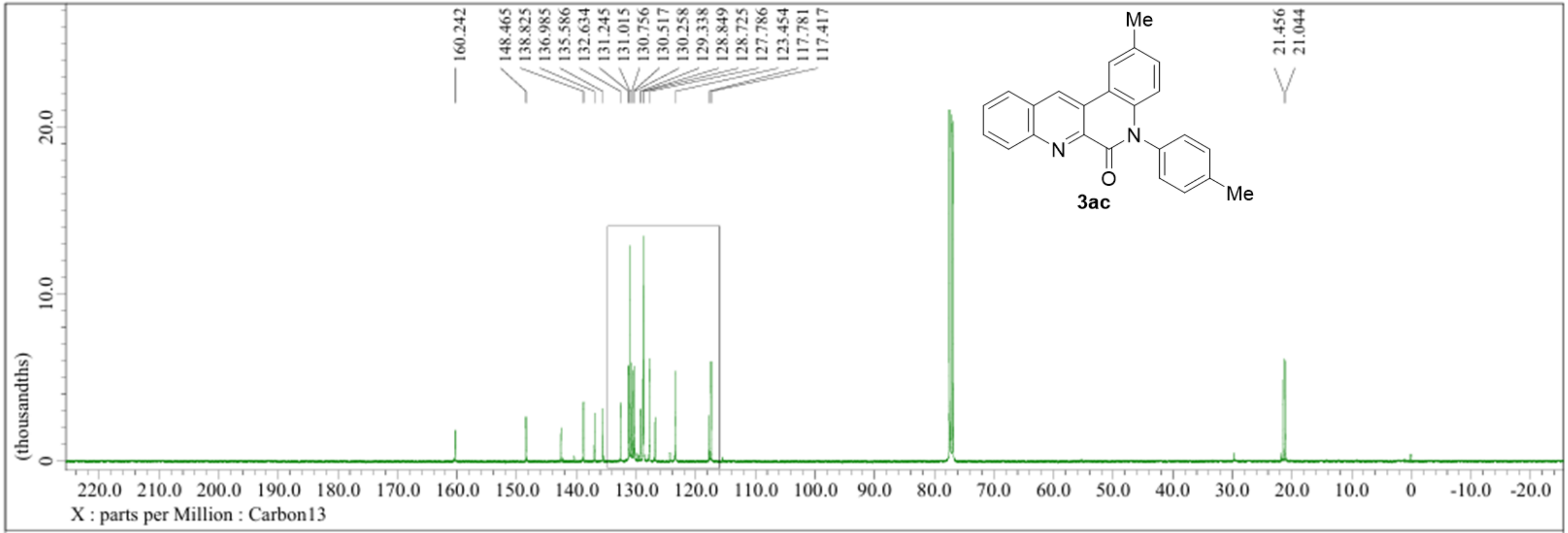

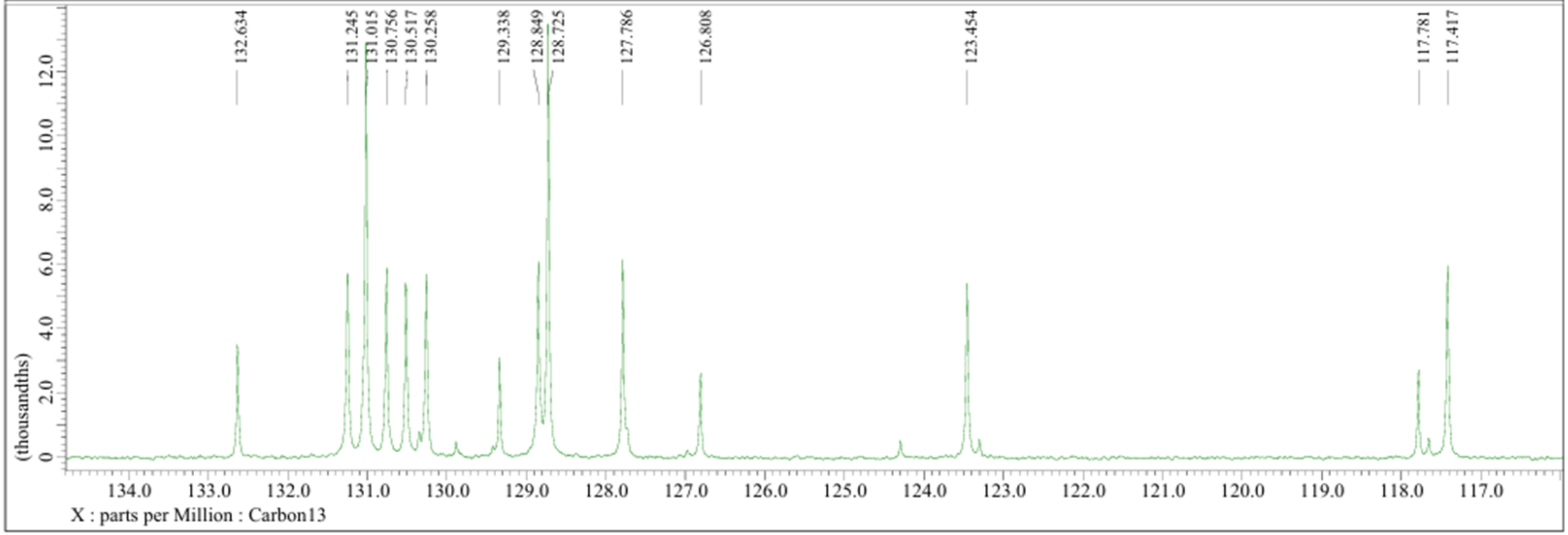

^{1}H NMR (400 MHz, $CDCl_3$)

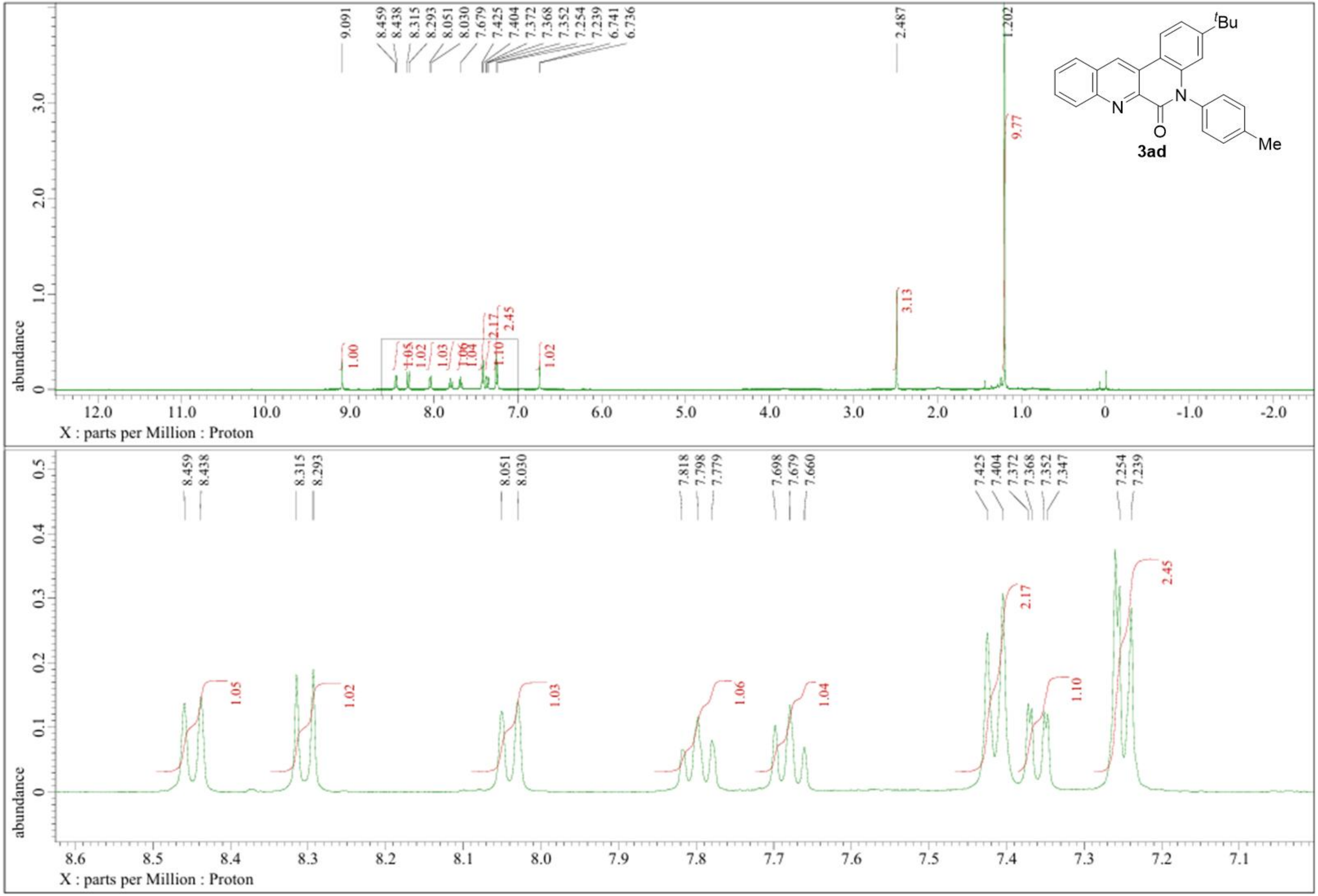

$^{13}C\{^{1}H\}$ NMR (100 MHz, $CDCl_3$)

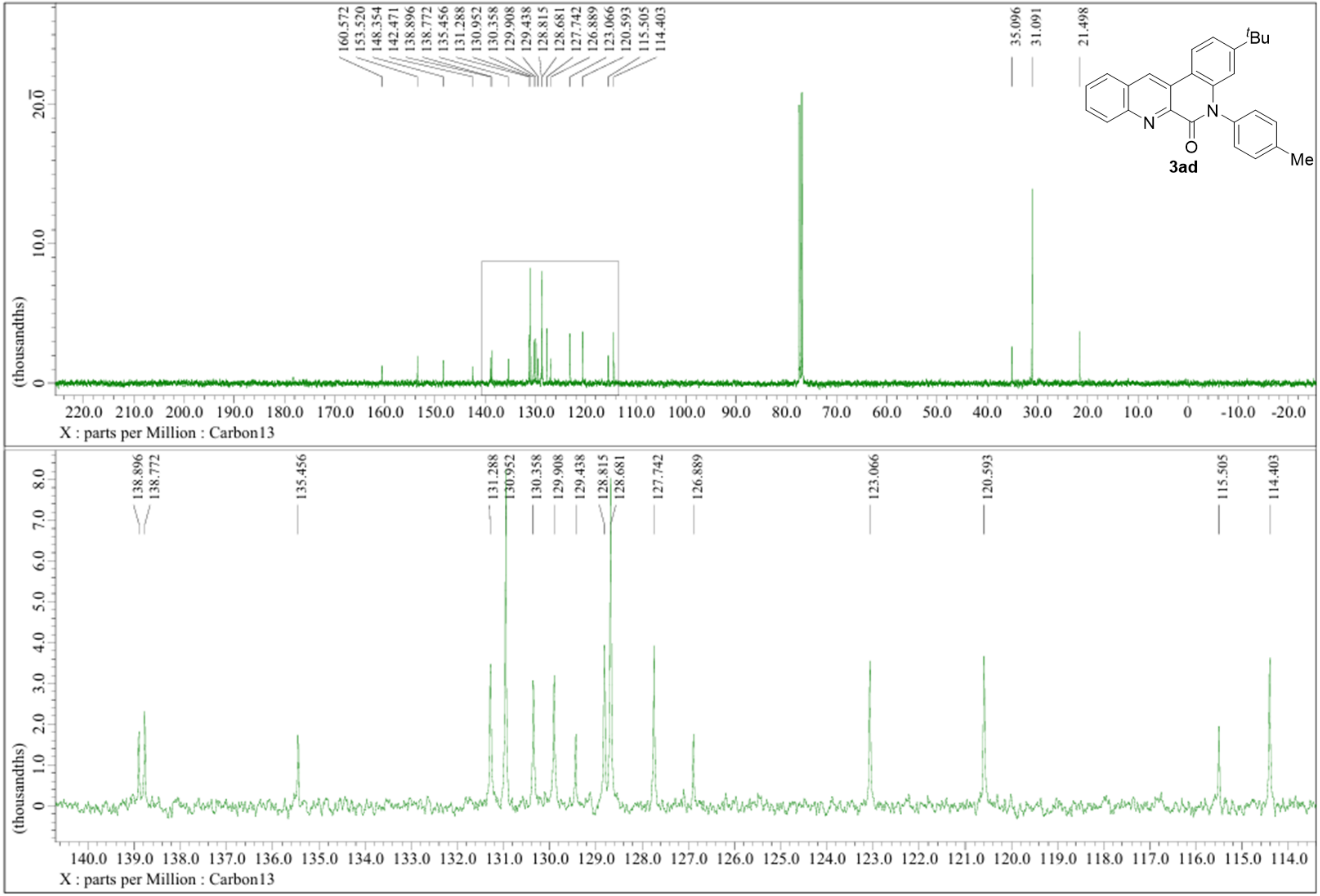

^{1}H NMR (400 MHz, $CDCl_3$)

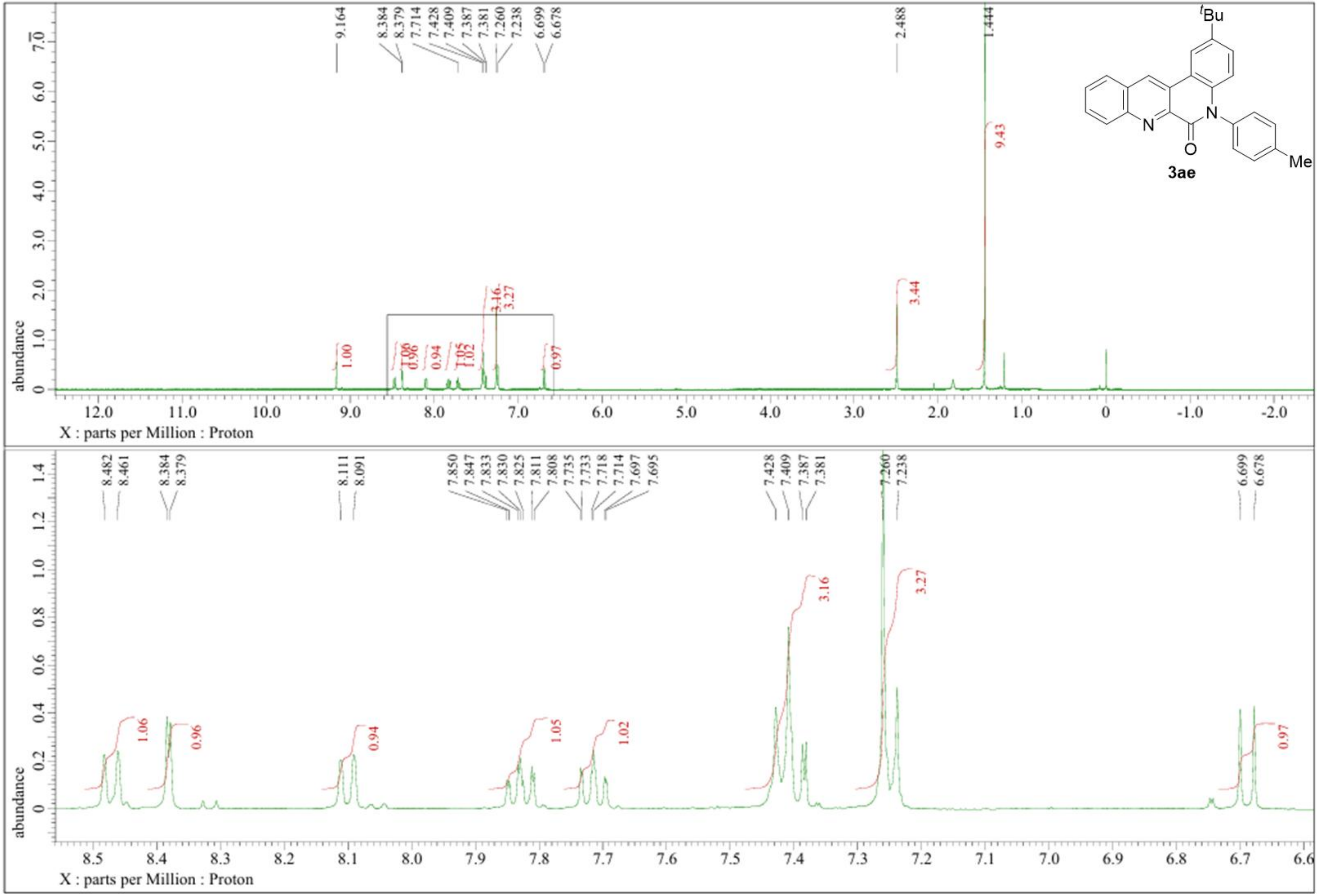

$^{13}C\{^1H\}$ NMR (100 MHz, $CDCl_3$)

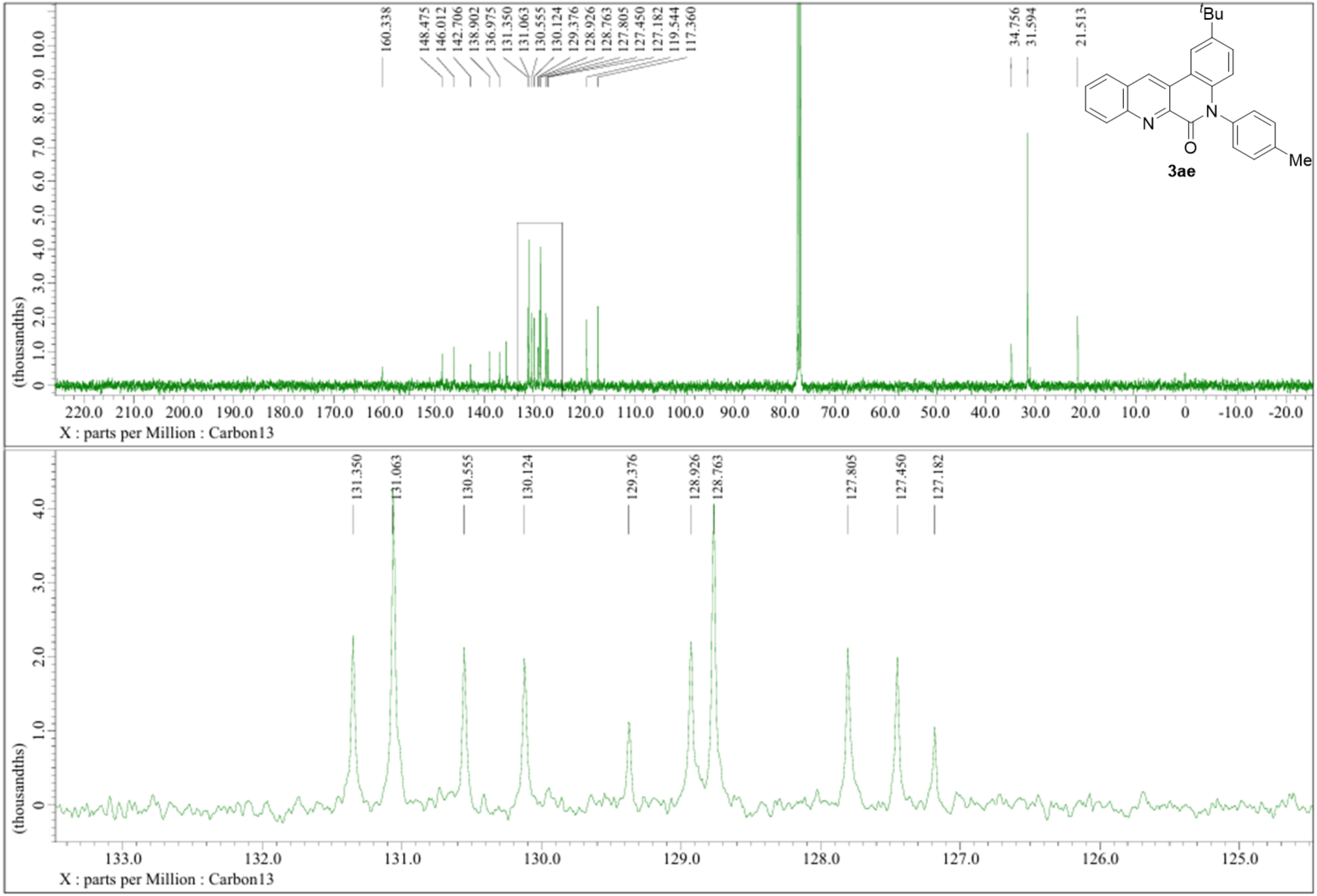

^{1}H NMR (400 MHz, $CDCl_3$)

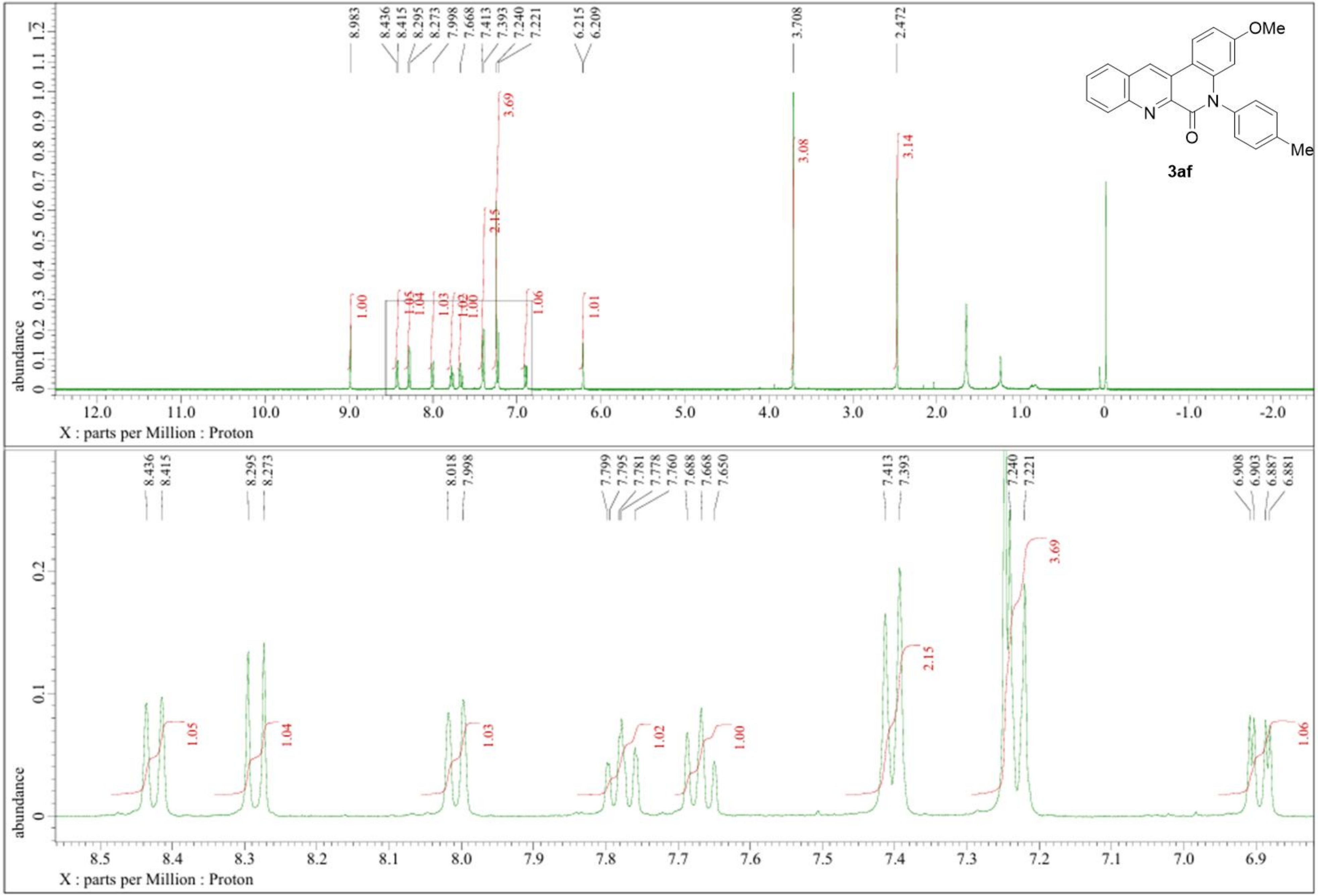

$^{13}C\{^1H\}$ NMR (100 MHz, $CDCl_3$)

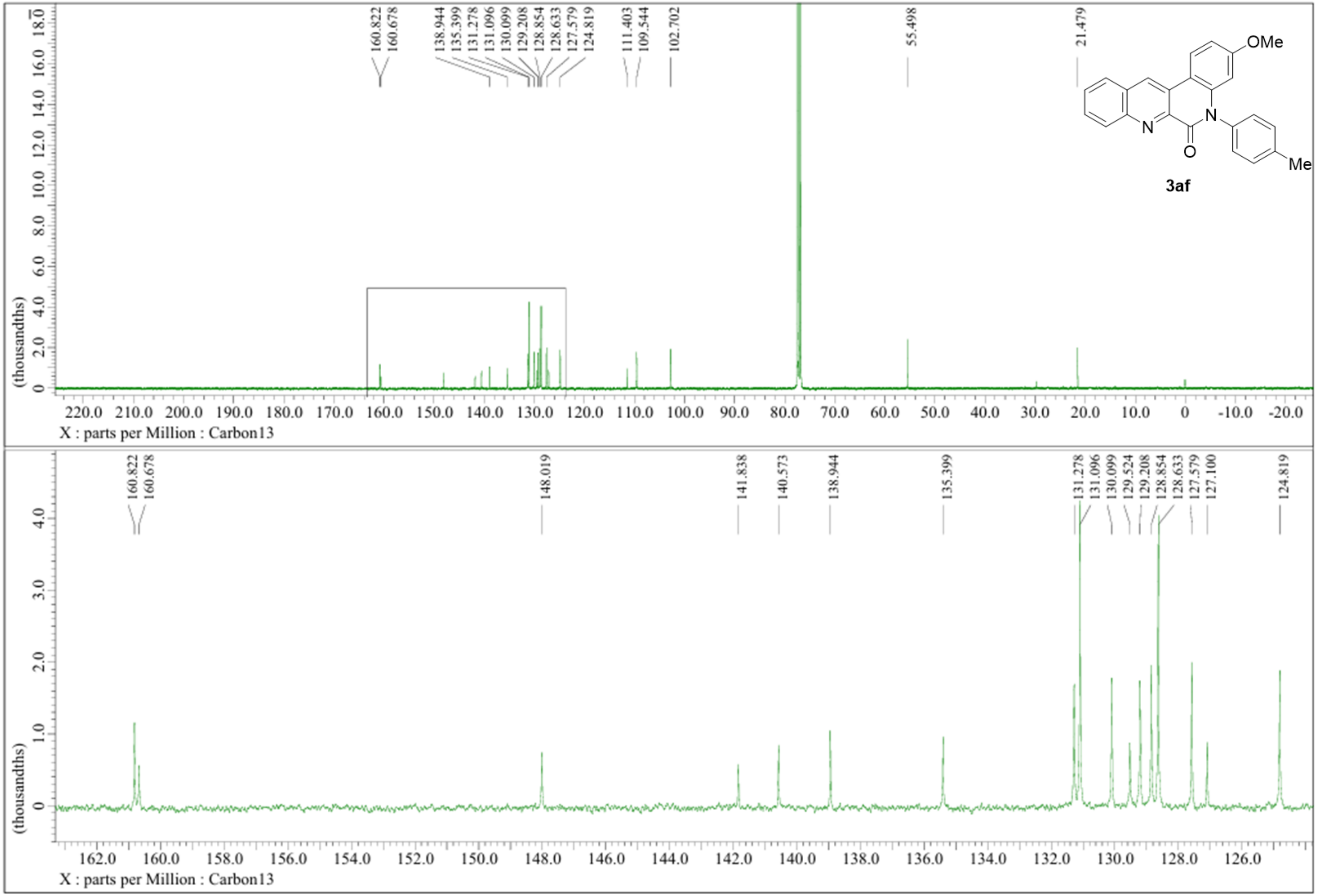

^{1}H NMR (400 MHz, $CDCl_3$)

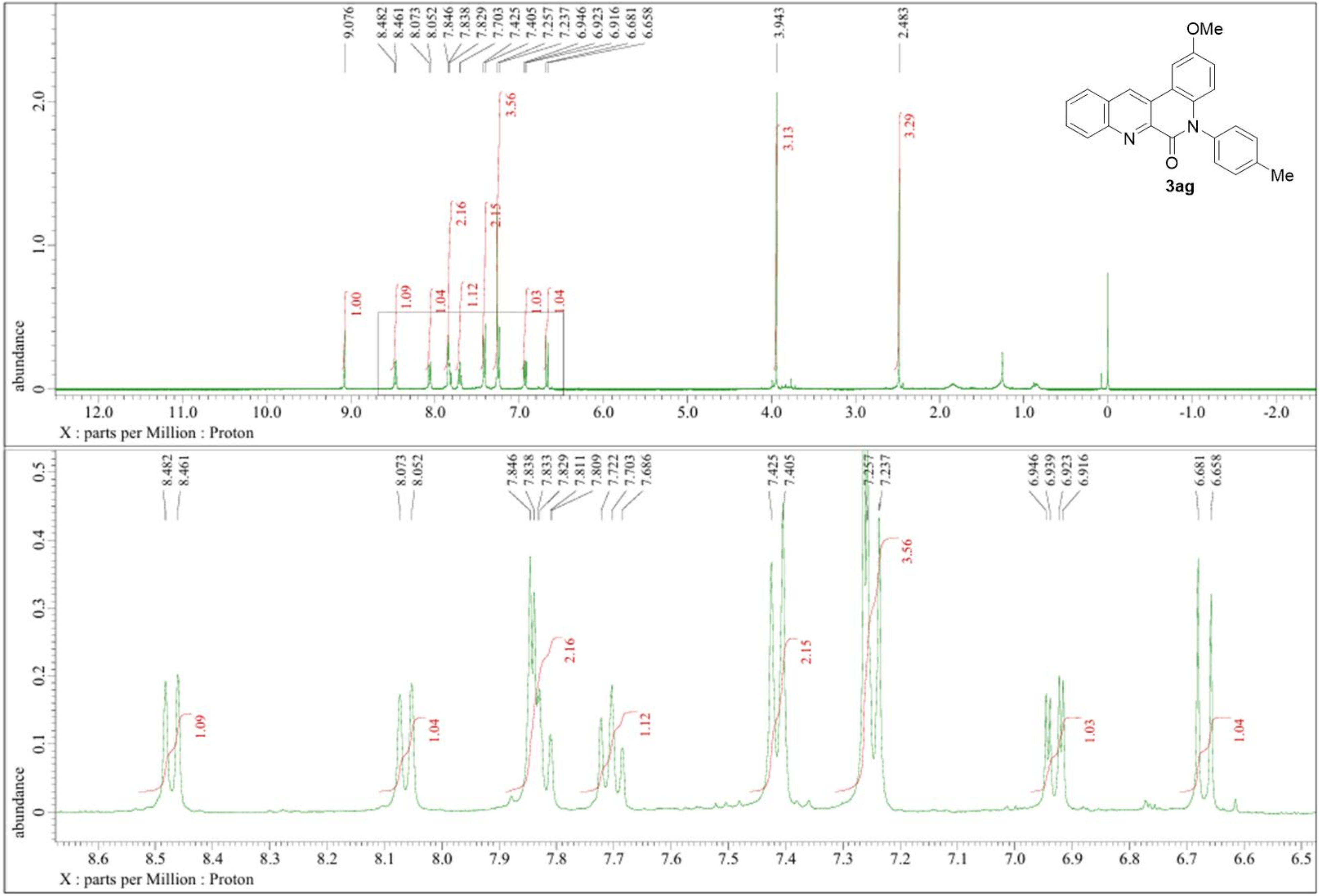

$^{13}C\{^1H\}$ NMR (100 MHz, $CDCl_3$)

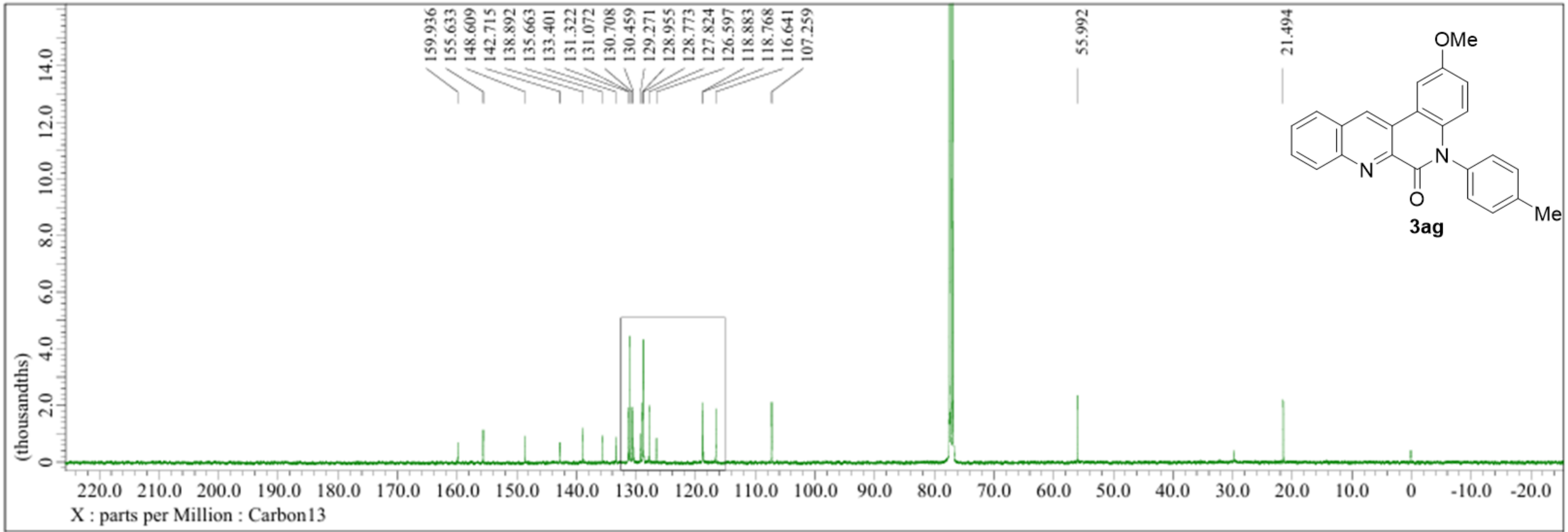

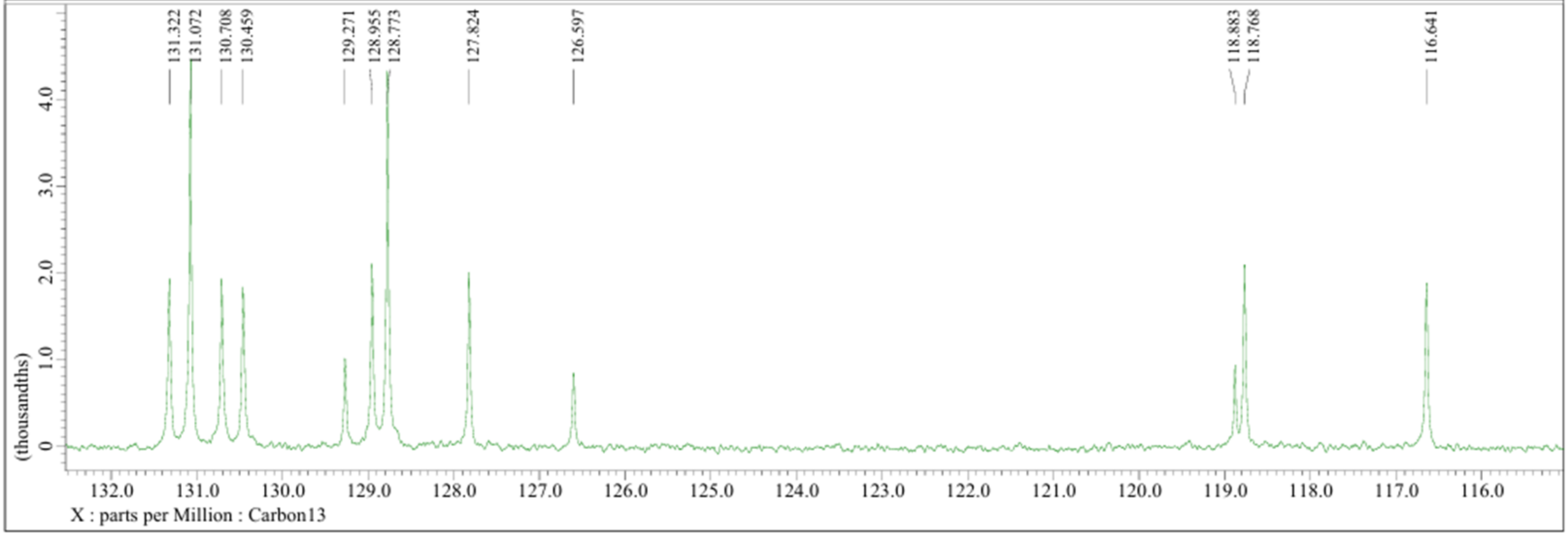

^{1}H NMR (400 MHz, $CDCl_3$)

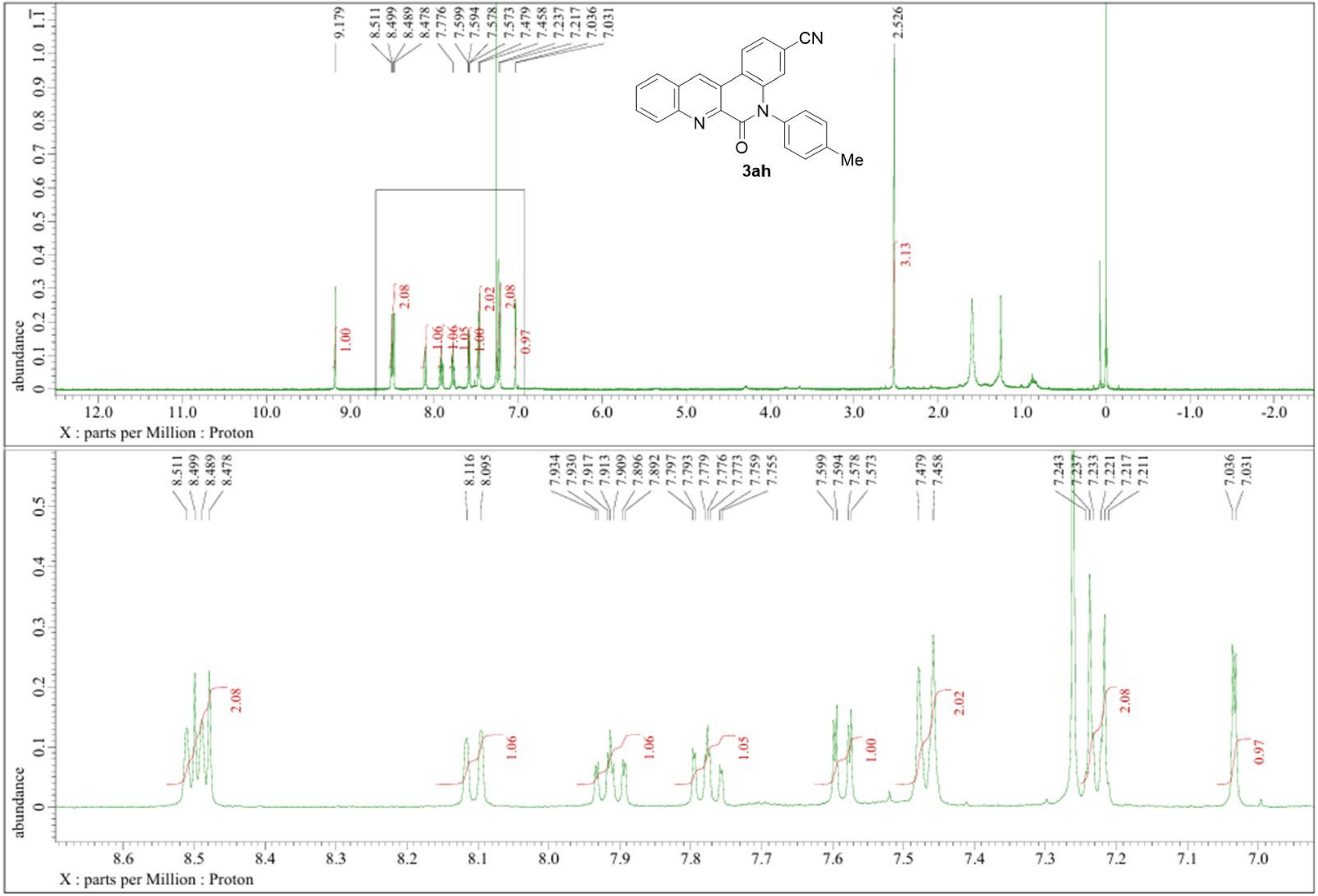

$^{13}C\{^{1}H\}$ NMR (100 MHz, $CDCl_3$)

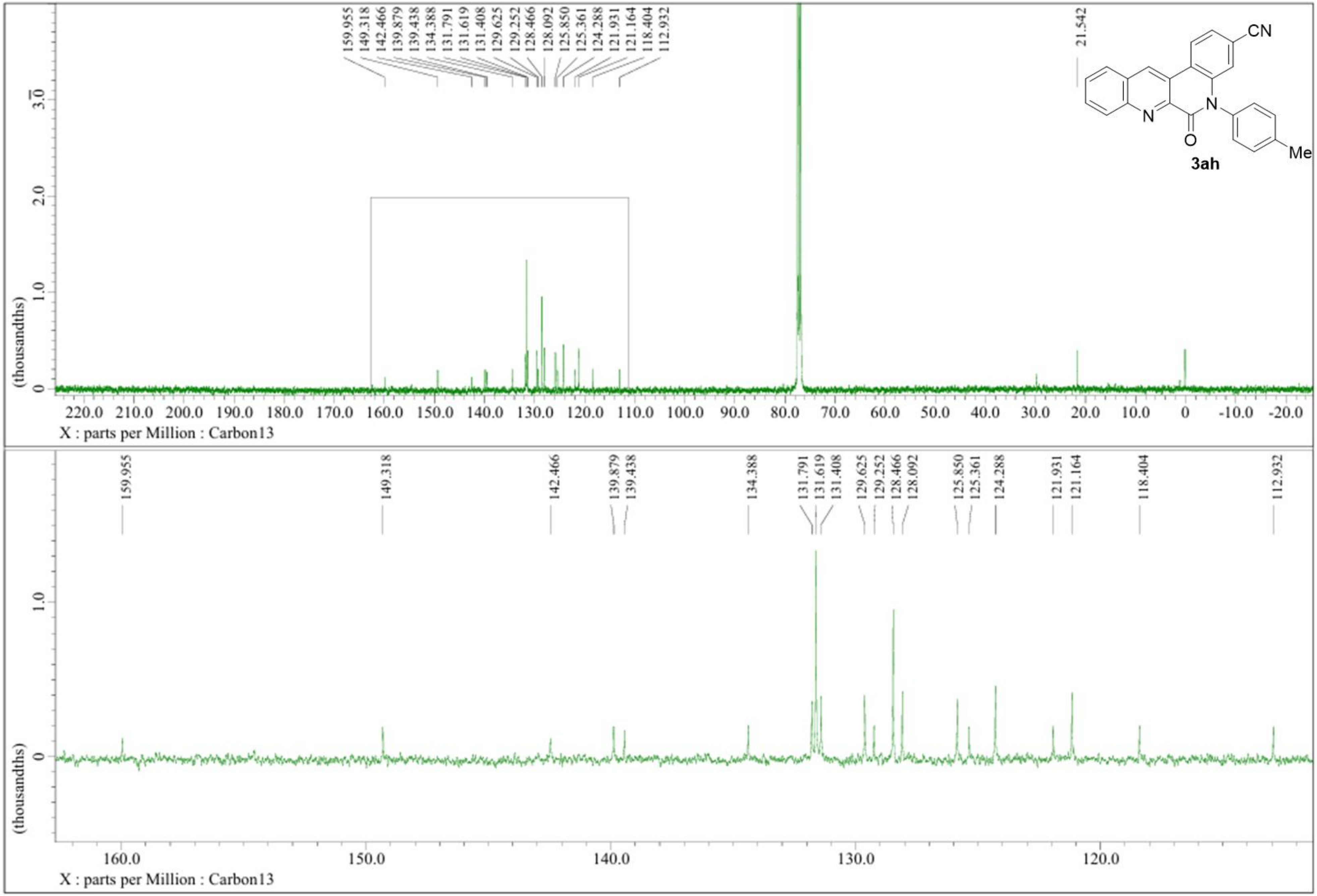

^{1}H NMR (400 MHz, $CDCl_3$)

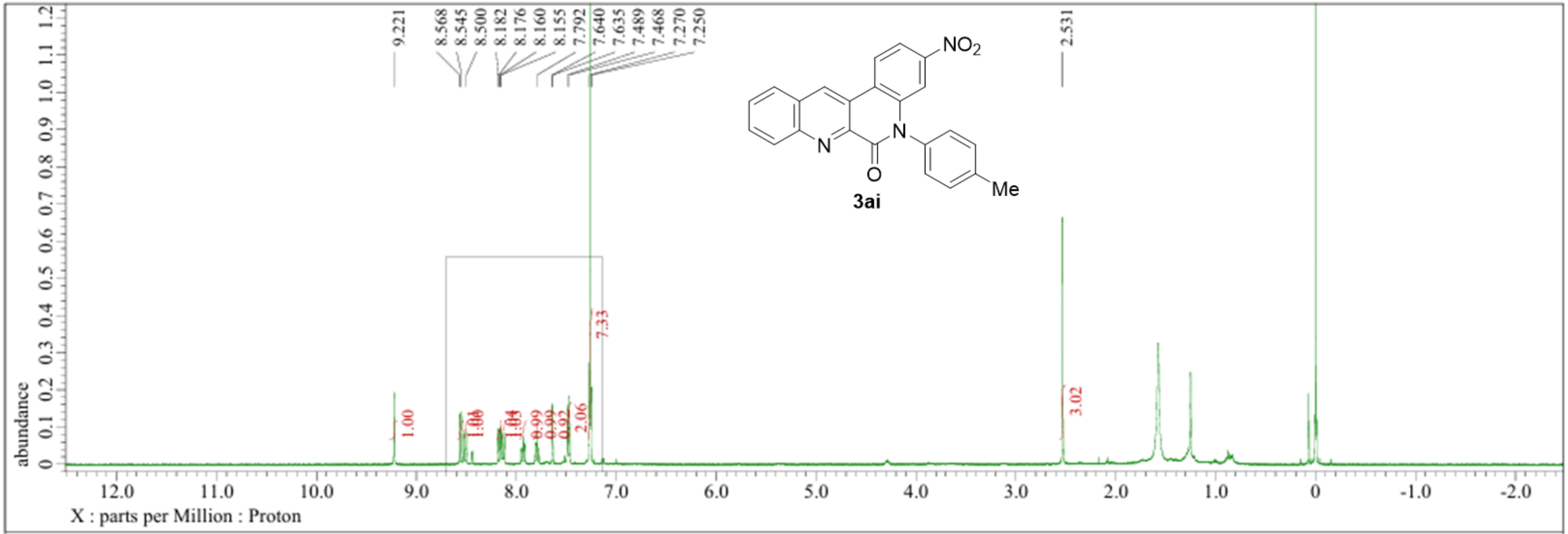

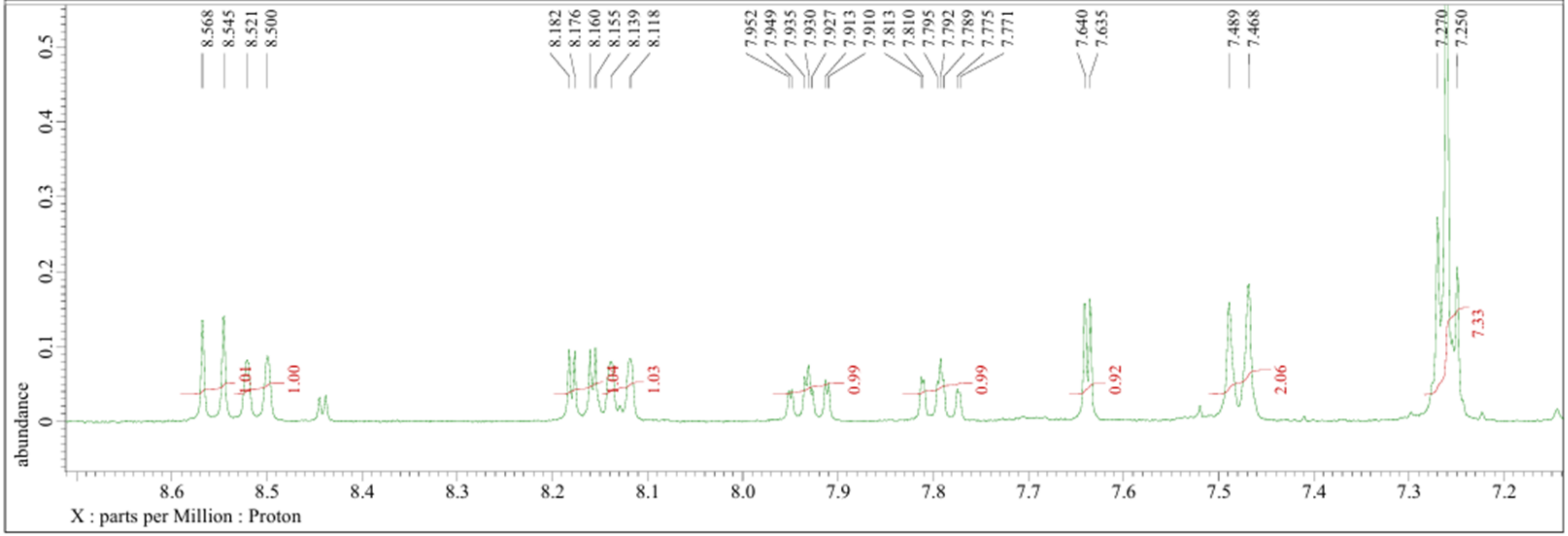

$^{13}C\{^1H\}$ NMR (100 MHz, $CDCl_3$)

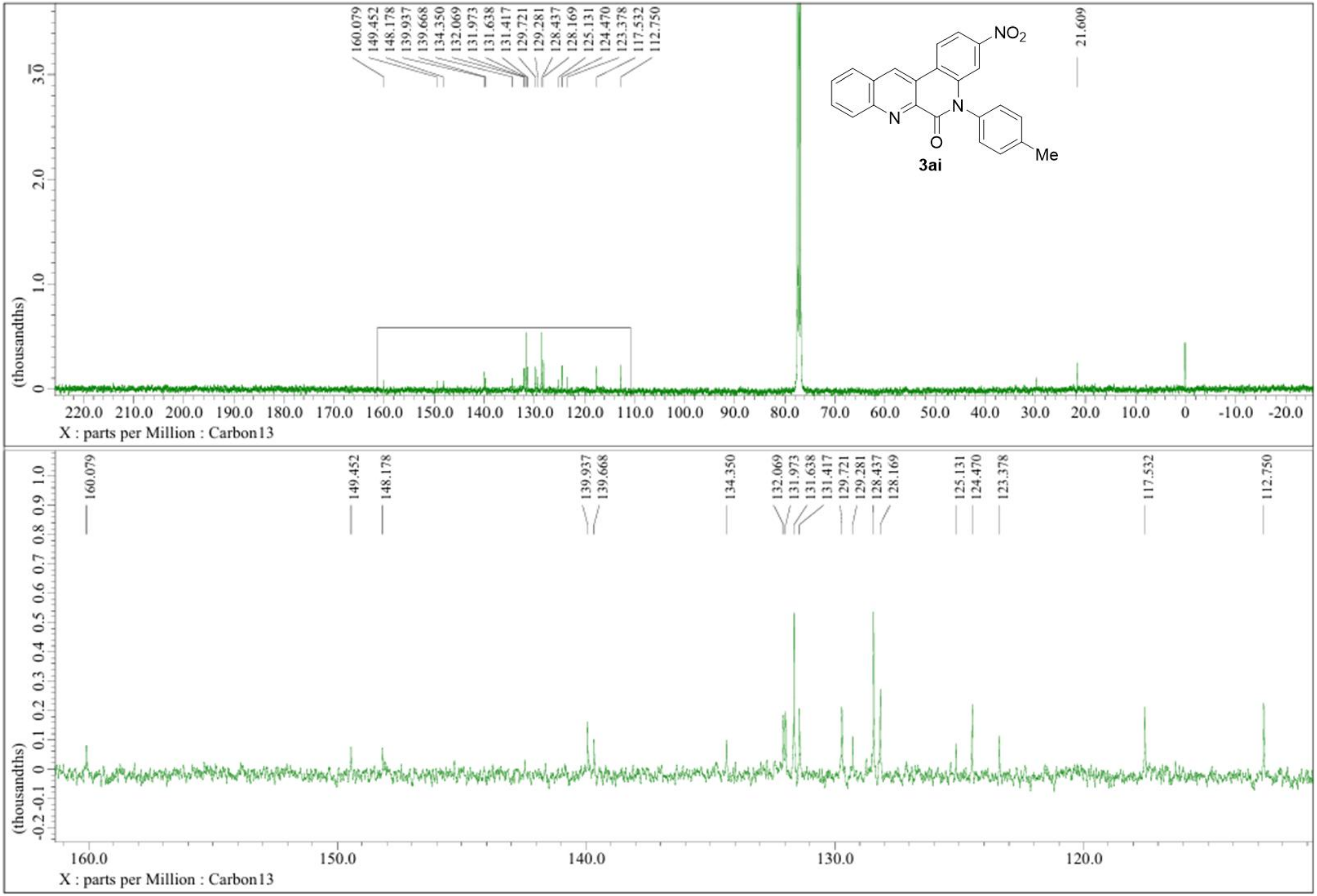

^{1}H NMR (400 MHz, $CDCl_3$)

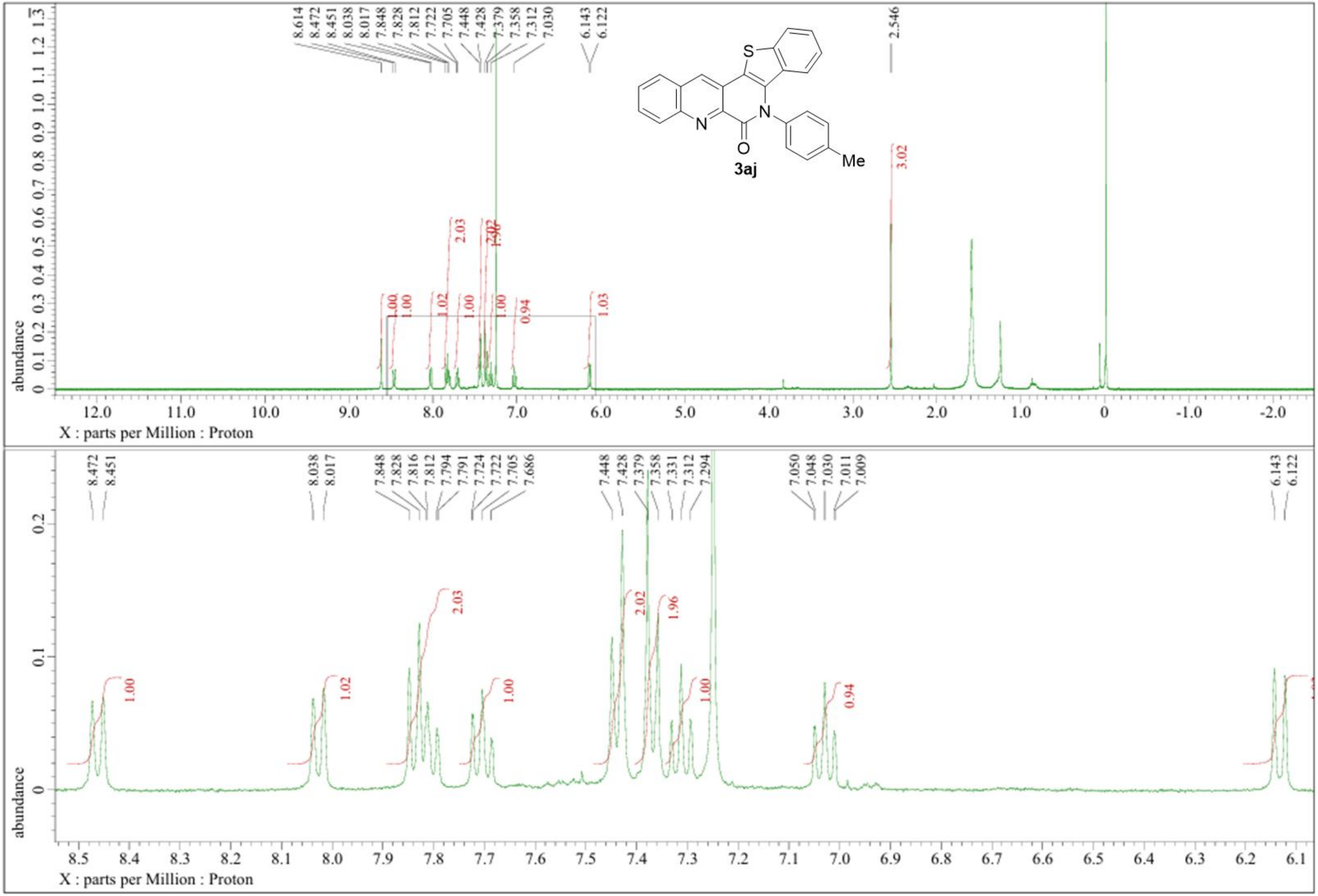

$^{13}C\{^1H\}$ NMR (100 MHz, $CDCl_3$)

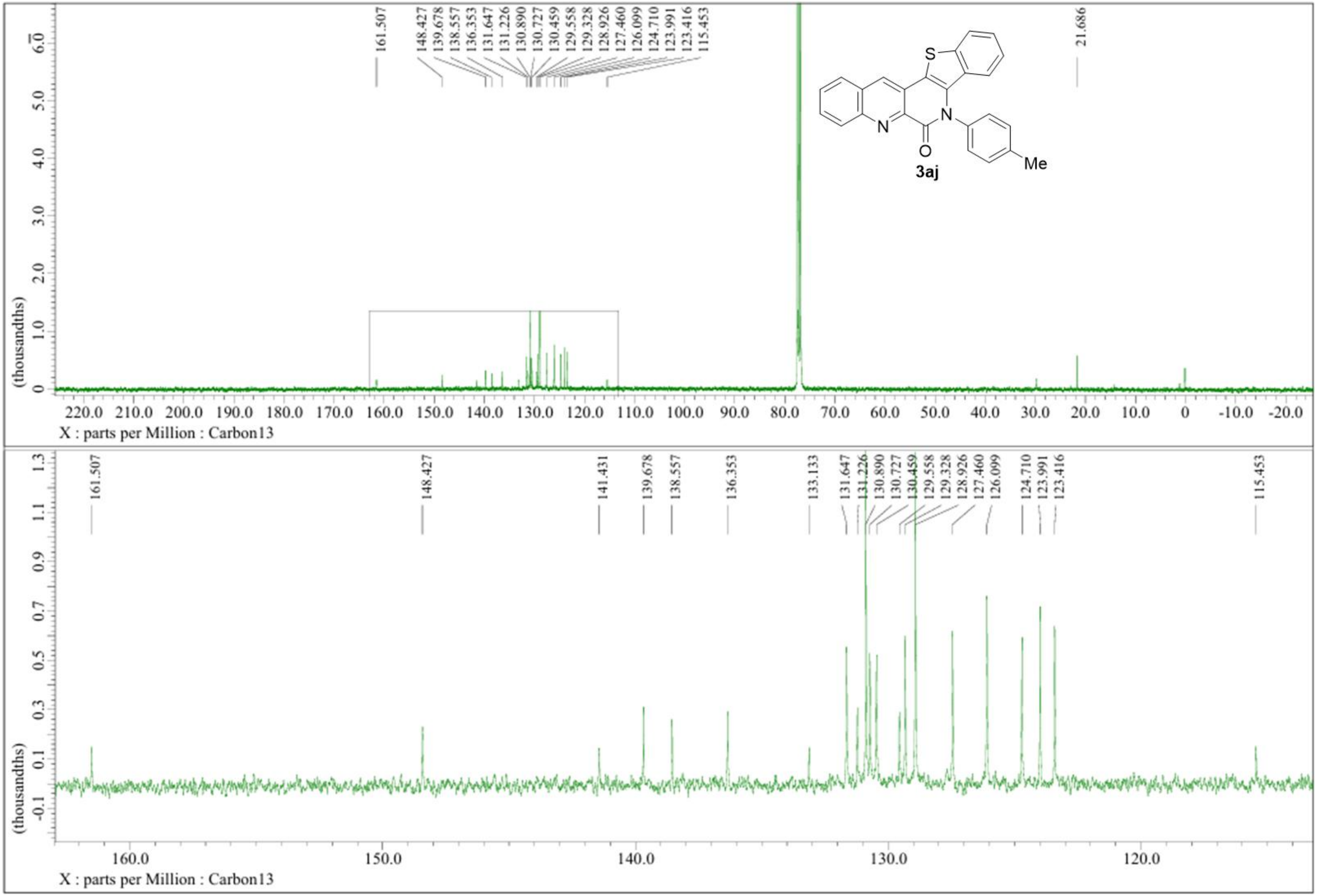

^{1}H NMR (400 MHz, $CDCl_3$)

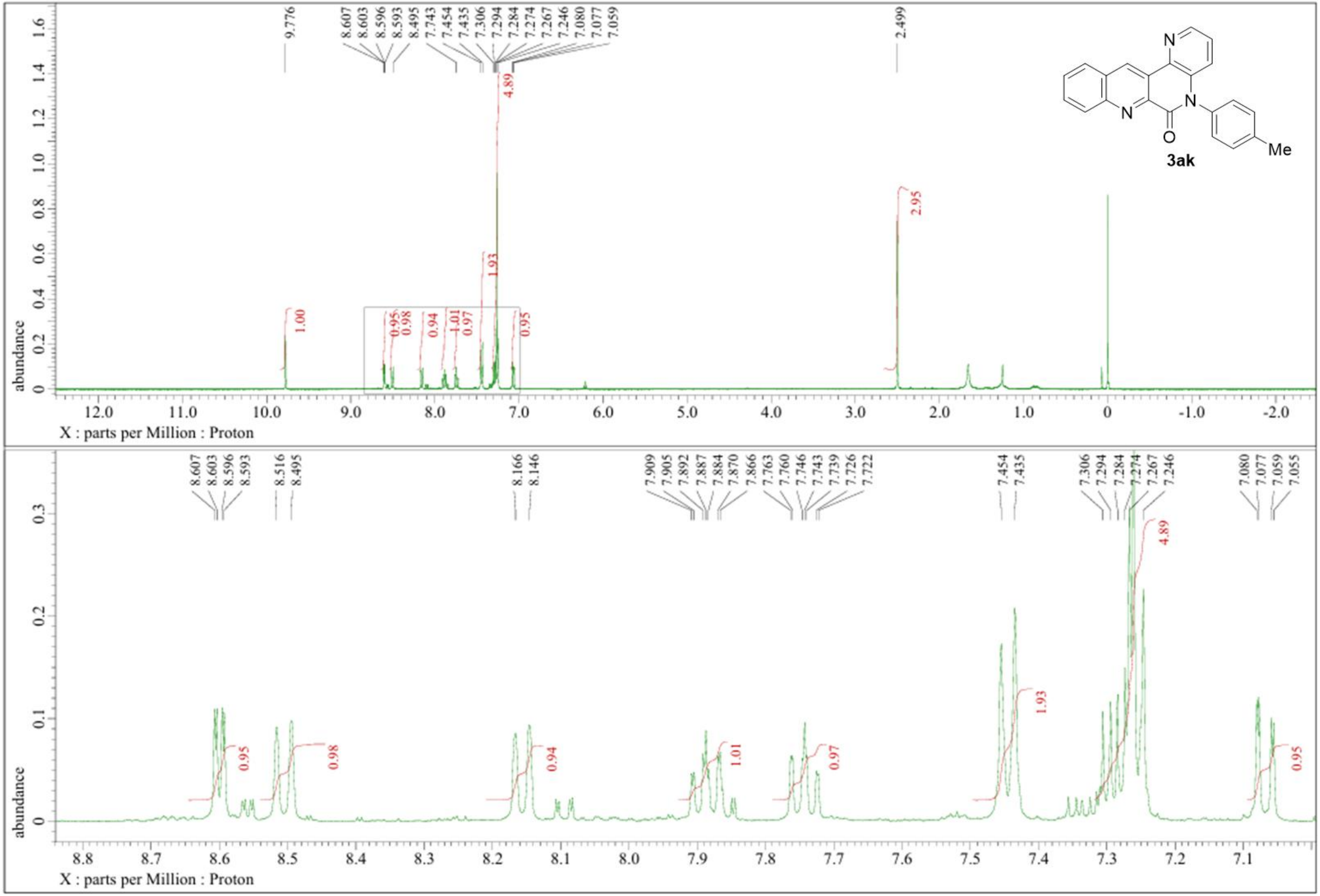

And structural isomer was contained.

$^{13}C\{^{1}H\}$ NMR (100 MHz, $CDCl_3$)

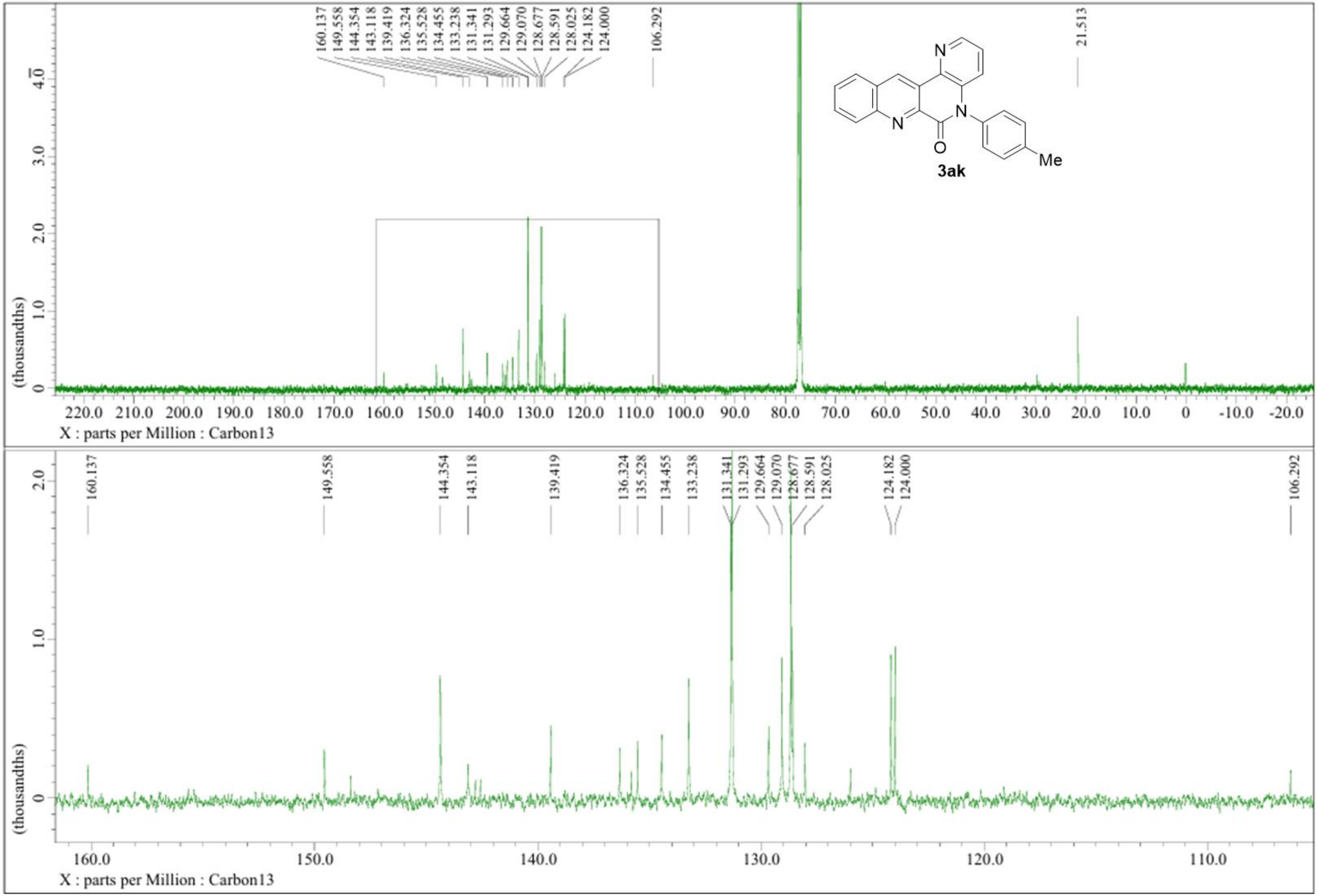

And structural isomer was contained.

References

1) Huang, Y.; Chem, T.; Li, Q.; Zhou, Y.; Yin, S.-F. *Org. Biomol. Chem.* **2015**, *13*, 7289-7293.
2) Xie, H.; Liao, Y.; Chen, S.; Chen, Y.; Deng, G.-J. *Org. Biomol. Chem.* **2015**, *13*, 6944-6948.
3) Mamidi, N.; Manna, D. *J. Org. Chem.* **2013**, *78*, 2386-2396.
4) Gao, Y.; Mao, Y.; Zhang, B.; Zhan, Y.; Huo, Y. *Org. Biomol. Chem.* **2018**, *16*, 3881-3884.
5) Smajlagic, A.; Srabovic, M.; Ademovic, Z.; Pehlic, E.; Huremovic, M.; Huseinovic, E. *J. Chem. Biol. Phys. Sci.* **2021**, *11*, 043-053.
6) Chakrabarty, M.; Ghosh, N.; Khasnobis, S.; Chakrabarty, M. *Synth. Commun.* **2002**, *32*, 265-272.
7) Karthikeyan, J.; Haridharan, R.; Cheng, C.-H. *Angew. Chem. Int. Ed.* **2012**, *51*, 12343-12347.
8) Lee, S. H.; Jang, B.-B.; Kafafi, Z. H. *J. Am. Chem. Soc.* **2005**, *127*, 9071-9078.
9) Shintani, R.; Misawa, N.; Takano, R.; Nozaki, K. *Chem. Eur. J.* **2017**, *11*, 2660-2665.
10) Zhang, J.; She, M.; Liu, L.; Feng, X.; Li, Y.; Liu, H.; Zheng, T.; Leng, X.; Liu, P.; Zhang, S.; Li, J. *Org. Lett.* **2021**, *23*, 8396-8401.
11) Jiang, P.-P.; Yang, X.-J. *RSC Adv.* **2016**, *6*, 90031-90034.
12) Attanasi, O. A.; Berretta, S.; Favi, G.; Filippone, P.; Mele, G.; Moscatelli, G.; Saladino, R. *Org. Lett.* **2006**, *8*, 4291-4293.
13) van Klink, G. P. M.; de Boer, H. J. R.; Schat, G.; Akkerman, O. S.; Bickelhaupt, F.; Spek, A. L. *Organometallics* **2002**, *21*, 2119–2135.
14) Noji, T.; Fujiwara, H.; Okano, K.; Tokuyama, H. *Org. Lett.* **2013**, *15*, 1946-1949.
15) Jensen, T.; Pedersen, H.; Bang-Andersen, B.; Madsen, R.; Jøgensen, M. *Angew. Chem. Int. Ed.* **2008**, *47*, 888-890.
16) Tymann, D. C.; Benedix, L.; Iovkova, L.; Pallach, R.; Henke, S.; Tymann, D.; Hiersemann, M. *Chem. Eur. J.* **2020**, *26*, 11974-11978.
17) Lu, H.-H.; Gan, K.-J.; Ni, F.-Q.; Zhang, Z.; Zhu, Y. *J. Am. Chem. Soc.* **2022**, *144*, 18778-18783.
18) Wegner, H. A.; Reisch, H.; Rauch, K.; Demeter, A.; Zachariasse, K. A.; de Meijere, A.; Scott, L. T. *J. Org. Chem.* **2006**, *71*, 9080-9087.
19) Guo, H.; Wang, Y.; Du, G.-F.; Dai, B.; He, L. *Tetrahedron* **2015**, *71*, 3472-3477.
20) Wan, Y.; Zhang, Z.; Ma, N.; Bi, J.; Zhang, G. *J. Org. Chem.* **2019**, *84*, 780-791.
21) Pawar, G. G.; Brahmanandan, A.; Kapur, M. *Org. Lett.* **2016**, *18*, 448-451.
22) Weiß, A.; Podlech, J. *Eur. J. Org. Chem.* **2019**, 6697-6701.
23) Rousseaux, S.; Liégault, B.; Fagnou, K. *Chem. Sci.* **2012**, *3*, 244-248.
24) Kuwabe, S.; Torraca, K. E.; Buchwald, S. L. *J. Am. Chem. Soc.* **2001**, *123*, 12202-12206.
